# Photodecarboxylation of the Siderophore Aerobactin with the Lewis Acidic Metal Ions Fe(III), Ga(III), and Ti(IV)

**DOI:** 10.1021/acs.inorgchem.5c02471

**Published:** 2025-10-01

**Authors:** Edith K. Amason, Thomas C. Brunold, Eszter Boros

**Affiliations:** Department of Chemistry, 5228 University of Wisconsin-Madison, Madison, Wisconsin 53706, United States

## Abstract

The class of α-hydroxy carboxylic acid-containing ferric siderophore natural products undergo photochemical modification by decarboxylation. To date, there is only limited mechanistic understanding of the metal-ion-mediated photodegradation of photoactive siderophores. This study investigates the photoreactivity of the α-hydroxy carboxylic acid-containing siderophore aerobactin (AB) and the corresponding Ga^3+^ and Ti^4+^ metal complexes in direct comparison with their Fe^3+^-bound counterpart. Using UV–vis and nuclear magnetic resonance (NMR) spectroscopy, complemented by time-dependent density functional theory (TD-DFT) calculations, we demonstrate that ligand-to-metal charge transfer (LMCT)-driven photocleavage of aerobactin–metal complexes is feasible beyond Fe^3^^+^. We show that photoirradiation at shorter wavelengths of [Ga­(AB)]^3–^ and [Ti­(AB)]^2–^ results in decarboxylative photocleavage at two distinct sites. While [Fe­(AB)]^3–^ exhibits distinct reactivity upon photoexcitation from 254 to 575 nm, producing C–C bond cleavage to release CO_2_ and form the corresponding tautomer, the analogous [Ti­(AB)]^2–^ complex can be selectively exited. Lower energy excitation of [Ti­(AB)]^2–^ within the LMCT band centered at 295 nm induces decarboxylation in direct homology with the parent ferric complex, whereas secondary decarboxylation of the lysine carboxylate is observed using short wavelength irradiation of [Ga­(AB)]^3–^ and [Ti­(AB)]^2–^. These experimental results, supported by TD-DFT findings, reveal that the coordinating hydroxamate groups, rather than the α-hydroxy carboxylate, are the source of efficient LMCT excitation and radical formation, challenging previous assumptions about aerobactin’s photochemical decarboxylation mechanism. We provide a mechanistic framework for siderophore-mediated photochemistry and highlight its applicability to xenometal ions.

## Graphical abstract

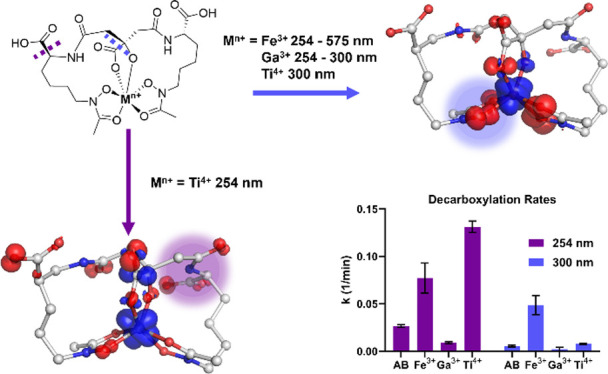

## Introduction

Siderophores are secondary metabolites with high affinity for Fe^3+^, secreted by bacteria under iron-limiting conditions. This class of molecules have been widely explored both in their native state and in the form of synthetic analogues that not only mimic the high binding affinity to Fe^3+^, but also become readily utilized by bacteria to source iron.^1,2^ Once secreted, these molecules capture iron from atmospheric dust, hydrothermal vents, and disturbed sediments, making the otherwise insoluble Fe^3^^+^ bioavailable. The Fe^3^^+^-siderophore complex is then internalized by the bacteria via one of three pathways: (1) competitive displacement by another natural chelator, (2) direct uptake of the intact Fe^3+^-siderophore, or (3) photoreduction.^3,4^

The structural diversity of known siderophores is extensive, incorporating a variety of denticities, ligand donor sets, and overall reactivity (Figure 1a–c). A growing number of photoactive siderophores^3,5–11^ have been identified to undergo selective degradation or chemical conversion in the presence of light (Scheme 1); the role and necessity of these phototransformations remain largely unknown. One hypothesis posits that photoactive siderophores play a crucial role in microbial iron acquisition in marine organisms, where photochemical transformations may enhance bioavailability.^3–6,8–10^ However, as several photoreactive siderophores are produced by nonmarine organisms, this hypothesis is likely not all-encompassing. Hexadentate siderophores such as aerobactin, petrobactin, and staphyloferrin contain an α-hydroxy carboxylate functional group, which is essential for photoredox activity (Figure 1d–f) producing the corresponding decarboxylated, hexadentate keto–enol tautomer (Scheme 1).^4^ Previous mechanistic studies hypothesized^3,4,9–11^ that the photodegradation mechanism involves a ligand-to-metal charge transfer (LMCT), where an electron is transferred from the α-hydroxy carboxylate to the Fe^3^^+^ metal center, forming Fe^2+^ which dissociates from the siderophore due to a loss of binding affinity.^4^ This process generates a radical carboxylate species, which undergoes C–C bond cleavage, releasing CO_2_. Finally, Fe^3+^ recoordinates to form the hexacoordinate product that has been readily identified using mass spectrometry.^12–16^

**Scheme 1:**
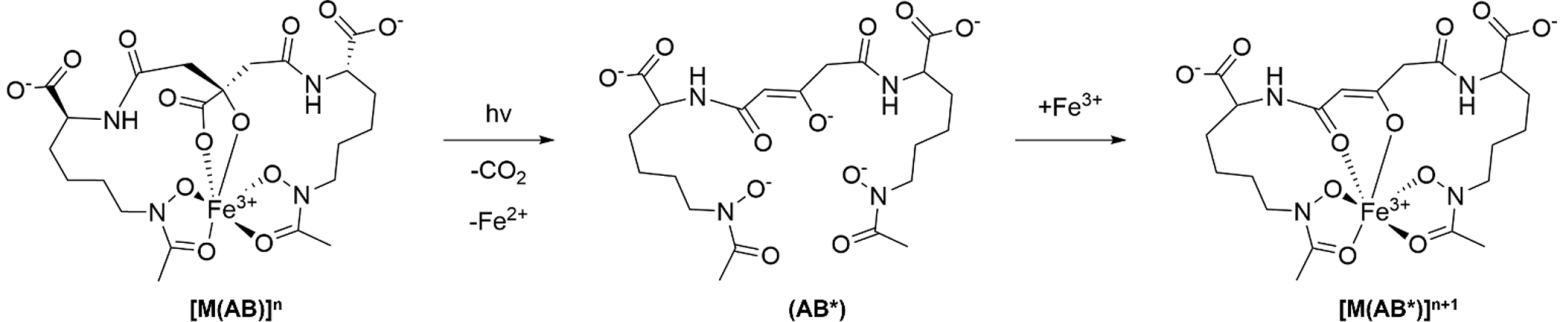
Postulated Photoredox Mechanism of Central Citrate-Based Siderophores, Specifically Aerobactin, Bound to Fe^3+^

**Figure 1:**
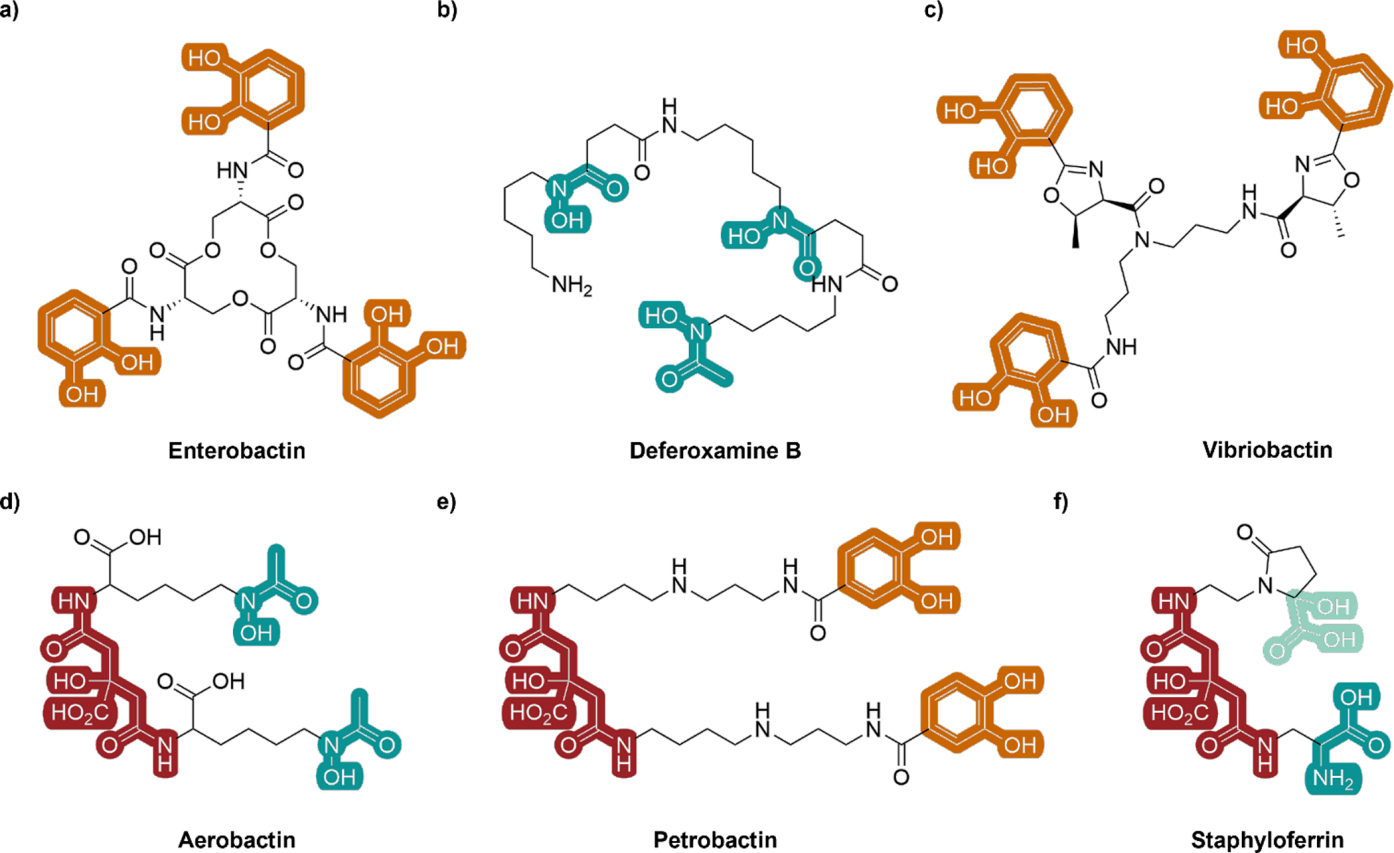
Examples of native photoinert siderophores (a–c) and photoactive siderophores (d–f). Red: moiety derived from citrate; orange: catechol; teal: hydroxamate; light teal: α-hydroxy carboxylate.

Aerobactin (AB) represents a structurally and biologically unique, photoactive siderophore produced by various (predominantly pathogenic) *Enterobacteriaceae*, such as *Escherichia coli*, *Klebsiella pneumoniae*, and *Yersinia pseudotuberculosis* that dwell in mammalian hosts.^17–22^ Unlike simple citrate complexes, AB possesses a symmetric structure containing a citrate based backbone and two terminal hydroxamate moieties (Figure 1d). This rigid ligand framework enables Fe^3^^+^ coordination in an octahedral geometry with a *K*_ML_ of 23.06 as determined by potentiometric titration, which not only enhances complex stability, but also facilitates efficient photoactivation and subsequent decarboxylation.^4,7,8,23–25^

Previous studies have successfully demonstrated the formation of Fe^3^^+^-AB complexes and their subsequent photoproducts following light irradiation; however, the mechanism of this transformation remains elusive. While an LMCT is widely accepted as the initial excitation step, several key aspects of the reaction pathway require further elucidation: (1) although the α-hydroxy carboxylate is hypothesized to be the primary site of radical initiation, it is unclear whether secondary organic radical pathways or metal-mediated electron transfer play a role in directing the bond-cleavage event. While computational methods, including density functional theory (DFT) and time-dependent DFT (TD-DFT), have been employed to model charge-transfer transitions and photoproduct stability, experimental validation of these predictions remains absent.^4,8,23,24^ (2) The initially proposed intermediate step of demetalation, followed by remetalation appears counterintuitive considering the affinity for Fe^3+^ remains high and the photoproduct is also efficiently internalized.^8^

Furthermore, the potential for AB to chelate metal ions besides Fe^3^^+^ has yet to be systematically explored. Numerous studies have demonstrated that siderophores can bind many Lewis acids, including Ga^3^^+^, Ti^4^^+^, and Zr^4^^+^, expanding their functional role beyond iron acquisition. Given the chemical similarities between Fe^3^^+^, Ga^3+^, and Ti^4^^+^, such as their comparable ionic radii (Fe^3^^+^ = 0.645, Ga^3+^ = 0.620, Ti^4^^+^ = 0.605 Å)^26^ and high charge densities, Ga^3+^ and Ti^4^^+^ can effectively substitute for Fe^3^^+^ in siderophore coordination.^27–34^ However, the photochemical consequences of this substitution remain unclear, as the excitation of the short-wave LMCT band of corresponding Ti^4+^ complexes has not been explored.

In this work, we investigate the photochemical properties of AB and the corresponding coordination complexes formed with Fe^3+^, Ga^3^^+^, and Ti^4^^+^. A wavelength-dependent analysis across the UV and visible spectrum utilizing various spectroscopic tools provides insight into selective excitation pathways, revealing thus far unknown photodecarboxylation at shorter wavelengths without the involvement of Fe^3+^. TD-DFT calculations were employed to provide additional support to the interpretation of our experimental findings.

## Results and Discussion

### Coordination Chemistry of the [M^*n*+^-Aerobactin] Complexes

The Fe^3+^ complex of AB was formed by the addition of a metal salt stock solution to a ligand stock solution in MeOH under neutral conditions. In congruence with the previously reported aqueous speciation data found in the literature, we assume that this species is predominantly found as the MH_–1_L complex above pH 5 and the MH_–2_L above pH 7.^7,8^ Furthermore, based on the chemical similarities of the Ti^4+^ and Ga^3+^ metal ions to Fe^3+^ we anticipate that the same speciation trends remain true. Under the same neutral conditions, the Ti^4+^ complex was observed to form readily.^35^ This suggests that the central α-hydroxy carboxylate enhances complex stability, in contrast to the softer hydroxamate oxygens. In our previous work on Ti^4+^ chelation, we observed improved stabilization of Ti complexes across a broad pH range by increasing the coordinating ligand’s basicity. Indeed, the p*K*_a_ of the citril/ α-hydroxide is estimated around 11, above the p*K*_a_ of the hydroxamate and catechol protons (8.9 and 9.3, respectively).^7,35^ As expected, Ga^3+^ also forms a 1:1 M-AB complexes, consistent with extensive literature on the strong interactions of this metal with hydroxamate-based chelators, particularly the nonphotoactive siderophore deferoxamine B (DFOB).^27,28,32,36,37^ NMR characterization data, particularly ^13^C data, indicate that that complexes form multiple isomers in solution (Scheme S2). Paramagnetic and multidimensional NMR spectra of the [Fe­(AB)]^3–^ complex affirm findings by Raymond and co-workers^7^ that two isomers are in equilibrium, annotated on the spectra where relevant (Figure 2 and Table 1). Variable temperature NMR data show no appreciable difference for the chemical shifts of [Fe­(AB)]^3–^, indicating that across 0–40 °C in MeOD-*d*_4_ no isomerization occurs, although it is likely that two isomers exist in solution (Figures 2 and S39). However, for both the [Ga­(AB)]^3–^ and [Ti­(AB)]^2–^ complexes under the same conditions, 0–40 °C in MeOD-*d*_4_, the signals of the α-protons corresponding to g, m around δ = 4.5 ppm appear to be merging into one peak as the temperature increases (Figures S40 and S41). Additionally, the citryl peaks in the region around δ = 2.75 ppm for the [Ga­(AB)]^3–^ complex possess a smaller *J*-coupling relative to the apo-aerobactin, whereas the *J*-coupling for the same peaks seems to increase for the isomers of [Ti­(AB)]^2–^ (Figures 2, S40, and S41). Table 1 provides a detailed assignment of chemical shifts; Figure 2 shows representative ^1^H NMR spectra for the aerobactin ligand and the corresponding [M­(AB)] complexes.

**Figure 2:**
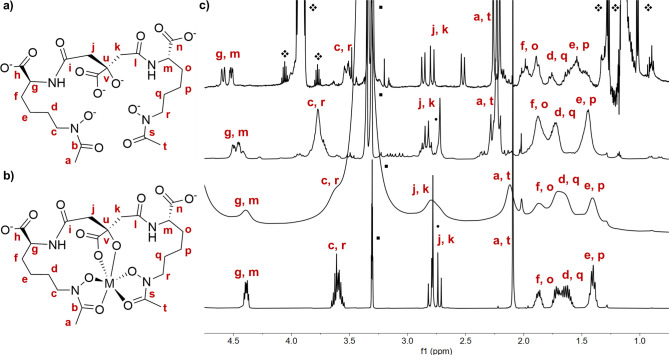
Chemical structure and atom labeling for aerobactin (a) and the corresponding metal complexes (b). (c) Bottom to top: ^1^H NMR spectra of aerobactin, [Fe­(AB)]^3–^, [Ga­(AB)]^3–^, [Ti­(AB)]^2–^. Residual solvent and complex precursor signals (iPrO, MeOD, and residual DMSO) are annotated.

**Table 1:** ^1^H-NMR Signal Assignments Reported as Their Chemical Shift (ppm) in MeOD-d_4_ for Apo-Aerobactin and the Corresponding Metal Complexes^a^

Assignment	Aerobactin (AB)	[Fe(AB)]^3–^	[Ga(AB)]^3–^	[Ti(AB)]^2–^
g, m	4.43–4.35 (2H, dt)	4.57–4.23 (2H, m)	4.51–4.41 (2H, m)	4.62–4.55 (1H, d)
				4.55–4.49 (1H, dd)
c, r	3.65–3.55 (4H, dq)	3.66–3.61 (4H, m)	3.81–3.73 (4H, qd)	3.56–3.49 (4H, d)
j, k	2.84–2.69 (2H, dd)	2.90–2.67 (4H, m)	2.89–2.78 (2H, dd)	2.89–2.49 (3H, dd)
				2.81–2.76 (1H, d)
a, t	2.10–2.09 (6H, s)	2.19–2.06 (6H, s)	2.25–2.22 (6H, s)	2.27–2.25 (3H, s)
				2.21–2.20 (3H, s)
f, o	1.92–1.34 (12H, m)	1.96–1.32 (12H, m)	1.92–1.43 (12H, m)	2.05–1.44 (12H, m)
d, q				
e, p				

a For detail on coupling constants, see the Supporting Information.

### Structural Modeling Using Computational Tools

In the absence of obtainable crystal structures, computational methods can be used to generate three-dimensional models of the different M­(AB) complexes and to support hypotheses arising from experimental data. While a previous study has characterized the [Fe­(AB)]^3–^ isomers using TD-DFT, we have expanded the computational analysis to match our experimental conditions and the metal complexes investigated.^24,38,39^

We used DFT to generate energy-minimized structures of the [Fe­(AB)]^3–^, [Ga­(AB)]^3–^, and [Ti­(AB)]^2–^ complexes and their putative photoproducts, providing a direct comparison of anticipated structural changes during photoirradiation. Figure 3 shows an overlay of the most energetically favored structural isomers for all three complexes, indicating that the three hexacoordinate complexes exhibit close structural homology to the native [Fe­(AB)]^3–^ and [Fe­(AB*)]^2–^ complexes, respectively (root-mean-square deviation (RMSD): [Ga­(AB)]^3–^ = 0.070, [Ti­(AB)]^2–^ = 0.125, [Ga­(AB*)]^2–^ = 0.103, [Ti­(AB*)]^1–^ = 0.232). For consistency, we adopted the established nomenclature for geometric isomers, where “C” refers to coordination via the carbonyl oxygen of the hydroxamate and “N” refers to coordination through the hydroxylamine oxygen (Scheme S1). Computed relative energies identify the *cis–trans C-mer* configuration as the lowest-energy isomer across all metal complexes and apo-aerobactin. For all complexes, the relative energy differences between the *cis–cis C-fac* and *cis–trans N-mer* isomers were consistently near 200 kcal/mol, reinforcing that the *cis–trans C-mer* configuration is significantly more stable; we posit that the *cis–cis C-fac* is the second isomer observed in the corresponding NMR spectra, indicating the fluxional nature of the coordination complex at room temperature.

**Figure 3:**
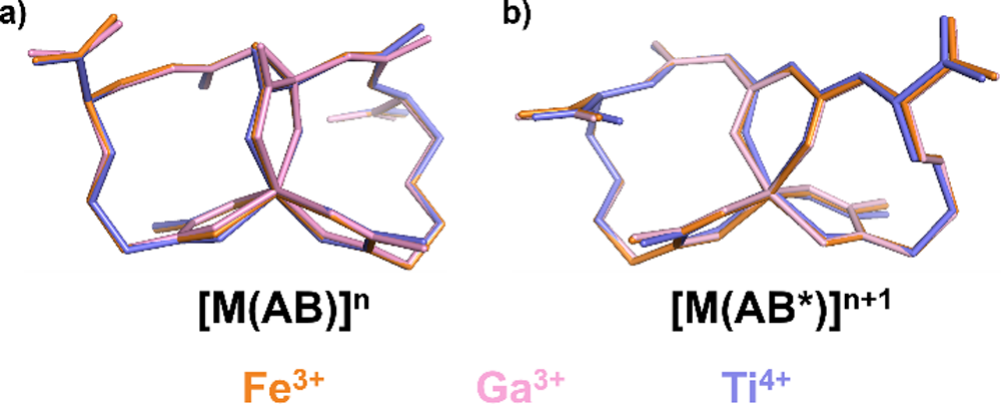
Structural overlay of the *cis–trans C-mer* isomers of (a) [M­(AB)]^n^ and (b) [M­(AB*)]^n+1^.

UV–vis spectral data collected on the complex shows the presence of the characteristic LMCT transition (λ_max_ = 399 nm) for the orange [Fe­(AB)]^3–^ complex in pH 8 seawater versus the same LMCT transition (λ_max_ = 398 nm) reported at pH 7, as characterized previously by Butler and co-workers and Raymond an co-workers, respectively.^7,8^ However, in MeOH, the same LMCT transition was observed to be blue-shifted (λ_max_ = 362 nm). A second, characteristic absorption feature at 520 nm was observed uniquely for [Fe­(AB)]^3–^ due to the complex’s electronic configuration providing an additional LMCT transition.^7^ The colorless [Ga­(AB)]^3–^ complex lacks these features due to its d^10^ electronic configuration and displays a single absorption band at 225 nm (Figures S1–S4). In contrast, the corresponding yellow [Ti­(AB)]^2–^ complex exhibits a characteristic LMCT band at 295 nm.

The photoproducts were modeled computationally in their enol form. Unlike their AB counterparts, the photoproduct complexes did not exhibit a uniform geometric preference. Among metal-bound photoproducts, the Ga^3^^+^ complex exhibited a slight 3–6 kcal/mol preference for the *cis–cis C-fac* isomer relative to both the *cis–trans* isomers. Conversely, the Ti^4^^+^ and Fe^3^^+^ bound photoproducts favored the *cis–trans C-mer* isomer (Figure 3b, Tables S5, and S6).

### Computed Absorption Spectra

To assign the experimentally observed absorption features, TD-DFT was used to compute absorption spectra for all geometric isomers and overlaid with their respective experimental absorption traces (Figures S61 and S62). Comparisons confirmed that the most thermodynamically favorable isomer exhibited electronic transitions closely matching those observed experimentally. The computed absorption spectrum of [Ga­(AB)]^3–^ is dominated by a hydroxamate-centered electronic transition at ∼220 nm (Figure 4b). For [Fe­(AB)]^3–^ and [Ti­(AB)]^2–^, numerous additional transitions are computationally predicted and experimentally observed, since in these complexes LMCT transitions involving the partially filled or empty metal 3d-based molecular orbitals (MOs) are now possible. Based on our TD-DFT results, the main contributor to the large increase in absorption below 300 nm in the spectrum of the [Fe­(AB)]^3–^ complex can be attributed primarily to a hydroxamate → Fe^3+^ LMCT transition, while the absorption band centered at ∼400 nm can be assigned to a α-hydroxy carboxylate→Fe^3+^ LMCT transition (Figure 4a). In the absorption spectrum of the [Ti­(AB)]^2–^ complex, a hydroxamate → Ti^4+^ LMCT transition is the main contributor to the prominent band centered at ∼295 nm. The significant red-shift of the hydroxamate → metal LMCT transition from [Fe­(AB)]^3–^ to [Ti­(AB)]^2–^ correlates well with the increased Lewis acidity of Ti^4+^ compared to Fe^3+^.

**Figure 4:**
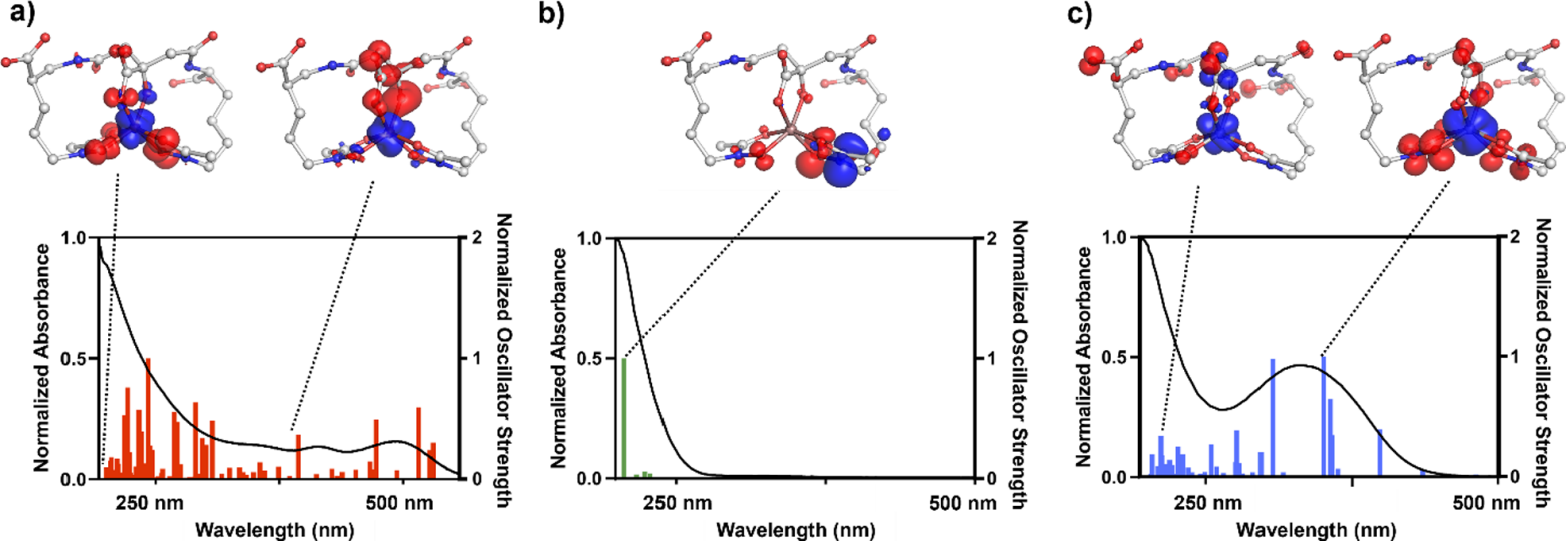
UV–vis spectra and TD-DFT computed excited states for the respective metal complex (a) [Fe­(AB)]^3–^, (b) [Ga­(AB)]^3–^, and (c) [Ti­(AB)]^2–^. Relevant excitations are shown at the top, with blue and red indicating gain and loss of electron density, respectively. The computational spectra were uniformly red-shifted by 6000 cm^–1^ prior to conversion to nm to ensure the best spectral overlay with the experimental spectra in accordance with literature precedent.^38,39^

### Wavelength-Dependent Photoirradiation Experiments

The photochemistry of [Fe­(AB)]^3–^ has been previously established by Butler and co-workers, and investigated in both natural sunlight and with a 450 W mercury lamp with broad spectrum emission bands, indicating that the characterized photocleavage could be wavelength dependent in a discrete environment.^8^ Due to the significance of the LMCT band, which can be used for selective photoexcitation to induce the decarboxylative bond cleavage, we proposed that coordination complexes lacking the ability to undergo ligand-to-metal charge transfer should remain inert to photodegradation. Indeed, a previous study investigating the Ga^3+^ complex of petrobactin indicated that the corresponding complex remained inert in the presence of natural sunlight.^5^ We conducted wavelength-dependent irradiation studies on apo-aerobactin and the corresponding Fe^3^^+^, Ga^3^^+^, and Ti^4+^ complexes. Unlike previous studies that relied solely on natural sunlight, our approach systematically examined the effects of discrete UV wavelengths: such as 254, 300, 350, 420, and 575 nm. For the [Fe­(AB)]^3–^ complex, the different irradiation wavelengths result in different photocleavage rates that correlate to available absorption features (Figure 5a–c). To determine if the starting material had fully converted to the photoproduct, solutions were irradiated at the respective wavelength until no change to the UV–vis spectrum was observed. Subsequent studies utilizing mass spectrometry, which are identified addressed in a later section, revealed that there is an initial conversion to the photoproduct (AB*). Following AB* formation if photoirradiation continues, there is a complete breakdown into small molecule fragments which is observed as the loss of absorbance by UV–vis. Notably, the rate of photocleavage is dependent on the irradiation wavelength’s ability to match an electronic transition of the [Fe­(AB)]^3–^ complex. Figure 5b highlights the feature in the UV–vis spectrum of the [Fe­(AB)]^3–^ complex that each irradiation wavelength targets. At a valley between two excited states, the photocleavage rate is observed to decrease relative to the fastest rate observed at 254 nm. Specifically at 350, 420, and 575 nm the rates of degradation are lower than both that of 254 and 300 nm where absorbance is higher.

**Figure 5:**
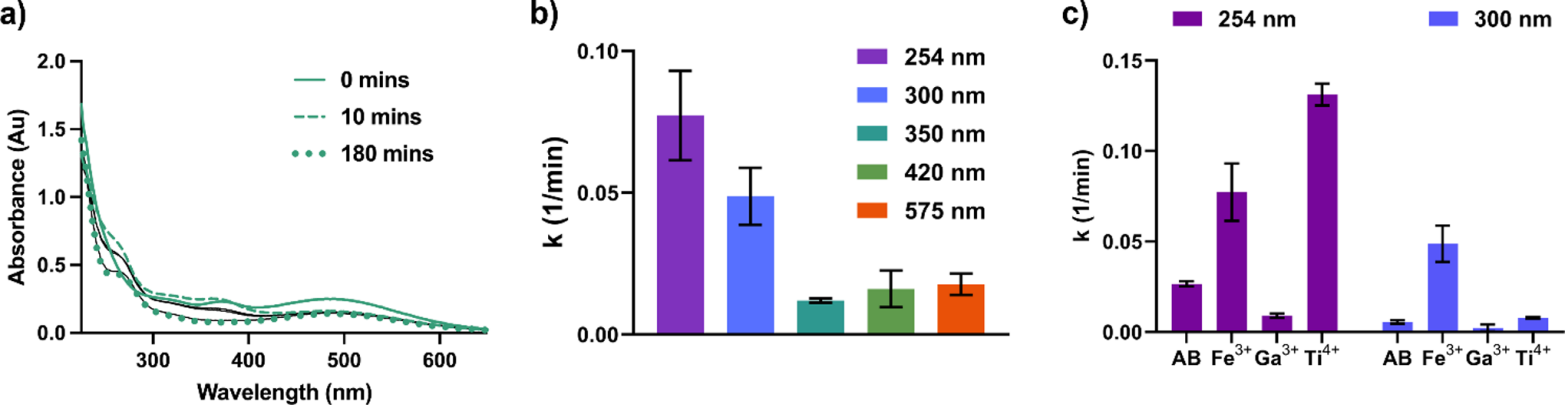
Wavelength-dependent photocleavage. (a) Representative UV–vis spectra for 350 nm irradiation of [Fe­(AB)]^3–^ with the starting material shown as a solid line, photoproduct shown as dashed line, and degradation product shown with dotted line, (b) wavelength-dependent photodegradation rates of [Fe­(AB)]^3–^, and (c) photochemical degradation rates of the M^*n*+^-AB complexes.

Figure 5d summarizes the rates of degradation for apo-aerobactin and all metal complexes at 254 and 300 nm. At 254 and 300 nm irradiation, all metal–aerobactin complexes, as well as apo-aerobactin, underwent photodegradation (Figures S49 and S50). Corresponding UV–vis spectra indicate the formation of a transient photodegradation species with a prominent absorption feature at 225 nm in all instances. As excitation wavelengths extend further into the visible region, photodegradation is no longer observed in non-native metal–aerobactin complexes as well as apo-aerobactin (Figures S51–S53). At 254 nm irradiation, the substitution of [Fe­(AB)]^3–^ for [Ti­(AB)]^2–^ increases the reaction rate, indicating that the Ti^4+^ ion could lower the energy needed for photochemical conversion. Alternatively, the photochemical conversion of the [Ga­(AB)]^3–^ complex is significantly slowed in comparison to both the alternative metal complexes as well as the apo-aerobactin. Upon 300 nm irradiation, [Ti­(AB)]^2–^ and apo-aerobactin undergo an appreciable reduction in photodegradation rate whereas the [Fe­(AB)]^3–^ and [Ga­(AB)]^3–^ complexes display little to no change in the rate of photodegradation. These findings have highlighted the need for a better and more comprehensive understanding of the reaction mechanism responsible for the decarboxylation of aerobactin.

A shift in excitation wavelength from 300 to 350 nm results in photodegradation of the [Fe­(AB)]^3–^ complex only, while all other metal complexes remain stable over a 3-h irradiation period, indicating wavelength-dependent reactivity. At 420 and 575 nm, [Fe­(AB)]^3–^ is also the sole complex that shows photodegradation, as only this complex exhibits absorption features in this energy range (Figure 5c), while all other metal complexes remain unreactive as they lack charge transfer excitations at wavelengths above 330 nm.

### Species Identification

Characterization techniques, including nuclear magnetic resonance (NMR) spectroscopy and liquid chromatography–mass spectrometry (LC-MS), were systematically optimized to monitor photoproduct formation in real time. This information paired with the data acquired by UV–vis and computational analysis enabled the identification of photoproducts. In analogy to previous reports, we observe the formation of the [Fe­(AB*)]^2–^ complex following irradiation at all tested wavelengths. Monitoring by LC-MS after irradiation at both 254 nm (Figure 6d) and 300 nm (Figure 7d) shows the clear transition from [Fe­(AB)]^3–^ at 618 *m*/*z* to [Fe­(AB*)]^2–^ at 572 *m*/*z*, which agrees with the photoproduct formation (Figure 6d) monitored by NMR spectroscopy. Notably, quaternary carbon resonances could not be resolved, even following extended spectrum acquisition, to the same extent as the corresponding diamagnetic gallium complex. Evidence for the formation of the [Ga­(AB*)]^2–^ complex is provided by LC-MS data obtained following irradiation at both 254 nm (Figure 6e) and 300 nm (Figure 7e). Figure 6e shows that the photoproduct readily forms with Ga^3+^, but to maintain the preferable coordinatively saturated complex, the photoproduct carbonyl oxygen that is weakly bound is displaced by a hydroxide group forming [Ga­(AB*)­(OH)]^3–^. While LC-MS monitoring at 300 nm does not display AB* formation after 2 h, NMR data paired with UV–vis data show the formation of a photoproduct that supports the {Ga­(AB*)­(OH)]^3–^ assignment (Figure 7e,h). The spectral data obtained prior to photoirradiation further supports that the structural changes occur as a consequence of the photoirradiation, and not due to fragmentation during mass spectrometric analysis.

**Figure 6:**
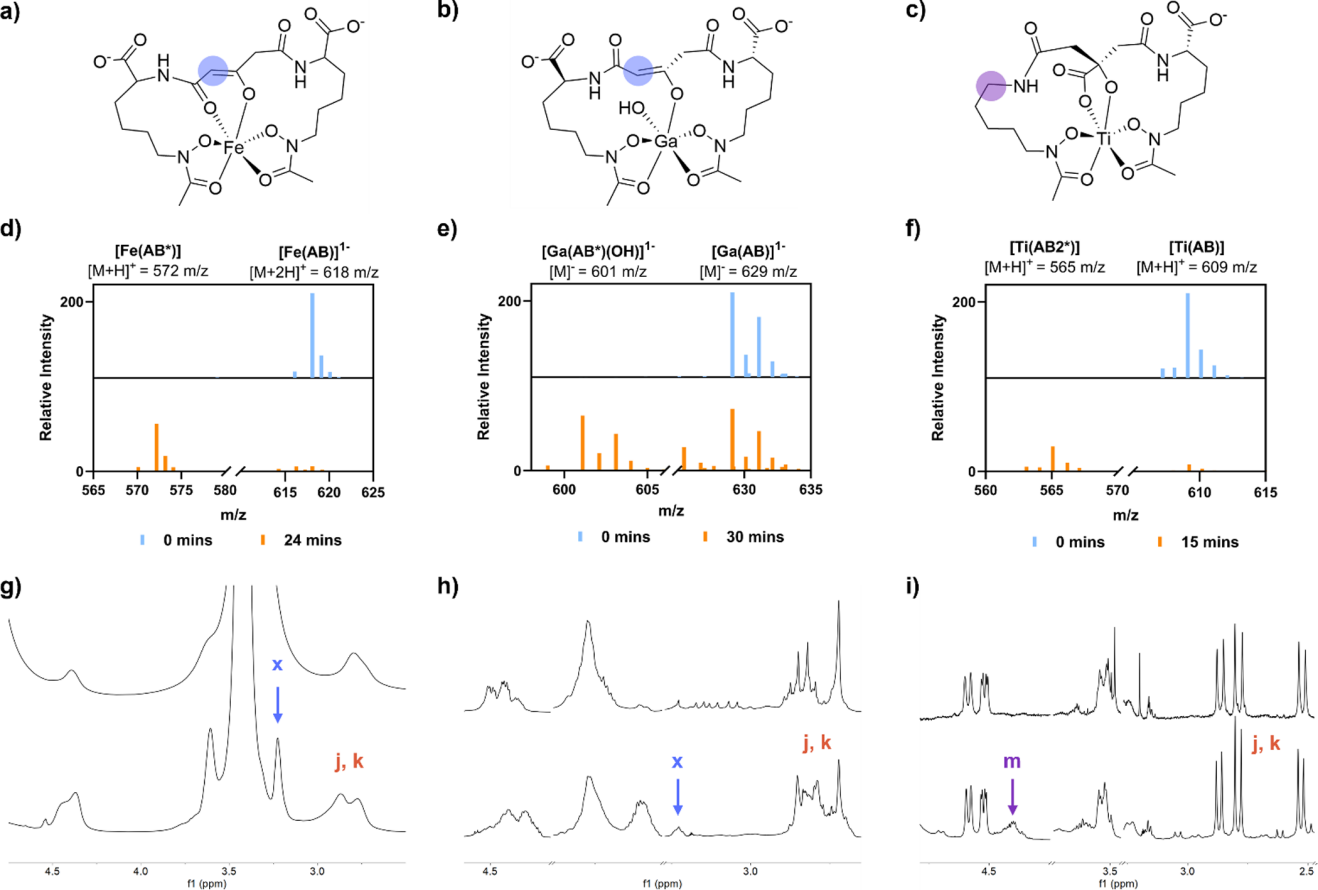
Wavelength-dependent photocleavage at 254 nm monitored by LC-MS under acidic conditions (d–f) and ^1^H NMR spectroscopy (g–i). (a) [Fe­(AB*)]^2–^, (b) [Ga­(AB*)]^2–^, (c) [Ti­(AB2*)]^2–^, (d) [Fe­(AB)]^1–^, (e) [Ga­(AB)]^1–^, (f) [Ti­(AB)], (g) [Fe­(AB)]^3–^, (h) [Ga­(AB)]^3–^, and (i) [Ti­(AB)]^2–^. Spectral annotations in panels (g–i) are previously defined in Figure 2.

**Figure 7:**
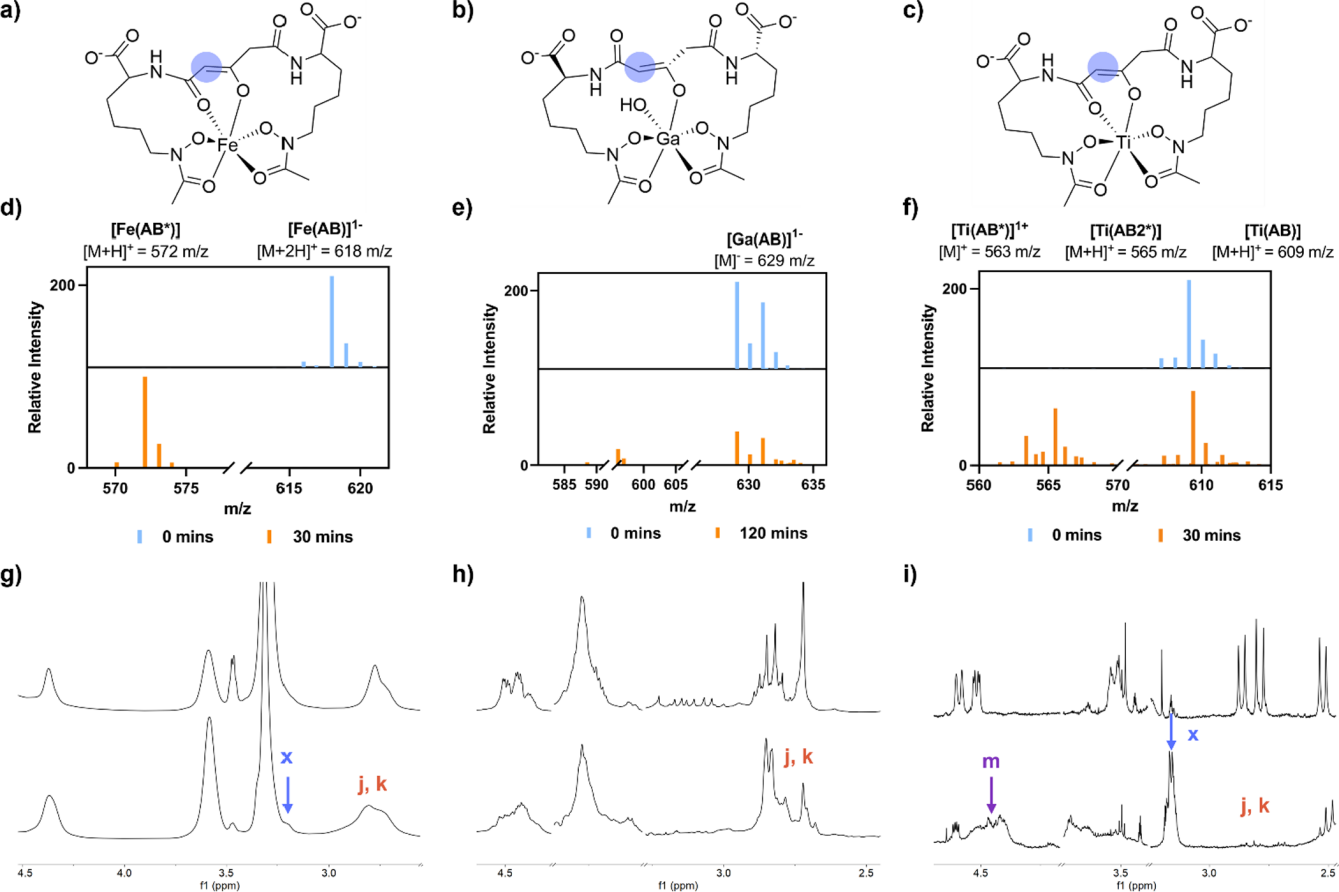
Photocleavage at 300 nm monitored by LC-MS under acidic conditions (d–f) and ^1^H NMR spectroscopy (g–i). (a) [Fe­(AB*)]^2–^, (b) [Ga­(AB*)]^2–^, (c) [Ti­(AB*)]^1–^, (d) [Fe­(AB)]^1–^, (e) [Ga­(AB)]^1–^, (f) [Ti­(AB)], (g) [Fe­(AB)]^3–^, (h) [Ga­(AB)]^3–^, and (i) [Ti­(AB)]^2–^. Spectral annotations in panels (g–i) are previously defined in Figure 2.

Analysis of the [Ti­(AB)]^2–^ photodegradation products did not reveal a *m*/*z* consistent with the anticipated [Ti­(AB*)]^1–^ (Figure 6f). Upon further analysis of the ^13^C NMR data following irradiation at 254 nm it became clear that a decarboxylation event was occurring (Figures S36 and S44), by loss of one of the lysine carboxylate groups, forming [Ti­(AB2*)]^1–^. The formation of [Ti­(AB2*)]^1–^ is consistent with the electron density difference map (EDDM) shown in Figure 4c for the transition at 225 nm, which indicates that this LMCT transition leads to a significant loss of electron density from the carboxylate that is responsible for decarboxylation. Figure 7f,i shows results obtained following lower energy irradiation at 300 nm, indicating the formation of the anticipated [Ti­(AB*)]^1–^. This observation is also consistent with our band assignments for [Ti­(AB)]^2–^ (Figure 4c), which suggest that irradiation at 295 nm should populate the hydroxamate → Ti^4+^ LMCT excited state that is electronically very similar in nature to the hydroxamate → Fe^3+^ LMCT excited state of the [Fe­(AB)]^3–^ complex (cf. Figure 4a,c). This result hints toward the wavelength dependent formation of the [Ti­(AB*)]^1–^ photoproduct that is homologous to the [Fe­(AB*)]^2–^.

### Proposed Mechanism of Photodegradation

Our experimental findings paired with the computational analysis enabled the elucidation of a mechanism of action that supports the formation of the aerobactin photoproduct. Scheme 2 pairs the potential radical species with the EDDMs for the initial LMCT transition and the subsequent electronic transition that occurs for the following step to procced. These EDDMs provided critical mechanistic insights into the photocleavage process of aerobactin. For both [Fe­(AB)]^3–^ and [Ti­(AB)]^2–^, the high-energy transitions primarily involve LMCT excitation originating from the hydroxamate groups (Scheme 2). This finding contradicts previous proposals, which suggested that charge transfer primarily occurs through the α-hydroxy carboxylate (Scheme 1).^4^ Alternatively, when [Ti­(AB)]^2–^ is irradiated at 254 nm and the secondary decarboxylation occurs, EDDMs depict that significant electron density is lost from the carboxylate undergoing decarboxylation forming [Ti­(AB2*)]^1–^ (Scheme 3). Additionally, EDDMs for the photoproduct complexes reveal that, following photoirradiation, primary electronic transitions shift to the α-ketoglutarate portion of aerobactin (Figures S56–S58), suggesting that this region plays a key role in stabilizing the radical species postcleavage and renders the dechelation of Fe^2+^ followed by rechelation of a secondary Fe^3+^ less likely.

**Scheme 2:**
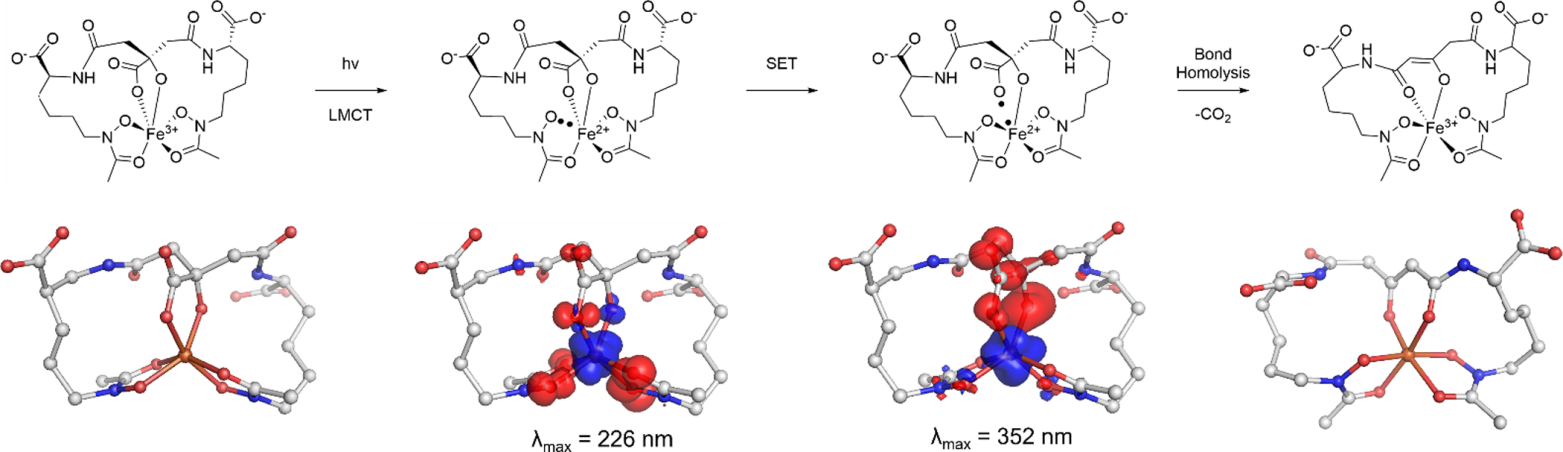
Top: Revised Mechanism of Action for Photochemical Cleavage of Citrate-Based Siderophores; Bottom: Computed EDDMs for the Relevant LMCT Transition and the Transition for the Single-Electron Transfer of [Fe­(AB)]^3–^ ^a^

a EDDMs depict electronic transitions with electron density gain shown in blue and electron density loss indicated in red.

**Scheme 3:**
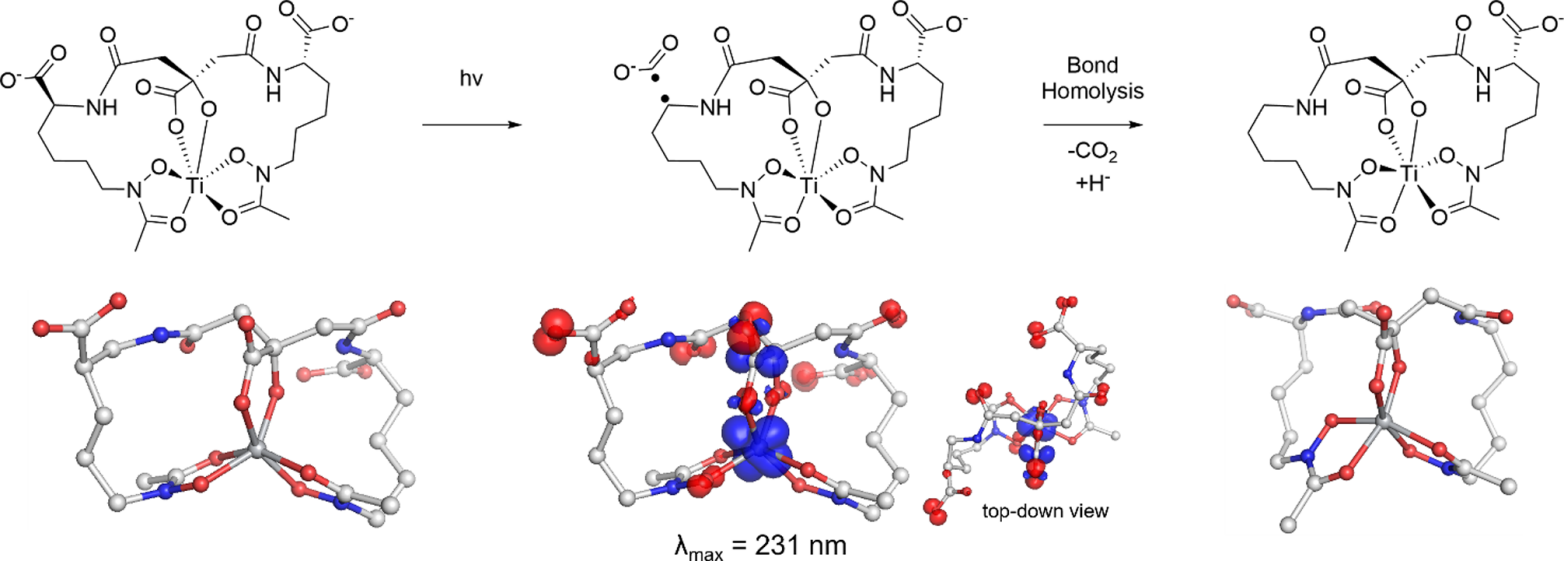
Top: Mechanism of Action for [Ti­(AB)]^2–^ at 254 nm Irradiation; Bottom: Computed EDDM for the Relevant Transition^a^

a EDDMs depict electronic transitions with electron density gain shown in blue and electron density loss indicated in red.

## Conclusions

This study expands our understanding of the photochemical properties of aerobactin, a biologically significant siderophore, by investigating its LMCT reactivity and photocleavage across both native (Fe^3^^+^) and non-native (Ga^3^^+^ and Ti^4+^) metal complexes. Spectroscopic studies monitoring reactants and products identified wavelength-dependent excitation pathways, revealing selective photocleavage behavior that enables controlled photoreactivity. DFT and TD-DFT calculations provided further insights into the electronic structures, geometric preferences, and radical stabilization mechanisms involved in aerobactin’s photochemical transformation. Specifically, we observe that aerobactin can be readily photodegraded at shorter wavelengths, producing a thus far unknown set of photodegradation products. Among the aerobactin bound metal ions investigated, we observe that Ti^4+^ can also produce the ketoenol photoproduct when irradiation is conducted at wavelengths that coincide with the complex’s characteristic LMCT band at 295 nm.

Our findings also challenge the conventional assumption that α-hydroxy carboxylates are the primary contributors to LMCT-driven cleavage, demonstrating that hydroxamate groups play a dominant role in initiating photoredox processes of this photoactive siderophore. Additionally, we show that non-native metal ions, particularly Ti^4^^+^, exhibit photochemical behavior homologous to Fe^3^^+^, suggesting that aerobactin’s reactivity extends beyond iron metabolism and may have broader applications in environmental and industrial settings in addition to advancing our understanding of siderophore-driven photochemistry.

## Experimental Section

### Materials and General Methods

All starting materials were purchased from Acros Organics, Alfa Aesar, Sigma-Aldrich, or TCI America and used without further purification. Aerobactin was purchased from BLD Pharma.

### Nuclear Magnetic Resonance (NMR) Spectroscopy

All spectra were recorded at the University of Wisconsin-Madison Department of Chemistry Paul Bender Chemical Instrumentation Center (CIC) using a Bruker Avance III 500 with a DCH liquid He cryoprobe (Bender Fund), a Bruker Avance Neo 500 with a 5 mm Prodigy-BBO liquid N_2_ cryoprobe (NSF CHE-2017891), a Bruker Avance-400 with a BBFO room temperature probe (UW Madison Instructional Laboratory Modernization Award) and a Bruker Avance III 600 with a TCIF liquid He cryoprobe (NIH S10 OD012245) and processed using MestReNova 14.3.3. Chemical shifts (δ) are reported in parts per million (ppm) relative to tetramethylsilane (TMS) at 0 ppm or CD_3_OD (^1^H: δ 3.31 ppm, ^13^C: δ 49.0 ppm). All spectra were acquired in CD_3_OD (^1^H: δ 3.31 ppm, ^13^C: δ 49.0 ppm). Data is presented as follows: chemical shift, multiplicity (s = singlet, d = doublet, t = triplet, q = quartet, and m = multiplet), integration, and coupling constant in hertz (Hz).

### Light Emitting Diode (LED) NMR Spectroscopy

All LED-NMR experiments utilized a LED with an emission centered at 365 nm (UHP-FB-365) from Prizmatix Ltd. (Holon, Israel). A glass optical fiber guides light into the NMR tube directly as an insert with the fiber optical tip sitting directly above the sample.

### Mass Spectrometry

Low-resolution electrospray ionization (ESI) mass spectrometry was carried out with a serial high performance liquid chromatography Agilent 1260 instrument Phenomenex, outfitted with a Luna 5 μm C18 column (150 mm × 3 mm, 100 Å, AXIA packed) at a flow rate of 0.8 mL/min with in- line single quadrupole mass spectrometer (Agilent G6125 Infinity LC/MSD) with positive and negative ion detection range of 100–2500 *m*/*z*. High-resolution ESI mass spectrometry was carried out at the University of Wisconsin-Madison Department of Chemistry Paul Bender Chemical Instrumentation Center (CIC) using a Thermo Scientific Q Exactive Focus Orbitrap MS system.

### UV–Vis Spectra

Spectra were collected with a NanoDrop 1C instrument (AZY1706045) and recorded from 190 to 850 nm in a quartz cuvette with 1 cm path length.

### Photochemical Cleavage Experiments

All photochemical cleavage experiments were conducted using the Rayonet Photochemical Reactor (RPR-200) from the Southern New England Ultraviolet Company. Wavelength dependent studies were recorded with 254, 300, 350, 420, and 575 nm lamps. All solutions were prepared in MeOH or MeOD-*d*_4_ at a concentration great enough to observe and monitor the lower energy transitions at approximately 0.5 mM. UV–vis experiments were prepared in a quartz cuvette with a 1 cm path length. All UV–vis experiments were conducted in triplicate to ensure that the cleavage was proceeding at the same relative rates. Alternatively, experiments conducted by NMR, external to the spectrometer, were prepared in quartz NMR tubes and irradiated at 254 nm prior to acquiring spectra.

### Aerobactin Complexes

Commercial aerobactin was prepared as a 50 mM solution in MeOH. Metal ion stocks of Ti­(iPrO)_4_*, FeCl_3_, and Ga­(NO_3_)_3_ were prepared as 50 mM solutions in MeOH as well. Stoichiometric equivalent amounts of metal ion stock and aerobactin stock solutions were added together and diluted to a relevant concentration. The solutions were then allowed to equilibrate under ambient conditions for at least 1 h. Consequent metal ion complexes were then characterized by ^1^H NMR, ^13^C­{^1^H} NMR, HRMS, and UV–vis.

* indicates that in solution, some TiO_2_ was observed to form and precipitate out. A PTFE syringe filter with 0.2 μm pores was utilized to ensure that the precipitate was removed prior to conducting photochemical and characterization experiments.

### Computational Parameters

Calculations were performed using the version 5.0.1 of the Orca5 software package.^40,41^ Computations were performed with the CAM-B3LYP functional^42^ with the D3­(BJ) dispersion^43^ and the def2-TZVP^44^ basis set. Calculations were performed with the CPCM polarizable continuum model solvation model^45–47^ with methanol as the solvent. Geometry optimized structures as well as electron density difference maps (EDDMs) were modeled in PyMOL^48^ with all hydrogens omitted for clarity. All atom coloring is as follows: carbons are depicted as gray, oxygens as red, and nitrogens as blue. All EDDMs are depicted with electropositive orbitals in blue and electronegative orbitals in red with an isosurface value of 0.01.

## Supplementary Material



## Notes

Experimental procedures, synthetic description and characterization details, DFT structures, and tabulated data (PDF)
