# Supplementary material for: Photodecarboxylation of the Siderophore Aerobactin with the Lewis Acidic Metal Ions Fe(III), Ga(III), and Ti(IV)
Source: Inorg Chem. 2025 Oct 1;64(40):20013–22. doi: 10.1021/acs.inorgchem.5c02471 (PMC12522143; doi:10.1021/acs.inorgchem.5c02471)
Supplement: Supplementary file 1 [file ic5c02471_si_001.pdf]

Supplementary Information for: "Photodecarboxylation of the Siderophore Aerobactin with the Lewis Acidic Metal Ions Fe(III), Ga(III) and Ti(IV)"

Edith K. Amason, Thomas C. Brunold, and Eszter Boros*

Department of Chemistry, University of Wisconsin-Madison, Madison, Wisconsin 53706, United States * Email: eboros@wisc.edu

Experimental Characterization 2
Isomer Naming 10
LC-MS Monitored Photocleavage 11
NMR Characterization 16
Variable Temperature NMR Experiments 38
Time Dependent Photoirradiation Monitored by NMR 41
Spectral Comparison Data Tables 48
UV-Vis Data 52
Density Functional Theory Calculations 58
Coordinates for Geometry Optimized Compounds 72
References 132

1. **Experimental Characterization**

Aerobactin. (AB) was obtained from BLD Pharma and used without further purification. The starting material was characterized using NMR, mass spectrometry, and UV-vis. ^{1}H NMR (500 MHz, MeOD) δ 4.39 (dt, *J* = 8.8, 5.1 Hz, 2H), 3.60 (dq, *J* = 18.6, 7.0 Hz, 4H), 2.78 (s, 2H), 2.77 (dd, *J* = 41.0, 14.5 Hz, 2H), 2.09 (s, 6H), 1.87 (dtd, *J* = 18.6, 9.8, 5.2 Hz, 2H), 1.62 (tt, *J* = 13.7, 6.9 Hz, 2H), 1.40 (dd, *J* = 11.3, 4.8 Hz, 4H). ^{13}C NMR (126 MHz, MeOD) δ 175.25, 174.05, 172.22, 171.03, 170.48, 73.79, 51.98, 43.50, 42.83, 30.85, 25.77, 22.35, 18.84.

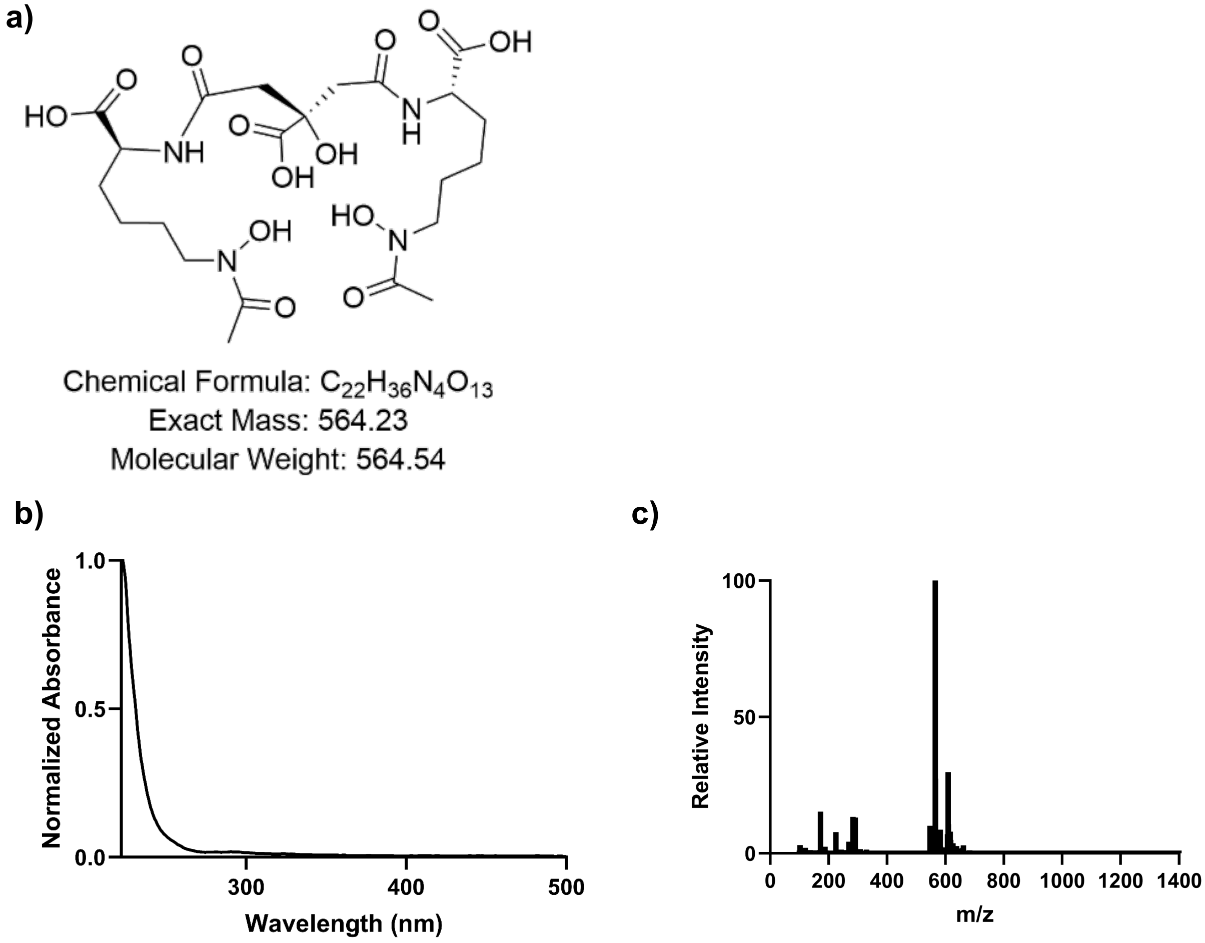

Figure S1. Compound structure **(a)**, absorbance spectrum **(b)**, and mass spectrum **(c)**.

Iron(III) Aerobactin. [Fe(AB)]$^{3-}$ was synthesized as mentioned previously used without further purification. The material was characterized using NMR, mass spectrometry, and UV-vis. ^{1}H NMR (600 MHz, MeOD) δ 4.57 – 4.23 (m, 1H), 3.66 – 3.61 (m, 7H), 2.90 – 2.67 (m, 3H), 2.12 (s, 4H), 1.96 – 1.32 (m, 12H). ^{13}C NMR (151 MHz, MeOD) δ 175.42, 174.07, 171.98, 75.75, 57.51, 55.64, 53.54, 32.06, 27.12, 23.69.

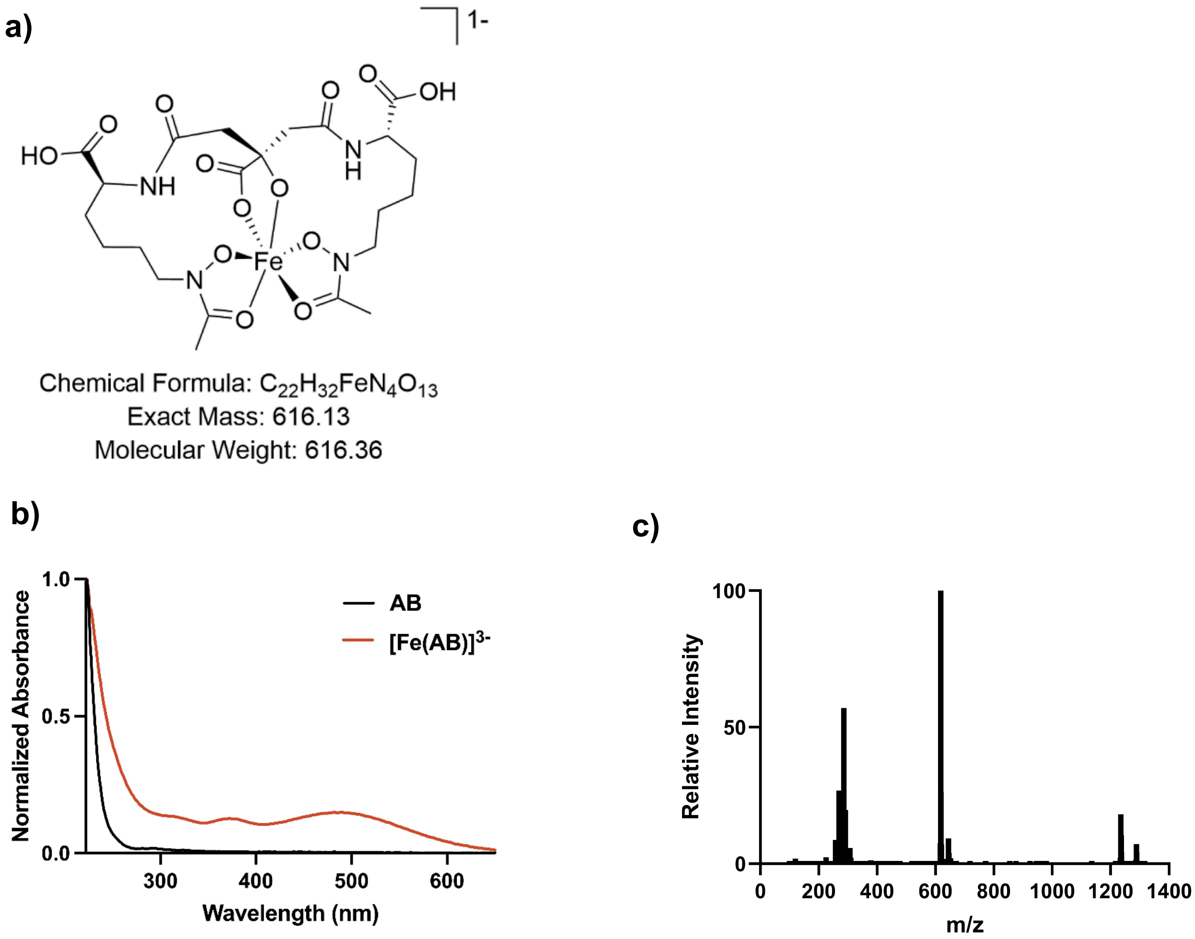

Figure S2. Complex structure **(a)**, absorbance spectra overlay **(b)**, and mass spectrum **(c)**.

Gallium(III) Aerobactin. [Ga(AB)]$^{3-}$ was synthesized as mentioned previously used without further purification. The material was characterized using NMR, mass spectrometry, and UV-vis. ^{1}H NMR (600 MHz, MeOD) δ 4.54 – 4.48 (m, 1H), 4.48 – 4.44 (m, 1H), 4.44 – 4.39 (m, 1H), 3.77 (qd, J = 14.1, 6.0 Hz, 7H), 2.83 (dt, J = 33.3, 15.0 Hz, 3H), 2.72 (s, 2H), 2.23 (s, 7H), 1.93 – 1.37 (m, 23H). ^{13}C NMR (151 MHz, MeOD) δ 175.67, 174.14, 173.38, 172.23, 163.74, 75.35, 55.34, 54.99, 53.41, 52.25, 45.03, 43.84, 32.35, 27.54, 23.59, 17.08.

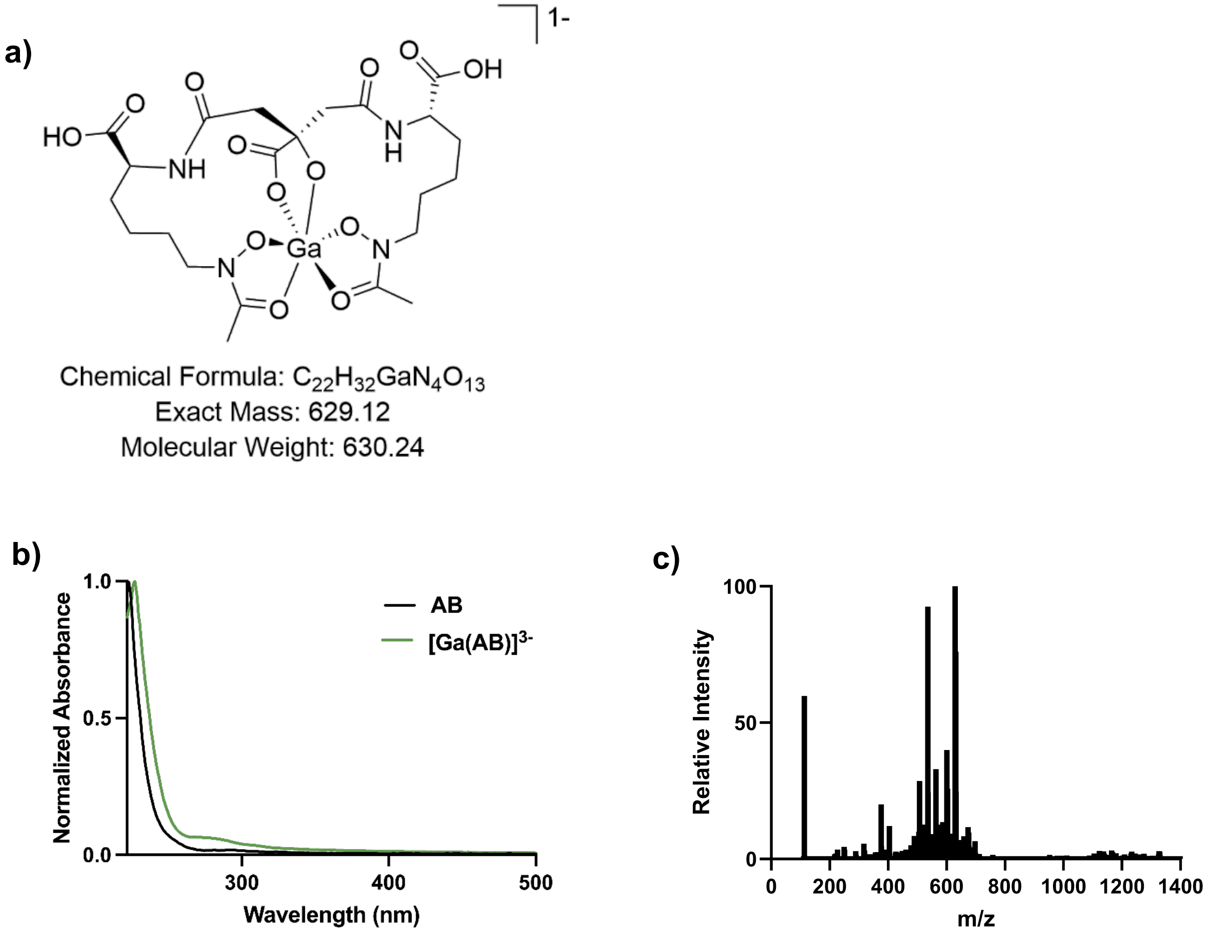

Figure S3. Complex structure **(a)**, absorbance spectra overlay **(b)**, and mass spectrum **(c)**.

Titanium(IV) Aerobactin. [Ti(AB)]$^{2-}$ was synthesized as mentioned previously used without further purification. The material was characterized using NMR, mass spectrometry, and UV-vis. ^{1}H NMR (500 MHz, MeOD) δ 4.59 (d, *J* = 11.8 Hz, 1H), 4.52 (dd, *J* = 9.8, 3.6 Hz, 1H), 3.53 (d, *J* = 13.5 Hz, 4H), 2.79 (d, *J* = 15.4 Hz, 1H), 2.69 (dd, *J* = 170.6, 13.6 Hz, 2H), 2.26 (s, 4H), 2.21 (s, 3H), 2.05 – 1.44 (m, 12H). ^{13}C NMR (126 MHz, MeOD) δ 186.22, 175.76, 175.16, 171.28, 168.87, 167.71, 96.16, 52.47, 51.72, 51.32, 46.76, 46.08, 32.17, 31.36, 27.29, 26.01, 24.18, 21.54, 16.58.

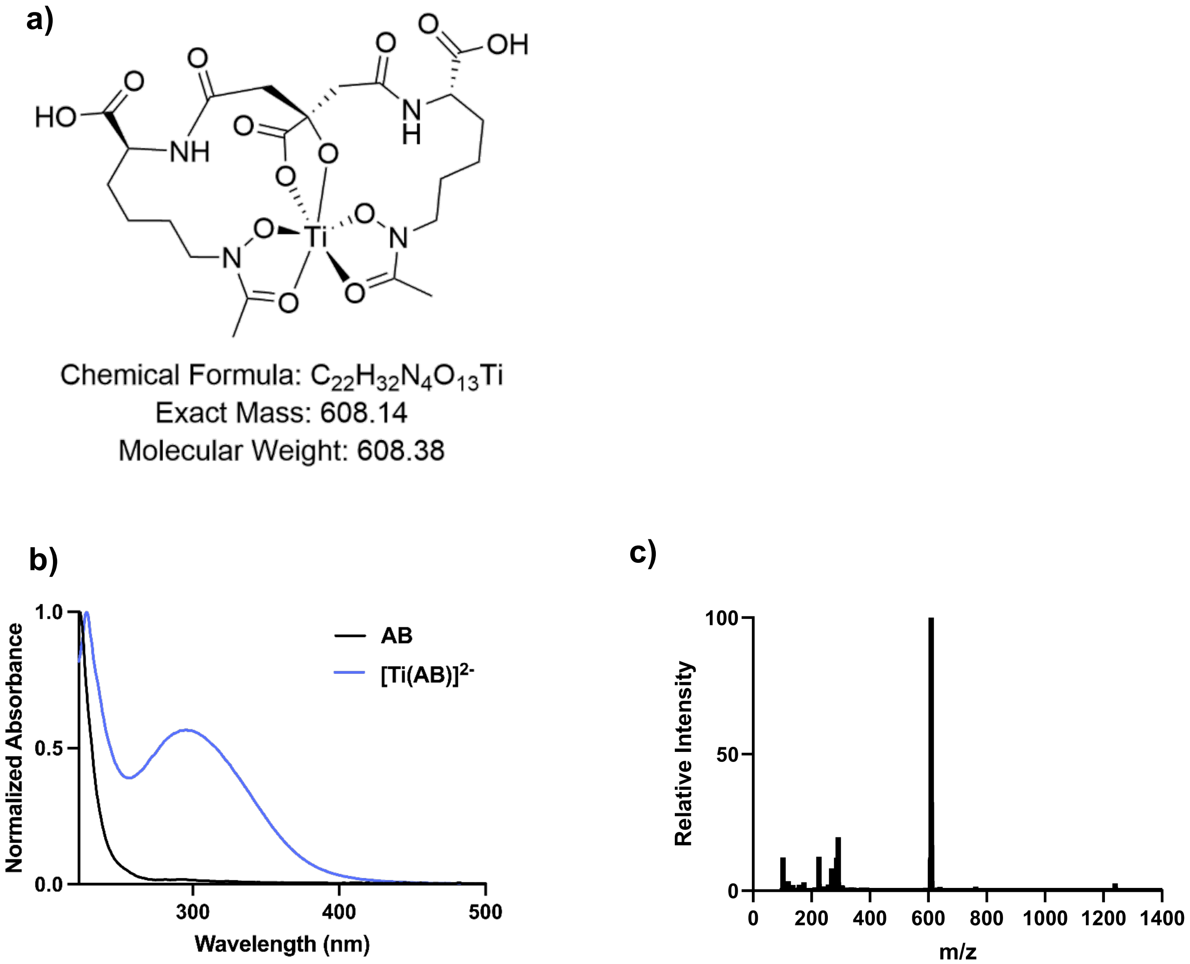

Figure S4. Complex structure **(a)**, absorbance spectra overlay **(b)**, and mass spectrum **(c)**.

Aerobactin*. (AB*) was obtained after photoirradiation at 254 nm and used without further purification. The material was characterized using NMR, mass spectrometry, and UV-vis. ^{1}H NMR (600 MHz, MeOD) δ 4.37 (s, 1H), 3.60 (dq, J = 13.8, 6.9 Hz, 2H), 3.18 – 3.14 (m, 0H), 2.78 (s, 1H), 2.77 (dd, J = 47.1, 14.8 Hz, 2H), 2.15 (s, 1H), 2.09 (s, 3H), 1.99 (s, 0H), 1.93 (s, 0H), 1.91 – 1.69 (m, 3H), 1.69 – 1.57 (m, 2H), 1.40 (p, J = 8.3 Hz, 2H). ^{13}C NMR (151 MHz, MeOD) δ 210.13, 176.66, 173.67, 172.47, 171.92, 101.20, 75.67, 53.37, 48.55, 44.33, 32.00, 23.77.

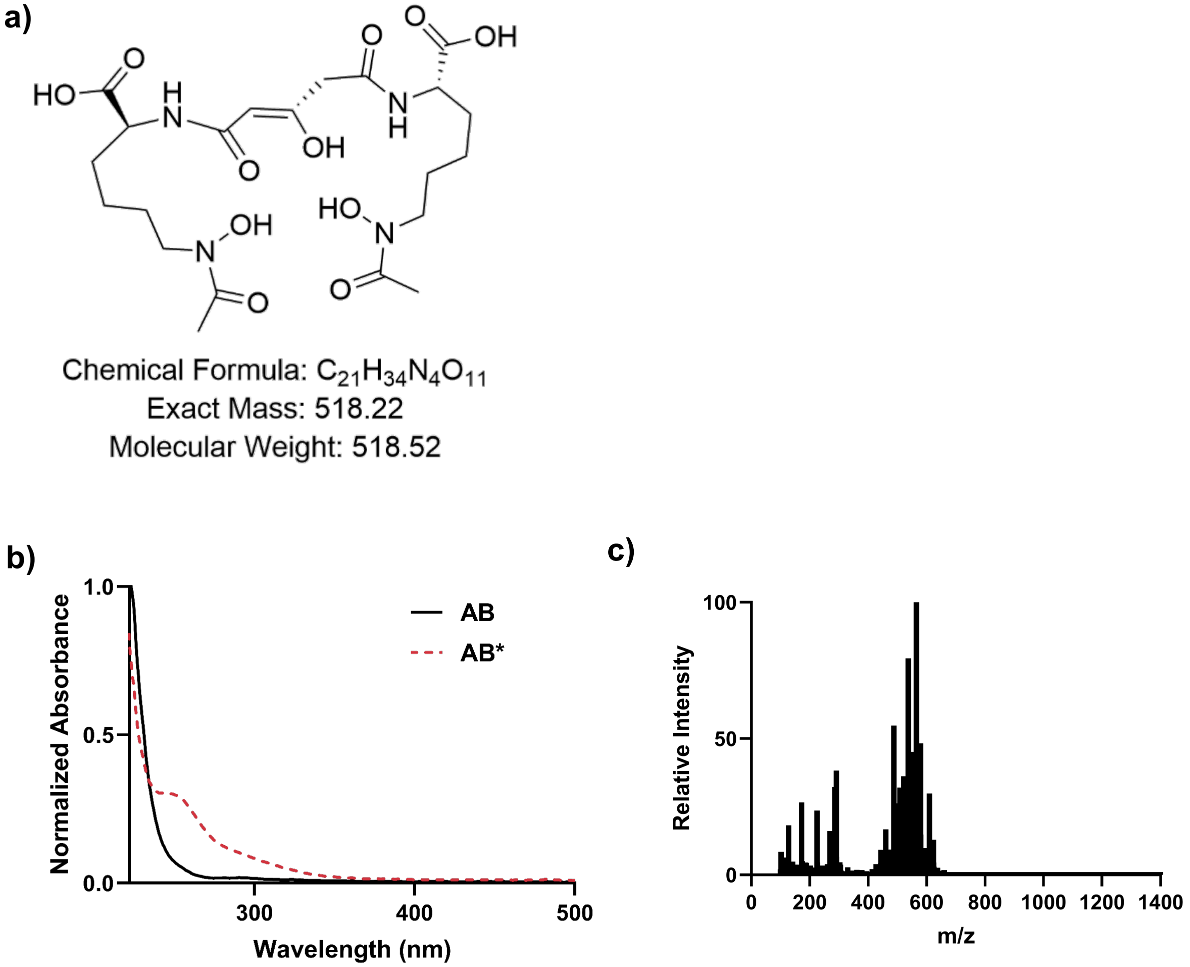

Figure S5. Compound structure **(a)**, absorbance spectra overlay **(b)**, and mass spectrum **(c)**.

Iron(III) Aerobactin*. [Fe(AB*)]$^{2-}$ was obtained through the photoirradiation of [Fe(AB)]$^{3-}$ at 254 nm and used without further purification. The material was characterized using NMR, mass spectrometry, and UV-vis. ^{1}H NMR (600 MHz, MeOD) δ 4.41 – 4.35 (m, 1H), 3.63 – 3.58 (m, 2H), 3.27 – 3.17 (m, 1H), 2.86 – 2.72 (m, 2H), 2.10 (s, 3H), 2.02 (s, 1H), 1.85 – 1.37 (m, 11H). ^{13}C NMR (151 MHz, MeOD) δ 172.66, 145.73, 80.51, 74.55, 51.98, 51.53, 51.29, 50.55, 43.93, 30.55, 25.63, 22.21, 18.84.

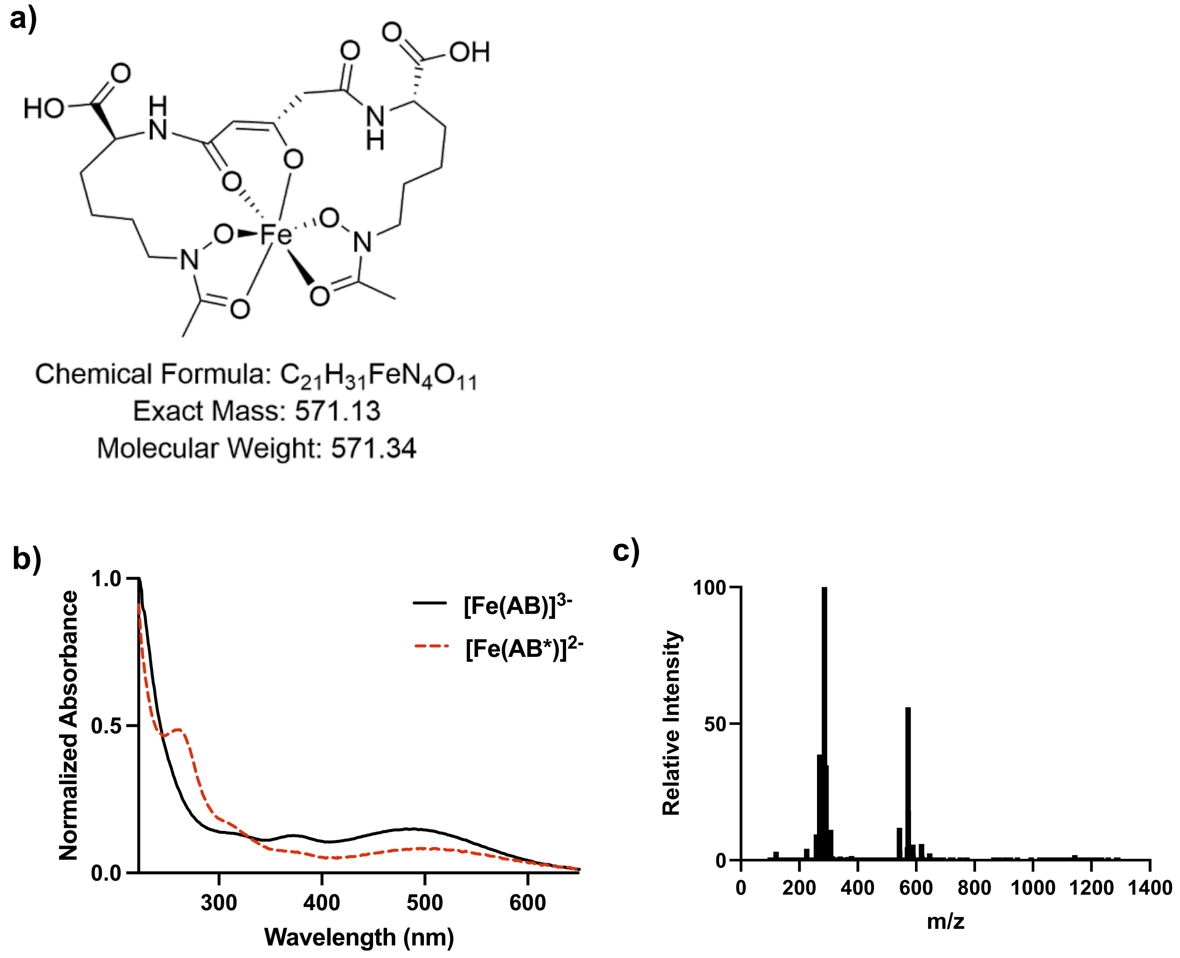

Figure S6. Complex structure **(a)**, absorbance spectra overlay **(b)**, and mass spectrum **(c)**.

Gallium(III) Aerobactin*. [Ga(AB*)]$^{2-}$ was obtained through the photoirradiation of [Ga(AB)]$^{3-}$ at 254 nm and used without further purification. The material was characterized using NMR, mass spectrometry, and UV-vis. ^{1}H NMR (600 MHz, MeOD) δ 4.44 (s, 1H), 4.38 (d, J = 10.7 Hz, 1H), 3.80 – 3.74 (m, 1H), 3.60 (dq, J = 13.0, 6.7 Hz, 1H), 3.23 (s, 0H), 2.88 – 2.67 (m, 4H), 2.23 (s, 4H), 2.15 (s, 1H), 2.13 (s, 1H), 2.10 (s, 2H), 2.02 (s, 0H), 1.92 – 1.35 (m, 13H). ^{13}C NMR (151 MHz, MeOD) δ 210.28, 176.72, 175.44, 173.68, 172.06, 163.76, 101.24, 75.38, 53.48, 48.54, 44.32, 32.21, 27.14, 23.77, 16.96.

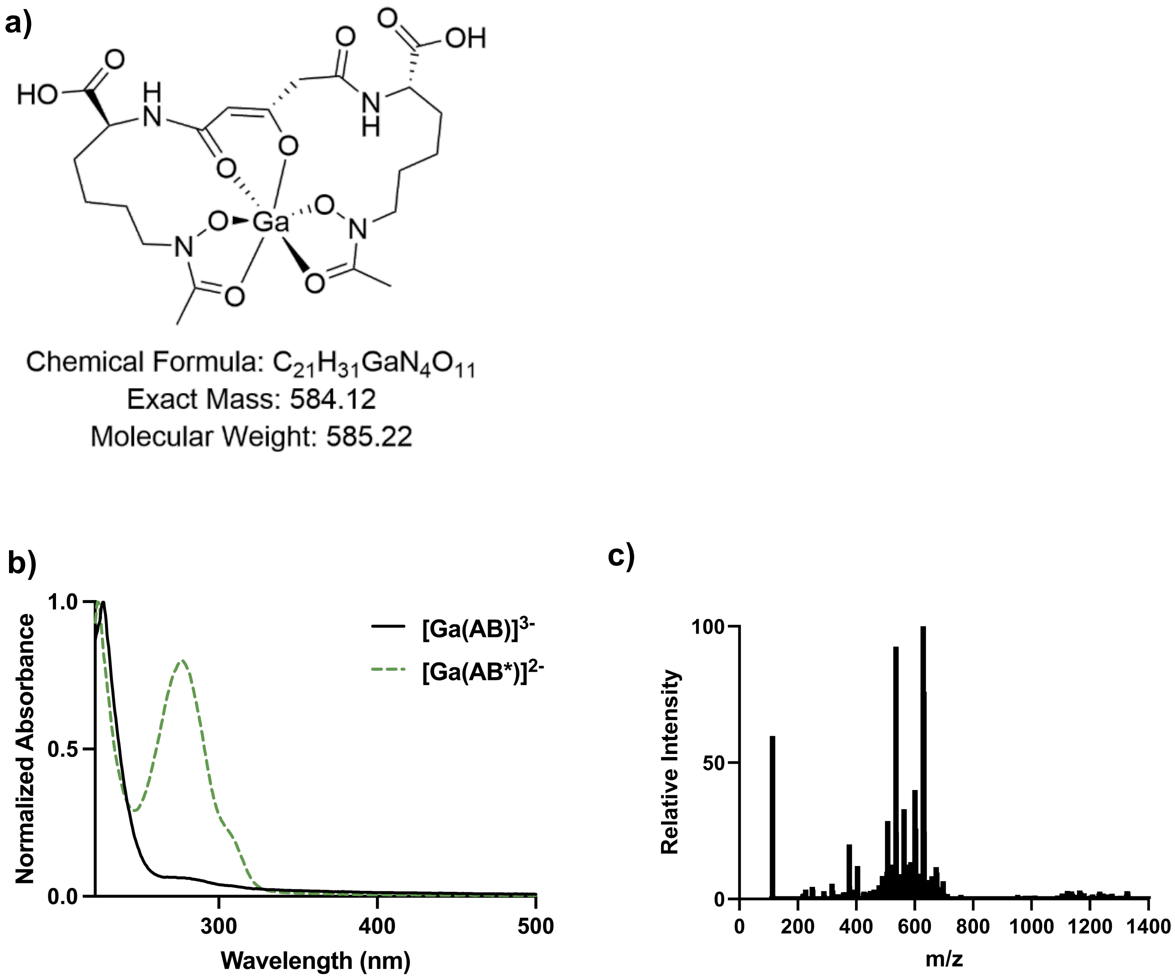

Figure S7. Complex structure **(a)**, absorbance spectra overlay **(b)**, and mass spectrum **(c)**.

Titanium(IV) Aerobactin 2*. [Ti(AB2)*] was obtained through the photoirradiation of [Ti(AB)] at 254 nm and used without further purification. The material was characterized using NMR, mass spectrometry, and UV-vis. ^{1}H NMR (600 MHz, MeOD) δ 4.59 (d, J = 11.9 Hz, 1H), 4.52 (dd, J = 9.8, 3.6 Hz, 1H), 4.45 – 4.35 (m, 1H), 3.53 (d, J = 15.2 Hz, 2H), 2.79 (d, J = 15.4 Hz, 1H), 2.70 (dd, J = 204.1, 13.6 Hz, 3H), 2.26 (s, 3H), 2.21 (s, 3H), 2.09 (s, 1H), 1.99 (s, 1H), 1.81 – 1.39 (m, 14H). ^{13}C NMR (151 MHz, MeOD) δ 186.21, 178.49, 177.57, 175.57, 173.67, 171.30, 168.87, 167.70, 165.63, 159.25, 157.94, 96.24, 46.86, 46.17, 32.11, 29.75, 16.62.

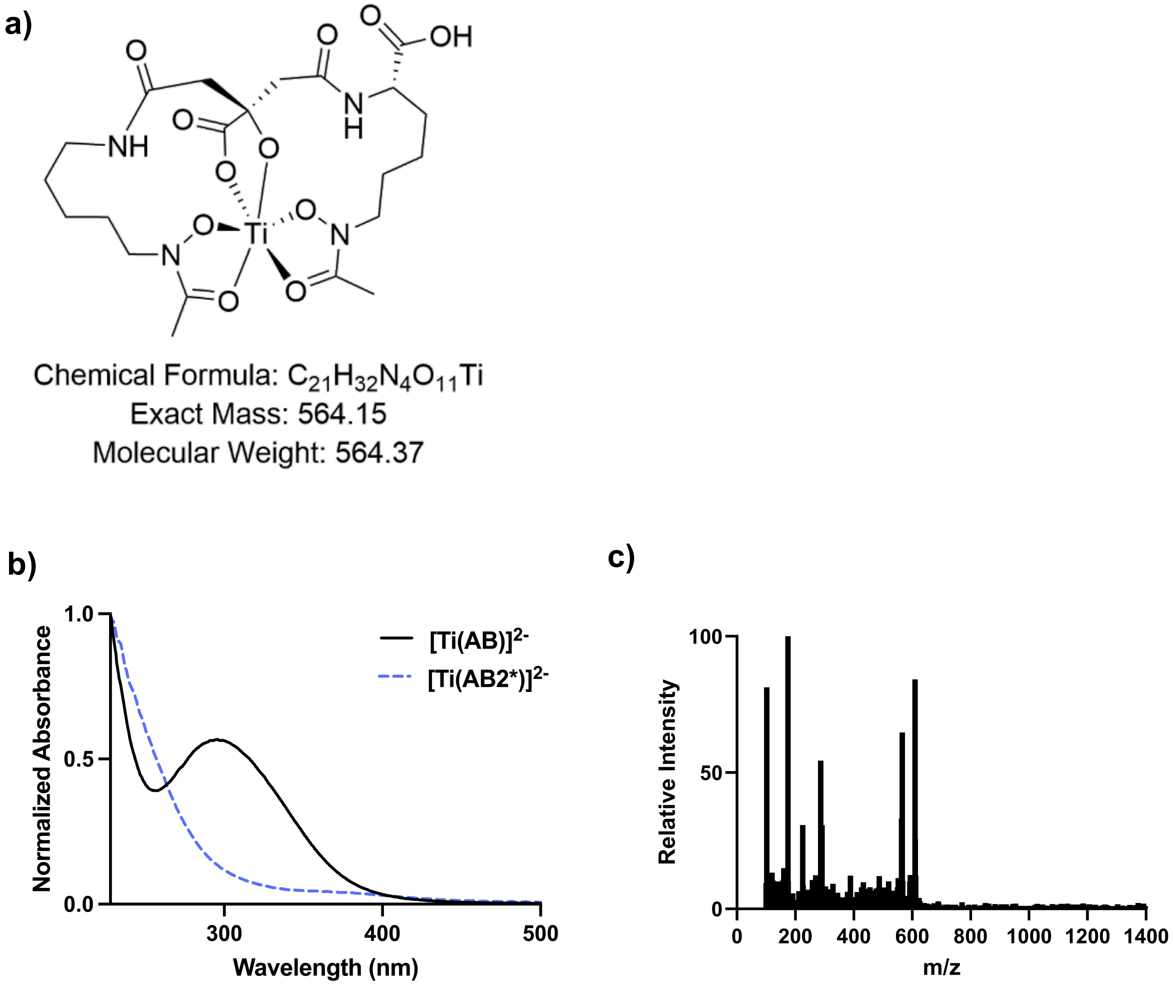

Figure S8. Complex structure **(a)**, absorbance spectra overlay **(b)**, and mass spectrum **(c)**.

Isomer Naming

Scheme S1. Naming of aerobactin isomers and aerobactin photoproduct isomers.

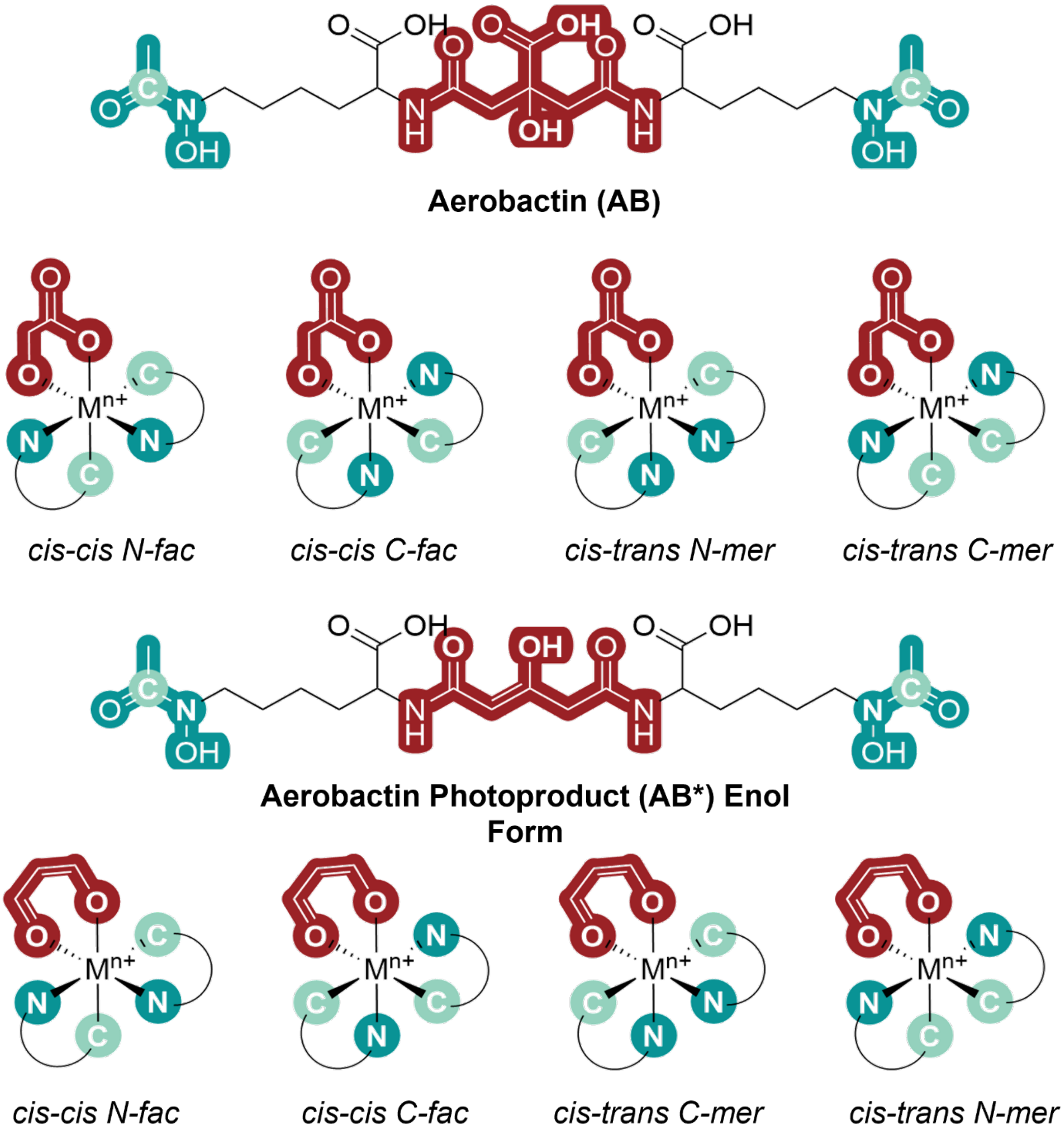

LC-MS Monitored Photocleavage

Scheme S2. Structures for identified species following 254 nm and 300 nm irradiation of aerobactin.

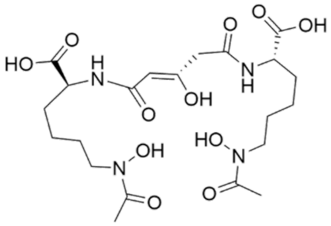

Chemical Formula: $C_{21}H_{34}N_4O_{11}$
Exact Mass: 518.22
Molecular Weight: 518.52

AB*

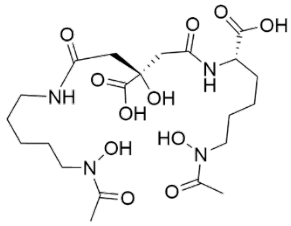

Chemical Formula: $C_{21}H_{36}N_4O_{11}$
Exact Mass: 520.24
Molecular Weight: 520.54

AB2*

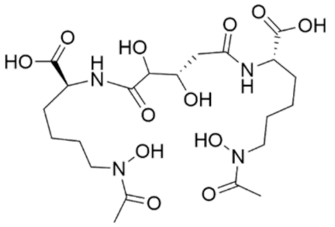

Chemical Formula: $C_{21}H_{36}N_4O_{12}$
Exact Mass: 536.23
Molecular Weight: 536.54

AB3*

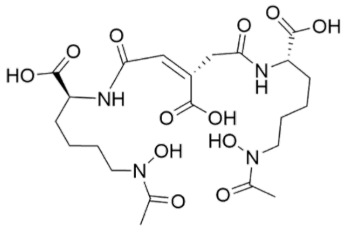

Chemical Formula: $C_{22}H_{34}N_4O_{12}$
Exact Mass: 546.22
Molecular Weight: 546.53

AB4*

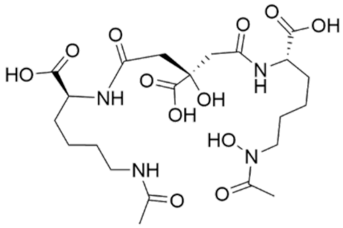

Chemical Formula: $C_{22}H_{36}N_4O_{12}$
Exact Mass: 548.23
Molecular Weight: 548.55

AB5*

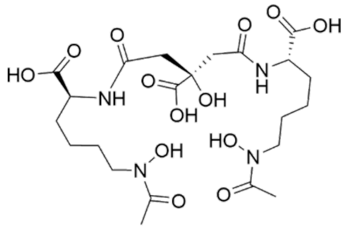

Chemical Formula: $C_{22}H_{36}N_4O_{13}$
Exact Mass: 564.23
Molecular Weight: 564.54

AB

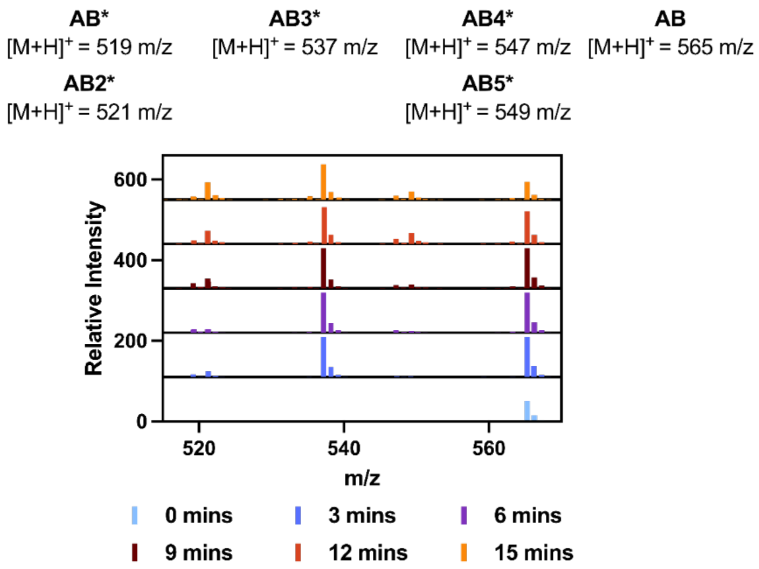

Figure S9. Mass-spectrometry monitored photocleavage of aerobactin at 254 nm.

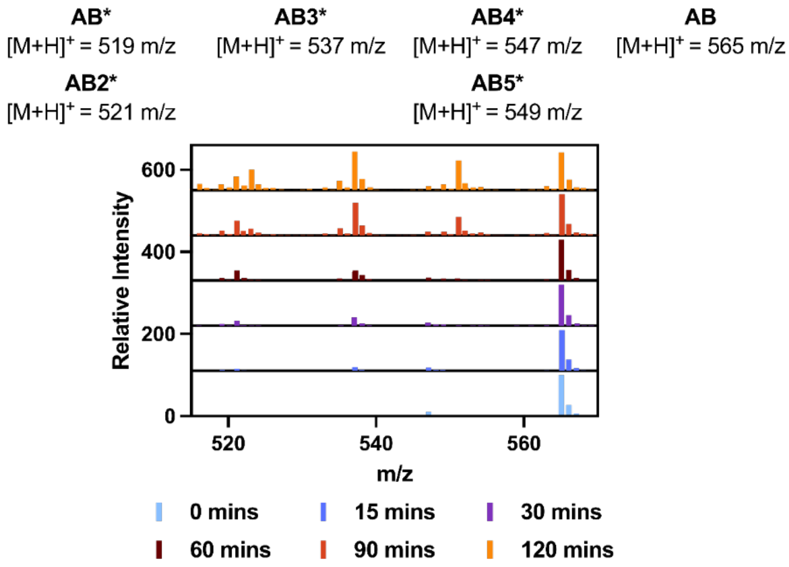

Figure S10. Mass-spectrometry monitored photocleavage of aerobactin at 300 nm.

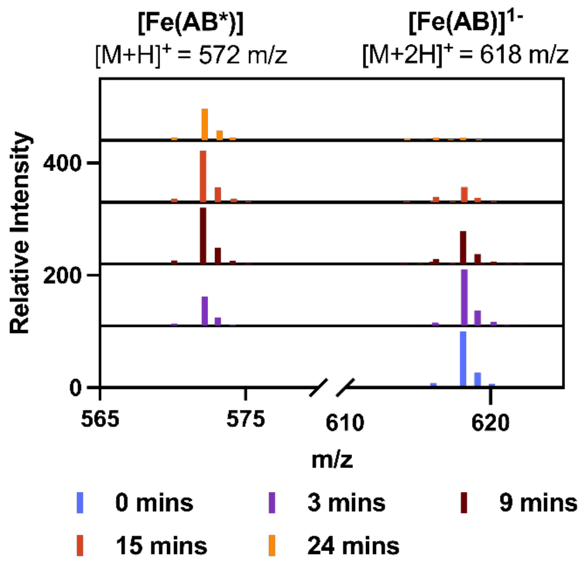

Figure S11. Mass-spectrometry monitored photocleavage of **[Fe(AB)]**$^{1-}$ at 254 nm.

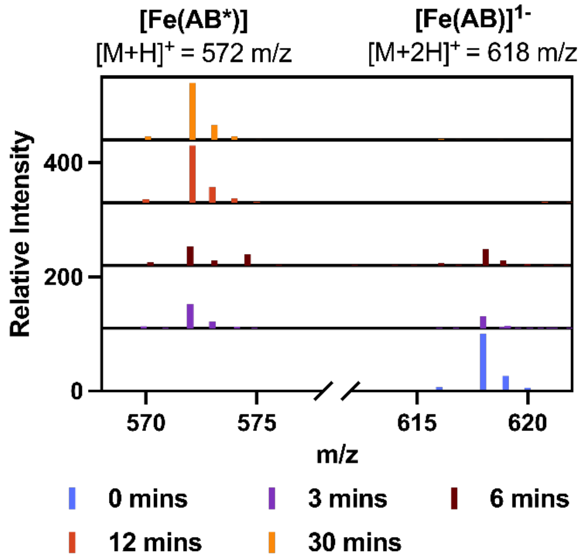

Figure S12. Mass-spectrometry monitored photocleavage of **[Fe(AB)]**$^{1-}$ at 300 nm.

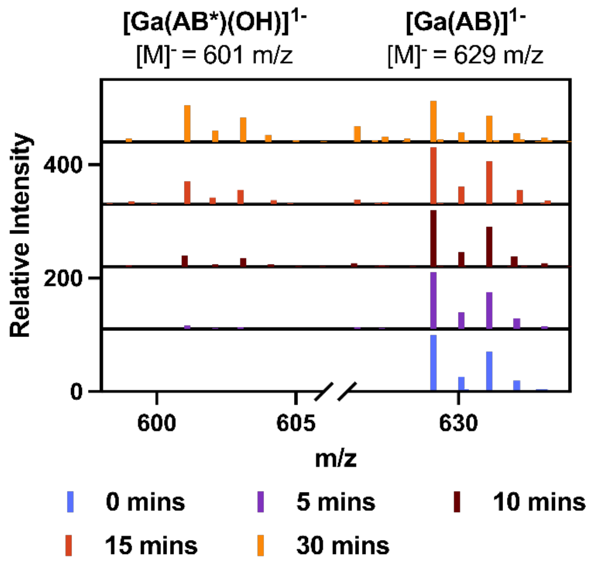

Figure S13. Mass-spectrometry monitored photocleavage of **[Ga(AB)]$^{1-}$** at 254 nm.

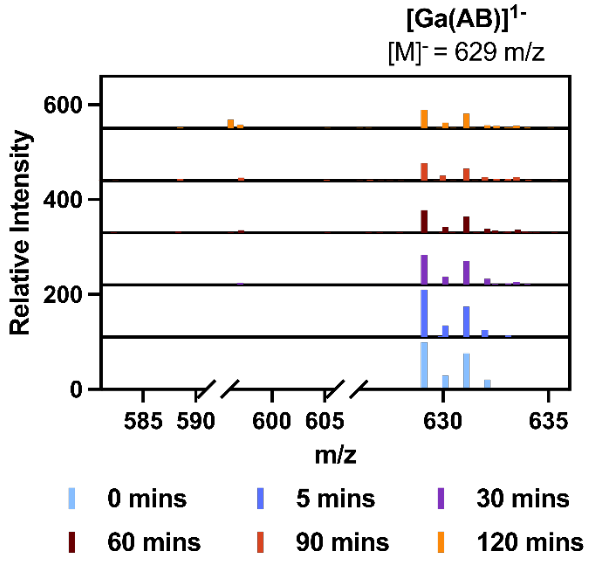

Figure S14. Mass-spectrometry monitored photocleavage of **[Ga(AB)]$^{1-}$** at 300 nm.

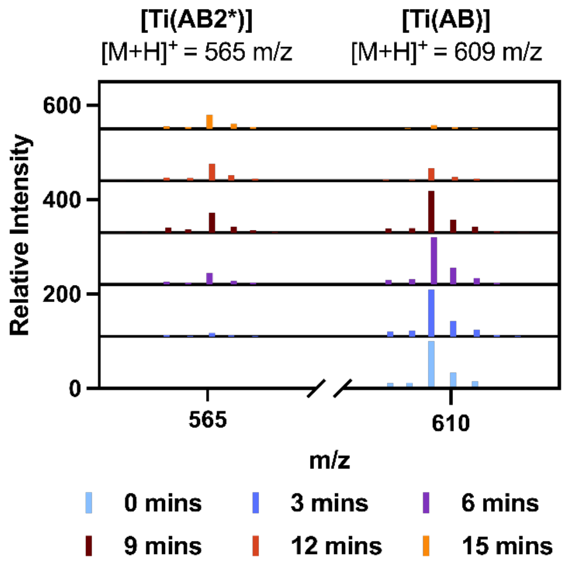

Figure S15. Mass-spectrometry monitored photocleavage of **[Ti(AB)]** at 254 nm.

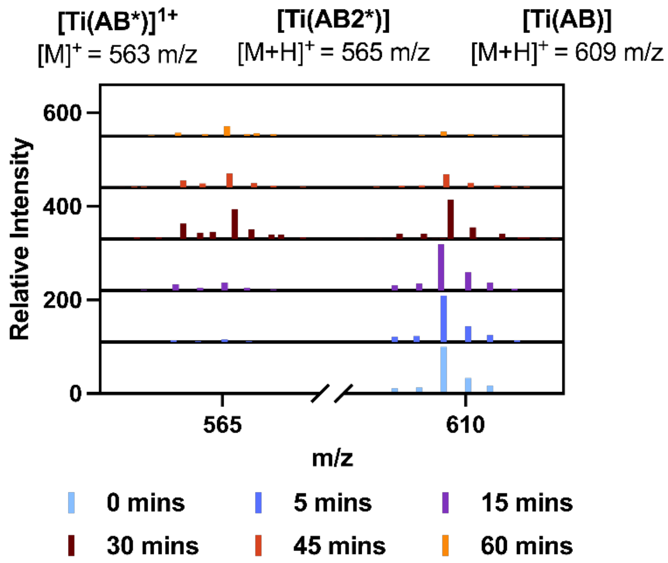

Figure S16. Mass-spectrometry monitored photocleavage of **[Ti(AB)]** at 300 nm.

NMR Characterization

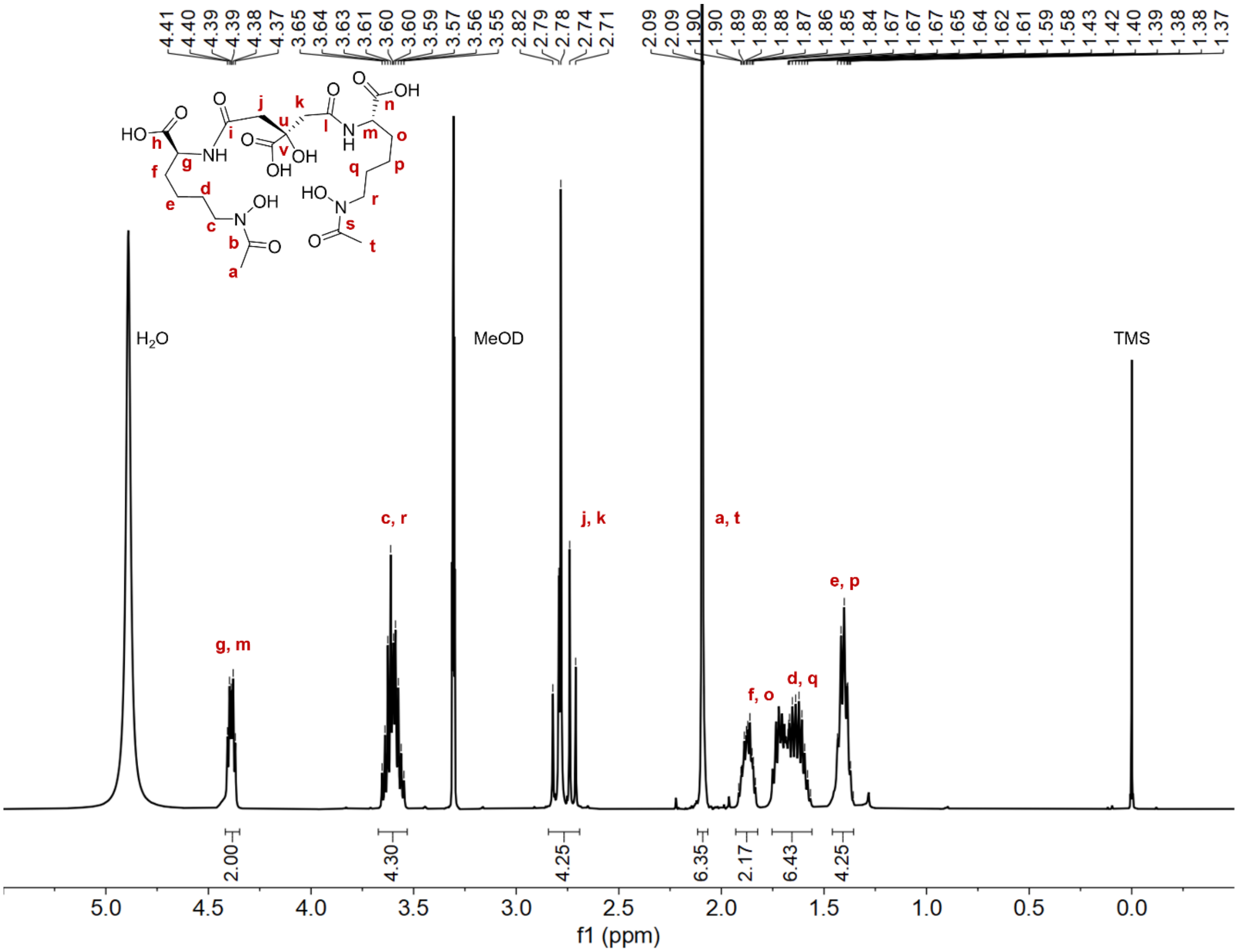

Figure S17. ^{1}H NMR (500 MHz) of **AB** in MeOD-d4.

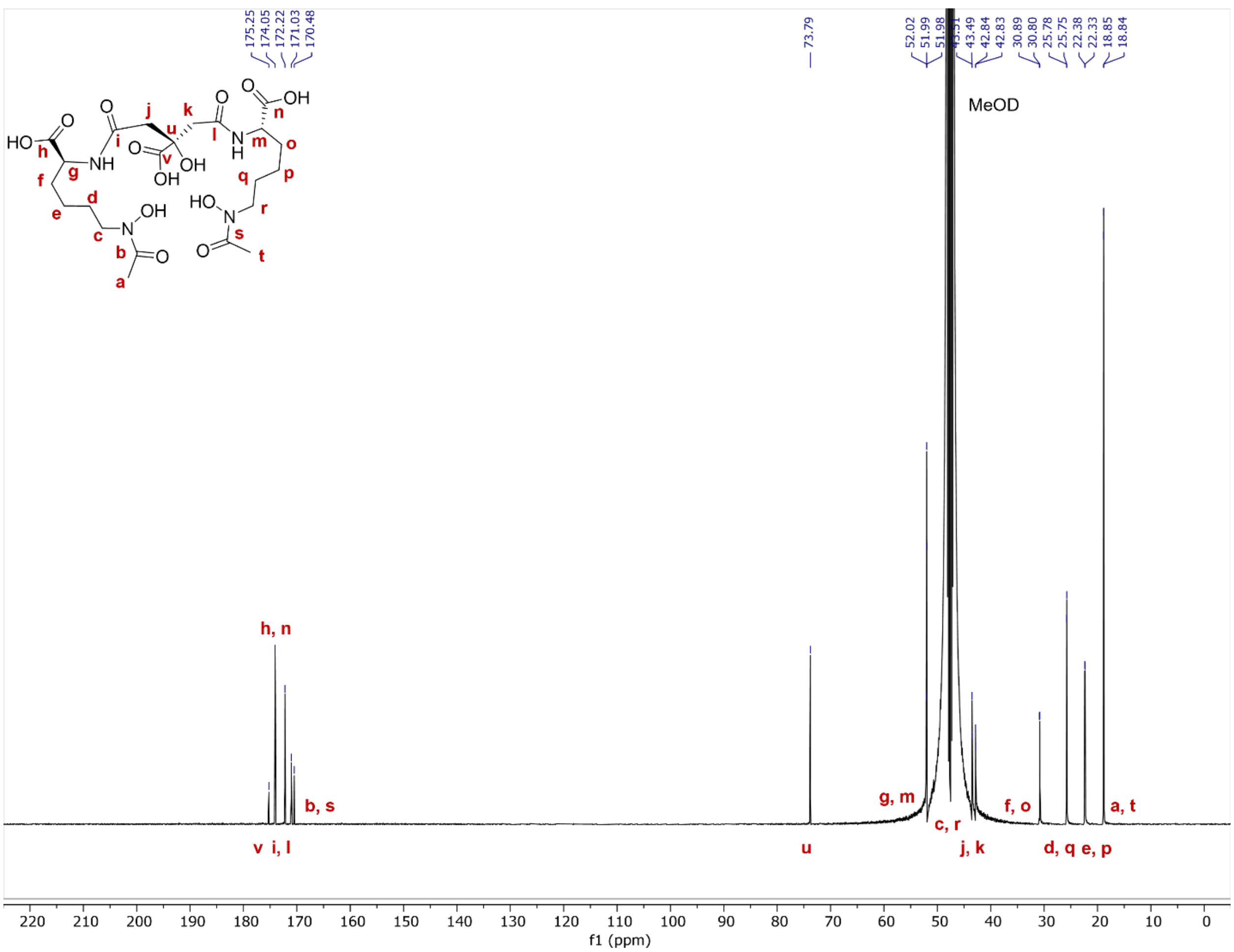

Figure S18. ^{13}C NMR (126 MHz) of **AB** in MeOD-d$_4$.

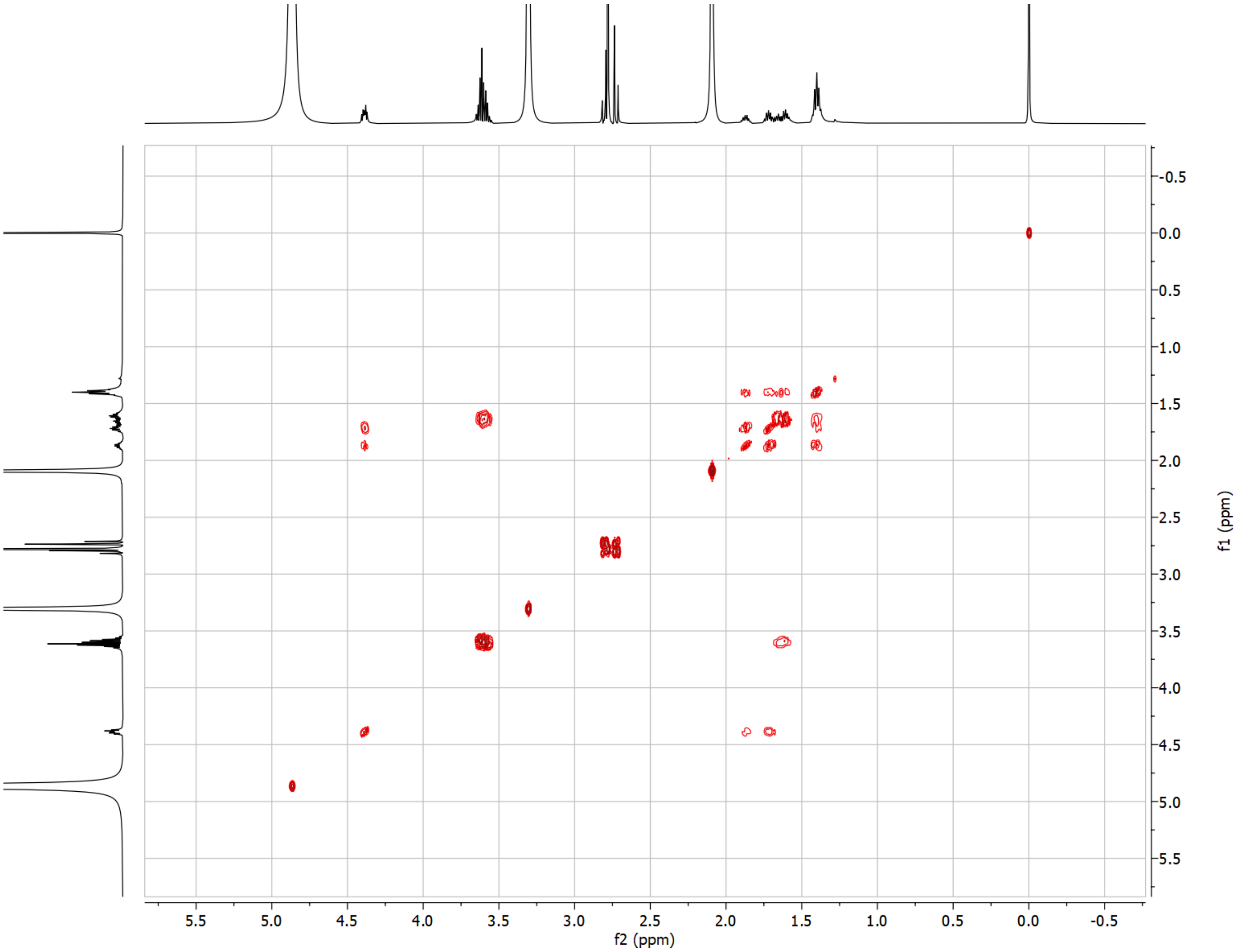

Figure S19. HH-COSY NMR (500 MHz) of **AB** in MeOD-d_4.

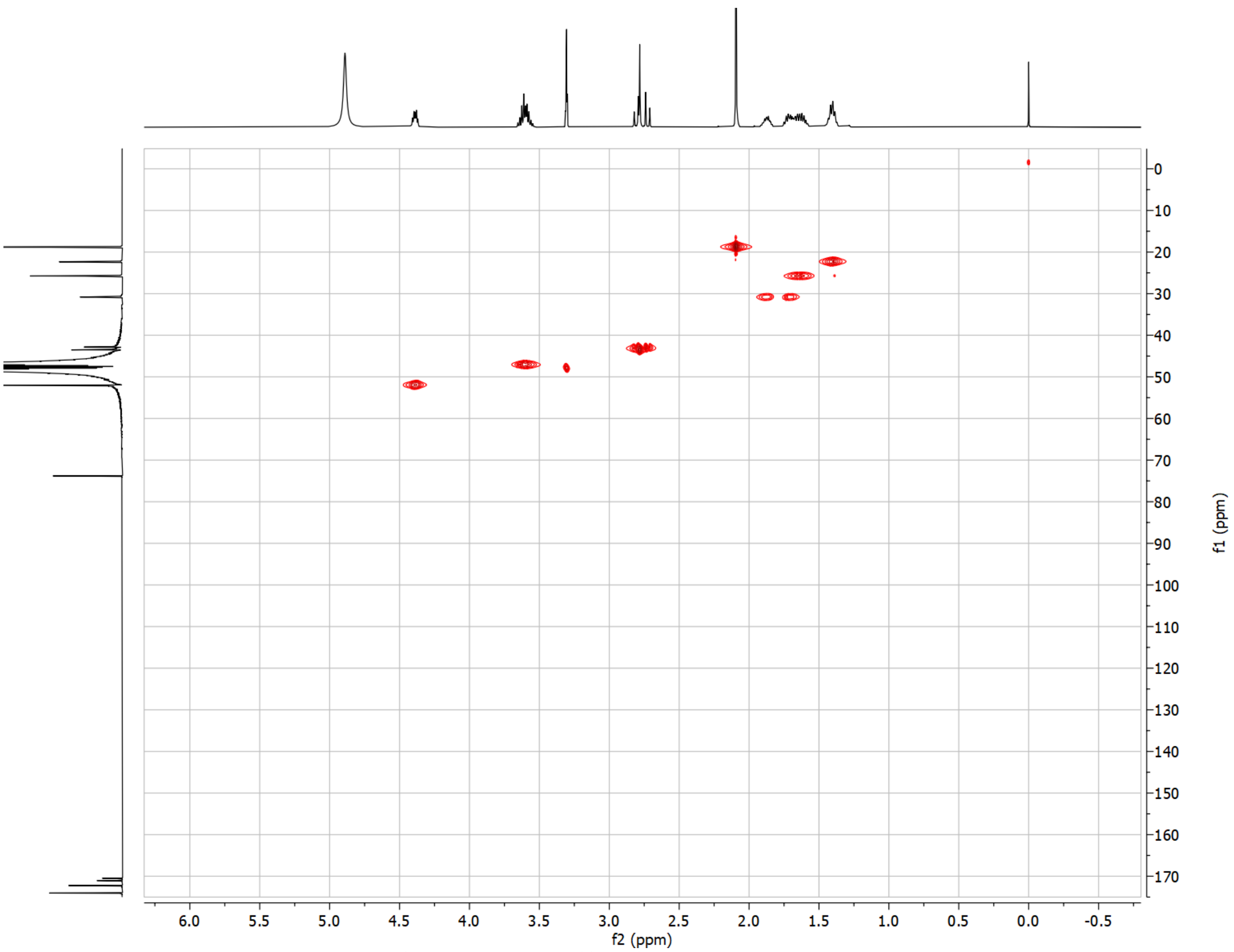

Figure S20. HC-HSQC NMR (^{1}H 500 MHz, $^{13}C\{^{1}H\}$ 126 MHz) of **AB** in MeOD-d_4.

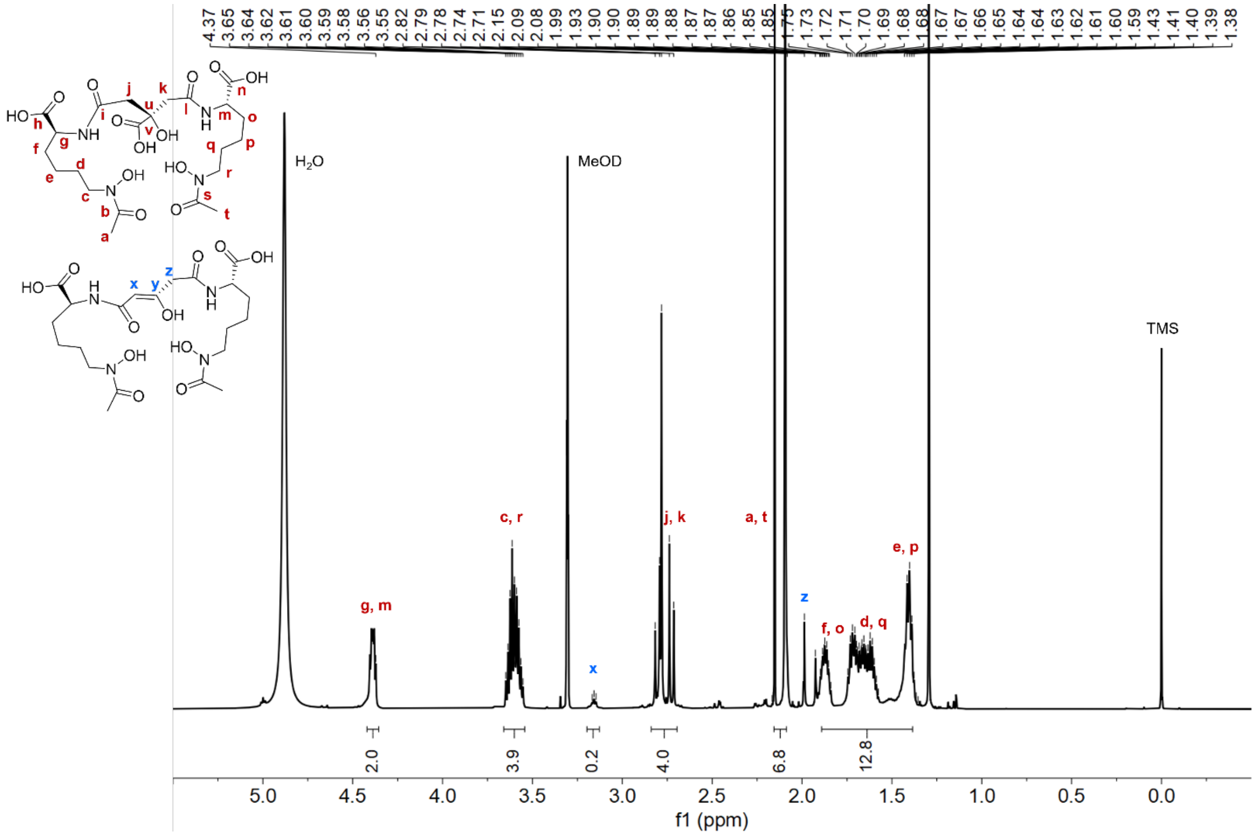

Figure S21. ^{1}H NMR (600 MHz) of **AB*** in MeOD-d$_4$.

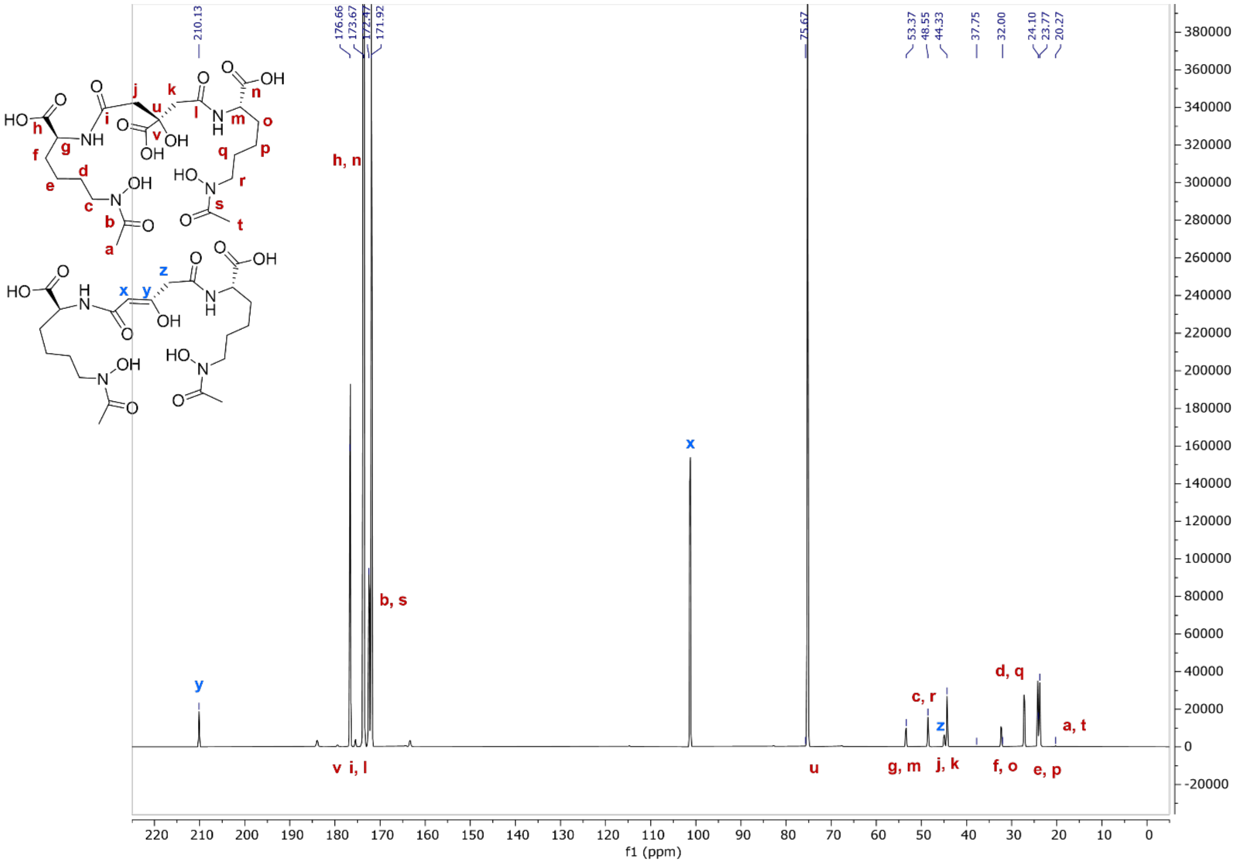

Figure S22. $^{13}C\{^1H\}$ NMR (151 MHz) of **AB*** in MeOD-d$_4$.

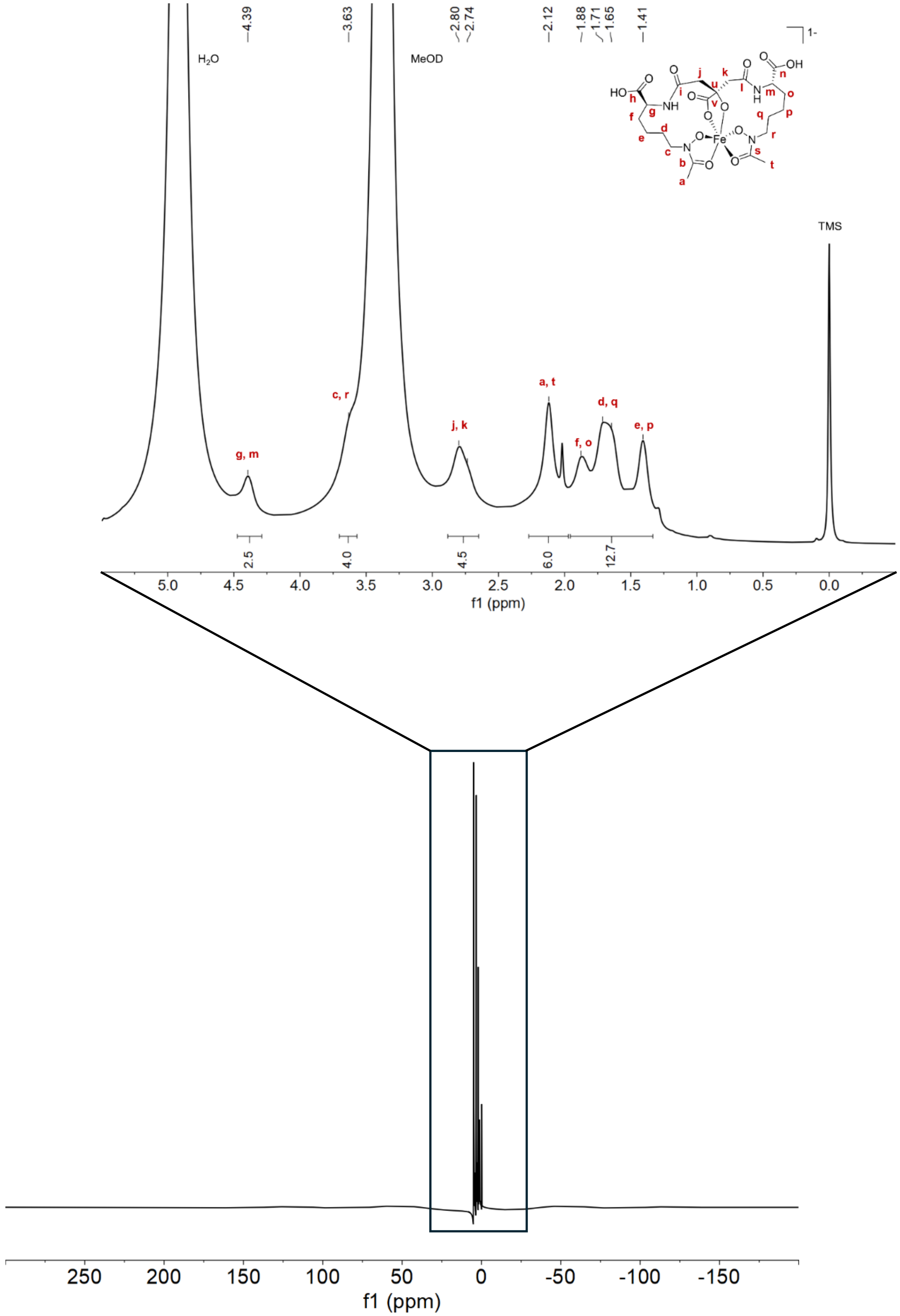

Figure S23. ^{1}H NMR (600 MHz) of **$[Fe(AB)]^{3-}$** in MeOD-d_4.

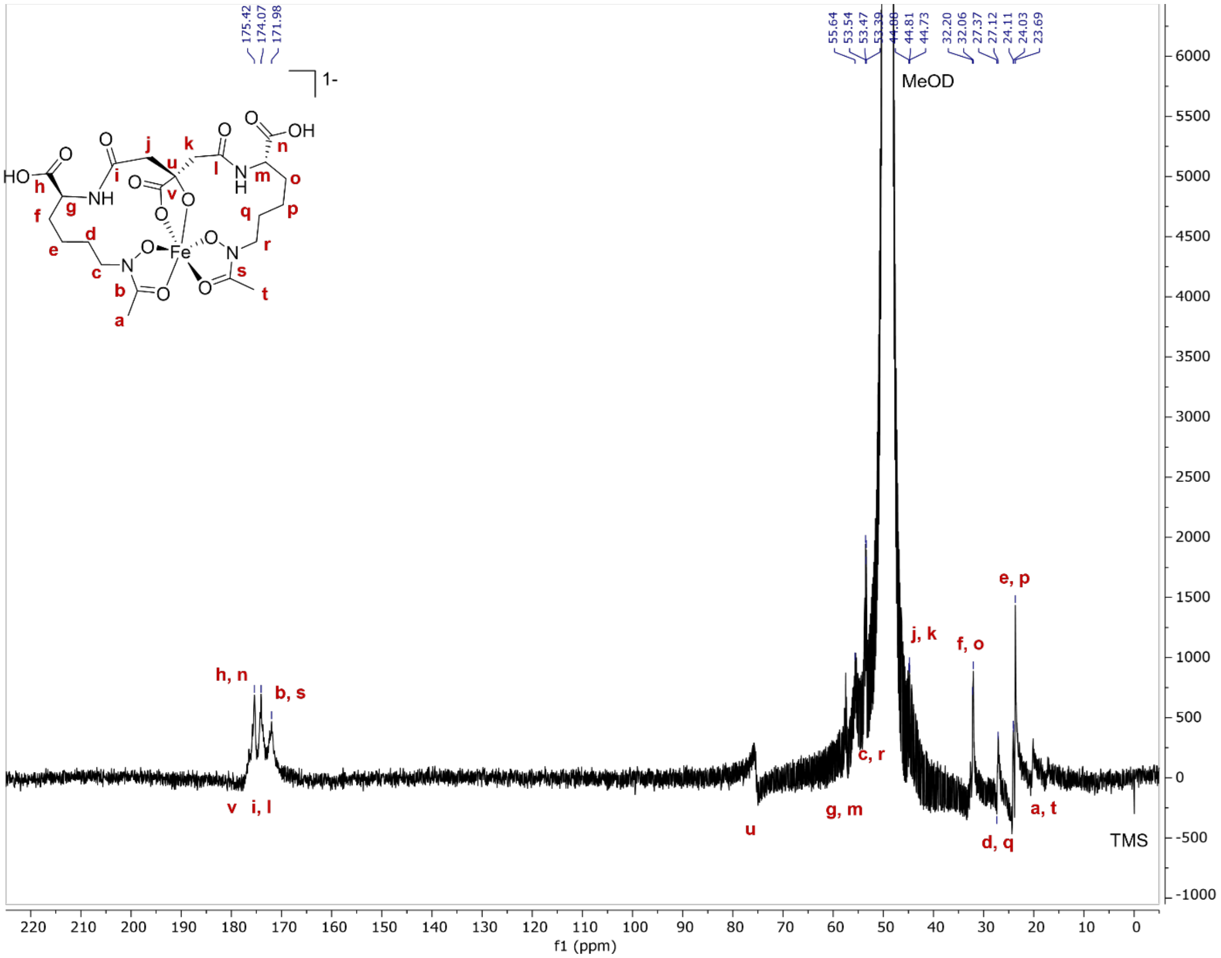

Figure S24. $^{13}C\{^{1}H\}$ NMR (151 MHz) of **$[Fe(AB)]^{3-}$** in MeOD-d4.

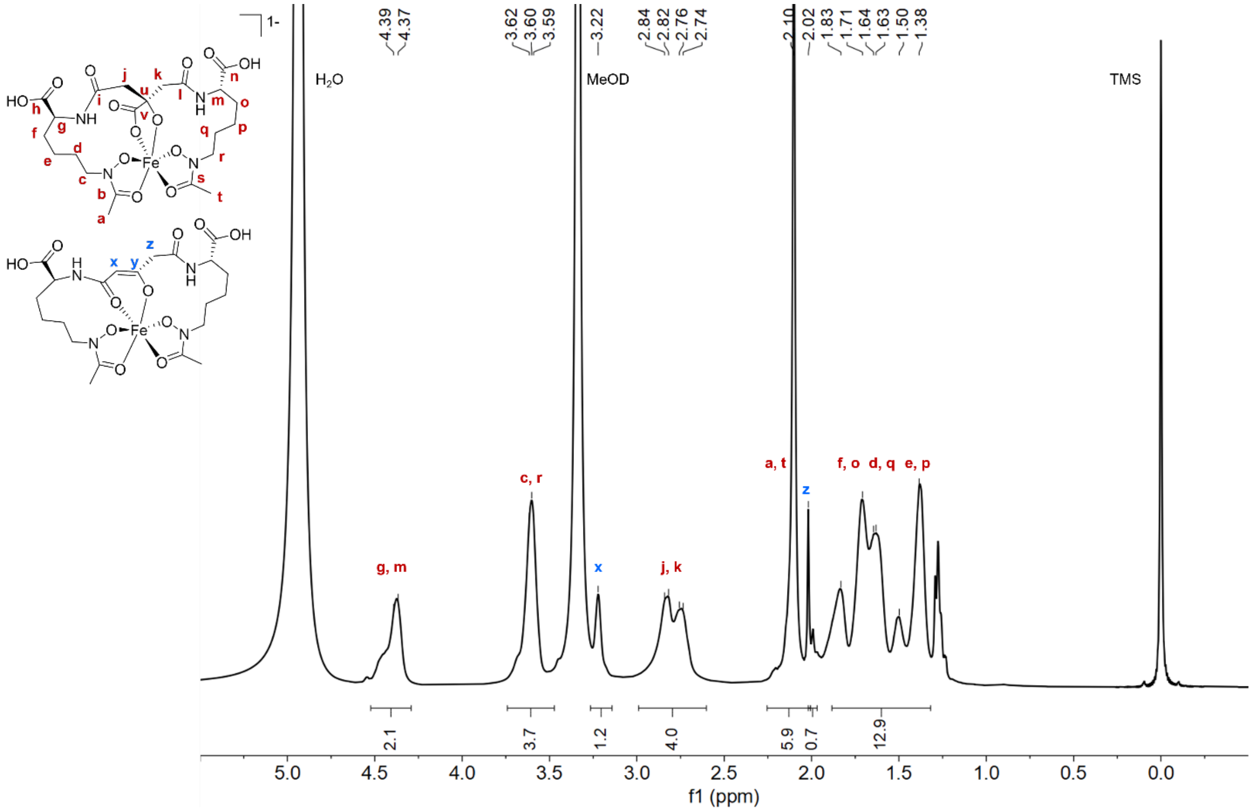

Figure S25. ^{1}H NMR (600 MHz) of **[Fe(AB*)]$^{2-}$** in MeOD-d$_4$.

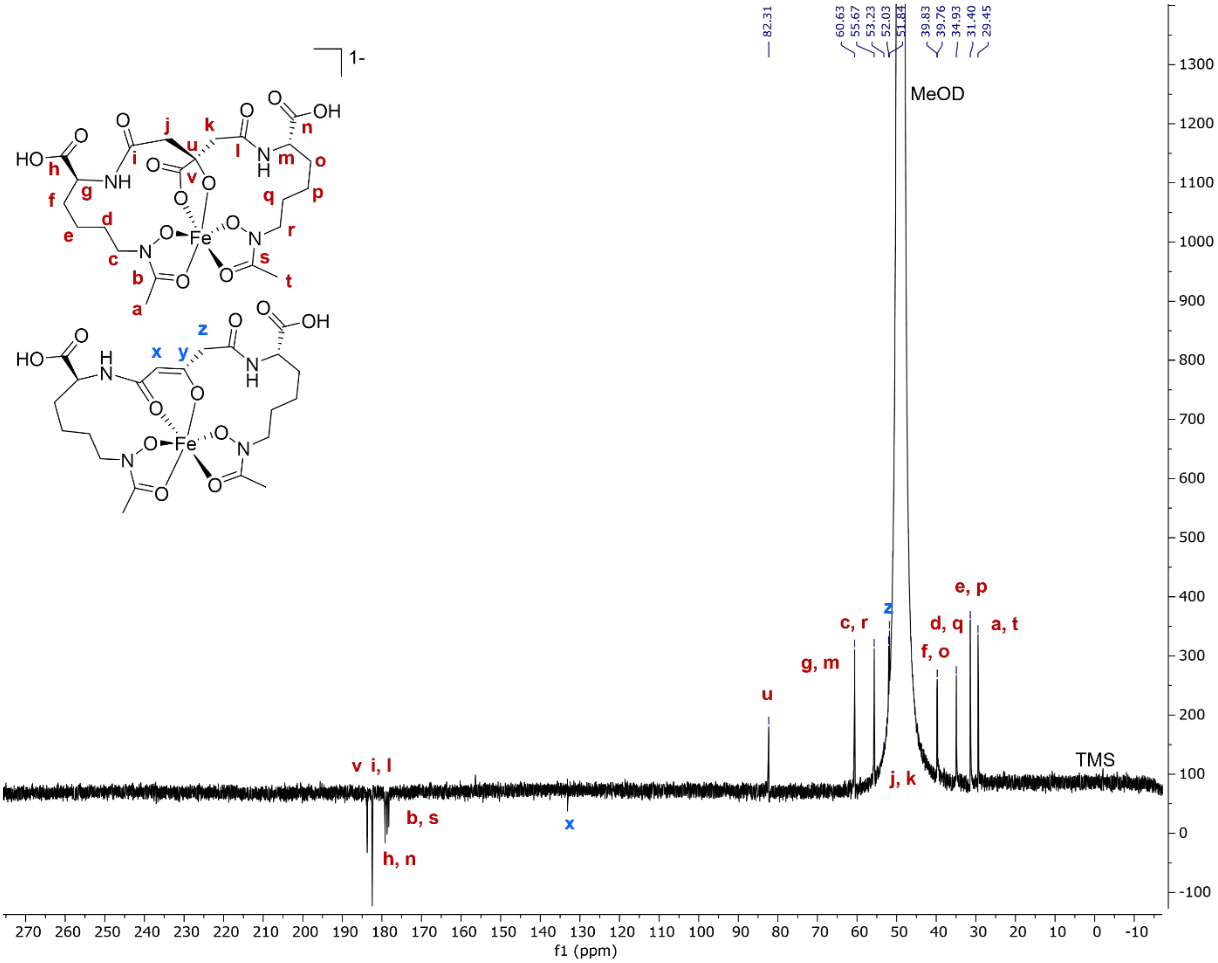

Figure S26. ${}^{13}C\{{}^{1}H\}$ NMR (151 MHz) of **[Fe(AB*)]$^{2-}$** in MeOD-d_4.

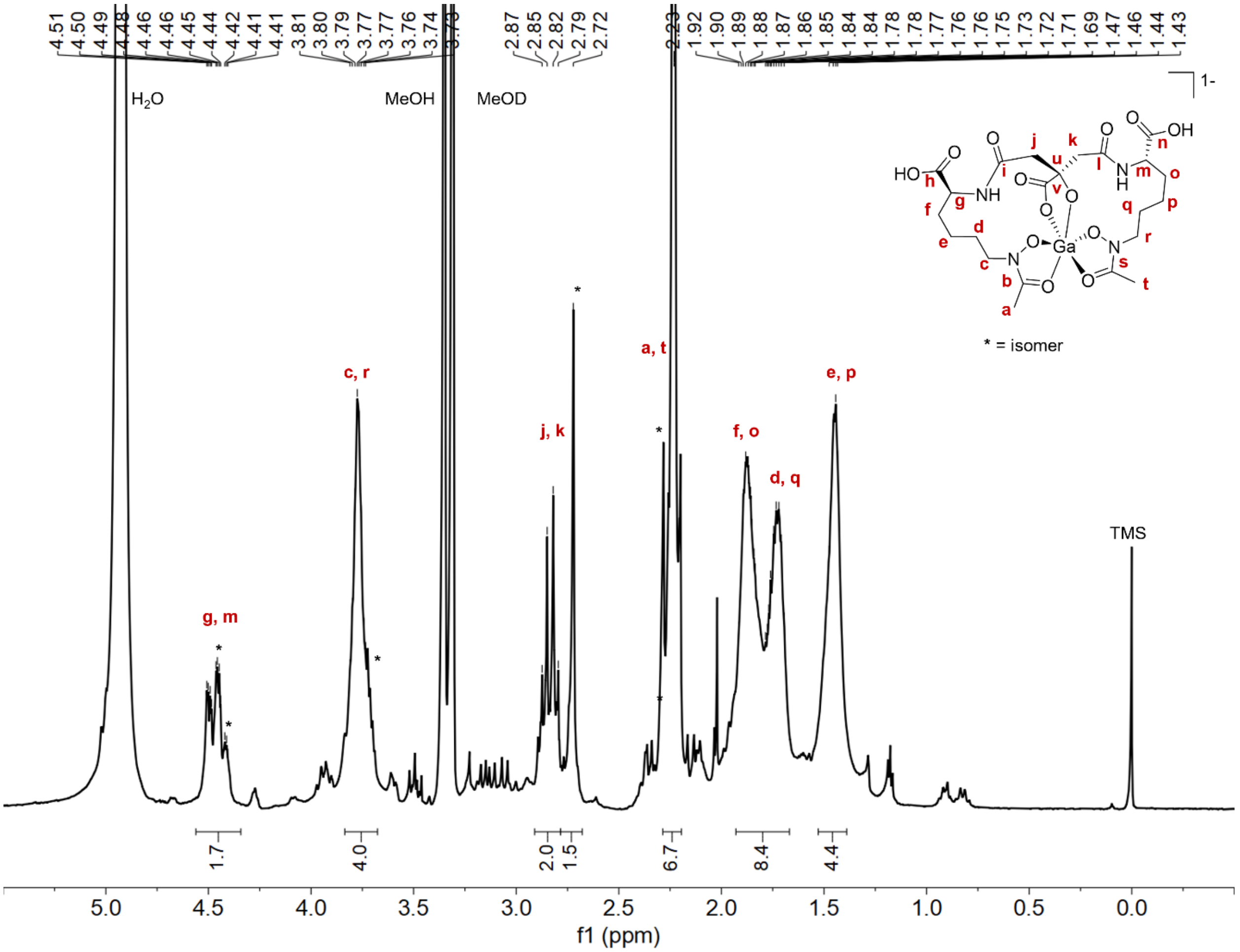

Figure S27. ^{1}H NMR (600 MHz) of **[Ga(AB)]$^{3-}$** in MeOD-d$_4$.

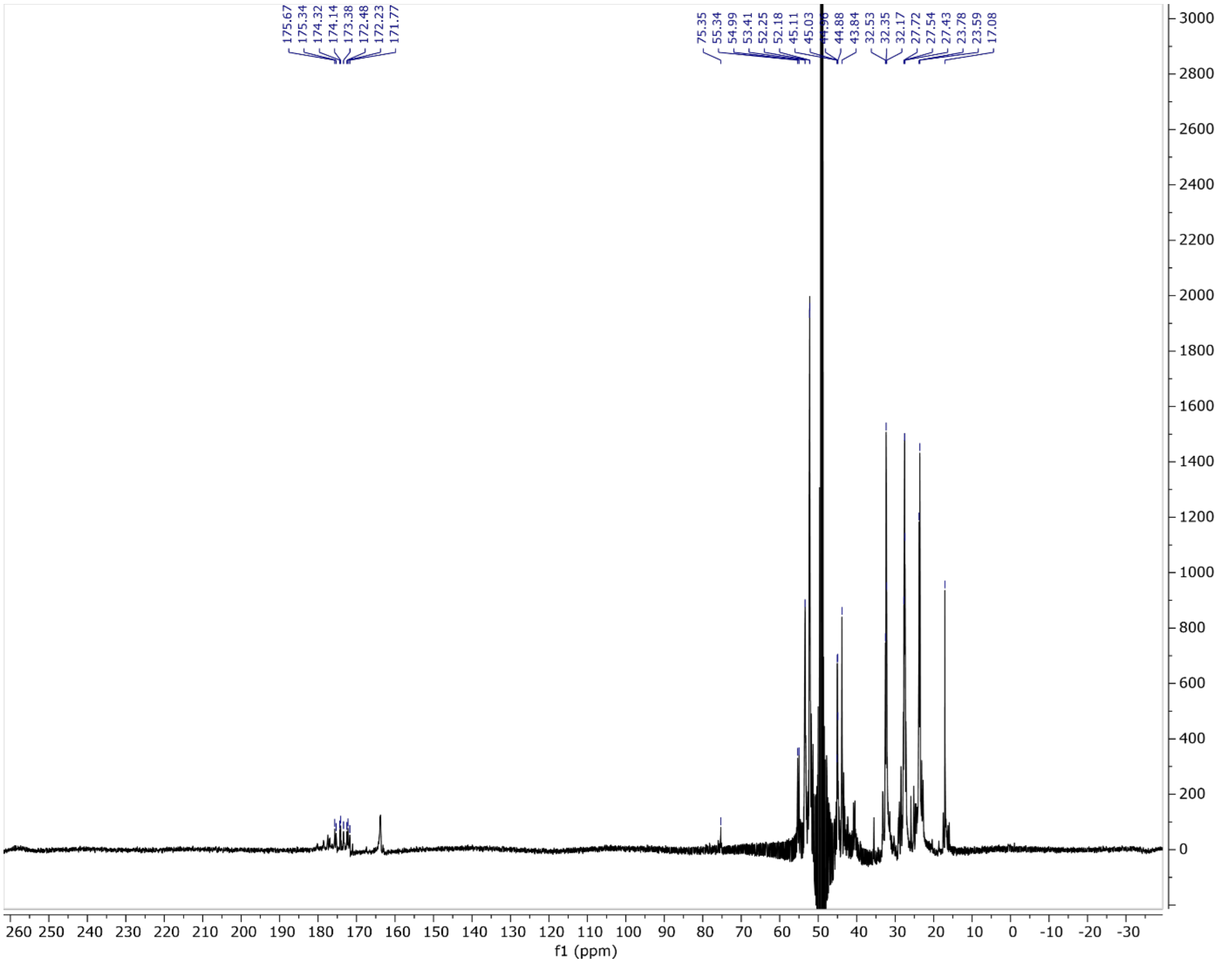

Figure S28. $^{13}C\{^{1}H\}$ NMR (151 MHz) of **$[Ga(AB)]^{3-}$** in MeOD-d_4.

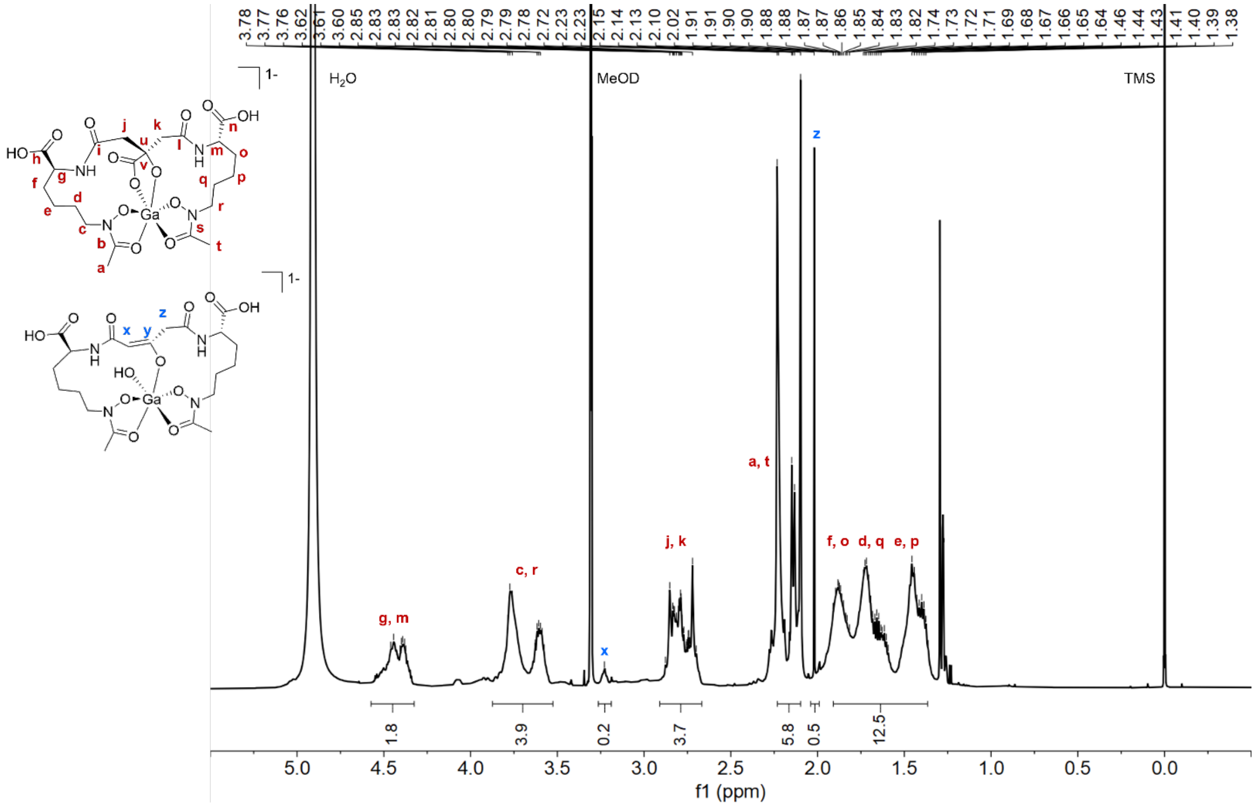

Figure S29. ^{1}H NMR (600 MHz) of **[Ga(AB*)]$^{2-}$** in MeOD-d_4.

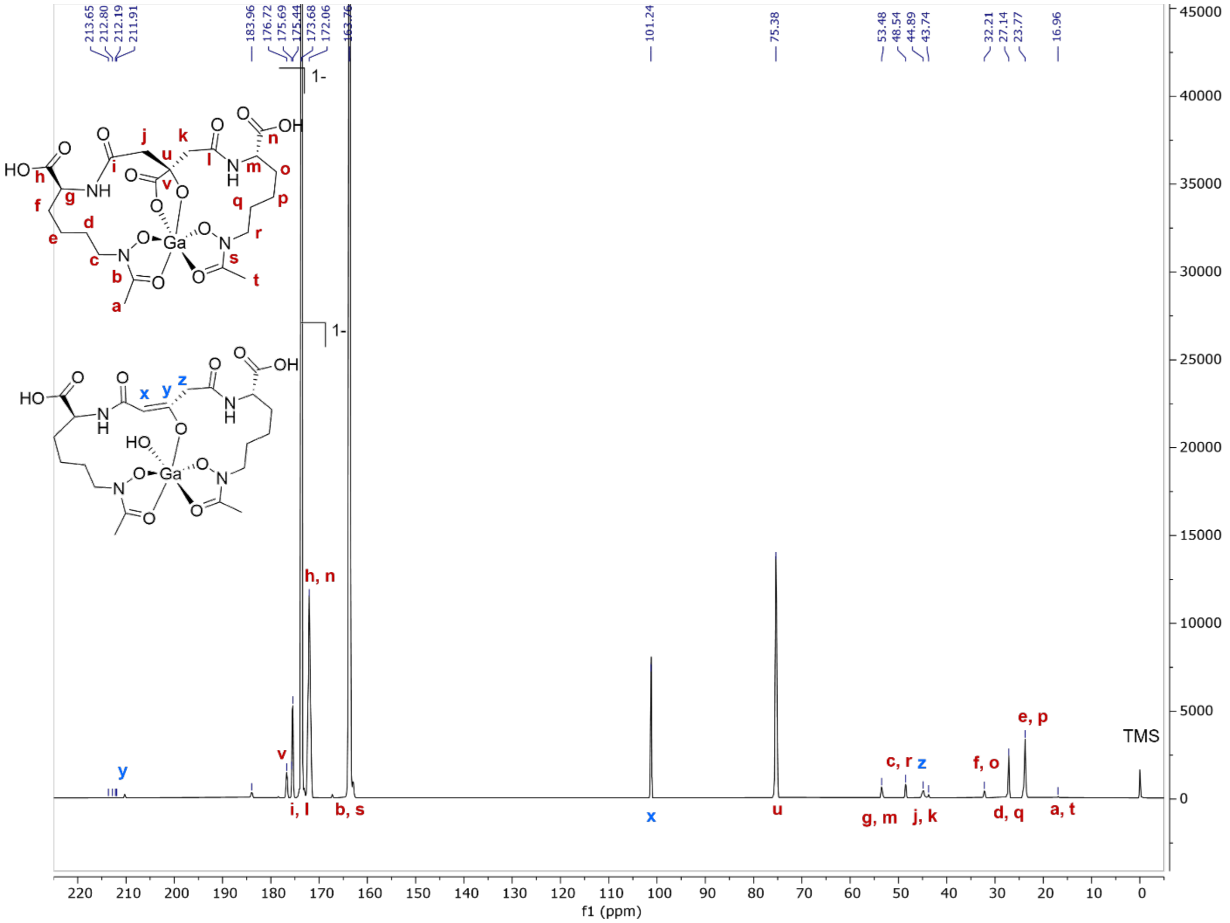

Figure S30. $^{13}C\{^{1}H\}$ NMR (151 MHz) of **$[Ga(AB^*)]^{2-}$** in MeOD-d_4.

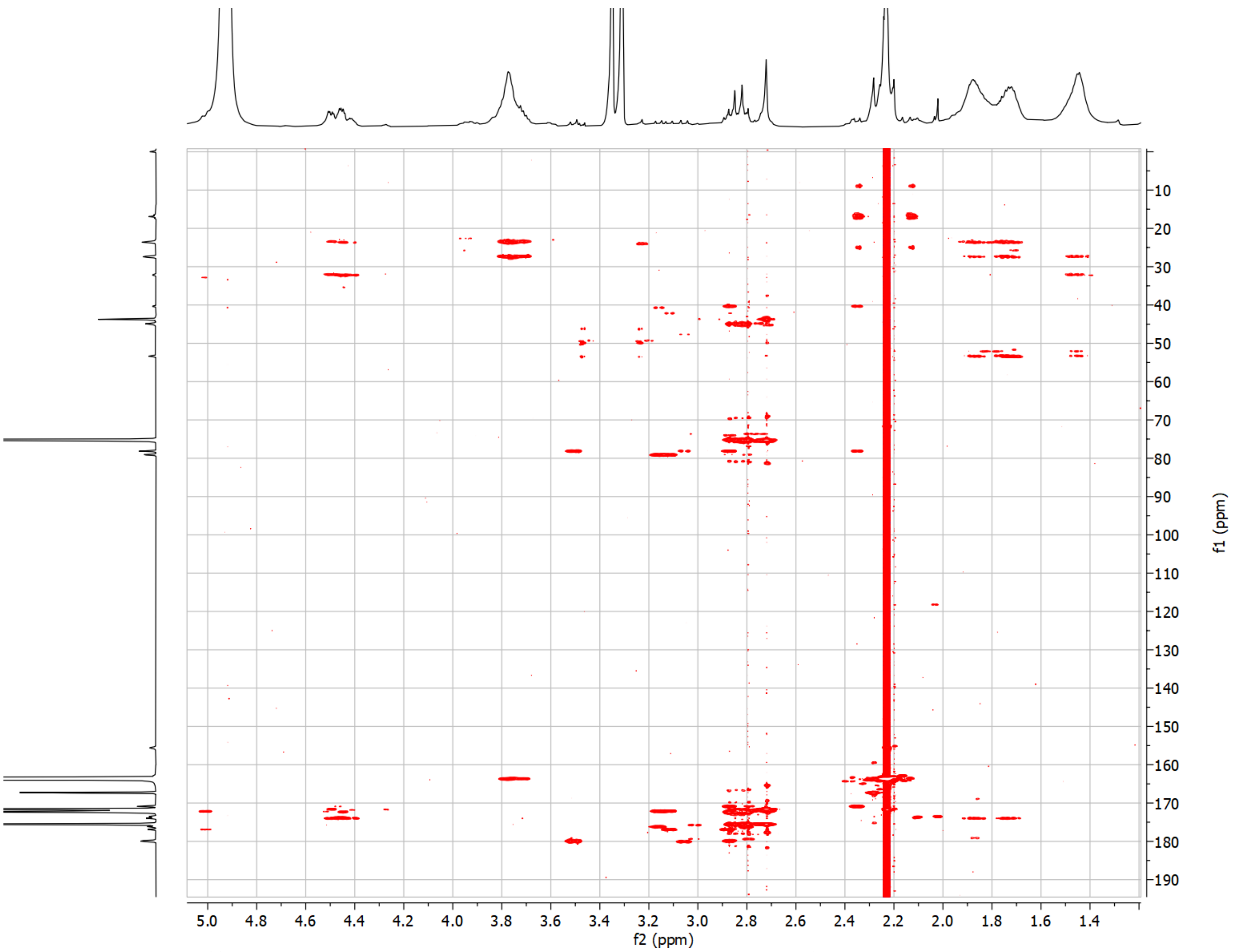

Figure S31. ^{1}H ^{13}C-HMBC NMR (500, 125 MHz) of **$[Ga(AB)]^{3-}$** in MeOD-d_4.

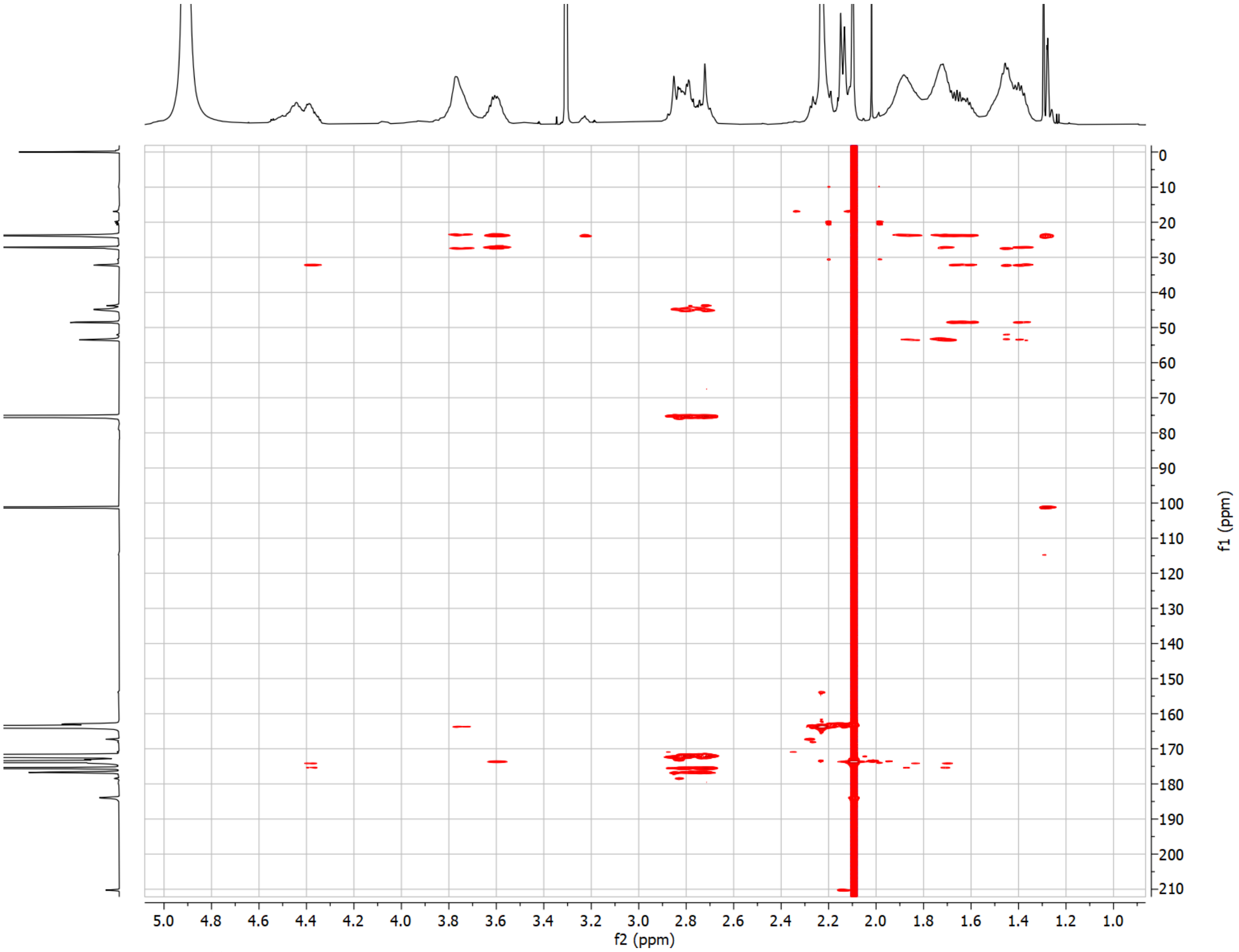

Figure S32. ^{1}H ^{13}C-HMBC NMR (500, 125 MHz) of **[Ga(AB)]$^{2-}$** in MeOD-d_4 following 254 nm irradiation.

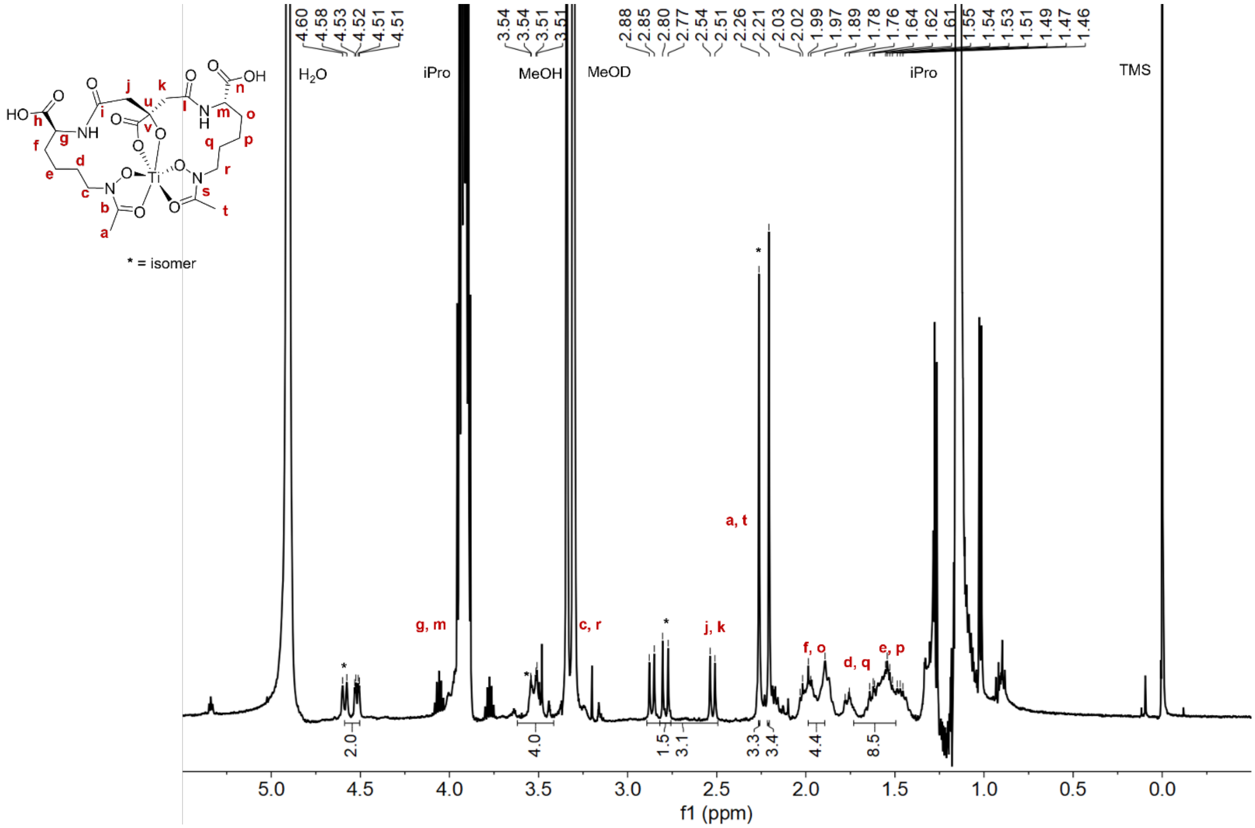

Figure S33. ^{1}H NMR (500 MHz) of **[Ti(AB)]$^{2-}$** in MeOD-d$_4$.

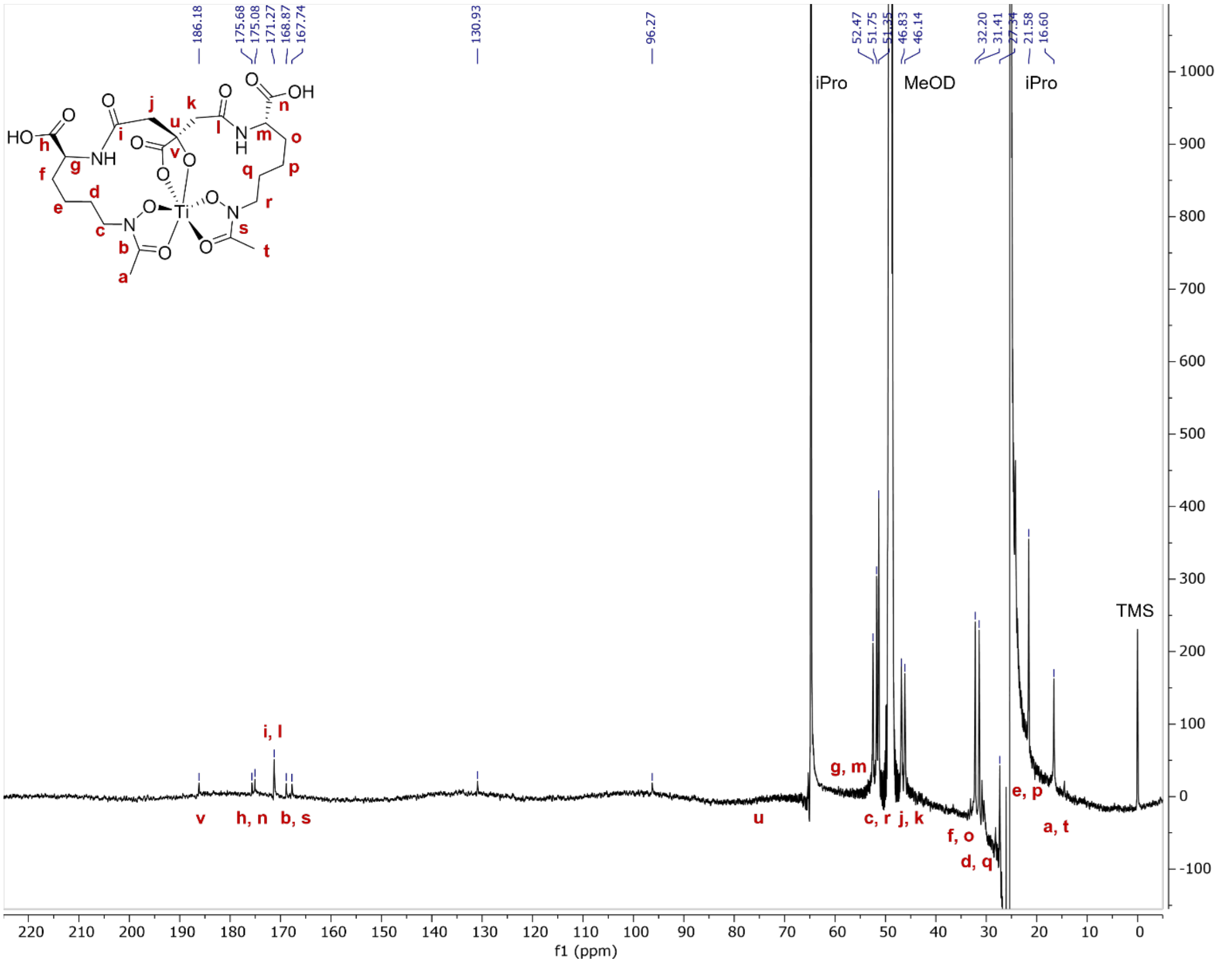

Figure S34. $^{13}C\{^{1}H\}$ NMR (126 MHz) of **$[Ti(AB)]^{2-}$** in MeOD-d_4.

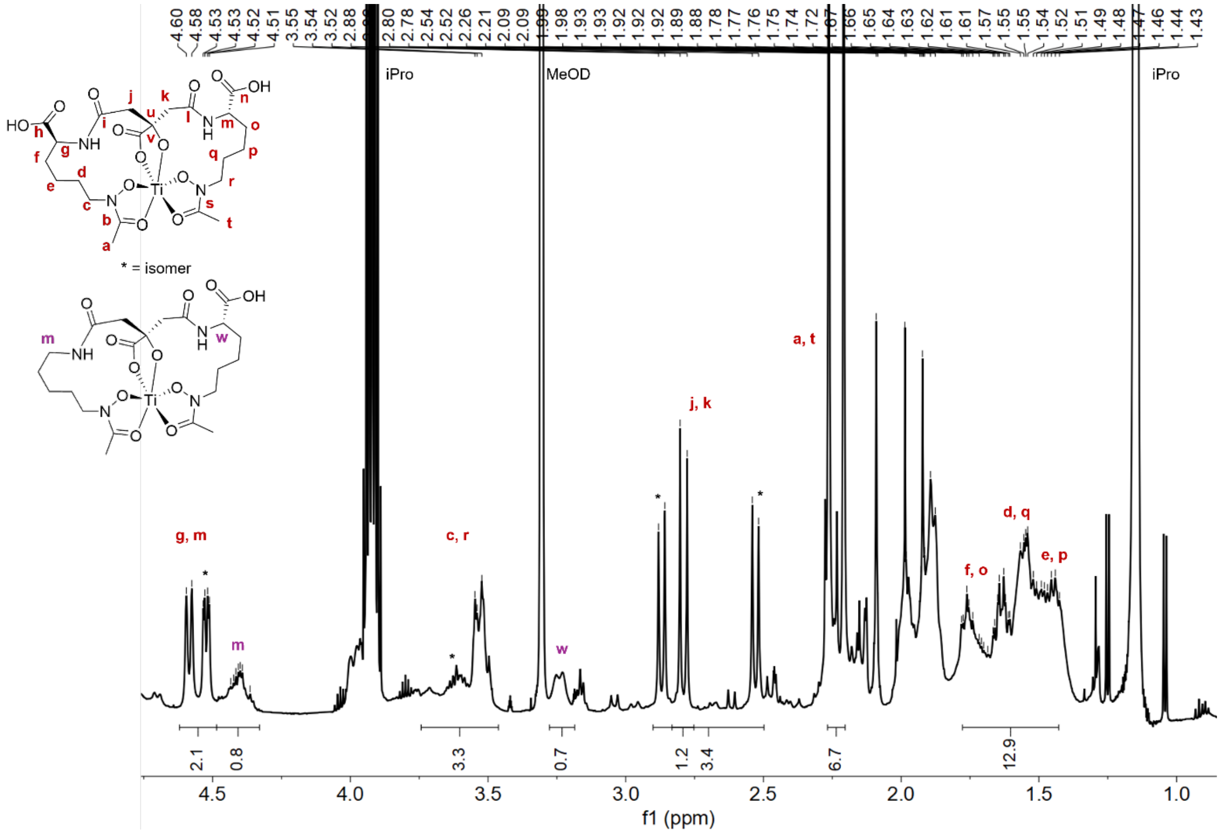

Figure S35. ^{1}H NMR (600 MHz) of **$[Ti(AB2^*)]^{2-}$** in MeOD-d_4.

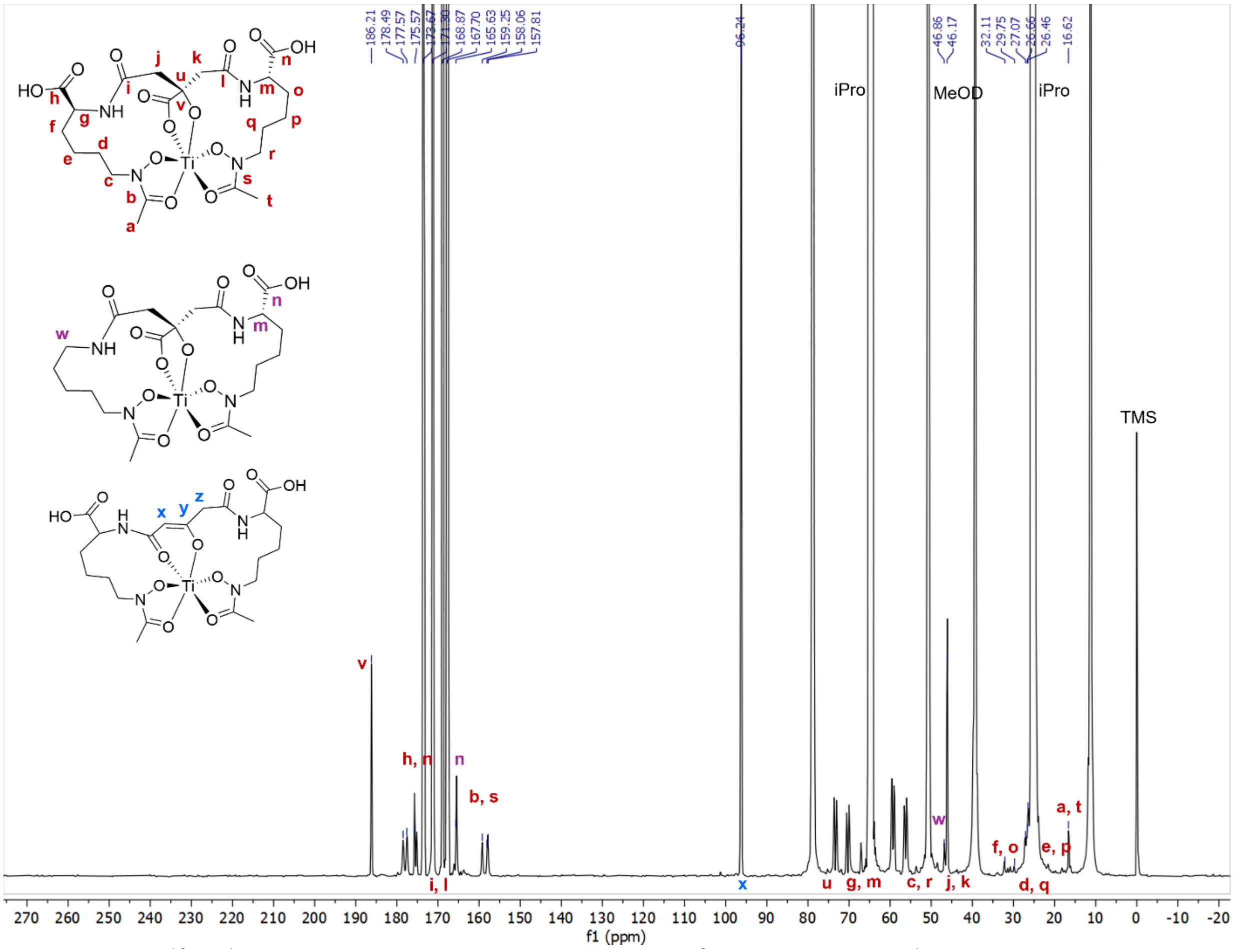

Figure S36. $^{13}C\{^1H\}$ NMR (151 MHz) of **[Ti(AB2*)]$^{2-}$** and **[Ti(AB*)]$^{1-}$** in MeOD-d$_4$.

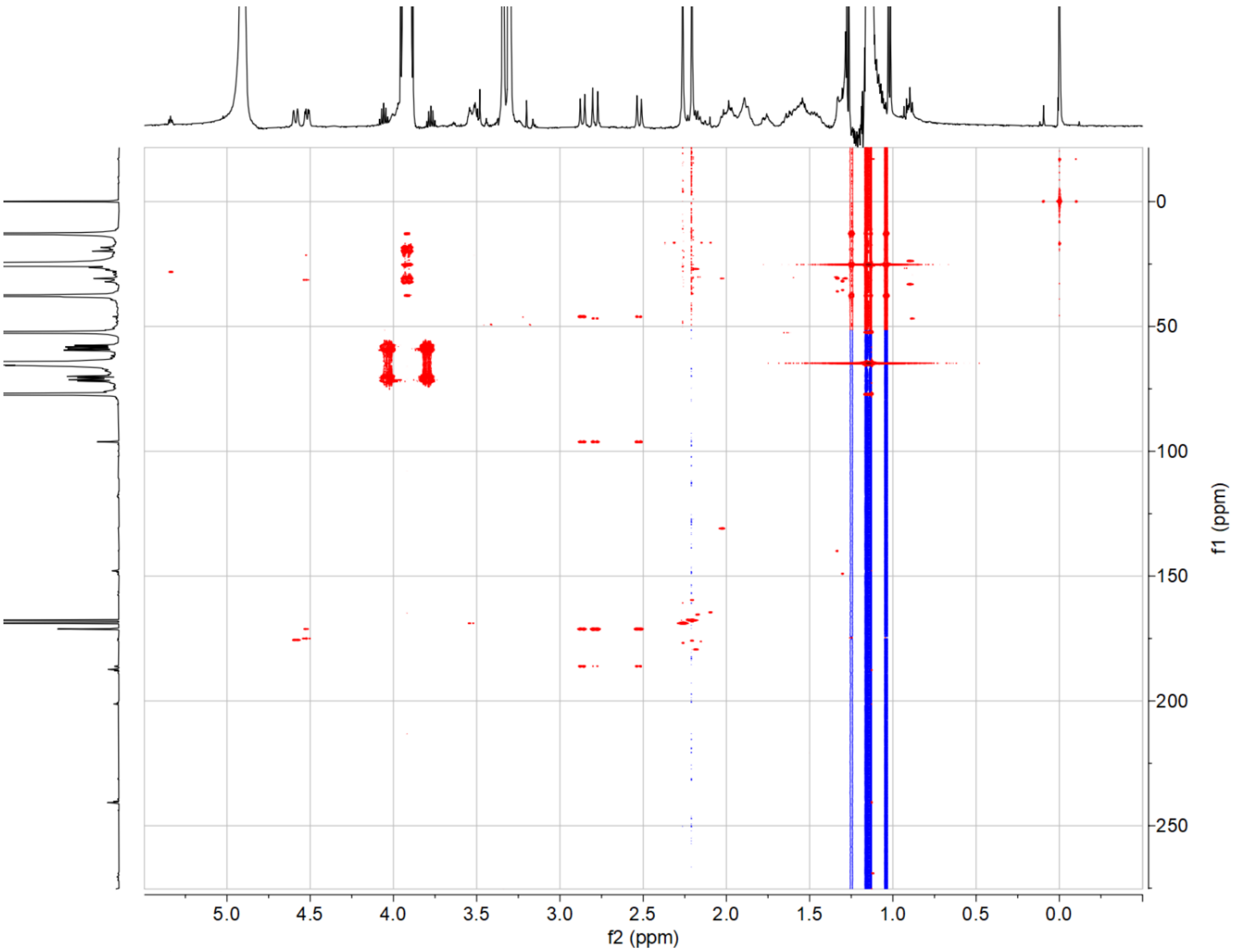

Figure S37. ^{1}H ^{13}C-HMBC NMR (500, 125 MHz) of **[Ti(AB)]$^{2-}$** in MeOD-d_4.

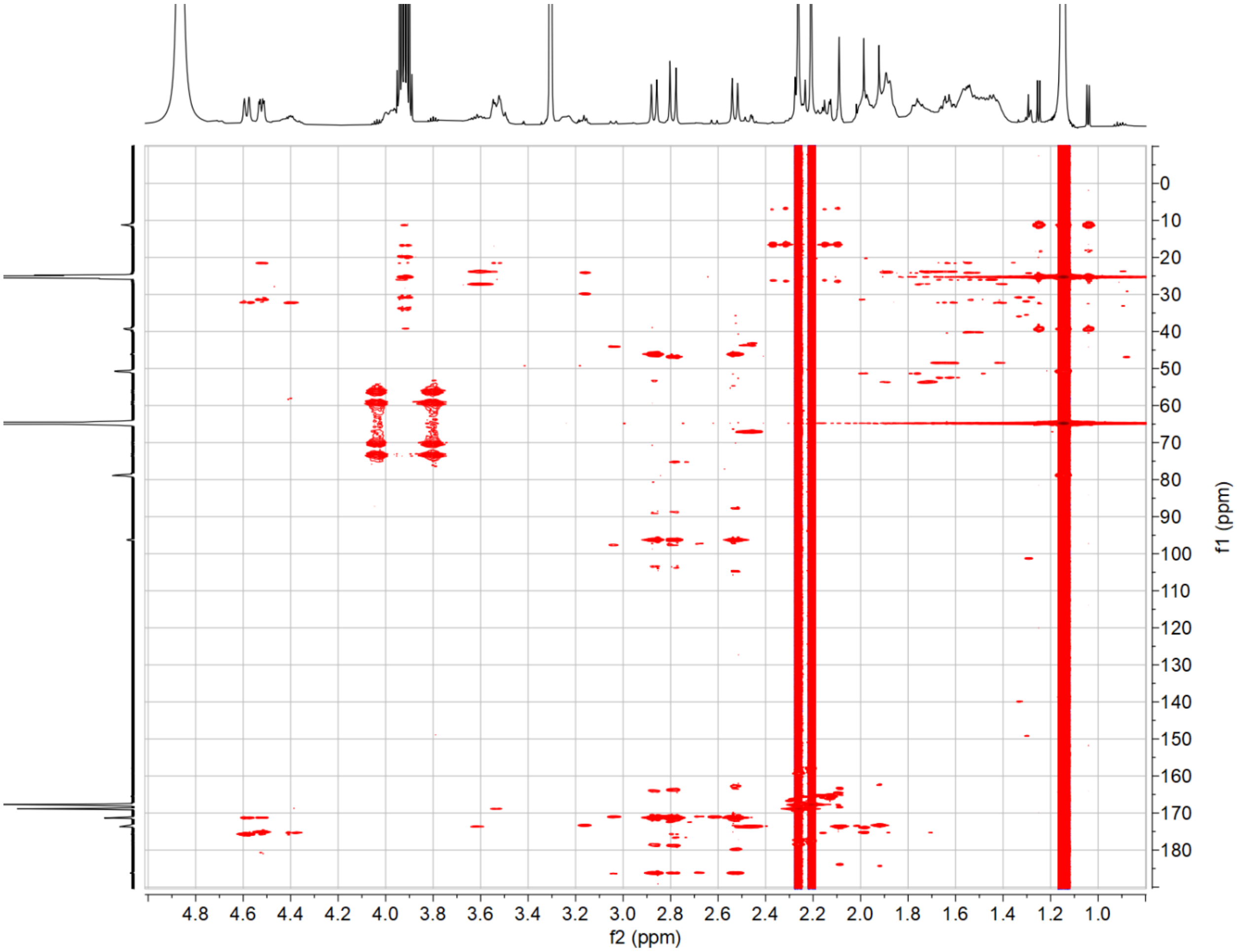

Figure S38. ^{1}H ^{13}C-HMBC NMR (500, 125 MHz) of **[Ti(AB2*)]$^{2-}$** in MeOD-d_4 following 254 nm irradiation.

Variable Temperature NMR Experiments

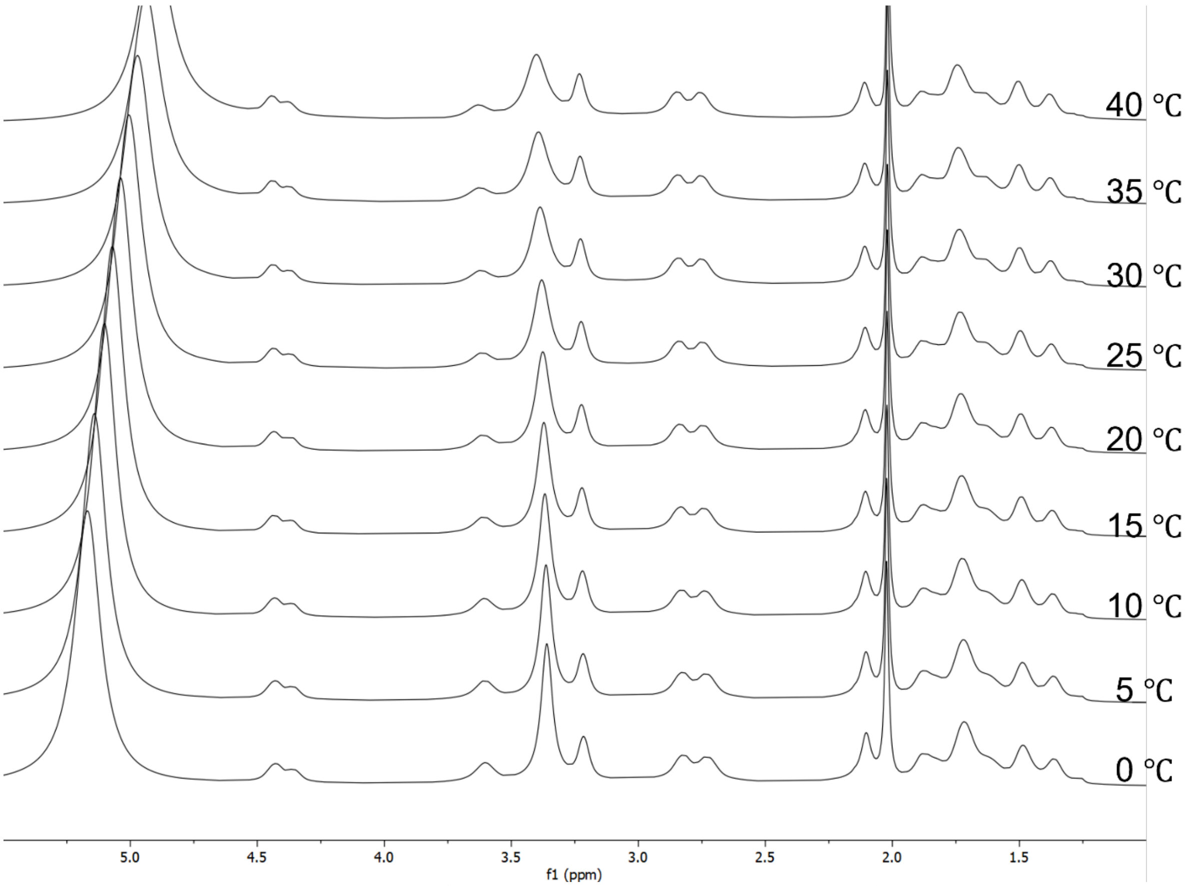

Figure S39. Variable temperature ^{1}H NMR (500 MHz) of **$[Fe(AB)]^{3-}$** in MeOD-d_4.

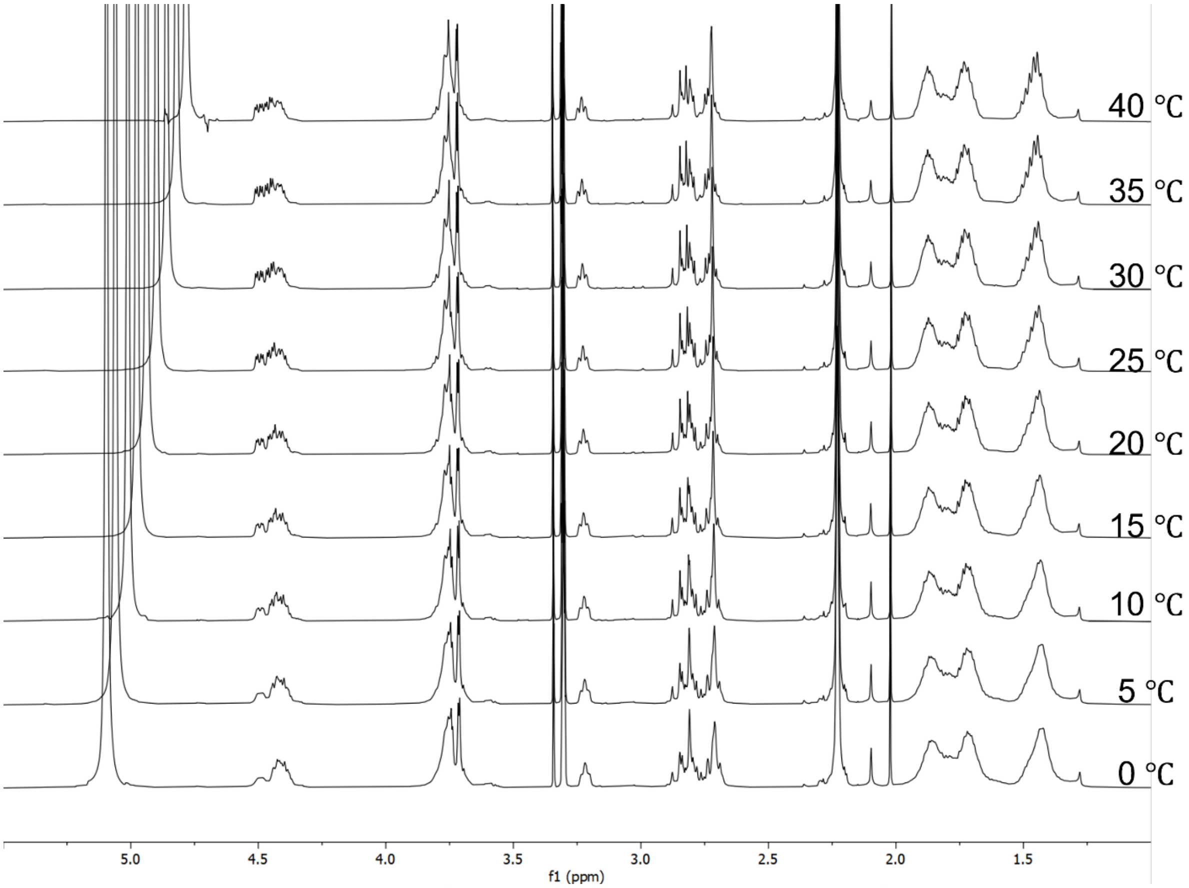

Figure S40. Variable temperature ^{1}H NMR (500 MHz) of **$[Ga(AB)]^{3-}$** in MeOD-d_4.

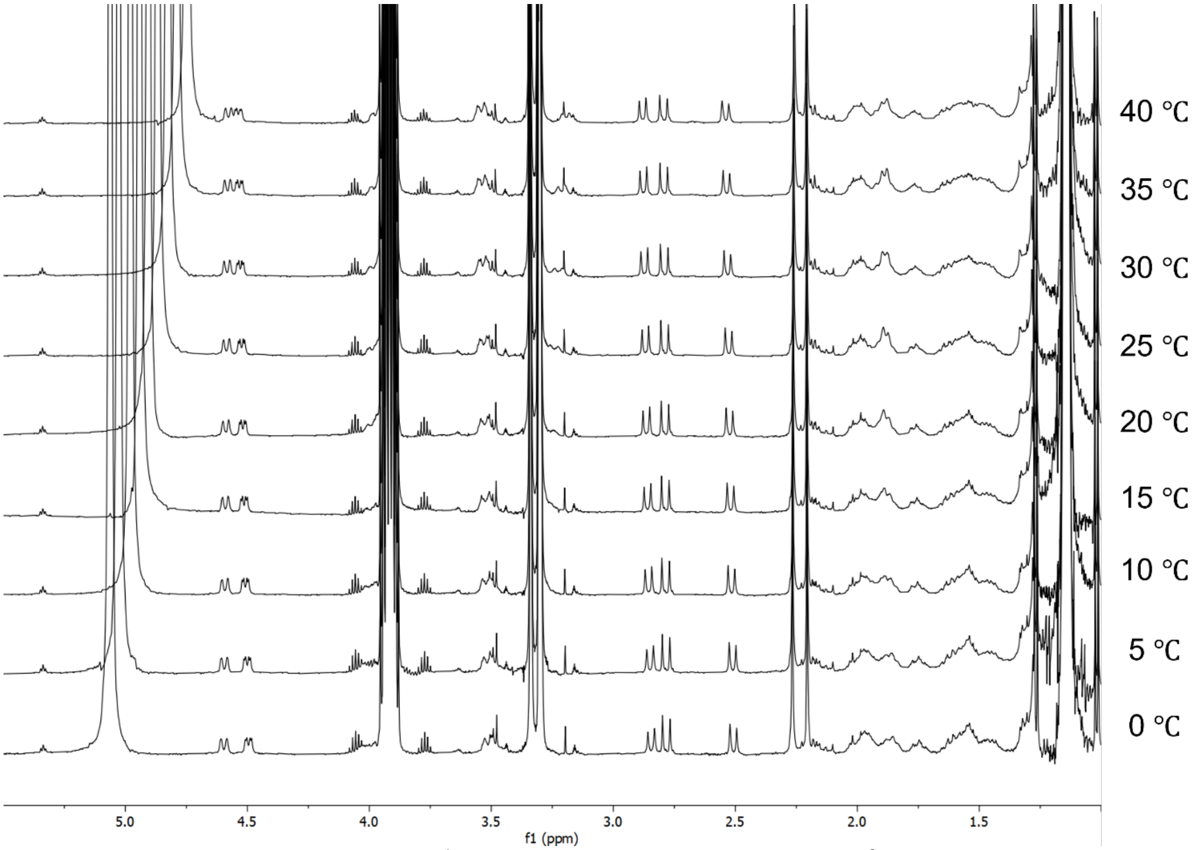

Figure S41. Variable temperature ^{1}H NMR (500 MHz) of **$[Ti(AB)]^{2-}$** in MeOD-d$_4$.

Time Dependent Photoirradiation Monitored by NMR

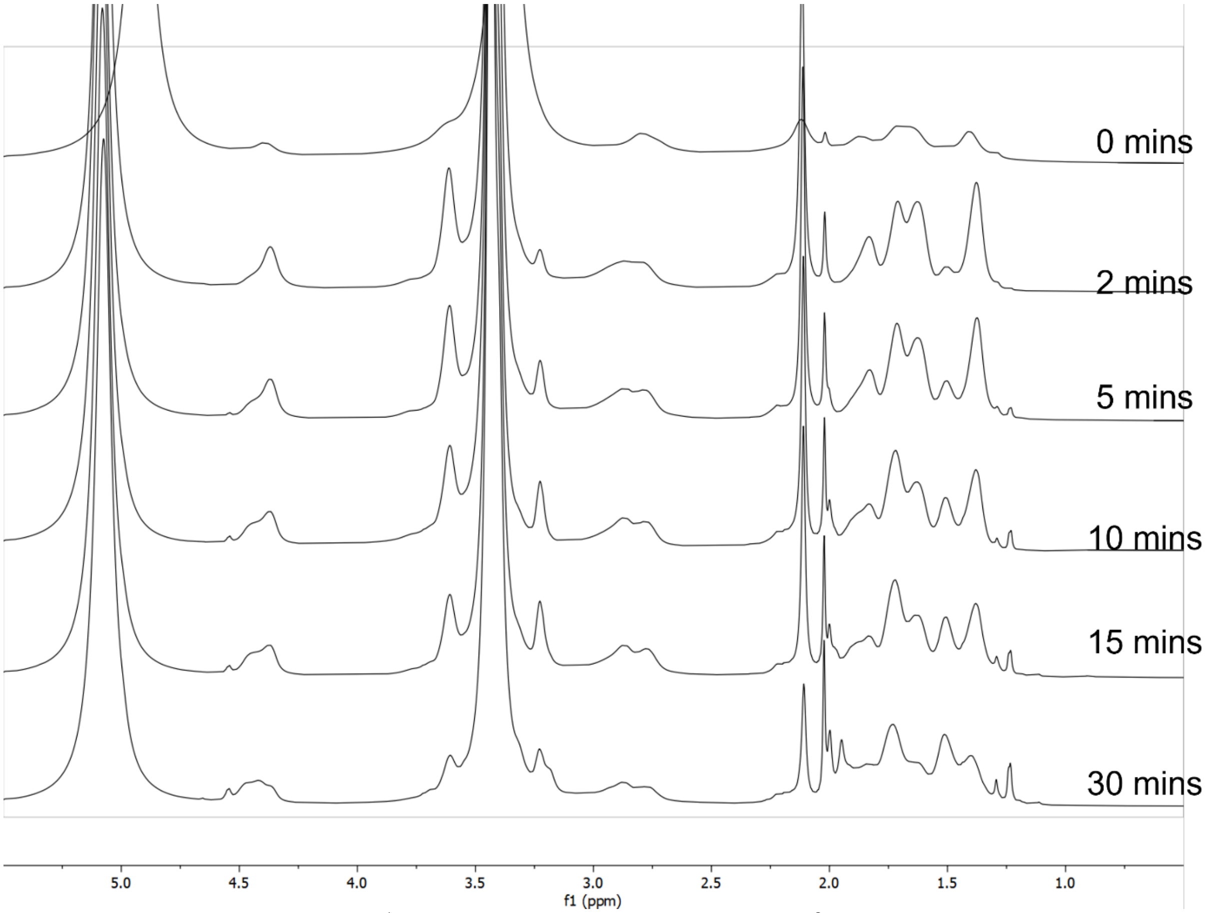

Figure S42. Time-dependent ^{1}H NMR (500 MHz) of **$[Fe(AB)]^{3-}$** in MeOD-d_4 following 254 nm irradiation.

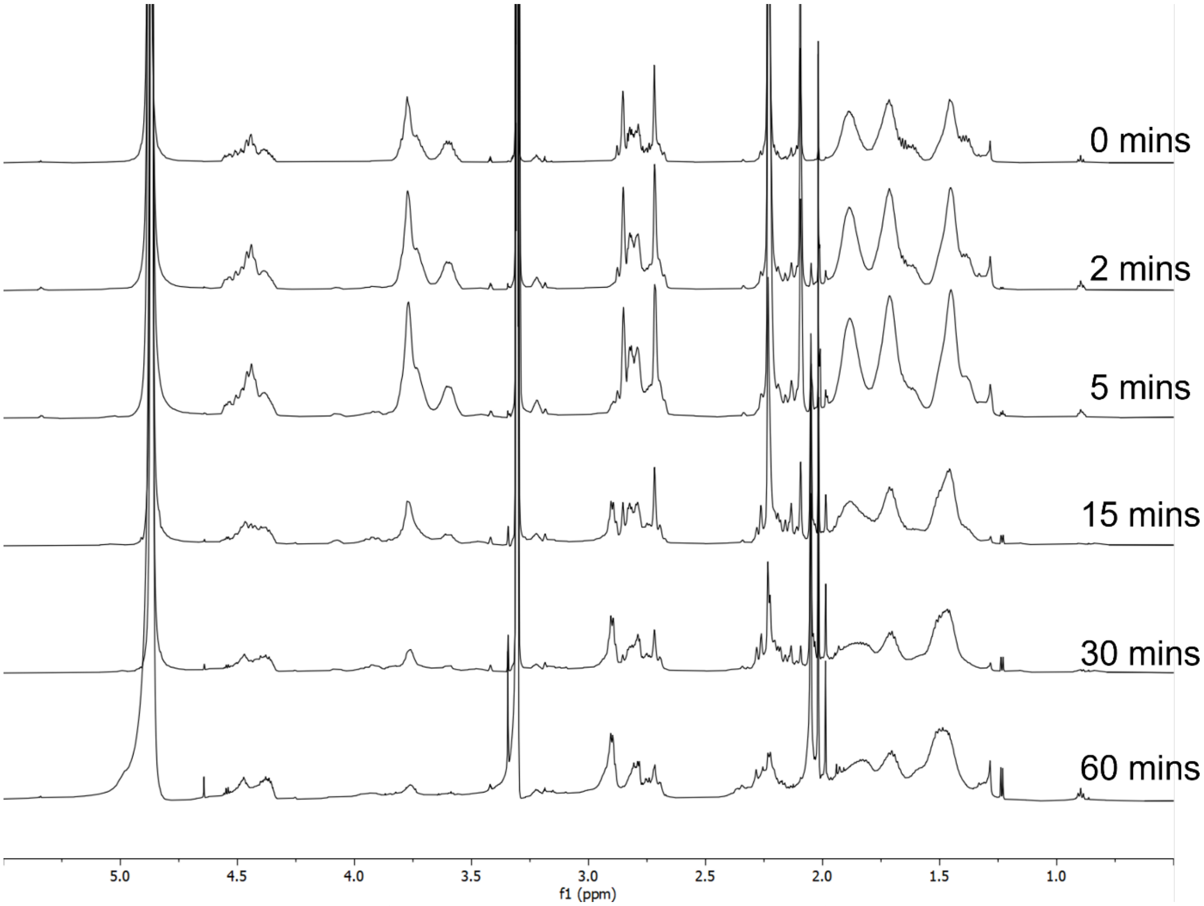

Figure S43. Time-dependent ^{1}H NMR (500 MHz) of **$[Ga(AB)]^{3-}$** in MeOD-d_4 following 254 nm irradiation.

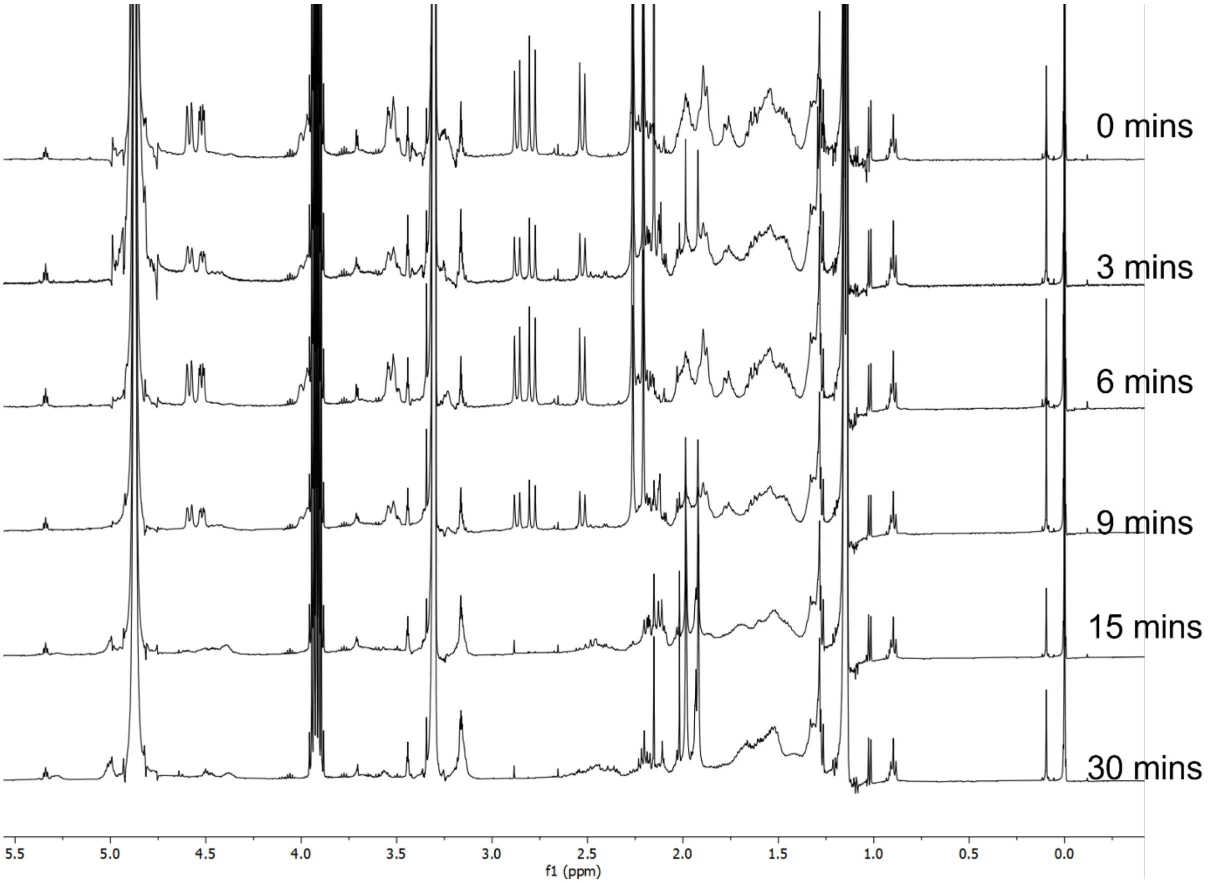

Figure S44. Time-dependent ^{1}H NMR (500 MHz) of **[Ti(AB)]$^{2-}$** in MeOD-d_4 following 300 nm irradiation.

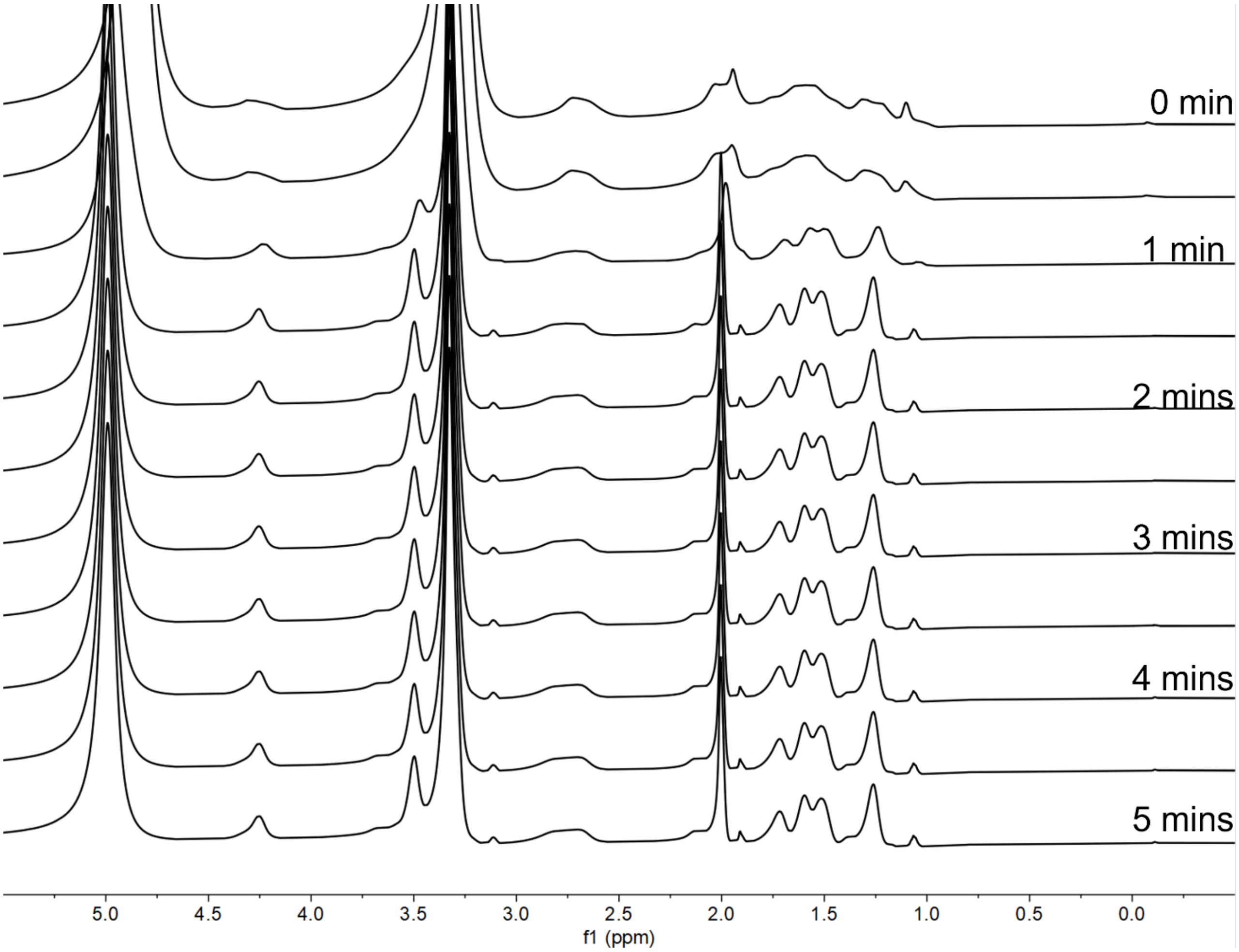

Figure S45. Time-dependent ^{1}H NMR (500 MHz) of **[Fe(AB)]$^{3-}$** in MeOD-d_4 following 365 nm irradiation by LED-NMR in 30 second increments.

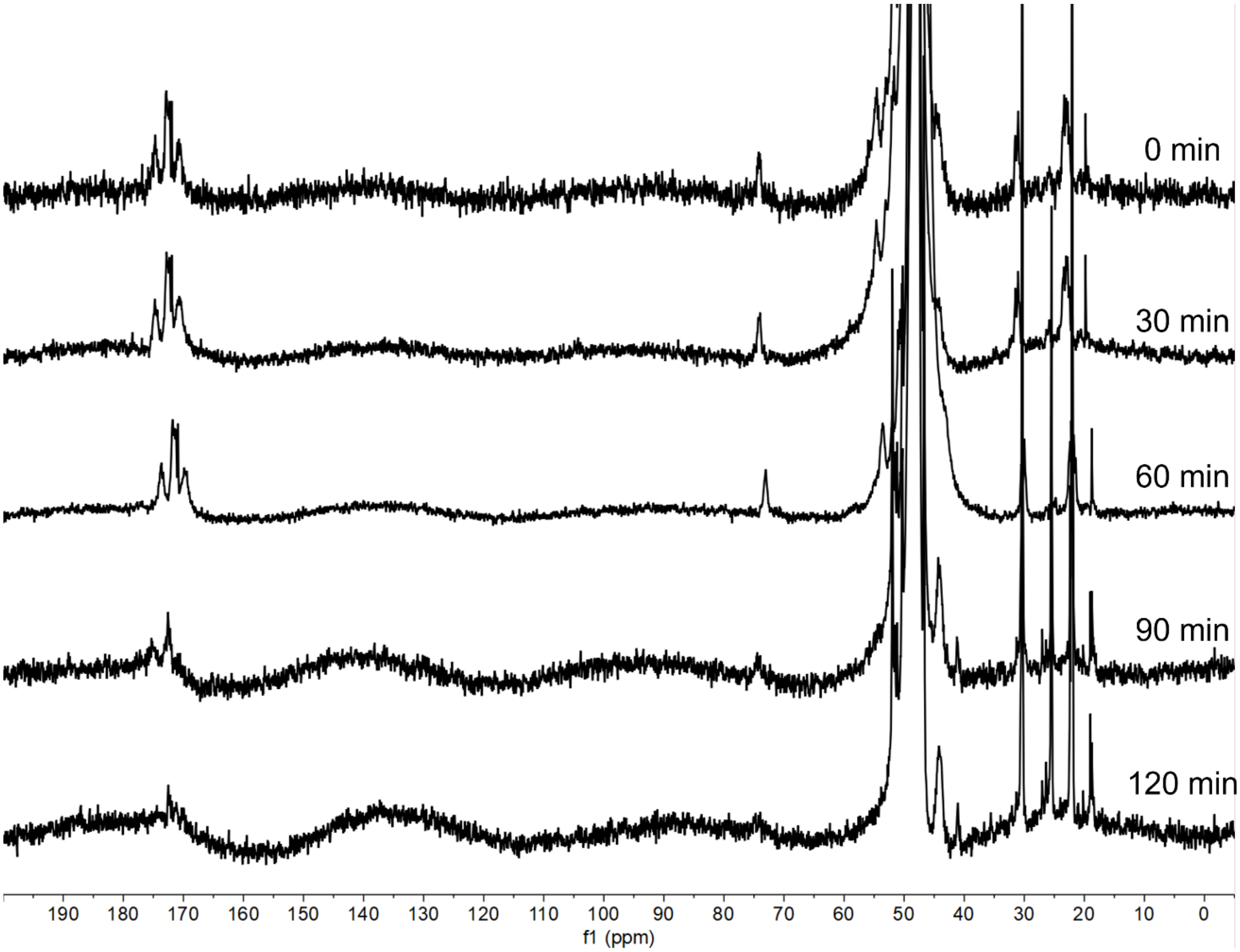

Figure S46. Time-dependent $^{13}C\{^{1}H\}$ NMR (125 MHz) of **$[Fe(AB)]^{3-}$** in MeOD-d_4 following 365 nm irradiation by LED-NMR in 30 min increments.

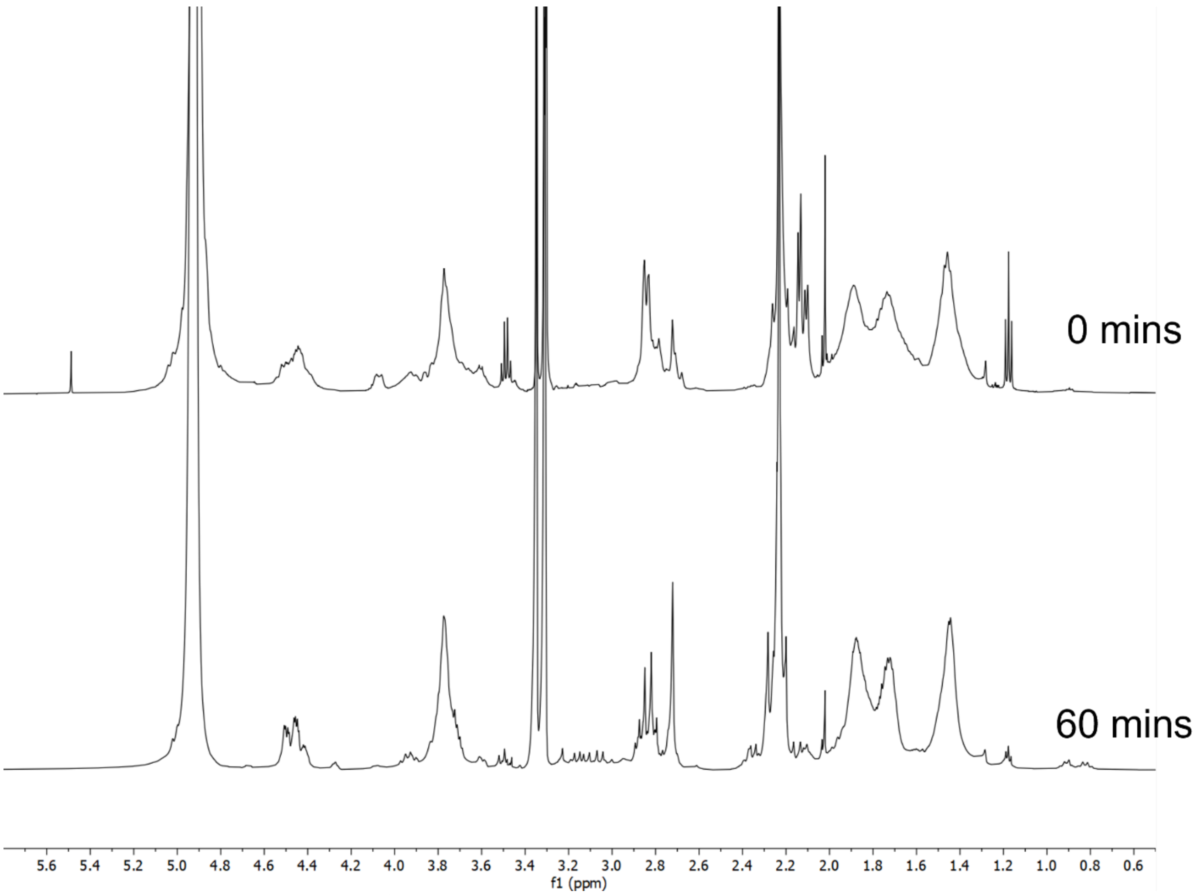

Figure S47. Time-dependent ^1H NMR (500 MHz) of **$[Ga(AB)]^{3-}$** in MeOD-d_4 following 300 nm irradiation.

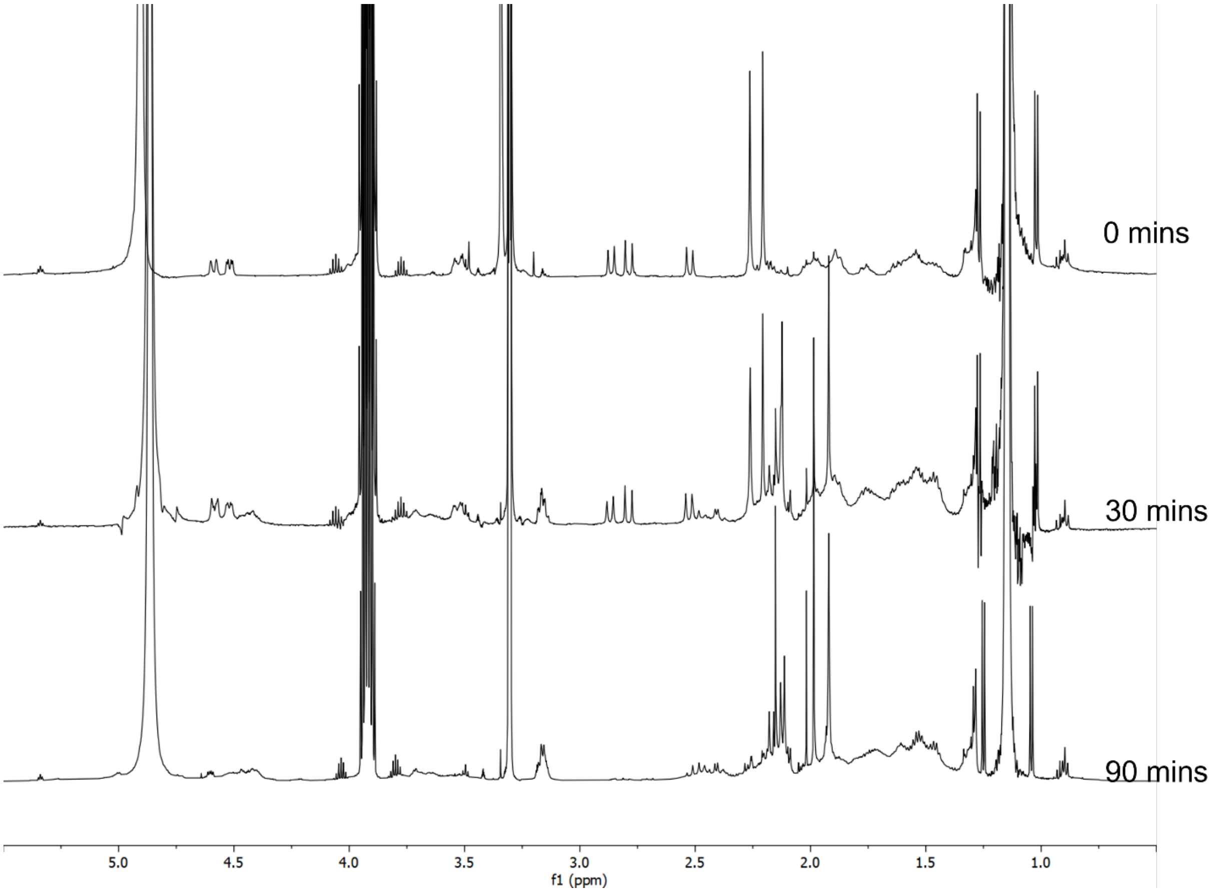

Figure S48. Time-dependent ^{1}H NMR (500 MHz) of **$[Ti(AB)]^{2-}$** in MeOD-d_4 following 300 nm irradiation.

Spectral Comparison Data Tables

Table S1. Tabulated ^{13}C chemical shift data for all apo- and metal aerobactin complexes.

Assignment	Aerobactin	$[Fe(AB)]^{3-}$	$[Ga(AB)]^{3-}$	$[Ti(AB)]^{2-}$
**v**	175.25	176	178.43	186.18
**h, n**	174.05	175.42	175.34	175.68
**i, l**	172.22	174.02	172.48	171.72
**b, s**	171	171.98	163.68	168.67
**u**	73.79	75.45	75.35	75.25
**g, m**	51.99	55.64	55.34	52.44
**c, r**	50	53.47	52.25	51.72
**j, k**	43.49	44.81	45.11	46.83
**f, o**	30.89	32.2	32.35	32.2
**d, q**	25.78	27.37	37.72	27.34
**e, p**	22.38	24.11	23.78	21.58
**a, t**	18.85	20	17.08	16.6

Table S2. Tabulated ^{1}H chemical shift data for all apo- and metal aerobactin photoproduct complexes.

Assignment	Aerobactin*						[Fe(AB*)]$^{2-}$					
	Shift	Range	H's	Integral	Class	J's	Shift	Range	H's	Integral	Class	J's
**g, m**	4.37	4.42 .. 4.36	1	1	s		4.38	4.41 .. 4.35	1	1	m	
**c, r**	3.6	3.66 .. 3.54	2	1.98	dq	6.92, 6.92, 13.83 13.83 13.83	3.6	3.63 .. 3.58	2	2	m	
**x**	3.16	3.18 .. 3.14	0	0.09	m		3.22	3.27 .. 3.17	1	1.17	m	
**j, k**	2.78	2.79 .. 2.78	1	0.81	s		2.79	2.86 .. 2.72	2	2.46	m	
	2.77	2.82 .. 2.70	2	2.01	dd	47.12, 14.85						
**z**	2.15	2.17 .. 2.14	1	0.77	s		2.1	2.12 .. 2.09	3	3.31	s	
**a, t**	2.09	2.11 .. 2.08	3	2.83	s		2.02	2.03 .. 2.00	1	0.61	s	
**f, o**	1.93	1.93 .. 1.92	0	0.07	s							
	1.82	1.91 .. 1.69	3	2.52	m							
**d, q**	1.64	1.69 .. 1.57	2	1.97	m		1.62	1.85 .. 1.37	11	11.15	m	
**e, p**	1.4	1.44 .. 1.36	2	1.99	p	8.26, 8.26, 7.62, 7.62						

Assignment	[Ga(AB*)]$^{2-}$						[Ti(AB2*)]$^{2-}$					
	Shift	Range	H's	Integral	Class	J's	Shift	Range	H's	Integral	Class	J's
**g, m**	4.44	4.48 .. 4.43	1	0.58	s		4.59	4.61 .. 4.56	1	1	d	11.89
	4.38	4.42 .. 4.36	1	0.65	d	10.68	4.52	4.54 .. 4.50	1	1.02	dd	3.63, 9.81
**m**							4.4	4.45 .. 4.35	1	0.71	m	

	3.77	3.80 .. 3.74	1	1.42	m							
**c, r**							3.53	3.56 .. 3.50	2	1.7	d	15.16
	3.6	3.65 .. 3.56	1	1.15	dq	6.71, 6.71, 6.73, 13.03						
**x**	3.23	3.26 .. 3.21	0	0.16	s		2.79	2.81 .. 2.77	1	1.09	d	15.36
							2.7	2.89 .. 2.51	3	3.34	dd	13.59, 204.14
**j, k**	2.78	2.88 .. 2.67	4	3.67	m							
							2.26	2.27 .. 2.25	3	3.15	s	
**z**	2.23	2.25 .. 2.20	4	3.58	s		2.21	2.22 .. 2.20	3	3.07	s	
**a, t**	2.15	2.16 .. 2.14	1	0.83	s		2.09	2.10 .. 2.08	1	0.91	s	
	2.13	2.14 .. 2.12	1	0.83	s		1.99	2.00 .. 1.97	1	1.41	s	
**f, o**												
	2.1	2.11 .. 2.08	2	1.72	s							
**d, q**	2.02	2.03 .. 2.00	0	0.44	s		1.61	1.81 .. 1.39	14	14.35	m	
**e, p**	1.68	1.92 .. 1.35	13	13.04	m							

Table S3. Tabulated ^{13}C chemical shift data for all apo- and metal aerobactin photoproduct complexes.

Assignment	AB*	$[Fe(AB^*)]^{2-}$	$[Ga(AB^*)]^{2-}$	$[Ti(AB2^*)]^{2-}$
**y**	210.13		210.27	
**v**	176.66	183.51	175.62	186.21
**h, n**	173.67	182.58	173.68	175.7
**i, l**	172.47	178.84	172.06	173.67
**m**				165.63
**b, s**	171.92	178.01	163.76	159.25
**x**	100	133.11	101.24	96.24
**u**	75.67	82.39	75.38	
**g, m**	53.37	60.41	53.48	
**c, r**	48.55	55.74	48.54	
**z**	44.96	51.8	44.79	
**w**				46.86
**j, k**	44.33		43.74	46.17
**f, o**	32	39.77	32.44	32.11
**d, q**	24.1	34.79	27.14	29.75
**e, p**	23.8	31.37	23.77	26.46
**a, t**	20.38	29.09	16.96	16.62

UV-Vis Data

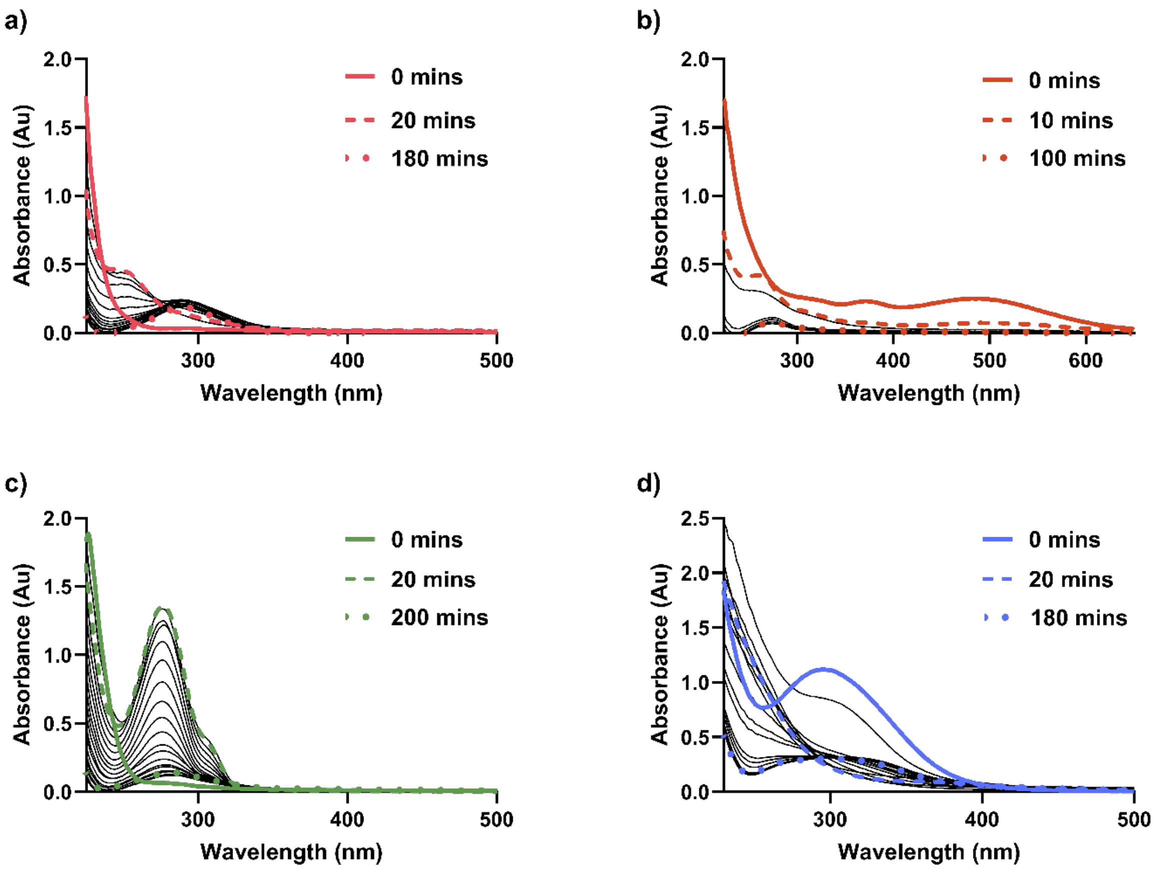

Figure S49. UV-vis spectra monitoring 254 nm photocleavage of **a)** AB, **b)** $[Fe(AB)]^{3-}$, **c)** $[Ga(AB)]^{3-}$, and **d)** $[Ti(AB)]^{2-}$. Starting material shown as solid line, photoproduct shown as dashed line, and degradation product shown with dotted line.

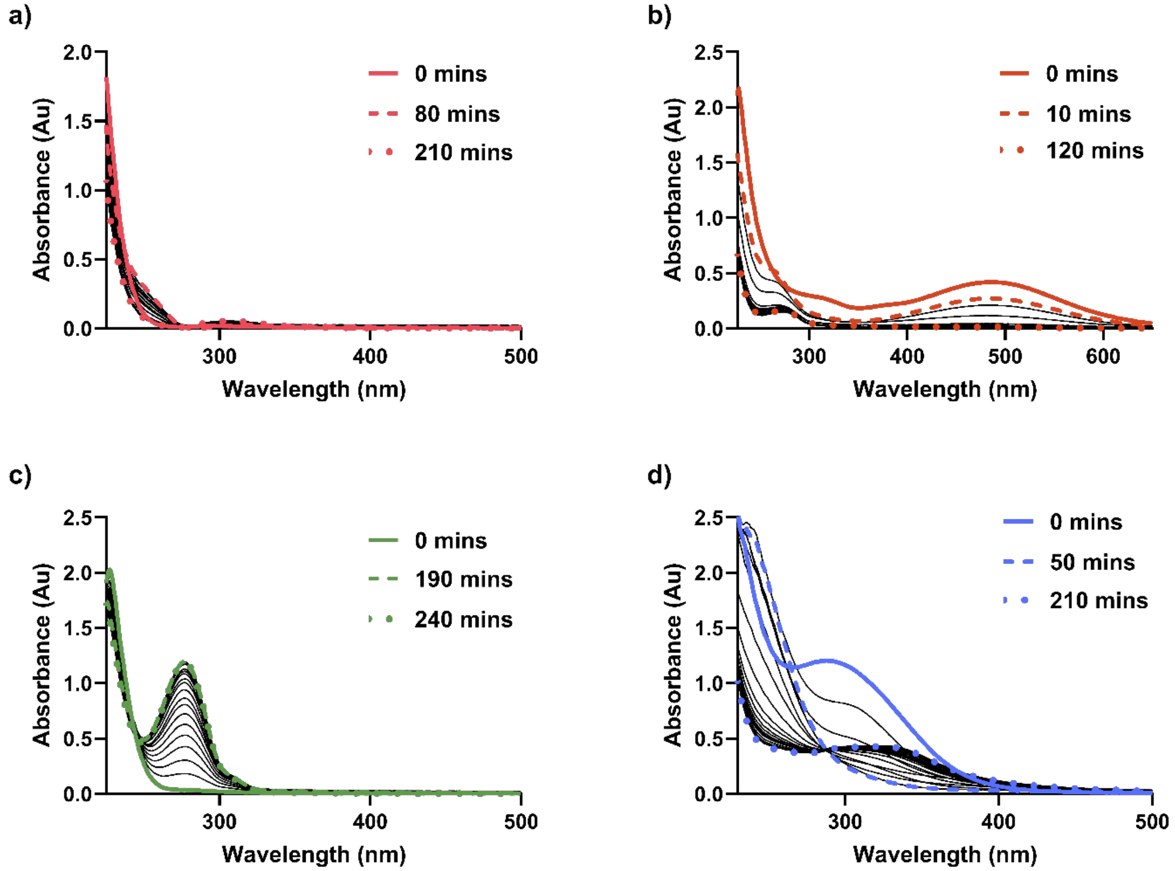

Figure S50. UV-vis spectra monitoring 300 nm photocleavage of **a)** AB, **b)** $[Fe(AB)]^{3-}$, **c)** $[Ga(AB)]^{3-}$, and **d)** $[Ti(AB)]^{2-}$. Starting material shown as solid line, photoproduct shown as dashed line, and degradation product shown with dotted line.

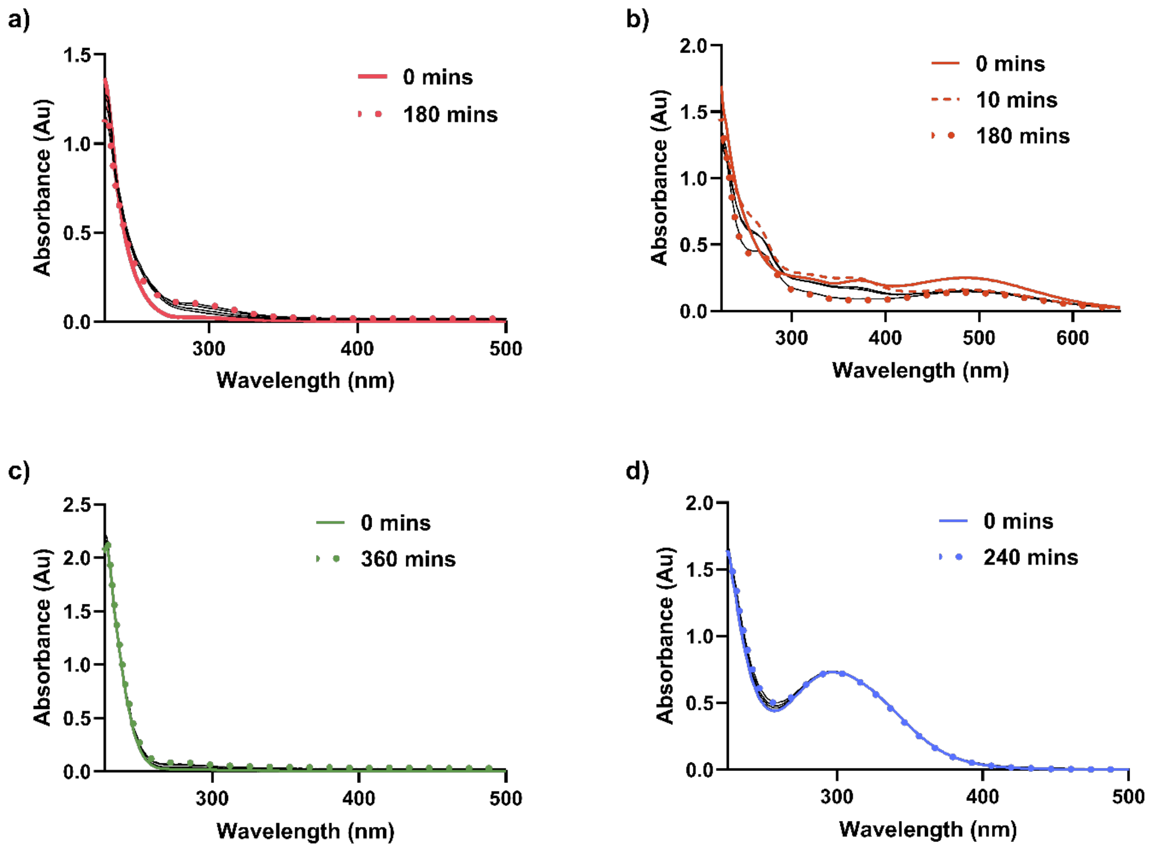

Figure S51. UV-vis spectra monitoring 350 nm photocleavage of **a)** AB, **b)** $[Fe(AB)]^{3-}$, **c)** $[Ga(AB)]^{3-}$, and **d)** $[Ti(AB)]^{2-}$. Starting material shown as solid line, photoproduct shown as dashed line, and degradation product shown with dotted line.

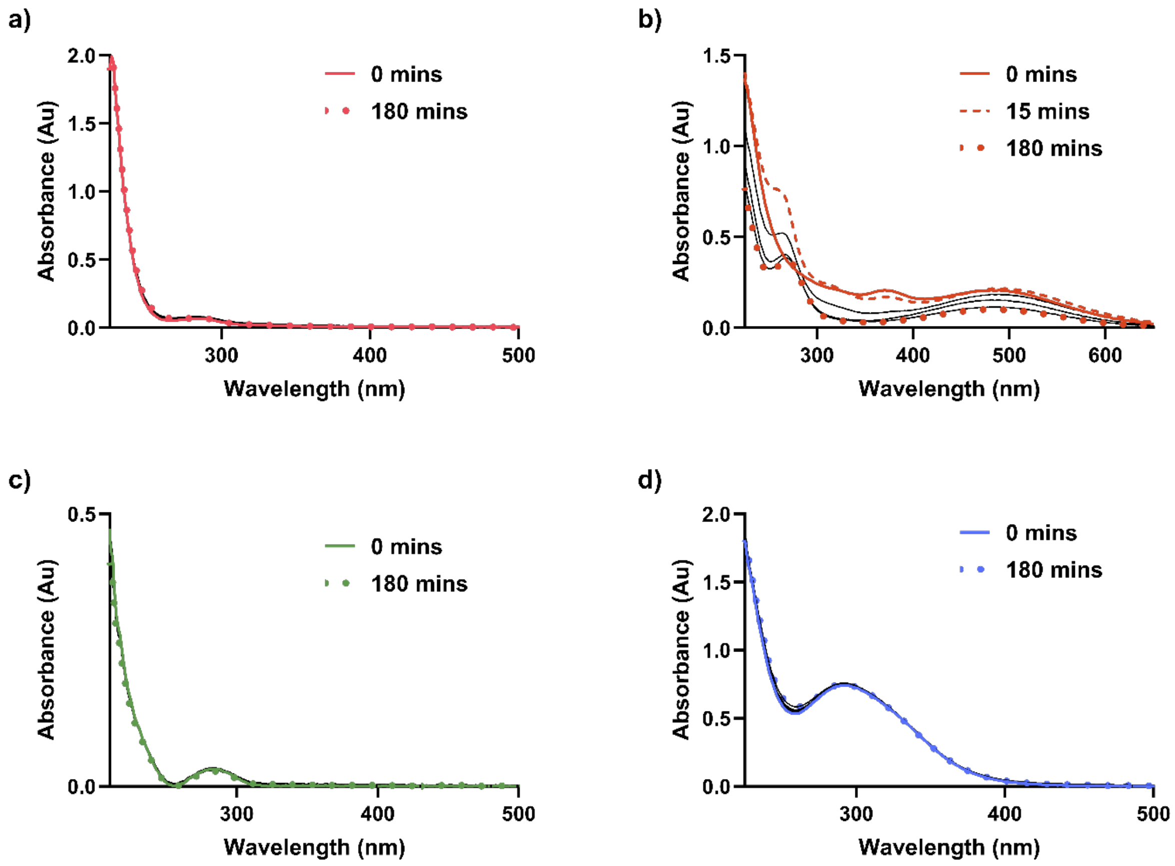

Figure S52. UV-vis spectra monitoring 420 nm photocleavage of **a)** AB, **b)** $[Fe(AB)]^{3-}$, **c)** $[Ga(AB)]^{3-}$, and **d)** $[Ti(AB)]^{2-}$. Starting material shown as solid line, photoproduct shown as dashed line, and degradation product shown with dotted line.

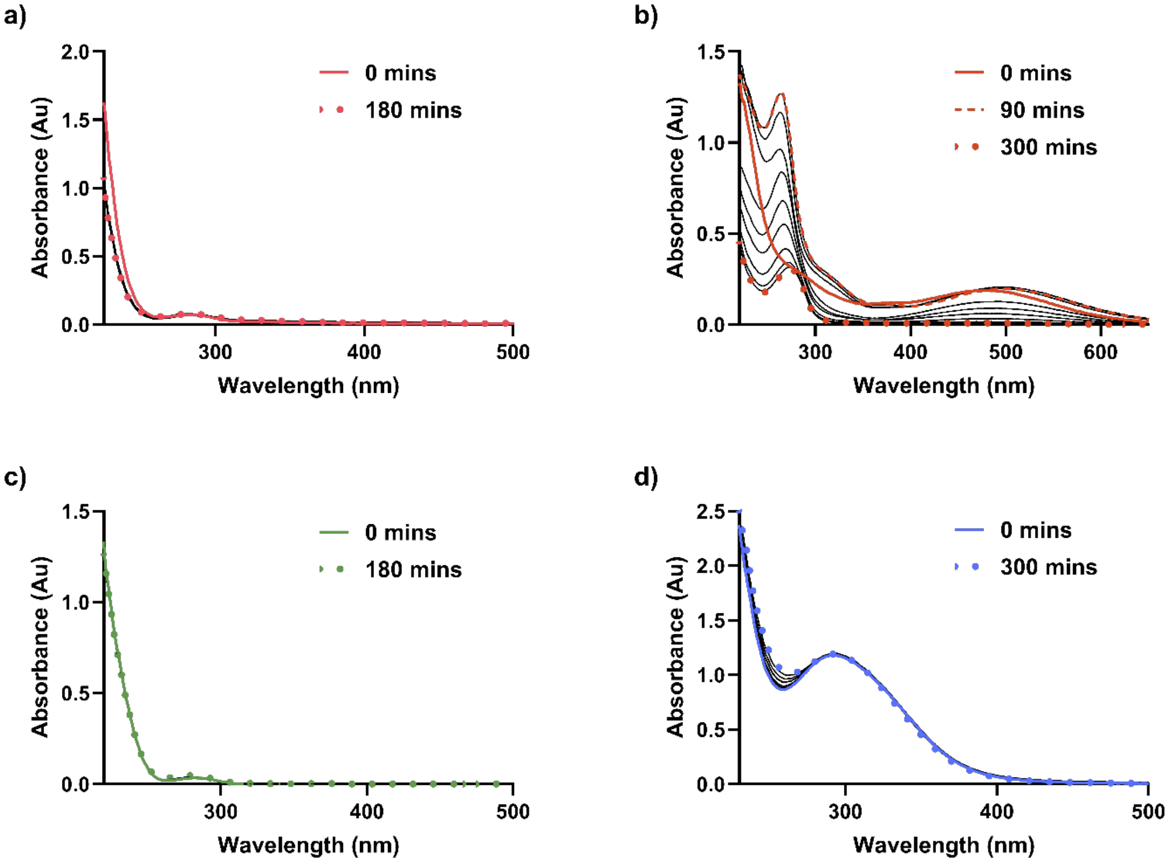

Figure S53. UV-vis spectra monitoring 575 nm photocleavage of **a)** AB, **b)** $[Fe(AB)]^{3-}$, **c)** $[Ga(AB)]^{3-}$, and **d)** $[Ti(AB)]^{2-}$. Starting material shown as solid line, photoproduct shown as dashed line, and degradation product shown with dotted line.

Table S4. Wavelength dependent photochemical degradation rates in (1/min).

Complex	254 nm			300 nm		
AB	0.027830	0.026930	0.024990	0.006586	0.004667	0.005494
Fe^{3+}	0.090310	0.081760	0.059700	0.053360	0.037240	0.055670
Ga^{3+}	0.007739	0.010000	0.009319	0.003386	0.000004	0.003357
Ti^{4+}	0.134710	0.134700	0.124300	0.007189	0.008113	0.008098

Table S5. Long wavelength dependent photochemical degradation rates in (1/min).

Complex	350 nm		420 nm			575 nm		
Fe^{3+}	0.012490	0.011420	0.011220	0.023450	0.013570	0.015450	0.015710	0.022060

Table S6. Wavelength dependent average photochemical degradation rates and standard deviations in (1/min).

Complex	254 nm		300 nm	
	Mean	Standard Deviation	Mean	Standard Deviation
**AB**	0.026583	0.001185055	0.00558	0.000785914
**Fe^{3+}**	0.077257	0.012895814	0.04876	0.008197936
**Ga^{3+}**	0.009019	0.000947059	0.00225	0.001587314
**Ti^{4+}**	0.131237	0.004904966	0.00780	0.000432086

Table S7. Long wavelength dependent average photochemical degradation rates and standard deviations in (1/min).

Wavelength	Mean	Standard Deviation
**254 nm**	0.0772567	0.0128958
**300 nm**	0.0487567	0.0081979
**350 nm**	0.0119550	0.0005350
**420 nm**	0.0160800	0.0052989
**575 nm**	0.0177400	0.0030565

Density Functional Theory Calculations

Table S8. Determination of the most favorable isomer of aerobactin and photoproduct complexes through single point energies.

	Single Point Energies (kcal/mol)			
**Complex**	***cis-cis C-fac***	***cis-cis N-fac***	***cis-trans C-mer***	***cis-trans N-mer***
AB	-1290705.39	-1290657.01	-1290718.25	-1290705.45
Ti^{IV}-AB	-1821664.60	n/a	-1821904.12	-1821655.03
Fe^{III}-AB	-2081660.15	n/a	-2081899.76	-2081661.65
Ga^{III}-AB	-2496579.26	n/a	-2496774.74	-2496569.41
AB*	-1171614.37	-1171619.80	-1171620.02	-1171627.61
Ti^{IV}-AB*	-1703081.2	-1703076.6	-1703081.5	-1703076.7
Fe^{III}-AB*	-1962852.5	-1963084.2	-1963088.2	-1963084.6
Ga^{III}-AB*	-2377976.9	-2377939.1	-2377973.8	-2377972.3

Table S9. Determination of the most favorable isomer of aerobactin (AB) and photoproduct (AB*) complexes with relative energies.

	Relative Energies (kcal/mol)			
**Complex**	***cis-cis C-fac***	***cis-cis N-fac***	***cis-trans C-mer***	***cis-trans N-mer***
AB	12.86	61.24	**0**	12.8
Ti^{4+}-AB	239.52	n/a	**0**	249.09
Fe^{3+}-AB	239.62	n/a	**0**	238.11
Ga^{3+}-AB	195.48	n/a	**0**	205.33
AB*	13.24	7.81	7.59	**0**
Ti^{4+}-AB*	0.35	4.93	**0**	4.86
Fe^{3+}-AB*	235.61	3.92	**0**	3.53
Ga^{3+}-AB*	**0**	37.86	3.13	4.65

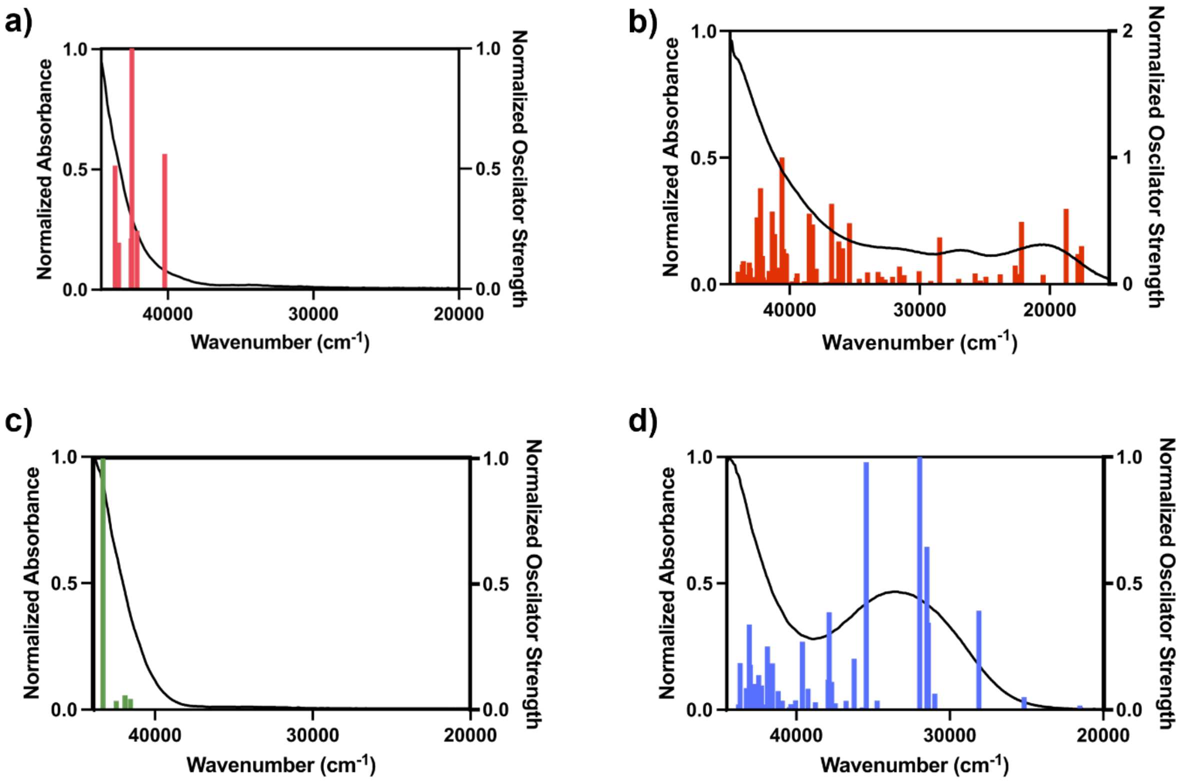

Figure S54. Overlay of experimental UV-vis spectra with the computational spectra of the excited state transitions of the most favorable isomer **a)** AB **b)** $[Fe(AB)]^{3-}$ **c)** $[Ga(AB)]^{3-}$ **d)** $[Ti(AB)]^{2-}$. All computational spectra have been red shifted 6000 cm^{-1} to align more closely with the experimental data.

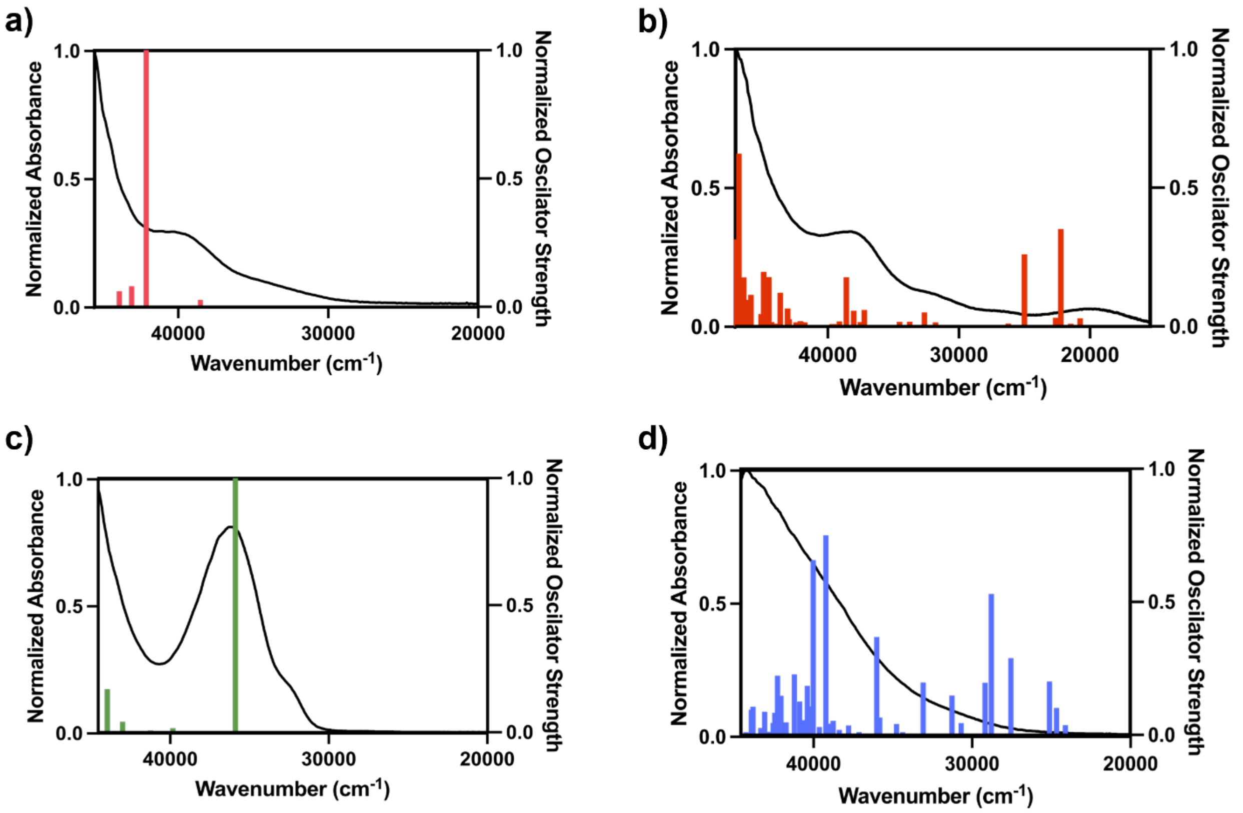

Figure S55. Overlay of experimental UV-vis spectra with the computational spectra of the excited state transitions of the most favorable isomer **a)** AB* **b)** $[Fe(AB^*)]^{3-}$ **c)** $[Ga(AB^*)]^{3-}$ **d)** $[Ti(AB^*)]^{2-}$. All computational spectra have been red shifted 6000 cm^{-1} to align more closely with the experimental data.

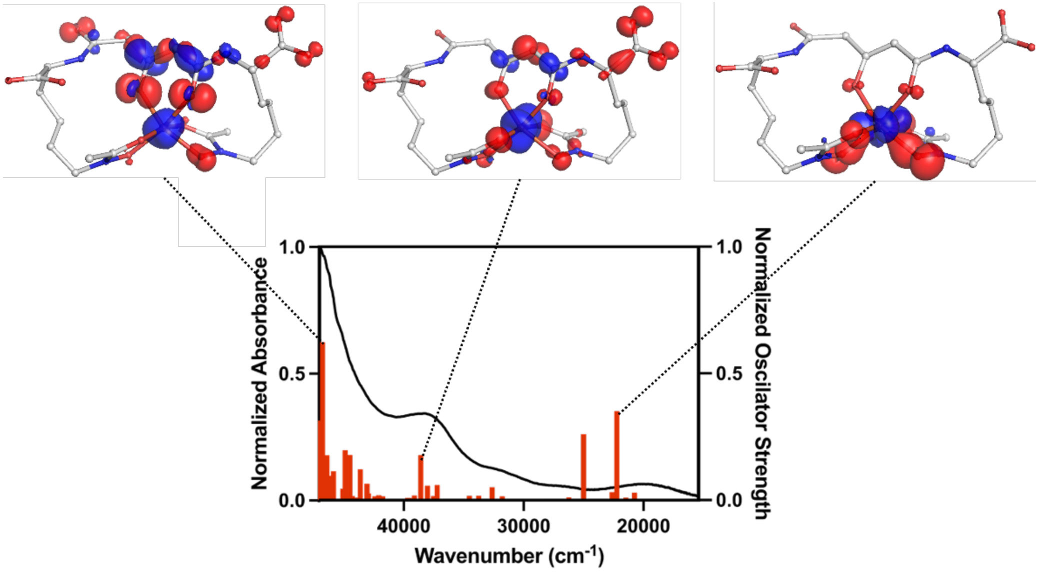

Figure S56. Overlay of experimental **[Fe(AB*)]$^{2-}$** UV-vis spectrum with the computational spectrum of the most favorable isomer. EDDMs depict electronic transitions with electron density gain shown in blue and electron density loss indicated in red. All computational spectra have been red shifted 6000 cm^{-1} to align more closely with the experimental data.

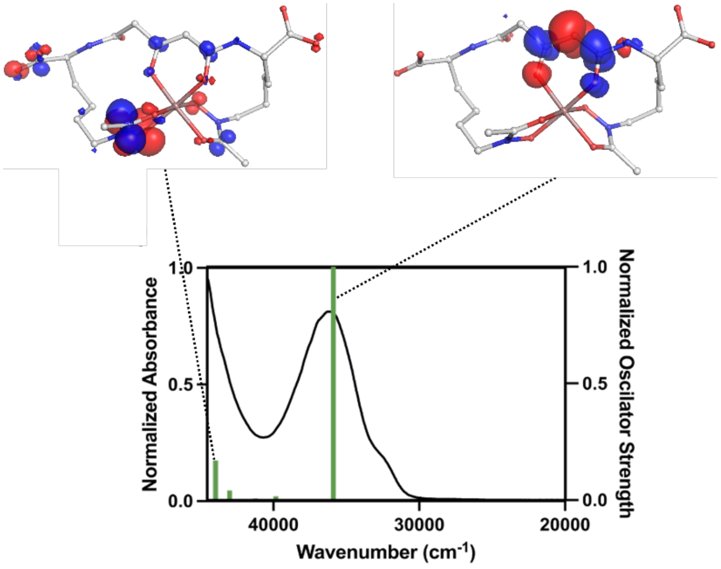

Figure S57. Overlay of experimental **[Ga(AB*)]$^{2-}$** UV-vis spectrum with the computational spectrum of the most favorable isomer. EDDMs depict electronic transitions with electron density gain shown in blue and electron density loss indicated in red. All computational spectra have been red shifted 6000 cm^{-1} to align more closely with the experimental data.

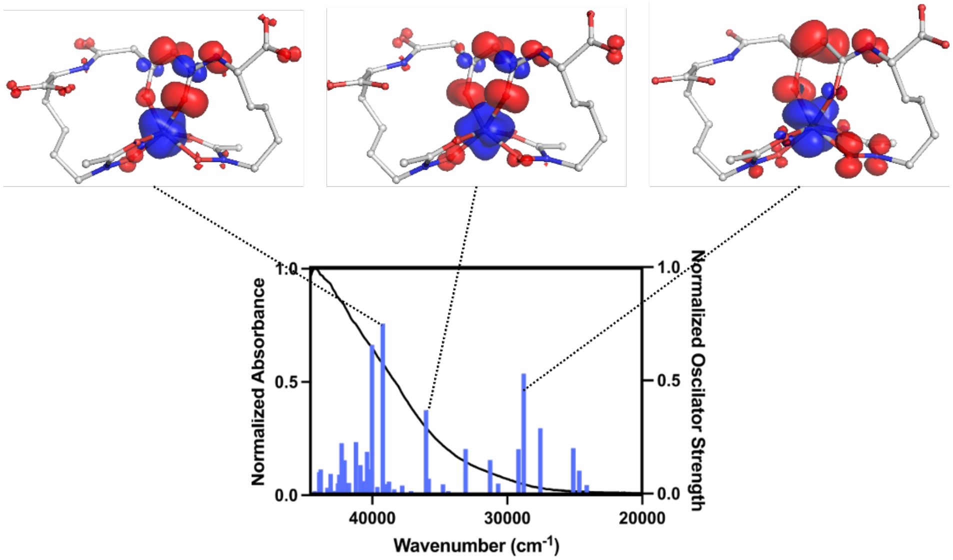

Figure S58. Overlay of experimental **[Ti(AB*)]$^{1-}$** UV-vis spectrum with the computational spectrum of the most favorable isomer. EDDMs depict electronic transitions with electron density gain shown in blue and electron density loss indicated in red. All computational spectra have been red shifted 6000 cm^{-1} to align more closely with the experimental data.

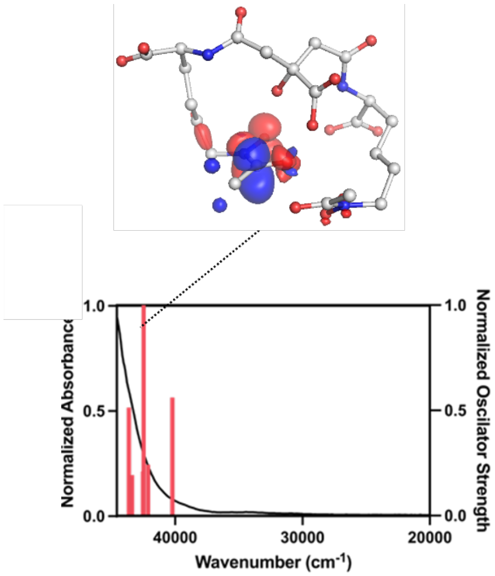

Figure S59. Overlay of experimental **AB** UV-vis spectrum with the computational spectrum of the most favorable isomer. EDDMs depict electronic transitions with electron density gain shown in blue and electron density loss indicated in red. All computational spectra have been red shifted 6000 cm^{-1} to align more closely with the experimental data.

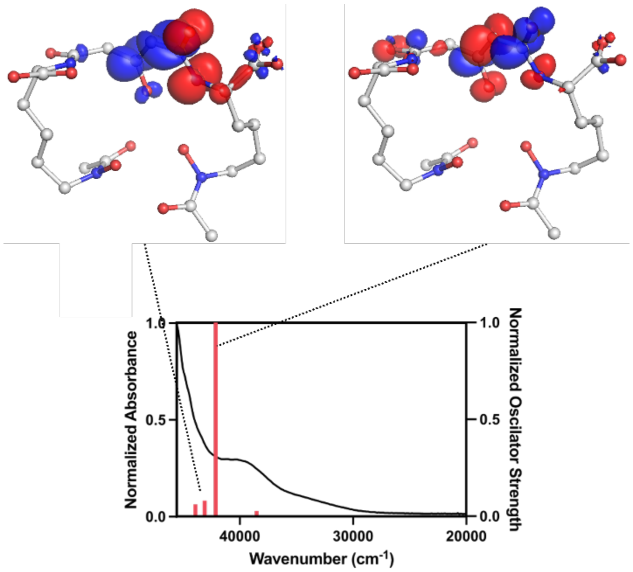

Figure S60. Overlay of experimental **AB*** UV-vis spectrum with the computational spectrum of the most favorable isomer. EDDMs depict electronic transitions with electron density gain shown in blue and electron density loss indicated in red. All computational spectra have been red shifted 6000 cm^{-1} to align more closely with the experimental data.

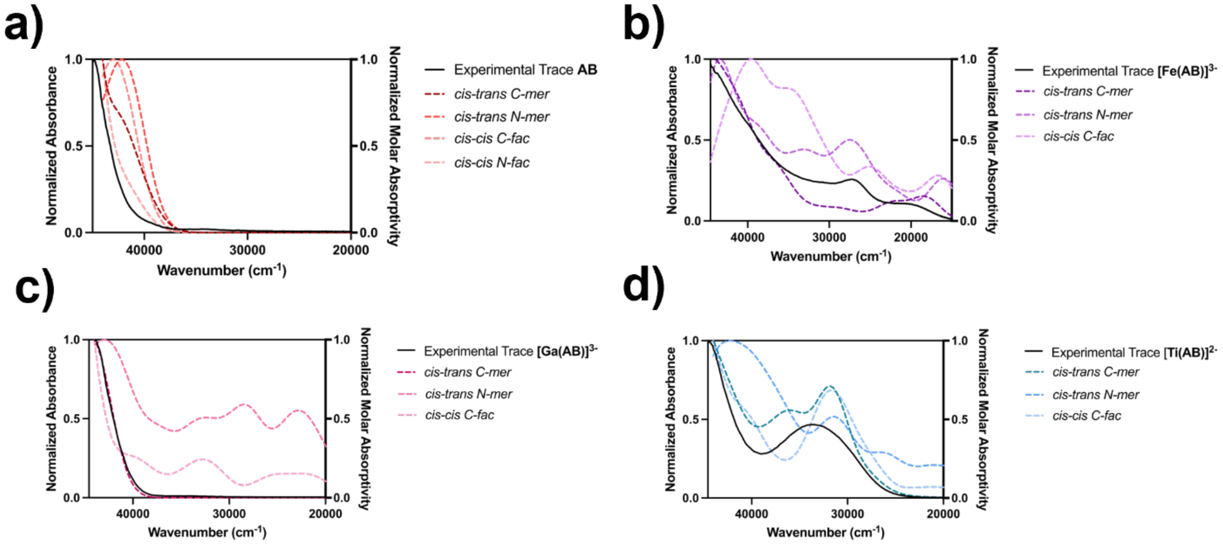

Figure S61. Overlay of experimental UV-vis spectra with the computational spectra of all optimized isomers. All computational spectra have been red shifted 6000 cm^{-1} to align more closely with the experimental data. **a)** AB, **b)** $[Fe(AB)]^{3-}$, **c)** $[Ga(AB)]^{3-}$, **d)** $[Ti(AB)]^{2-}$.

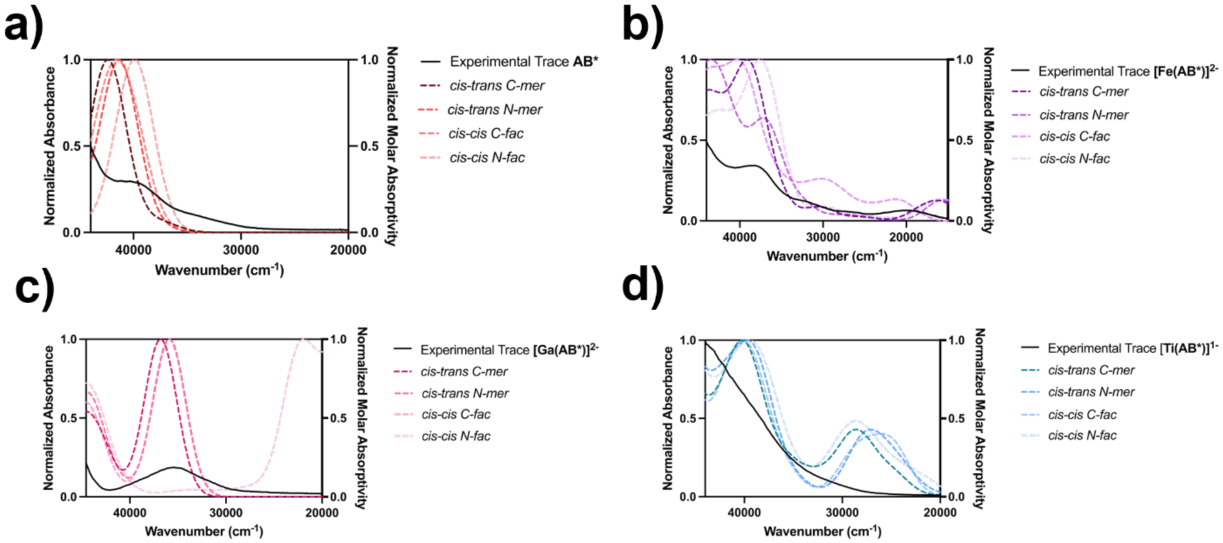

Figure S62. Overlay of experimental UV-vis spectra with the computational spectra of all optimized isomers. All computational spectra have been red shifted 6000 cm^{-1} to align more closely with the experimental data. **a)** AB*, **b)** $[Fe(AB^*)]^{2-}$, **c)** $[Ga(AB^*)]^{2-}$, **d)** $[Ti(AB^*)]^{1-}$.

Geometry Optimized Structures of [M(*CCF*-AB)]$^{n-4}$ Complexes

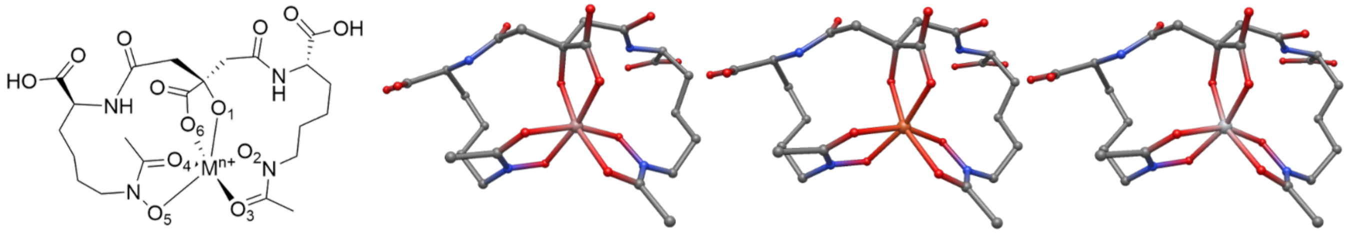

M^{n+} = Ga^{3+}, Fe^{3+}, or Ti^{4+}

Figure S63. Chemical structure of **[M(*CCF*-AB)]$^{n-4}$** with coordinating atoms annotated (left), with DFT-optimized configurational molecular model structures of [Ga(*CCF*-AB)]$^{1-}$, [Fe(*CCF*-AB)]$^{1-}$, or [Ti(*CCF*-AB)] respectively (right).

Table S10. Optimized metal-center based bonding of **[M(*CCF*-AB)]$^{n-4}$** structures.

**Complex**	**[Ga(*CCF*-AB)]$^{1-}$**	**[Fe(*CCF*-AB)]$^{1-}$**	**[Ti(*CCF*-AB)]**
M-O_1	1.896	1.905	1.825
M-O_2	1.943	1.961	1.898
M-O_3	2.050	2.130	2.067
M-O_4	2.030	2.100	2.035
M-O_5	1.976	2.002	1.943
M-O_6	1.998	2.057	1.983
O_1-O_6	83.171	80.436	79.766
O_2-O_3	78.996	76.234	75.609
O_4-O_5	79.169	76.447	75.639
O_1-O_3	167.031	161.756	159.165
O_2-O_4	162.700	158.939	156.515
O_4-O_6	166.933	162.954	160.610

Geometry Optimized Structures of [M(*TCM*-AB)]$^{n-4}$ Complexes

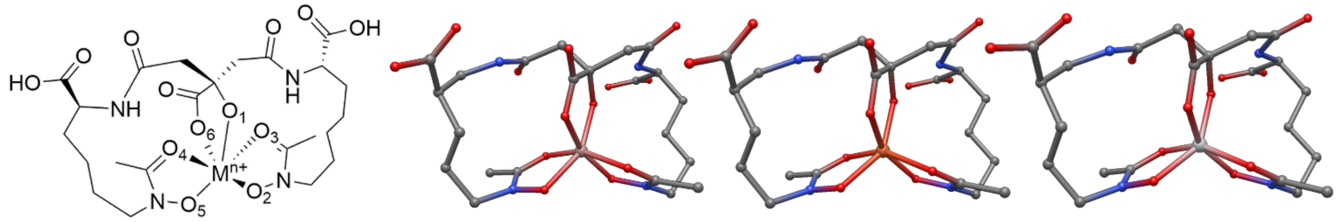

M^{n+} = Ga^{3+}, Fe^{3+}, or Ti^{4+}

Figure S64. Chemical structure of **[M(*TCM*-AB)]$^{n-4}$** with coordinating atoms annotated (left), with DFT-optimized configurational molecular model structures of [Ga(*CCF*-AB)]$^{1-}$, [Fe(*CCF*-AB)]$^{1-}$, or [Ti(*CCF*-AB)] respectively (right).

Table S11. Optimized metal-center based bonding of **[M(*TCM*-AB)]$^{n-4}$** structures.

**Complex**	**[Ga(*TCM*-AB)]$^{1-}$**	**[Fe(*TCM*-AB)]$^{1-}$**	**[Ti(*TCM*-AB)]**
M-O_1	1.893	1.916	1.836
M-O_2	1.978	2.011	1.949
M-O_3	2.034	2.087	2.021
M-O_4	2.068	2.100	2.017
M-O_5	1.954	1.944	1.947
M-O_6	2.038	2.082	1.996
O_1-O_6	81.029	78.757	78.794
O_2-O_3	78.003	75.502	74.911
O_4-O_5	78.005	75.926	75.297
O_1-O_5	152.877	145.900	143.300
O_2-O_6	159.473	154.548	153.198
O_3-O_4	155.913	151.951	151.618

Geometry Optimized Structures of [M(*TNM*-AB)]$^{n-4}$ Complexes

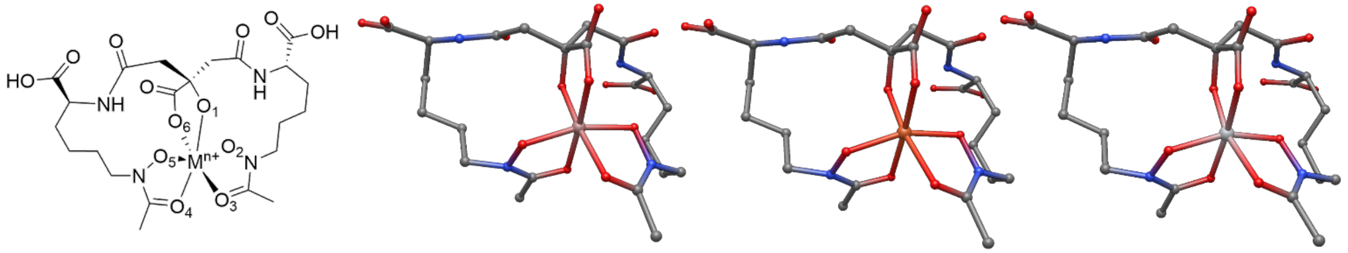

M^{n+} = Ga^{3+}, Fe^{3+}, or Ti^{4+}

Figure S65. Chemical structure of **[M(*TNM*-AB)]$^{n-4}$** with coordinating atoms annotated (left), with DFT-optimized configurational molecular model structures of [Ga(*CCF*-AB)]$^{1-}$, [Fe(*CCF*-AB)]$^{1-}$, or [Ti(*CCF*-AB)] respectively (right).

Table S12. Optimized metal-center based bonding of **[M(*TNM*-AB)]$^{n-4}$** structures.

**Complex**	**[Ga(*TNM*-AB)]$^{1-}$**	**[Fe(*TNM*-AB)]$^{1-}$**	**[Ti(*TNM*-AB)]**
M-O_1	1.899	1.903	1.820
M-O_2	2.080	2.173	2.085
M-O_3	1.948	1.961	1.901
M-O_4	1.966	2.108	2.059
M-O_5	1.966	2.006	1.938
M-O_6	1.971	2.023	1.959
O_1-O_6	84.655	81.754	80.566
O_2-O_3	77.405	75.053	75.518
O_4-O_5	79.503	76.566	75.450
O_1-O_2	174.626	171.764	165.223
O_3-O_5	163.729	159.932	158.457
O_4-O_6	177.218	176.798	173.581

Geometry Optimized Structures of [M(*CCF*-AB*)]$^{n-3}$ Complexes

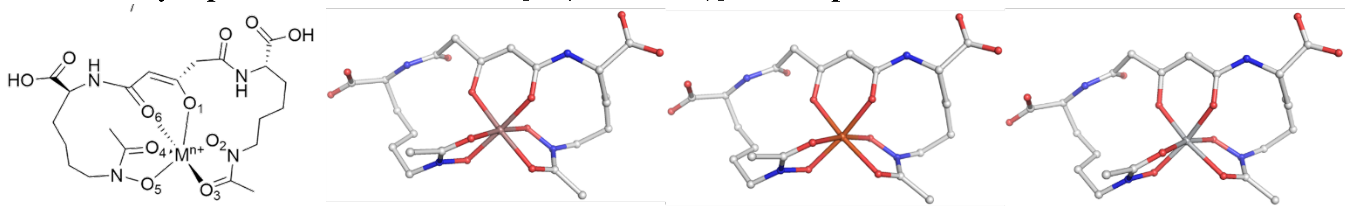

M^{n+} = Ga^{3+}, Fe^{3+}, or Ti^{4+}

Figure S66. Chemical structure of **[M(*CCF*-AB*)]$^{n-3}$** with coordinating atoms annotated (left), with DFT-optimized configurational molecular model structures of [Ga(*CCF*-AB*)], [Fe(*CCF*-AB*)], or [Ti(*CCF*-AB*)]$^{1+}$, respectively (right).

Table S13. Optimized metal-center based bonding of **[M(*CCF*-AB)]$^{n-3}$** structures.

**Complex**	**[Ga(*CCF*-AB*)]**	**[Fe(*CCF*-AB*)]**	**[Ti(*CCF*-AB*)]$^{1+}$**
M-O_1	1.953	1.982	1.885
M-O_2	1.948	1.966	1.898
M-O_3	2.008	2.097	2.042
M-O_4	1.996	2.097	2.023
M-O_5	1.954	1.964	1.898
M-O_6	1.983	2.054	1.973
O_1-O_6	89.288	86.347	85.379
O_2-O_3	80.762	77.343	76.082
O_4-O_5	80.482	77.225	76.612
O_1-O_3	175.895	172.748	170.480
O_2-O_4	172.860	170.970	165.798
O_5-O_6	172.909	168.155	164.192

Geometry Optimized Structures of [M(*CNF*-AB*)]$^{n-3}$ Complexes

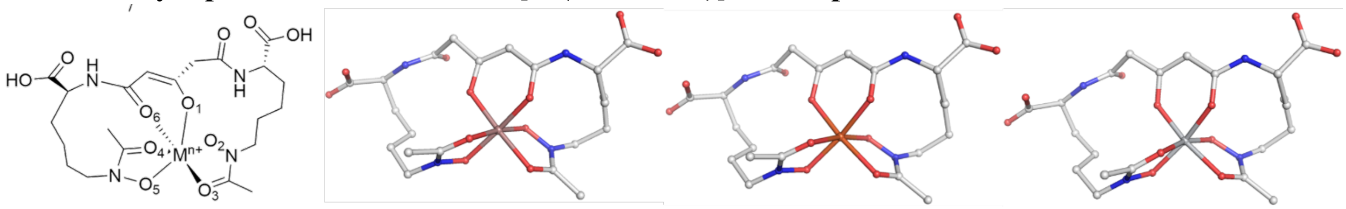

M^{n+} = Ga^{3+}, Fe^{3+}, or Ti^{4+}

Figure S67. Chemical structure of **[M(*CNF*-AB*)]$^{n-3}$** with coordinating atoms annotated (left), with DFT-optimized configurational molecular model structures of [Ga(*CCF*-AB*)], [Fe(*CCF*-AB*)], or [Ti(*CCF*-AB*)]$^{1+}$, respectively (right).

Table S14. Optimized metal-center based bonding of **[M(*CNF*-AB*)]$^{n-3}$** structures.

**Complex**	**[Ga(*CNF*-AB*)]**	**[Fe(*CNF*-AB*)]**	**[Ti(*CNF*-AB*)]$^{1+}$**
M-O_1	1.999	2.015	1.917
M-O_2	1.936	1.979	1.921
M-O_3	1.984	2.108	2.007
M-O_4	1.960	2.072	2.008
M-O_5	1.920	1.965	1.901
M-O_6	2.047	2.018	1.943
O_1-O_6	85.420	85.423	84.168
O_2-O_3	80.328	76.364	76.512
O_4-O_5	81.964	77.742	76.503
O_1-O_2	163.528	166.105	164.767
O_3-O_5	176.289	164.791	160.627
O_4-O_6	169.885	173.466	171.970

Geometry Optimized Structures of [M(*TCM*-AB*)]$^{n-3}$ Complexes

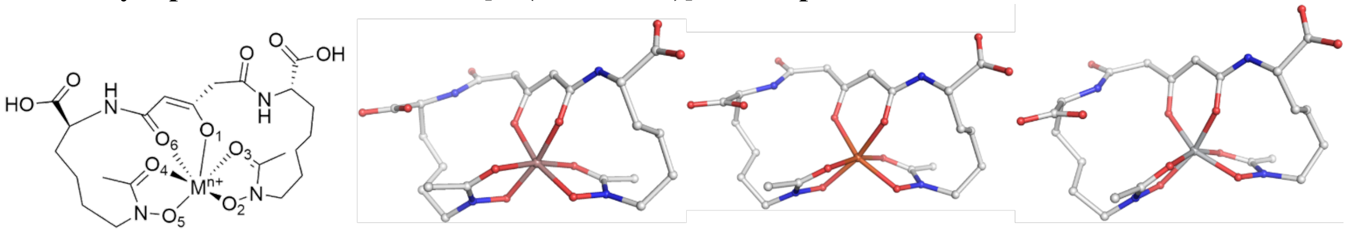

M^{n+} = Ga^{3+}, Fe^{3+}, or Ti^{4+}

Figure S68. Chemical structure of **[M(*TCM*-AB*)]$^{n-3}$** with coordinating atoms annotated (left), with DFT-optimized configurational molecular model structures of [Ga(*CCF*-AB*)], [Fe(*CCF*-AB*)], or [Ti(*CCF*-AB*)]$^{1+}$, respectively (right).

Table S15. Optimized metal-center based bonding of **[M(*TCM*-AB)]$^{n-3}$** structures.

**Complex**	**[Ga(*TCM*-AB*)]**	**[Fe(*TCM*-AB*)]**	**[Ti(*TCM*-AB*)]$^{1+}$**
M-O_1	1.954	1.990	1.896
M-O_2	1.961	1.988	1.935
M-O_3	2.004	2.089	2.006
M-O_4	1.979	2.052	1.990
M-O_5	1.960	1.987	1.917
M-O_6	1.985	2.055	1.961
O_1-O_6	86.519	84.069	83.408
O_2-O_3	79.705	76.416	75.694
O_4-O_5	80.398	77.199	76.058
O_1-O_2	164.122	157.201	154.613
O_3-O_4	169.908	167.626	161.306
O_5-O_6	168.080	162.747	157.956

Geometry Optimized Structures of [M(*TNM*-AB*)]$^{n-3}$ Complexes

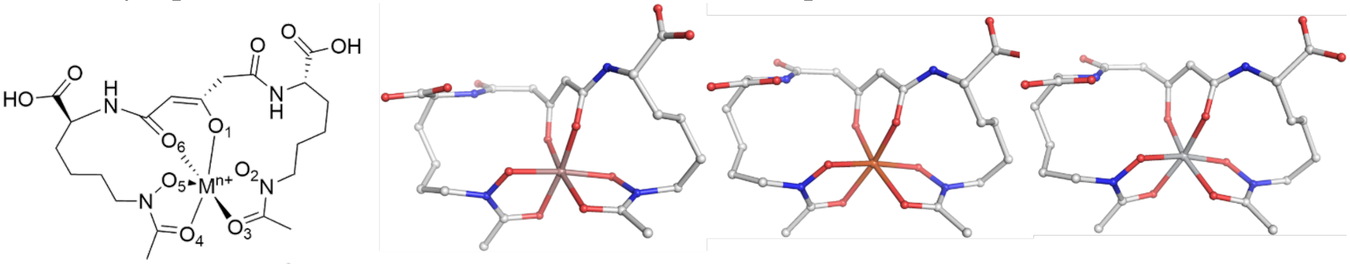

M^{n+} = Ga^{3+}, Fe^{3+}, Sc^{3+}, Ti^{4+}, or Zr^{4+}

Figure S69. Chemical structure of **[M(*TNM*-AB*)]$^{n-3}$** with coordinating atoms annotated (left), with DFT-optimized configurational molecular model structures of [Ga(*CCF*-AB*)], [Fe(*CCF*-AB*)], or [Ti(*CCF*-AB*)]$^{1+}$, respectively (right).

Table S16. Optimized metal-center based bonding of **[M(*TNM*-AB*)]$^{n-3}$** structures.

**Complex**	**[Ga(*TNM*-AB*)]**	**[Fe(*TNM*-AB*)]**	**[Ti(*TNM*-AB*)]$^{1+}$**
M-O$_1$	1.948	1.981	1.876
M-O$_2$	1.944	1.981	1.923
M-O$_3$	2.021	2.108	2.036
M-O$_4$	2.006	1.968	1.990
M-O$_5$	1.931	2.076	1.921
M-O$_6$	1.979	2.033	1.957
O$_1$-O$_6$	92.100	89.255	86.835
O$_2$-O$_3$	80.087	76.711	75.909
O$_4$-O$_5$	80.585	77.287	76.492
O$_1$-O$_3$	171.447	167.893	167.326
O$_2$-O$_5$	173.834	172.800	169.171
O$_4$-O$_6$	171.048	166.941	165.741

Coordinates for Geometry Optimized Compounds

***cis-cis-C-fac* AB**

O	0.82134490287484	2.31466496863325	1.45096609254463
N	-2.08991357459973	2.17244336638226	-0.72767369882320
C	-4.30985806279997	-1.64497033884973	0.63907485185496
H	-4.76432660181845	-1.35705616537448	1.58934970751332
N	-3.05914761259668	-2.31452265926094	0.90106509193693
H	-2.83285000664143	-3.10976767871766	0.32422095084601
C	-5.23726195789895	-2.63204418080037	-0.03853140714151
O	-6.50373076631071	-2.24536537310534	-0.18034026114045
C	-2.14818482552951	-1.82183062737544	1.74734282433067
O	-2.35946470044876	-0.83723573183806	2.44927798797361
C	-2.54899544200264	3.42777491693953	-0.56413972892644
C	-3.12769176366069	4.09025909416661	-1.78581102144360
H	-2.32715652444129	4.36289787454664	-2.47536031726017
H	-3.82454614169894	3.44720410658581	-2.31817697604893
H	-3.63851751022014	4.99374819348294	-1.46695222420428
C	-0.81457398091990	-2.52595248144352	1.77230848505742
H	-0.65697027324694	-3.10876795464566	0.86855166857519
H	-0.79499528080732	-3.20385329488989	2.62564458643638
C	0.32421441757483	-1.51850927233115	1.91207450136805
C	0.31583795327838	-0.89761760771407	3.31843058879139
C	1.69284353702312	-2.20511268231209	1.76332851544010
H	1.68694117759858	-3.14672431141602	2.30951108469509
H	2.46448155143482	-1.57023712046281	2.19677018914886
C	2.01342066898119	-2.48442043343205	0.31035768615743
O	1.34324689865508	-3.24089130903164	-0.38228862812256
N	3.09029173213519	-1.84102412473214	-0.16099695533558
H	3.64641014450366	-1.27404908682762	0.45868222146714
C	5.05275436936435	-1.71581375692769	-1.52256936135037
O	5.69512246418674	-1.98877752880782	-2.65629143605002
N	1.98182164776726	2.69921534133996	-0.43423173567278
C	1.90709806562512	2.34954865117117	0.85297441595928
C	3.17483226985411	2.00006180752211	1.56734130952753
H	3.11960487516156	2.42111829102425	2.56848919489247
H	3.22628927702705	0.91478680423716	1.66292047816387
H	4.07318799391098	2.35156590716165	1.07139567452483
C	3.12752451549161	2.59618027733848	-1.33805896735150
H	2.81671663194483	3.11847736625260	-2.23932587908641
H	3.96209235549452	3.15085043522283	-0.91343537708525
C	3.53921667218645	1.16614124960759	-1.66181198307404
H	4.27844673211295	1.21884223145341	-2.46533650638609
H	4.05211713625529	0.74387874110441	-0.79845206980315
C	2.37664823931770	0.27258431144798	-2.06973611738945
H	1.67723885480462	0.18320557670088	-1.23722652687752
H	1.82460941651911	0.74463943948122	-2.88563936075734
C	2.77216717633978	-1.12390745700708	-2.53429228030203
H	1.86429313820497	-1.67416574840212	-2.77749446739344
H	3.34876796038004	-1.05850813935950	-3.45897265464506
C	3.55788961207812	-1.98180437806335	-1.51573114458432
H	3.43561510359496	-3.02690982630357	-1.81172925751500
C	-3.16637739402255	0.59455073275543	-2.38594274207675
H	-4.06048011239759	1.22133500704687	-2.42226903711928
H	-2.97356863867330	0.27924630218628	-3.41374945114764
C	-1.96309690093208	1.43615625565245	-1.97642918970951
H	-1.73935688340407	2.15082349624673	-2.76658420486485

H	-1.08221076535786	0.80650506062475	-1.86271641437669
C	-3.43834284287824	-0.64987787696306	-1.54767922110094
H	-2.49618913100750	-1.17803016299157	-1.38289280225265
H	-4.06963149968027	-1.32218813074289	-2.13440347462140
C	-4.11382976375177	-0.37624232380431	-0.21074552878587
H	-3.52189924802171	0.30904987469053	0.39037050649625
H	-5.08297955294233	0.10087096512962	-0.37238080807392
O	-4.88636629007796	-3.70232579109692	-0.45866141946974
O	5.66500453492983	-1.29396512230244	-0.57784494279816
O	0.28031306056535	-0.52484443240919	0.91414872901306
O	0.51689282465924	0.40400059094612	3.41202593325413
O	-1.53009543868092	1.56750886990498	0.37950311313848
O	-2.48027787660128	4.00931151708562	0.51441786597281
O	0.77277345303313	2.90372397021971	-1.07457771860674
O	0.21618993850471	-1.57794850836170	4.30937997481076
H	-0.59381751232170	-0.10766086435434	0.84722299362492
H	0.61191862567122	3.85967543663518	-1.04506436597771
H	-6.67380369979090	-1.38173886734827	0.22434079982626
H	0.60339088285188	0.87394728223979	2.54899712024099
H	5.11543998258425	-2.36898518586851	-3.33264826302157
H	-0.68312921829736	2.03751422250816	0.58318178419128

***cis-cis-N-fac* AB**

H	-0.70377800000000	2.31360100000000	-3.54531100000000
N	-1.94054100000000	2.10353100000000	-0.56103300000000
C	-4.45697300000000	-1.68770800000000	0.53489200000000
H	-4.89136500000000	-1.93290900000000	1.50453600000000
N	-3.07959000000000	-2.13637000000000	0.54132800000000
H	-2.79653900000000	-2.66095200000000	-0.27282000000000
C	-5.25033700000000	-2.46185700000000	-0.53713600000000
O	-6.48552700000000	-2.27621800000000	-0.53627000000000
C	-2.32014700000000	-2.13415500000000	1.64201400000000
O	-2.70947900000000	-1.64250400000000	2.70297400000000
C	-1.21428500000000	1.73042000000000	-1.59374100000000
C	-1.61192000000000	2.10482500000000	-2.98388800000000
H	-2.11804900000000	1.26449800000000	-3.46016900000000
H	-2.26472900000000	2.97237500000000	-3.01747100000000
C	-0.96031500000000	-2.78555700000000	1.53557200000000
H	-0.87620900000000	-3.37261900000000	0.62242000000000
H	-0.86215700000000	-3.45505400000000	2.38976700000000
C	0.20823600000000	-1.78041500000000	1.54661800000000
C	0.12570600000000	-0.90231100000000	2.82124900000000
C	1.55889300000000	-2.55101600000000	1.55824600000000
H	1.46324600000000	-3.48428100000000	2.10923400000000
H	2.31031500000000	-1.93702600000000	2.04828200000000
C	1.98669100000000	-2.85542800000000	0.14051600000000
O	1.50224100000000	-3.79100800000000	-0.49828200000000
N	2.90904300000000	-2.02144000000000	-0.35810500000000
H	3.20459200000000	-1.27861600000000	0.25178300000000
C	4.78209200000000	-2.32381500000000	-1.99560800000000
O	5.08721200000000	-2.50916800000000	-3.19625900000000
N	2.79677600000000	2.32844000000000	0.44827500000000
C	3.23185300000000	1.34876000000000	1.24410500000000
C	4.67419500000000	1.26206200000000	1.63899800000000
C	3.52061200000000	2.84293500000000	-0.70775900000000
C	3.28831800000000	1.95639700000000	-1.93378400000000
C	3.87164500000000	0.54669000000000	-1.78244300000000
H	4.85936200000000	0.49724700000000	-2.24435300000000
H	4.04710400000000	0.35226400000000	-0.72640100000000
C	2.98580000000000	-0.57047400000000	-2.33245500000000
H	1.94243700000000	-0.34632800000000	-2.10744700000000
H	3.07831700000000	-0.62088600000000	-3.41767000000000
C	3.30306900000000	-1.95741000000000	-1.75085900000000
H	2.69886000000000	-2.69667900000000	-2.27166400000000
C	-4.31564700000000	1.77665400000000	-1.31462600000000
C	-3.29420400000000	2.64610000000000	-0.57954600000000
C	-4.20982400000000	0.27592800000000	-1.07304100000000
H	-3.19854300000000	-0.06561600000000	-1.30618000000000
H	-4.86826200000000	-0.21996900000000	-1.78919600000000
C	-4.58126000000000	-0.17356000000000	0.33084500000000
H	-3.94858500000000	0.32017700000000	1.06978500000000
H	-5.61273300000000	0.11311300000000	0.54314700000000
O	-4.60422400000000	-3.19245600000000	-1.32096800000000
O	5.55504100000000	-2.38219400000000	-1.01677400000000
O	0.13746600000000	-0.97678300000000	0.41870900000000
O	-0.12378300000000	0.33663100000000	2.60882200000000
O	-1.48221600000000	1.74691900000000	0.67302700000000
O	-0.16805800000000	1.06254700000000	-1.39547600000000

O	1.44294600000000	2.39934200000000	0.30260200000000
O	0.31003100000000	-1.40073300000000	3.92893300000000
O	2.40593200000000	0.54051600000000	1.70542200000000
H	5.28626400000000	2.06631300000000	1.24586000000000
H	4.57777200000000	2.90076300000000	-0.46139400000000
H	3.16095700000000	3.85420000000000	-0.89055500000000
H	2.21223800000000	1.89644100000000	-2.09528100000000
H	3.71538500000000	2.46198900000000	-2.80132000000000
H	-5.30590500000000	2.13107000000000	-1.01718600000000
H	-4.23876200000000	1.96133500000000	-2.38568600000000
H	-3.28012400000000	3.64809500000000	-1.01020400000000
H	-3.55305400000000	2.74660900000000	0.47014000000000
H	5.08466500000000	0.30413400000000	1.32054600000000
H	4.71103600000000	1.29011200000000	2.72802500000000
H	4.37794700000000	-2.21010900000000	-3.75304100000000
H	-6.70229800000000	-1.59077000000000	0.08475500000000
H	1.02215300000000	2.05279700000000	1.08063800000000
H	0.18838000000000	-1.51867900000000	-0.35991600000000
H	-0.08179900000000	0.51274000000000	1.67623200000000
H	-0.59903900000000	1.40543300000000	0.59631400000000

***cis-trans-C-mer* AB**

H	2.73066592006541	2.13967008153768	-0.29592923561789
C	-1.54997546200625	3.59779270635289	1.64341484192220
C	-3.44777455181148	-1.36775391024541	-1.59136757308326
H	-4.02948022612936	-2.27750326731254	-1.74659567712542
N	-2.20913175124487	-1.73338493001902	-0.94943316637537
H	-1.37161445137561	-1.22198579805599	-1.18659401021663
C	-3.14195561452789	-0.76150983291989	-2.94266285217885
O	-4.17526881273297	-0.61134031580151	-3.76556733465976
C	-2.14450904780806	-2.73767437633212	-0.05956130754477
O	-3.11540633243134	-3.42882679398102	0.22185222834812
N	-2.34530523035875	3.43884330311438	0.57104358797041
C	-3.78684972710602	3.25760028672736	0.54956290602618
H	-4.14113991450463	3.80211735855436	-0.32535901718968
H	-4.20931680214856	3.74778437004683	1.42387678169350
C	-0.80188631860698	-2.99801474340831	0.59563453690498
H	-0.21176786078025	-3.64558361913703	-0.05580092074904
H	-1.00938213592076	-3.55877626897763	1.50199895800864
C	0.03656439925175	-1.76495266917989	0.93847046334264
C	-0.82905129358672	-0.74178131296924	1.68188790311691
C	1.19882615501912	-2.09879981891758	1.90090635251037
H	0.83175721428255	-2.74457879764280	2.69537187934526
H	1.55729974435812	-1.17391071937262	2.35112217264098
C	2.33300996519843	-2.79591761629263	1.18855228895771
O	2.25272201317331	-3.94866614312294	0.79843336276293
N	3.43468145630866	-2.03055807914216	1.00128211325640
H	3.33725876336221	-1.04600475857165	1.19960871809839
C	5.85169006405679	-1.95069035594476	0.65003522482128
O	6.80998131592146	-1.75363454889216	-0.04658180643356
C	1.04075643827950	1.74075535952647	-1.54691155301847
N	1.53728483113863	1.07728576062827	-2.57466119500265
C	2.86983888457686	1.09228056515078	-3.14497499927304
H	3.42243379150455	1.89431737395913	-2.66374687667999
H	2.76390887059619	1.34272407728466	-4.20085361672713
C	1.91875461335814	2.68060524735665	-0.78138195985642
H	1.30631783726870	3.16500549918144	-0.02558664942884
H	2.35617270678930	3.43342535561932	-1.43556560591763
C	3.58484969221097	-0.24010286375237	-2.97913070530491
H	2.96636477301407	-1.02700367185308	-3.41440467157656
H	4.50325428998985	-0.20174951002173	-3.56752241596173
C	3.89781595072286	-0.57105631428353	-1.52841732476060
H	4.70183612773770	0.07632833085156	-1.16775556945314
H	3.01811397625795	-0.34731980633493	-0.92276194368085
C	4.26555178377698	-2.03361497689963	-1.33829645384876
H	3.45190405142332	-2.65746134881526	-1.71323707966399
H	5.15478507081683	-2.28645516899241	-1.91525441242724
C	4.50958906701134	-2.43139574751115	0.11510751893289
H	4.56408235154926	-3.52133467701337	0.17000545063042
C	-4.25076064302470	1.80904171082093	0.49512816574580
H	-5.34318250619231	1.82187900120611	0.44577484164179
H	-3.98665305328329	1.30624834356340	1.42855334236661
C	-2.20891156657620	3.45422519532558	2.98964557899595
H	-1.42790888132326	3.43306760216342	3.74361684643078
H	-2.86711545598651	4.30191557048537	3.18398110630891
C	-3.68907671621991	1.01224165977408	-0.67004941717698
H	-2.60733628306508	0.94717909943543	-0.56635734192588

H	-3.88228966532702	1.54112607672937	-1.60746102204521
C	-4.27760638849113	-0.39046863153873	-0.73920137717061
H	-4.33340661610880	-0.82827601167847	0.25820868616901
H	-5.29959708470341	-0.35335641227100	-1.12136686603258
O	-2.05041784175108	-0.38428733355992	-3.28260405462013
O	5.94163403231542	-1.80634653976878	1.97136633154952
O	0.51801468512014	-1.11981583314987	-0.22678527446971
O	-0.84016608275145	0.49600018395035	1.23416445778945
O	-0.34930127576816	3.83459804806396	1.53443635353816
O	-1.78289833812111	3.63186952229161	-0.67811052268491
O	-0.16224478725988	1.55874843021431	-1.23917651664751
O	-1.44536536013607	-1.05478070830967	2.66869648143986
O	0.69140728151237	0.22352510219910	-3.25465342773051
H	-2.80106535178673	2.54386868080582	3.05908715035011
H	0.92555354152203	-1.75521369425635	-0.82865624226375
H	5.08794568580594	-1.99706943770705	2.39440257104080
H	-4.99332428601713	-0.98627952330254	-3.40640093935712
H	-0.38829365396295	0.62062211514432	0.36773964080513
H	-1.16314874234810	2.87843490713234	-0.83133413461801
H	-0.18360023201188	0.32796296206145	-2.82739477296291

***cis-trans-N-mer* AB**

O	0.28597804304979	4.59666359828664	-1.26806628086598
N	-2.29354060564708	2.03444778657221	1.13058231627105
C	-2.75050782450408	-1.79593863051719	-1.39482100493730
H	-3.55774497158127	-2.51154882934061	-1.21362540382830
N	-1.68207786135284	-2.05060920208109	-0.45290474172465
H	-0.78291809902282	-2.25670314224175	-0.86273601677258
C	-2.19343734151275	-2.07840325153442	-2.77835988575018
O	-3.06902538929353	-2.08716969577384	-3.77847441898995
C	-1.84972162325862	-2.20936497293723	0.86687005378581
O	-2.91311207440495	-1.99734218716026	1.44152097650688
C	-1.28932025538335	2.81695309920593	1.55324873591227
C	-1.46116807641583	4.29366351968571	1.38180776213074
H	-2.43404645982064	4.62227788042122	1.74189116133096
H	-0.67510754459081	4.78747282119498	1.94478131986110
H	-1.35728946797494	4.56485929863294	0.33239023852343
C	-0.62082667750212	-2.67962877138835	1.61775781713648
H	0.05303225038203	-3.18457704004119	0.92855514511739
H	-0.94960041393948	-3.40067771429053	2.36261313693614
C	0.16697883105951	-1.57124430005900	2.33331433001735
C	-0.57446714257562	-1.07697579773795	3.59078416602126
C	1.49740441957486	-2.13528531305554	2.85750700797335
H	1.29091702464943	-2.90666795558126	3.59404562674747
H	2.03937873630151	-1.33031338349066	3.36020817972637
C	2.43872208490899	-2.74174789652958	1.83511964236514
O	3.10052206529548	-3.73316865914318	2.09842776898478
N	2.53730644551181	-2.09311368148063	0.65501033665256
H	1.87687105809439	-1.34251754451405	0.50263939721234
C	4.74129815299718	-2.20018259686517	-0.46424389436008
O	5.54915014882442	-2.67975110384259	-1.21527438522712
N	1.24339659603893	2.55380958196387	-1.35123010299180
C	0.57373120286517	3.59729678502060	-1.91006983858852
C	0.15513988582935	3.43869966667019	-3.34783344335472
H	1.01898969488887	3.53051461861530	-4.00700090992906
H	-0.31236663007639	2.47351281700351	-3.53444352528308
H	-0.54845278081999	4.23341360051375	-3.57660365563757
C	2.00051731906186	1.55790499170398	-2.09131314445812
H	3.04071026990502	1.89112330025520	-2.16358067858449
H	1.59905367153653	1.51447121530081	-3.09861901528635
C	1.93796933530668	0.18630327245973	-1.45274272999803
H	2.33983026711668	0.27309459029766	-0.44450734261098
H	0.89430182403619	-0.12572515945848	-1.36730202073095
C	2.72283048732840	-0.83061679867525	-2.27201478213945
H	2.35674471341315	-0.79976624581693	-3.30102621111444
H	3.77285220280046	-0.52709112266380	-2.31872054449610
C	2.62698229615149	-2.28216637630196	-1.80838550228534
H	1.58166756040522	-2.59131720581127	-1.75452353881835
H	3.10364926099117	-2.91360594017034	-2.55692974124227
C	3.27821112121993	-2.62866698154506	-0.46428555692615
H	3.29998186660134	-3.71370027260106	-0.36445261713951
C	-2.92872732168754	2.12850211846822	-1.28597333449544
H	-2.18489173629352	2.87816999706483	-1.55963035967512
H	-3.81340806988697	2.33198838986490	-1.89479616196221
C	-3.33586006284358	2.36089583383662	0.16690427361321
H	-4.20698579479120	1.76689051206695	0.44058921409940
H	-3.60680237774055	3.40270027167394	0.31230393420790

C	-2.38085339469136	0.74289274350695	-1.62352439855224
H	-1.44535162365767	0.57919274027498	-1.08941777437095
H	-2.12918760423356	0.73443682940334	-2.68709830456077
C	-3.35393431106173	-0.38556535615981	-1.31667205373007
H	-3.75717887673167	-0.28956718015336	-0.31385187520038
H	-4.20909239816163	-0.33583918127352	-1.99465488405620
O	-1.02696312998785	-2.27049403321659	-2.99999469851838
O	5.09787616656083	-1.24930709423726	0.39393902099476
O	0.47851758551673	-0.49714415168005	1.47405433123766
O	-0.62382167185523	0.22554279722074	3.79645260560647
O	-2.07997662655333	0.67560090963889	1.26559013900865
O	-0.27375941740237	2.33391060467163	2.07110613479697
O	1.77392927491530	2.78184804255883	-0.08719695317038
O	-1.03062811513455	-1.84892083018120	4.39475029174805
H	1.09399237412779	2.52288398712998	0.56145930770005
H	-0.31719511032551	0.78500418326069	3.04125999102631
H	-3.98094788763801	-1.95736052575785	-3.47881715706341
H	-0.32939477889508	-0.07267655590420	1.13925071104464
H	-2.76802056742077	0.32961201657210	1.85600668032305
H	4.33006487940432	-0.97358873980384	0.92327113480706

cis-cis-C-fac* AB

O	1.02386455099725	2.51860121138648	2.23196457496271
N	-2.01028045293132	1.87423375267917	-1.50619808774859
C	-4.19504793889855	-1.17209821041272	0.97142249271732
H	-4.28760636720997	-0.76492152803196	1.98039186125679
N	-3.00077841885990	-1.97014656372854	0.91226841089886
H	-3.02086281878422	-2.79593312419903	0.33611103869815
C	-5.38949949423448	-2.06913846107507	0.72060181022712
O	-6.58744211116672	-1.52851118004216	0.94579599032441
C	-1.84617382974702	-1.58830537802038	1.50591808406535
O	-1.78510077947839	-0.57681446806883	2.20072476931145
C	-2.21084157871440	2.99761705346730	-0.80077166052696
C	-3.19554114413878	3.98200473746337	-1.36834648365971
H	-2.89100113556265	4.30716507484728	-2.36291536077153
H	-4.19122198452490	3.54907036358506	-1.44693235888408
H	-3.22947155666937	4.84103448215751	-0.70559722361509
C	-0.72418147803623	-2.49735714803230	1.23632601307404
H	-0.96978357575717	-3.48020620337250	0.86101869801234
C	0.57547058342815	-2.20221648500442	1.33801206533712
C	1.63549948896540	-3.19306985538871	0.95353127274770
H	1.22044464452929	-4.19689978227846	0.97586736408658
H	2.46138324301570	-3.14196638020021	1.66515421910512
C	2.12275391571854	-2.93969061717031	-0.46589994502505
O	1.46792404387736	-3.29609080002908	-1.43518895759412
N	3.28769458672427	-2.28886957610289	-0.57255663873436
H	3.83141034947905	-2.07424430011534	0.25002051565667
C	5.34595243663952	-1.63521367863035	-1.58539214908477
O	6.10324463592491	-1.42197756316578	-2.65763503150579
N	2.15340436470105	2.36790414192093	0.31824551490745
C	2.09972253566688	2.34924851773152	1.65582086824501
C	3.39062117339407	2.12618887013390	2.38743362804467
H	3.15470849230198	1.82664553983720	3.40440607540203
H	4.00655143992055	1.36337414144135	1.91693140703437
H	3.96192485258267	3.05533778492807	2.41915289887599
C	3.31292400952018	2.43453990883801	-0.55183174637796
H	3.03726536922461	3.10963247137182	-1.36113096915367
H	4.12542579537761	2.90409206807935	-0.00117366073642
C	3.75850086716361	1.09031343521223	-1.10416187510044
H	4.58521487240198	1.28539773123370	-1.79293982376383
H	4.16331799917384	0.49059860093265	-0.28797956982785
C	2.65249769184518	0.32632150524914	-1.81595428749290
H	1.90846799408375	0.00318496478419	-1.08554635011241
H	2.13261649944618	0.99642973407785	-2.50440155550702
C	3.12605659428992	-0.87257971428535	-2.62567547453645
H	2.26030022867061	-1.35311086317352	-3.07661799888014
H	3.75283061253327	-0.53187590261445	-3.45218757054728
C	3.88604013055395	-1.96344626878061	-1.84437384124467
H	3.89110559129333	-2.86115577350862	-2.47029319462484
C	-4.07373995223535	0.76044864536281	-2.45224154709480
H	-4.80257236005607	1.50523065504660	-2.12564023535989
H	-4.41069395766753	0.41839822541778	-3.43285298828261
C	-2.72242239091958	1.42618185480010	-2.69175454454732
H	-2.85082442654386	2.28150056378740	-3.35286332432131
H	-2.04550262819177	0.73308934191274	-3.18766473698781
C	-4.08858594819364	-0.41680250986263	-1.47894090107914
H	-3.20838060085665	-1.04250727365537	-1.64049317297009

H	-4.95653542546633	-1.03723319960584	-1.71556789036361
C	-4.16750673414400	0.00574046013540	-0.01701518616858
H	-3.30895748110572	0.61236230410493	0.26011074672872
H	-5.05388276420752	0.62493968753230	0.13871677997560
O	-5.31142377979982	-3.19784519449457	0.31433258628096
O	5.83447593606600	-1.58079127311167	-0.48789583611602
O	1.00517060390773	-0.98959676703555	1.72771504614070
O	-1.17216001707821	0.91050626179475	-0.96761955809528
O	-1.59587437666312	3.22571805843151	0.24331261052277
O	0.95498083651895	2.61589634672625	-0.33896025238557
H	5.61654825484524	-1.53748422529297	-3.48695562373892
H	0.31366262490803	2.90206051276470	0.35349942704036
H	-6.52573732635734	-0.62996104018885	1.30202428555676
H	-0.30527150982308	1.34625285287551	-0.83241411986543
H	1.96903849433306	-0.95489355337176	1.76490467719532

cis-cis-N-fac* AB

O	2.74855627707650	-3.25830316684644	-1.19907725767198
N	-1.59449495550065	-2.21191203969044	1.11046111482046
C	-4.23541024166973	2.39581212937358	0.11356125164137
H	-5.06340258947336	2.76974390995232	-0.49280097970162
N	-3.05573788871796	2.49031261310218	-0.72080206553835
H	-2.16537017320729	2.23314752088178	-0.31926561153792
C	-4.15814621468383	3.35947238128982	1.29140834628112
O	-4.80087662915207	3.22051992175760	2.29680299418198
C	-3.13031074194501	2.58983344623126	-2.06614005981381
O	-4.17557144966273	2.76149420393154	-2.66629669035890
C	-1.54924854928241	-3.14356723890517	0.14520989874161
C	-2.62855716790834	-4.19128089451774	0.16922281167550
H	-2.53206645854086	-4.81192049614821	1.06065263701675
H	-3.62523239312466	-3.75494381356841	0.16940670564300
H	-2.50733671007759	-4.81498348934939	-0.71100080598464
C	-1.80669959165430	2.54735811860141	-2.81622942346295
H	-1.47770720820097	3.58071438118353	-2.92503729267945
H	-2.02377586372284	2.16668568751569	-3.81451000080841
C	-0.71793500305054	1.73368290377972	-2.18779401805833
C	0.25894027920207	2.31400189606138	-1.47452291276149
H	1.24451089422882	-3.14826190809951	0.05756080997916
C	1.39447069870378	1.64518023106947	-0.84654872502153
O	1.67832108201198	0.44835941115831	-1.03642051438570
N	2.15218083100926	2.44244658207438	-0.07505632688190
H	1.85100064390192	3.40048191808680	0.00242384557004
C	4.44070286522027	1.37342956968059	0.14261959310511
O	5.57426338655609	1.60221035278098	0.49561861113741
N	2.87559687095054	-2.82567659585527	0.97602832090841
C	3.45301260590423	-2.99973237672079	-0.21689573979381
C	4.94090458980173	-2.86113693874093	-0.30729470821445
H	5.29529322592090	-3.53350460585472	-1.08453098091152
H	5.17718893297851	-1.83765860544389	-0.59888656799380
H	5.45077763063567	-3.08167971049015	0.62624317951353
C	3.43274007872787	-2.41000917739131	2.25001762265665
H	2.69806907057001	-2.70116038760546	2.99914445073801
H	4.33179231320068	-2.99309287173378	2.43892760047830
C	3.73639062497283	-0.92233833124537	2.33533922077029
H	4.15810886994575	-0.72968160791222	3.32491506949758
H	4.52236901018517	-0.68694180289338	1.61794598903610
C	2.51873375224851	-0.03055888004595	2.12413389471553
H	2.02149483541005	-0.28922980135313	1.19089993787803
H	1.80137333938795	-0.23071622662027	2.92275939597601
C	2.85312547044356	1.46189393955351	2.13416578178056
H	1.99067808489894	2.03408697431006	2.47680533258738
H	3.65446786653999	1.64791390124462	2.85214685389526
C	3.27728209582913	2.11079563092638	0.80208767245436
H	3.70011388633367	3.07868919736162	1.05890668081761
C	-3.79200534400194	-1.16294695198172	1.63350946543540
H	-4.28376361451352	-1.66802744412259	0.79834017679626
H	-4.52645864039197	-1.11184755349999	2.44108228735250
C	-2.62834549088574	-2.01799285680430	2.11352883875914
H	-2.98403687247283	-2.99635290844389	2.42922397790928
H	-2.13554663423938	-1.55946528723711	2.96958830040445
C	-3.39666036111346	0.24043708375657	1.20238079382731
H	-2.56408551046280	0.17444188799317	0.50120125034210

H	-3.03595595826991	0.80344190295879	2.06821552137243
C	-4.55562769938019	0.96765513399689	0.54050507000393
H	-4.86165386794053	0.41936817669779	-0.35252603176457
H	-5.41733591180166	1.00077634506492	1.20688569366817
O	-3.37475100507111	4.42582158730907	1.13733675509895
O	4.18165465133759	0.46728900586234	-0.77290549620575
O	-0.79531581729809	0.40908412586671	-2.35152348219474
O	-0.64313812051408	-1.20504290084148	1.08178171209758
O	-0.64413857242118	-3.16799755320486	-0.69113041997301
O	1.49069280215720	-2.93560345697263	0.98840226763850
H	-1.54622881056964	0.16955121184940	-2.91073868321153
H	0.20624030195119	3.38798285457420	-1.37186504241752
H	0.22169511976007	-1.65760226504857	1.16412570420988
H	3.17904907432324	0.34787410284400	-0.90208607583061
H	-2.91397800140258	4.38707690450670	0.28288447476506

cis-trans-C-mer* AB

H	-3.53659612230683	2.87854806803397	-3.43982296664483
C	-1.69393046081173	2.24207657616861	-2.50977079794542
C	-4.12153162762578	-1.38992251119491	1.90919231742230
H	-3.92820641982019	-1.25701693014545	2.97548707594230
N	-3.04528152129887	-2.15320626206137	1.33346097204537
H	-3.28453971357174	-2.77724074307268	0.57941305586106
C	-5.39857116134584	-2.19306856616365	1.76404917419905
O	-6.45400451144160	-1.75381102051803	2.44992580907201
C	-1.74432287637606	-1.85296147635611	1.55251763077021
O	-1.40332098169473	-1.04609670318059	2.41066880833506
N	-2.21313062264783	1.84077486836227	-1.35094020201884
C	-3.43915613383351	2.30135289937555	-0.71781037463660
H	-3.69303320741734	3.26791298218127	-1.14423994365597
H	-3.19456088462357	2.47175795125841	0.32992802385013
C	-0.82674228111404	-2.56838994399856	0.64588362238665
H	-0.74953184456594	1.39481334524943	-0.21501342716766
C	0.23858479778384	-2.01079209097680	0.06751619063348
C	0.97799291222036	-2.69873259174794	-1.03905312125687
H	0.87348002453412	-2.09808927922379	-1.94981630493733
H	0.52541020302889	-3.66422507928829	-1.24865523575852
C	2.47184592428341	-2.92501866259763	-0.88847860017000
O	3.03841527960463	-3.72532087947046	-1.62277470231253
N	3.12428822321041	-2.18709581075679	0.01732294503536
H	2.61505012167226	-1.52088094304017	0.58073091227294
C	4.94614994032385	-1.42247792914088	1.35564143126294
O	6.24440239923386	-1.40568880512215	1.65091463150775
N	2.09992140625130	1.98020276883266	-1.04011260827704
C	1.53936737343955	2.65700643093760	-0.02894703460624
C	2.35225859005884	3.74333417789772	0.61391207227811
H	1.75411192273964	4.18005945255724	1.40726257351115
H	3.27606982853222	3.34689261374134	1.03361450297191
H	2.61131513968554	4.51689371567666	-0.10810042466590
C	3.47502416420011	2.06663025072951	-1.50169157505827
H	3.45059709458448	2.02991726306506	-2.59133121796709
H	3.84342033689621	3.04812623444957	-1.22293285205867
C	4.38348181786148	0.97274144035541	-0.93908354460065
H	5.40059510924812	1.36659159505568	-0.88235845397107
H	4.07127102087837	0.77053372279802	0.08670593411205
C	4.39088930037314	-0.29890564399709	-1.77911400280919
H	3.36708922969895	-0.61455120649095	-1.97378156255663
H	4.82268346749742	-0.06355619383420	-2.75450546152663
C	5.17943478028376	-1.46376526363400	-1.19604609638527
H	5.24767079251106	-2.24425349605587	-1.95073593257137
H	6.20364074350805	-1.15489052943583	-0.97692668476358
C	4.56260187652554	-2.11947819624886	0.06491263733472
H	4.96698669717000	-3.13262360390691	0.13586844987631
C	-4.62326232572793	1.35441440677454	-0.86526327769043
H	-5.48579031087686	1.85734113899421	-0.41968964666296
H	-4.84413785380086	1.24017033559732	-1.92867444585675
C	-2.50049621207003	3.19549144151996	-3.34199948300915
H	-2.48888616264390	4.18783675329663	-2.88950157431708
H	-2.04188014118592	3.25368219400143	-4.32433200338101
C	-4.46656797426384	-0.02811162554155	-0.23932051750380
H	-3.62977789181116	-0.54712461088750	-0.70731095194654
H	-5.36462818458300	-0.60409762461510	-0.47758899380243

C	-4.26395464114687	0.00448267751924	1.26865446925712
H	-3.35392766098332	0.54767585622789	1.51536178248170
H	-5.08824222590000	0.53765155346264	1.74826538823976
O	-5.50650457969809	-3.16189591130031	1.06043865178320
O	4.15715956057322	-0.90767384453942	2.10276357921277
O	0.67683133137379	-0.78583129046999	0.42030509100019
O	-0.57800439805675	1.85964225464119	-2.90197544183638
O	-1.51247947460861	0.90260563488560	-0.62642563575896
O	1.39753202130959	0.90482584992377	-1.55373001512522
O	0.38887661385557	2.42074940729911	0.35802713508446
H	-1.12987378877138	-3.54975579564196	0.30878514889065
H	6.78109174682971	-1.88239672379065	1.00069658847957
H	-6.23986262761525	-0.99541693858497	3.01290085351887
H	0.99771792031183	-0.26522504412713	-0.35478789734078
H	0.59666611214629	1.26724991028903	-2.05722944607542

cis-trans-N-mer* AB

H	-2.22232980900245	5.43132225623831	0.29419357191556
N	-2.37328033691270	2.43561989399895	-1.09040595916125
C	-3.34718452466573	-1.38392327609247	1.07248038960609
H	-3.00883367262025	-1.42988246127325	2.11086767816281
N	-2.25478004219609	-1.73249020088924	0.20487867705330
H	-1.89679267640472	-1.04462210781899	-0.44117478015445
C	-4.50123056301213	-2.36869104514374	0.92980638099507
O	-5.29238063749675	-2.48199718031048	2.00305499256767
C	-1.61877792704382	-2.91237795091069	0.35027572079031
O	-1.99649095207513	-3.72828190663336	1.19611144932702
C	-1.88642823284972	3.47565554644349	-0.41114993582621
C	-2.71526424787055	4.72669616410927	-0.36820151468272
H	-2.79409126664848	5.16738282580138	-1.36210859566193
H	-3.72188112076154	4.52882140859256	-0.00424539521034
C	-0.47936181542034	-3.23103994266325	-0.52131458877038
H	5.55778196910251	-2.34949124544309	2.36883002621273
C	0.16880735904453	-2.42223780449597	-1.37053118222753
C	1.35264721722071	-2.88149862135961	-2.16320889812328
H	1.37125410045040	-2.38123824583367	-3.13131260071887
H	1.26512506425573	-3.95109892709022	-2.35495891153316
C	2.72916415479559	-2.67786717964710	-1.54261256836076
O	3.72695660349188	-2.73230930520208	-2.24937151008456
N	2.77679430575598	-2.48468172582478	-0.22083034852771
H	1.92344849229481	-2.47502595092863	0.31926053551605
C	3.67357022169369	-2.34859513088151	1.98387143196939
O	4.70377456446095	-2.34880267951771	2.82552878095773
C	1.60418881171997	1.87206369181226	-2.21962905121783
N	1.92610062357487	2.12608281838786	-0.95353537767986
C	3.21038503872553	2.60472548960547	-0.45433366331562
H	3.55865337579983	3.41034637633995	-1.09703869254683
H	2.99992440516233	3.03187437927434	0.52366075879150
C	2.55594106134281	2.32522275066465	-3.28578981705357
H	3.49003173818889	1.76775063559842	-3.23725027960580
H	2.08845682265330	2.15051605226005	-4.24956757640691
C	4.26074798871771	1.50384291050860	-0.36010407459082
H	4.56568117690737	1.21821332703071	-1.36882326262533
H	5.14597289409517	1.92334016434929	0.12323080368392
C	3.77722227094450	0.26172623104255	0.37401282302769
H	3.67008384239760	0.47165419849923	1.44160536988283
H	2.78316181428665	0.00861805514369	0.01005447196427
C	4.68125083942501	-0.93816056950298	0.14826027570195
H	4.94918866118634	-1.00356700332945	-0.90507246609497
H	5.61731749964525	-0.84328593619395	0.70162733664348
C	4.00712856649352	-2.27402809813460	0.50618931298938
H	4.70563428543562	-3.08405511211569	0.28039656374526
C	-4.76703732633056	1.68342695304224	-0.93532209354349
H	-4.95425991225093	2.27664107798528	-0.03617647133933
H	-5.66991788517317	1.75641845466046	-1.54413729756930
C	-3.66107052512916	2.33703400001163	-1.75480683341521
H	-3.96194558769120	3.33958433892906	-2.04639553027424
H	-3.48756970475904	1.77128394367407	-2.67029732733892
C	-4.53329907802201	0.21919961787978	-0.55941723086150
H	-3.96341066272709	-0.27000631877367	-1.35106959258463
H	-5.49784327729981	-0.28888846472652	-0.52145399955642
C	-3.83727581540804	0.04104271834920	0.78695631225412

H	-2.96928104326051	0.69354416549870	0.86508107043611
H	-4.52114763206418	0.33195551959478	1.58598173631928
O	-4.76751441823919	-2.97818373460687	-0.06892343102764
O	2.55069019422032	-2.38721896973084	2.41210922526429
O	-0.23278113677777	-1.15459529514366	-1.53301092536701
O	-1.67256743212874	1.25056685384127	-1.04601323599040
O	-0.78207069103243	3.43400755385047	0.15063979083618
O	0.54107872268565	1.29755769193190	-2.52617469273920
O	1.09032054868350	1.63983940124934	0.02792190977943
H	2.78886624210891	3.38401741557979	-3.18249658944537
H	-0.85595413600279	1.34557639252570	-1.60453151392239
H	-4.96065407772148	-1.97150332392884	2.75607997196765
H	0.32293182290007	2.28210930244693	0.08261798966624
H	0.35514109654594	-0.58156429865177	-2.05705775714642
H	-0.12896922941495	-4.24807856395295	-0.42872478572493

***cis-cis-C-fac* [Ga(AB)]$^{1-}$**

Ga	-0.12337096997249	1.40495817369822	0.38623814398361
N	-2.42668942612961	2.51660478467623	-0.67864079036078
C	-4.26204670615241	-2.30087966334219	-0.23468619956825
H	-4.75727805793570	-2.94191284752550	0.49917432245093
N	-2.84606641993019	-2.25898928111352	0.08331707444965
H	-2.24727863829306	-2.59558146198953	-0.65659874248420
C	-4.44964719020849	-2.96758399902256	-1.61374004627987
O	-5.63175665869847	-3.17040818104930	-1.96141268969411
C	-2.35795200579379	-2.05662385409133	1.31234031365195
O	-3.08044727311807	-1.72129991897112	2.25423375796322
C	-1.89593375668539	3.51827637163769	-0.02038030548858
C	-2.54362693376702	4.86619028534609	-0.00399293369133
H	-2.61562507415114	5.28024238752093	-1.00912757993774
H	-3.54760817193474	4.81687694337445	0.41558391570847
H	-1.93580709199734	5.52565517129083	0.60813446179086
C	-0.88361732684208	-2.33218200399398	1.51336948470505
H	-0.50263695969226	-2.96744302788539	0.71769428719603
H	-0.81041112223533	-2.88202260328606	2.45081372190706
C	0.05203332294688	-1.10484638344955	1.58636800991082
C	-0.44795523610387	-0.12217075306394	2.67524159605296
C	1.46263996682826	-1.56486681263917	2.04237489091611
H	1.38138778119014	-2.24096537496737	2.89243407524247
H	2.01872373699776	-0.68545527519810	2.36580902909199
C	2.22715029199610	-2.26325787908968	0.94317035689400
O	1.86384862455532	-3.34162437632587	0.46750089994068
N	3.33206028868636	-1.63628541424531	0.53175970707002
H	3.66307480481169	-0.81714052463348	1.01791976466162
C	5.60687594365568	-1.44610893159546	-0.31726615894726
O	6.50024645166464	-1.82839396596607	-1.09914911125238
N	2.20370137448601	2.00442186243254	-0.99515229606177
C	2.54646130690106	2.13373343292114	0.26286935822885
C	3.95899342658337	2.39711479052172	0.66063370172544
H	3.94769262141297	2.98492569061412	1.57557574143344
H	4.47158405823805	1.45370807965028	0.85906063523531
H	4.51572927213075	2.93031244426584	-0.10539575013477
C	3.10471796613765	1.90664524888171	-2.13759489522337
H	2.46697013464192	2.02104259521872	-3.01093606568133
H	3.79727757748375	2.74690721419275	-2.11911268969618
C	3.86450430182904	0.58763984617622	-2.17360545017476
H	4.50880532355764	0.59261138269640	-3.05672014808618
H	4.52774608744526	0.55389998017170	-1.31089728331937
C	2.95461803646217	-0.63406689223492	-2.18883174444082
H	2.16114906414752	-0.50217738397321	-1.45322500419238
H	2.45923288931469	-0.69720574839573	-3.16147740469549
C	3.65473742680471	-1.95935436661287	-1.91463990893873
H	2.93502608059119	-2.76201282196186	-2.07864429360310
H	4.47553556981670	-2.11856950739599	-2.61505857348062
C	4.22724822063179	-2.11966457673871	-0.49421470676753
H	4.39588305667335	-3.18656918096502	-0.33340768379783
C	-4.67594893335343	1.51230173677372	-0.85514862281405
H	-4.67106592227679	1.63070362440083	0.23125274946471
H	-5.67091464531951	1.80935247630704	-1.19308561575053
C	-3.66580338125273	2.49881830620403	-1.43318145494954
H	-4.06873806464667	3.50698201269403	-1.42858892228739
H	-3.42035474791498	2.23964288380611	-2.46576627652127

C	-4.40680230653365	0.05088411626228	-1.21766452902108
H	-3.33309362748442	-0.08589674866377	-1.30290332973345
H	-4.83898655864931	-0.17184207226686	-2.19667911232459
C	-4.93083251110120	-0.92620063547063	-0.17686883824092
H	-4.74923853363220	-0.52267811931893	0.81903399352758
H	-6.00795059520822	-1.06687330592751	-0.27599106932352
O	-3.41962863235769	-3.24746857975568	-2.26778865562805
O	5.72297817206912	-0.58966175143356	0.58988260194357
O	0.13597345064933	-0.47280364056560	0.35949567480361
O	-0.73740161925802	1.05371487755835	2.25446392959229
O	-1.77818215824931	1.32487146515495	-0.62979755163518
O	-0.81066462786761	3.32378465020908	0.60592515673472
O	0.88888474342156	1.78081376124148	-1.26825783535067
O	-0.51373590188950	-0.47118859348141	3.85183864869166
O	1.65010641187427	2.02341986270727	1.15628026461035

***cis-trans-C-mer* [Ga(AB)]$^{1-}$**

Ga	0.29718086362679	1.43822552869105	0.97517887385656
C	-3.15380337718113	2.58441373148099	1.90463848833724
C	-3.08795586374543	-1.99127275982539	-1.17745157417490
H	-3.91123188073728	-2.69233087461041	-1.01421726942392
N	-2.10512938895027	-2.15817741846301	-0.12378192167056
H	-1.19078368775824	-2.45007551486393	-0.44685294324185
C	-2.44734678120708	-2.38631198171317	-2.52863677877823
O	-3.23174123156012	-2.45029161762943	-3.49803747286716
C	-2.25338141423163	-1.84101682395244	1.16322197648951
O	-3.30063455506900	-1.38960968075490	1.63790342115188
N	-2.27779739184330	2.67553120303288	0.89123419568532
C	-2.58930355728032	2.99543876224603	-0.50436822127362
H	-1.61861649916935	3.15254132425076	-0.96557065288017
H	-3.13666165355549	3.93955305755740	-0.55517827489441
C	-1.05942167433083	-2.14252142129910	2.04145864834871
H	-0.70509906047338	-3.13470473970699	1.77643446416888
H	-1.40395161940093	-2.16886376892115	3.07140966806384
C	0.13013091600524	-1.15020122548854	1.93090584746753
C	0.19726299903351	-0.22228386791628	3.16841566785614
C	1.46299425387899	-1.93612203541839	1.97081582779202
H	1.41654266801746	-2.69490039789281	2.75250549391675
H	2.27480694889325	-1.25167017909346	2.21800603138463
C	1.75901755773168	-2.60386090337756	0.64559860595553
O	0.96261372686975	-3.36895996692399	0.09867602999063
N	2.94745862392669	-2.30186610430466	0.12058174279288
H	3.59819574825356	-1.72198103939058	0.62914340755649
C	4.96137414707067	-2.58149976521058	-1.21794514896617
O	5.53131710027035	-3.09281058909041	-2.20348324430237
C	2.57638701327748	2.34834631393475	-0.19359507338928
N	1.80975274592621	2.08433399424999	-1.21738905939332
C	2.19598292845316	1.91011701927111	-2.60824218476473
H	3.20136584775914	2.30082951012536	-2.73818729161842
H	1.51244650860931	2.51071553860119	-3.20782820918941
C	4.01244215730423	2.71807352651089	-0.33549216569644
H	4.30719946454415	3.27414396542177	0.55039959354255
H	4.20342902310880	3.31913461641139	-1.21953927790504
C	2.12294820809775	0.44183819999780	-3.01174520264307
H	1.09238807800952	0.10258873924170	-2.90036292570742
H	2.37083530586926	0.37317890522734	-4.07289694026305
C	3.04416806277429	-0.43430610561566	-2.17851744116494
H	4.08579831353881	-0.22183295274814	-2.43359252781673
H	2.92117479592009	-0.16077213911973	-1.12973257019987
C	2.76537915289138	-1.92482573755050	-2.30015964924216
H	1.68911505942987	-2.09712441238551	-2.24913981536600
H	3.11180292154788	-2.31254226297273	-3.25893184420365
C	3.42653328767658	-2.73290027773416	-1.17482921020718
H	3.18806893175270	-3.78748225659217	-1.31109733374430
C	-3.34573895361497	1.90981380056834	-1.25969171783668
H	-3.46591478366387	2.25848929402786	-2.28816017373791
H	-4.35434203363195	1.82068824811361	-0.85871753093949
C	-4.62210611185498	2.64736555281246	1.56518235389160
H	-5.14402297601484	2.98830356811283	2.45545039240935
H	-4.84911412793411	3.31245249294607	0.73604354099200
C	-2.67371744042153	0.54059974576196	-1.23259375293770
H	-2.08323872036758	0.43944574655907	-0.32354354412974

H	-1.96942446364093	0.44763569901278	-2.06229413977786
C	-3.69145605075607	-0.58633128062697	-1.25913673816083
H	-4.37333406477617	-0.45036832605507	-0.42102897003169
H	-4.28652033456570	-0.54585316182968	-2.17242219462723
O	-1.21556461118269	-2.60438916110151	-2.55033972474836
O	5.50440376804841	-1.94718285289377	-0.28493967178538
O	0.07471785317003	-0.38530262478643	0.77034009680446
O	0.45116554507613	1.00725335364825	2.90163438223574
O	-2.80439527623239	2.44042924322020	3.07846374188484
O	-0.94965596047512	2.81594947734382	1.22651797963000
O	2.07078004199638	2.25344755622533	0.98056414228957
O	0.07947388046637	-0.67380908481992	4.30267906381485
O	0.52626624549646	1.73476736564238	-0.96075028073270
H	-4.98862121760978	1.65130175126061	1.31615623801922
H	4.61947606891410	1.81322848117113	-0.39203925189472

***cis-trans-N-mer* [Ga(AB)]$^{1-}$**

Ga	-0.23385445309218	1.29540384533714	0.89213585187622
N	-2.64477602157015	2.56164059957340	0.79500815866229
C	-4.08525337055500	-1.77136752806396	-0.75453135456145
H	-4.77091425731547	-2.39322680048990	-0.17569107095693
N	-2.73887909905768	-2.02310969326841	-0.28369164560442
H	-2.07580536868799	-2.22588369858554	-1.01653753554947
C	-4.19292528585622	-2.21869632298898	-2.22729465288365
O	-5.34484255622322	-2.22834135471664	-2.71006088675546
C	-2.37596245762328	-2.14366050347645	0.99596879708591
O	-3.16305368817082	-1.96368347060891	1.93062608768950
C	-1.82677792524596	3.53829080442140	1.10529908389909
C	-2.24159510609683	4.96838204708181	0.96737630991283
H	-3.13736298213314	5.18393584799779	1.54798455709650
H	-1.42815794712682	5.59464053809532	1.32137481537788
H	-2.44664636489992	5.21196159446621	-0.07511147258711
C	-0.94478125249288	-2.56728941238889	1.23867137883329
H	-0.58709792607741	-3.15654538620124	0.39716856024181
H	-0.93906306648096	-3.19228628868674	2.12944532975047
C	0.05875243163520	-1.41223805132726	1.42609060730890
C	-0.15087926722531	-0.68287665674817	2.78222152713593
C	1.48379589978329	-2.00035942953518	1.59621519458016
H	1.44321160204081	-2.82815038092405	2.30727882817982
H	2.11483447693694	-1.23343824505141	2.04688257210819
C	2.19032299258248	-2.50654775138408	0.35469847604255
O	1.63303469562266	-3.11745246471595	-0.55654945006231
N	3.50699420420580	-2.26373517824608	0.36218441697896
H	3.93608961860448	-1.82698243169296	1.16596439811416
C	5.86494798729593	-2.75971580363939	0.06822938913765
O	6.77596316036719	-3.31519075826747	-0.57845834746637
N	1.89583687745044	2.05102231705936	-0.68467155695818
C	0.86041759238193	2.14948740425115	-1.49072299105162
C	1.02775941304748	2.39546854968602	-2.95200094768957
H	2.03715175658798	2.67565702594953	-3.23324950791822
H	0.74313386858458	1.49429410831369	-3.49578634599651
H	0.33943422230228	3.18847803504007	-3.24046607926846
C	3.24842335317255	1.69617841176170	-1.10475472917267
H	3.87351990721254	1.83509503397249	-0.22576744712816
H	3.59256517329392	2.38881921670525	-1.87033922174068
C	3.26652125660591	0.25067093007986	-1.57570867447020
H	2.64840756313323	-0.31288283428077	-0.88254506834016
H	2.77789596423649	0.18494653111673	-2.55076470535429
C	4.63759241272771	-0.39992151765631	-1.66369271662344
H	5.21049262239434	0.05727884090271	-2.47334868565075
H	5.19704206785363	-0.20836346975449	-0.74375116561709
C	4.52034267696128	-1.91002256123985	-1.89526863111079
H	3.62491980931184	-2.11444181958454	-2.48558780472011
H	5.36873217921355	-2.28087919892099	-2.46853631042314
C	4.47140135726245	-2.74162681858133	-0.60519953723330
H	4.22570438738638	-3.77052989004779	-0.87295854996952
C	-4.12200309556812	2.14198817857352	-1.17307000033778
H	-3.51937087152967	2.78538467175261	-1.81854782878375
H	-5.16569882992904	2.30942937034621	-1.45549785966990
C	-3.98963473200815	2.64036590635329	0.26381804799692
H	-4.62846104900607	2.06448529685220	0.93411267925796
H	-4.31225012152436	3.67674818132816	0.31593584799674

C	-3.75798506436621	0.68120472373061	-1.44157392008959
H	-2.69093567916622	0.54245563042496	-1.28453351327521
H	-3.95712006238909	0.48080724072176	-2.49808169630443
C	-4.52758617108454	-0.31379467021683	-0.58658461115528
H	-4.41401142298349	-0.06332803681665	0.46560321349508
H	-5.59269159191587	-0.26077654751016	-0.82246040710012
O	-3.13253030252248	-2.51840576136268	-2.82126768688934
O	5.97243612988811	-2.21092146003624	1.18849800804164
O	-0.01256252285357	-0.51802574543073	0.37502338722136
O	-0.07045248397384	0.59947178411095	2.72858015715920
O	-2.18055333878165	1.29978026941571	0.96531342360115
O	-0.66031523083823	3.23668911055377	1.50453071938773
O	1.64620013977580	1.80995153844802	0.63628301863949
O	-0.29319360621135	-1.31579715267346	3.82433349042300
O	-0.30502322727601	2.03081151069707	-1.01546171676288

***cis-cis-C-fac* [Ga(AB*)]**

Ga	-0.26795890098251	0.97239815997472	0.09227930576794
N	-2.27271252405072	1.57390110268527	-1.67670920157743
C	-4.32906586967519	-0.77667258842095	1.44100906277818
H	-4.22686768405923	-0.25210805490762	2.39339387237559
N	-3.16412169407516	-1.60902126100703	1.21449803966650
H	-3.40170952947524	-2.58457183108609	1.08347051341809
C	-5.56063389608656	-1.70503924791868	1.56906564119293
O	-6.63181484689254	-1.14446793488204	1.87200020404208
C	-1.91862626287174	-1.19970261623271	1.00461218737663
O	-1.63617196182533	0.03229538997927	1.17603250596988
C	-2.29028654643188	2.57493316244630	-0.83258053994350
C	-3.30415565879278	3.66106358091772	-0.93215748240530
H	-3.80541440844475	3.69170343463184	-1.89402206322081
H	-4.05116164951526	3.52730565748241	-0.14886583545406
H	-2.79946993838702	4.60923801987041	-0.75583621541128
C	-0.95188380383918	-2.18382632990022	0.61799288336543
H	-1.24673240784361	-3.22162573161088	0.65896972451497
C	0.31195750702082	-1.87028627279410	0.20827538866765
C	1.32148507315617	-2.97808714686701	0.00998838722708
H	0.81368770542543	-3.91991267272659	-0.18755928529357
H	1.87125424238097	-3.08325975716707	0.94776218503428
C	2.27830150914212	-2.69467197084993	-1.13012797777930
O	1.93609646636012	-2.86688788724557	-2.30063340564168
N	3.48323503976737	-2.26049899413602	-0.76841487187962
H	3.72748803794190	-2.18169731340873	0.20947873493156
C	5.84528736968944	-1.70362935528062	-0.78395919255751
O	6.89140139906539	-1.43077034130500	-1.40436399560404
N	2.21170900741580	2.07724900956699	0.12943264998304
C	1.93154755711694	1.88620679327774	1.39371412217082
C	2.97703807048867	2.03901140773763	2.44837930800204
H	2.51208764713935	1.87537306751440	3.41584619768425
H	3.77506369129577	1.30973681355266	2.31049779539872
H	3.41986618978809	3.03386891437832	2.42718853416336
C	3.51560821350611	2.36605629241557	-0.44297881624222
H	3.33513478503070	3.05783509509163	-1.26563224356808
H	4.10626276908665	2.88672660695221	0.30674327597226
C	4.25469285875242	1.12659987534313	-0.92899978109452
H	5.24502979509062	1.45100550271487	-1.26092123658189
H	4.41286538786911	0.45979447218933	-0.07972614212235
C	3.54542627644654	0.39084797505773	-2.05636952687391
H	2.56850666924417	0.06480759580261	-1.70532258873161
H	3.35915731169235	1.09632568840731	-2.87098103636633
C	4.30092742739852	-0.79859888407338	-2.63068547306889
H	3.71927412251340	-1.19262888727003	-3.46343135748880
H	5.26435753769033	-0.48437935740448	-3.03330325355879
C	4.58559691550933	-1.94818290485047	-1.65356524881603
H	4.81546445434147	-2.83299364941724	-2.25413057022919
C	-4.63118615566059	0.80869987068258	-2.14422779766686
H	-5.17913230620893	1.67073320358928	-1.75839915549746
H	-5.19399165052331	0.45778024335358	-3.01125240306888
C	-3.27654303738677	1.25592355120417	-2.68900213099057
H	-3.40033897591109	2.11399025852300	-3.34861791993901
H	-2.82438310614587	0.45719200716576	-3.27047367601097
C	-4.58469337549037	-0.28656260798545	-1.07379137825470
H	-3.72406743895555	-0.93323080702032	-1.25426045575305

H	-5.47010270804626	-0.91781099917925	-1.17373000234485
C	-4.53542636225206	0.27141005534497	0.34512376124204
H	-3.73868285312680	1.00237610618298	0.43336496955253
H	-5.46989993509095	0.78854492404274	0.56463069569085
O	-5.38509254778947	-2.92601711217714	1.35620462059139
O	5.72116289056855	-1.81917080264905	0.45698129838039
O	0.76419667022244	-0.68354146823759	0.00330362743838
O	-1.28340090396796	0.64423436101226	-1.53798370056998
O	-1.42656067323225	2.61203769376327	0.09864862942242
O	1.20148419157542	1.92919166762172	-0.76885053363791
O	0.74391482330445	1.55044722753490	1.71254837322367

***cis-cis-N-fac* [Ga(AB*)]**

Ga	0.25485284080986	-1.56767158818426	0.23092332686346
N	-2.12732733943741	-2.23744424843492	1.32418601600757
C	-4.05227788186418	2.40182694608963	-0.35798166250379
H	-4.76576942404808	2.89579658377418	-1.02022782088052
N	-2.80532405580647	2.18511680232466	-1.06364349317009
H	-2.00937284960046	2.58297862848974	-0.57920096381160
C	-3.77236431766499	3.36232151186928	0.82045392686165
O	-4.77118985391365	3.73828644458598	1.46309760950144
C	-2.65445230584157	1.49619019672611	-2.19262209787137
O	-3.58388586488705	1.04144547673909	-2.85083796816213
C	-1.98070667049119	-3.07913220492272	0.33376170725076
C	-3.04939068989349	-4.02771997719814	-0.07575597753676
H	-3.86957752171223	-4.08929824791029	0.63057949711823
H	-3.43986751886143	-3.71195022684881	-1.04381767054733
H	-2.60166806194399	-5.01232890640047	-0.20120290670959
C	-1.20453443884096	1.29542214188126	-2.63309624062848
H	-0.80366722613347	2.23873510078075	-3.00155961339067
H	-1.21029564343674	0.56867689674273	-3.44208739650426
C	-0.37487941659301	0.75900749014158	-1.50059501105161
C	0.60546288886513	1.58065236581264	-0.92078059997089
H	0.79769796980001	2.54801236731197	-1.35592999938681
C	1.24642085114018	1.21208014720598	0.24598000886678
O	1.12939024046887	0.16119536071538	0.89274454877662
N	2.12994957925981	2.21795022470887	0.78418057990378
H	2.12729296568662	3.07434585959741	0.23778800208619
C	5.66590317410685	1.90844267089299	-0.71887549811225
O	5.55188398372193	2.34213716084564	0.34626853090558
N	2.79987870332739	-2.34123971588219	0.67669859319649
C	2.77653575993987	-2.04680756919356	-0.60121417961440
C	4.01265138166492	-1.88482880230250	-1.41142672539087
H	3.82840529347131	-2.31544922636323	-2.39364691517263
H	4.21194997125855	-0.82086604970321	-1.54546029338435
H	4.88438460519513	-2.35323584502479	-0.96751988882112
C	3.95015360783182	-2.21766034113731	1.56322317413052
H	3.72812358620356	-2.82616748479800	2.43743648954649
H	4.81680019064721	-2.64915363865103	1.07023031071869
C	4.21413144965760	-0.76404129223325	1.93713447424199
H	5.20643742379489	-0.69628066015808	2.38748227038825
H	4.25604779773229	-0.18331031632154	1.01338213616921
C	3.17088159994836	-0.18224606701099	2.88794072522562
H	2.18462120389047	-0.54303690155297	2.63608258936647
H	3.38807108857239	-0.52731741508828	3.90035302215417
C	3.18930655726787	1.33754829891532	2.94926323244437
H	2.48172547334564	1.69197775978551	3.71723832338082
H	4.15987227112917	1.68279595650334	3.31348665061526
C	2.88805503777818	2.25089587455836	1.82808519812420
H	3.37282420153682	3.21654377160529	1.92866838501446
C	-4.31693196161902	-1.07484021553487	1.39351803395141
H	-4.67372896979018	-1.58314988558121	0.49385819600736
H	-5.18517514312147	-0.94810333331809	2.04498989124482
C	-3.32935525814409	-1.98214401587845	2.11095329232976
H	-3.78893827105986	-2.93358241906215	2.36717389525957
H	-2.97425880333562	-1.52772017006218	3.03315186992156
C	-3.73503816797472	0.27772235433236	1.01503977356359
H	-2.79838204301156	0.12186287177780	0.48095199112209

H	-3.48224637481046	0.83318779461048	1.92273199514078
C	-4.67297656569234	1.09381967998333	0.14044169691063
H	-4.96833697299218	0.49733221149688	-0.72364404251567
H	-5.58313314804090	1.34861043535648	0.68432400698762
O	-2.57734143116347	3.66985616242502	1.03703562900817
O	5.77734576109657	1.47237883048939	-1.78129745031182
O	-0.66174218861668	-0.40165522977561	-1.10996513073967
O	-1.08455673190955	-1.40082068396323	1.59583047285924
O	-0.87967010132656	-3.07092571808443	-0.31323834826508
O	1.59307705174554	-2.46850340908569	1.30042342123816
O	1.64328870268420	-1.86602057340892	-1.15445559995028

***cis-trans-C-mer* [Ga(AB*)]**

Ga	-0.13867124509648	0.93345454620158	-0.38785653272844
C	-2.14321823529653	1.20181665334630	-2.23461607914463
C	-3.86879596217207	-0.75558767336997	1.88756065725819
H	-3.44672508512815	-0.50832986319917	2.86592411244442
N	-2.93141453110962	-1.60059354440760	1.16767257041656
H	-3.26876340986185	-2.54773572207869	1.04894848200267
C	-5.13941796073591	-1.59909734848751	2.16372861936285
O	-6.03770995078486	-1.03173801648271	2.81487715141281
C	-1.74787935110660	-1.24555526437579	0.68187532947028
O	-1.37009383348707	-0.04184079628443	0.82538207128646
N	-2.45058170700492	2.07236325625929	-1.30779127438391
C	-3.75498961203221	2.68758990430631	-1.06874925921088
H	-3.95057060407992	3.43318857796067	-1.84049106249967
H	-3.62111240937474	3.21723135032330	-0.13112077272155
C	-0.94193390184751	-2.23332828697105	0.02687668928480
H	-1.30664332465555	-3.24902941512660	-0.00642384689945
C	0.25956277537361	-1.93661050175555	-0.55074567558993
C	1.01679547484739	-3.02958797385579	-1.26528863533137
H	0.75664885807221	-2.96204324315269	-2.32497379078585
H	0.68177743831263	-4.01076219201947	-0.93004264198317
C	2.53647478032768	-3.02865530705677	-1.23756896088386
O	3.13914986813270	-3.79560952052597	-1.99147986348937
N	3.14887226716439	-2.21066024890885	-0.38692667454719
H	2.59447070190536	-1.55711113866439	0.14430530680546
C	4.97960962712945	-1.30540654513278	0.95553520167129
O	6.20794429597471	-1.21710922373853	1.16071286902650
N	2.30816991708585	2.15349916205123	-0.35782931079407
C	2.01211338063565	1.98998708920376	0.90607782151029
C	2.99985638390675	2.23774426683520	1.99013105008059
H	2.46458852540020	2.61021308170701	2.86068393263931
H	3.47174072160356	1.28661163155686	2.24575192240761
H	3.77215951058853	2.94635843058016	1.70646490974443
C	3.61196558209735	2.50481044760475	-0.91216833560196
H	3.40961071029525	2.84052691597170	-1.92693824271642
H	4.00884952616049	3.35202967500404	-0.35625483337322
C	4.59885105009024	1.34620318503654	-0.89868652499409
H	5.56951719806201	1.73139539161864	-1.22127841905664
H	4.72459703545558	1.01374613960050	0.13034235929684
C	4.18134344606646	0.18399467419766	-1.79263169849593
H	3.12416764647900	-0.03458074812814	-1.63811253081187
H	4.27201461371276	0.50152301098363	-2.83448305320796
C	4.98749207290356	-1.09933263400858	-1.59745621089461
H	4.86579281331849	-1.72401424207067	-2.48288484106114
H	6.05243217033905	-0.87821578935204	-1.50793743530492
C	4.57300505135086	-1.95163082410166	-0.38365696319060
H	5.10073132769066	-2.90250624841326	-0.45004760624812
C	-4.93799586295068	1.71601374579111	-0.97289963735945
H	-5.73655452296318	2.26335226350700	-0.46620595349392
H	-5.31312941162180	1.49379127356983	-1.97142120254520
C	-3.04587204862320	0.92323195920556	-3.38553775578888
H	-3.74998018080405	1.72845344117899	-3.57136891906983
H	-2.42195460216958	0.77985295617390	-4.26547955798728
C	-4.67703865502935	0.40029821542986	-0.24770065430009
H	-3.93982178880536	-0.18280544512184	-0.80170736309035
H	-5.59932699957562	-0.18561676618471	-0.27516364981791

C	-4.21316982983904	0.56335418209787	1.18879241746153
H	-3.34222199335905	1.21164212222841	1.22276998990347
H	-4.99965744285093	1.03136777613685	1.78130803317706
O	-5.15881209788708	-2.77076753697069	1.72286115449750
O	4.06515035652784	-0.90888276107553	1.71241482375824
O	0.80216728775189	-0.76518453082731	-0.60433659294889
O	-1.03036836903652	0.59148831761828	-2.14952271191044
O	-1.49385541950059	2.35106480700847	-0.37577669763210
O	1.33179842330250	1.88327883824832	-1.26857215567304
O	0.84516212648001	1.57057456758959	1.20725039237926
H	-3.59816261575469	-0.00073250428443	-3.21210393973021

***cis-trans-N-mer* [Ga(AB*)]**

Ga	-0.24327733274029	1.06811498027723	-0.91482759499970
N	-2.69731135653476	1.98363412211663	-1.70831778971048
C	-3.66014754222639	-0.99676848210666	1.53951004748785
H	-3.26230030065931	-0.63933461420353	2.49310568189716
N	-2.63031334300437	-1.73350632136368	0.83162777616150
H	-2.87144524187659	-2.70955368905670	0.70586709875299
C	-4.79334724273178	-1.99691458433804	1.88524862601052
O	-5.75531988000843	-1.52983991711856	2.52431067164993
C	-1.55102235504603	-1.23881629466750	0.24346633968329
O	-1.28029333172951	-0.00575807098008	0.38443273807947
C	-2.36207378630129	2.80586362161724	-0.74516202336844
C	-3.31080818102695	3.83066890098549	-0.22785794122396
H	-4.15994762204347	3.99578219977472	-0.88264371232131
H	-3.67444816354281	3.51976838850718	0.75199500190068
C	-0.72894828548942	-2.14197391729335	-0.51453704766230
H	-0.89973878508873	-3.19964311277253	-0.38159327674837
C	0.21416819724436	-1.74289074402217	-1.41424698229708
C	1.08398961937568	-2.77901033390367	-2.06967436658564
H	1.16181097083323	-2.56347183443143	-3.13513902275721
H	0.65908799144551	-3.77676817873980	-1.96000703262481
C	2.51168000471938	-2.84249484782419	-1.54214693270648
O	3.36525245434162	-3.45686655950298	-2.18160934179923
N	2.75930735439423	-2.22966855733499	-0.38697869193721
H	2.00874003536292	-1.82408164592347	0.15600305187171
C	3.85615912857328	-1.62985580315385	1.70219452292700
O	4.89721110700961	-1.42545375202423	2.35743188905222
C	2.14116594727218	2.30958465638484	-1.43337509299768
N	2.31130677402791	1.77067616431058	-0.25168374424572
C	3.52760695361065	1.64432891252868	0.55272415236065
H	3.71898112853446	2.59380193258901	1.05654436480466
H	3.24843960021510	0.91879286056504	1.31072861414818
C	3.23394187829326	3.04460701080774	-2.13157455943475
H	3.84291827816095	2.35439519475486	-2.71571750006374
H	2.77133028643214	3.75247569663700	-2.81436447716874
C	4.79011789292446	1.18789263556006	-0.17449348349933
H	5.20168978521728	2.02487609026127	-0.73340523977334
H	5.51781103118389	0.97414167681811	0.61031359161399
C	4.67039283210011	-0.02127991874291	-1.11146934299065
H	3.65813875526490	-0.08818307126587	-1.51522226569026
H	5.31183963410254	0.17414024603756	-1.97194538357525
C	5.08817876303073	-1.38371512699765	-0.55643855447811
H	5.36036256183718	-2.01451581001713	-1.40169918854153
H	5.97849790870672	-1.27958040030465	0.06360335694691
C	4.05168255143049	-2.16029634919547	0.26279454740866
H	4.44020223945690	-3.17636131236274	0.38057381911749
C	-5.07720225676509	1.19673637178979	-1.39992123150129
H	-5.42359464837665	1.94299855482496	-0.68236021311901
H	-5.93051926260524	0.98398964438761	-2.04635699005891
C	-4.01618891184830	1.80887896823256	-2.31144945389829
H	-4.36063954089616	2.76740716051104	-2.69862862370434
H	-3.83451342191218	1.15833186429344	-3.16248908440613
C	-4.65433637455122	-0.07260067238431	-0.65017967953358
H	-3.86505687995720	-0.57928962629264	-1.20818726197611
H	-5.49709875902209	-0.76604103345589	-0.61352979270352
C	-4.19127662149268	0.21611471458818	0.77324331416423

H	-3.41989252563672	0.97942271819659	0.76506679950279
H	-5.02696902844069	0.60988049489644	1.35158280629147
O	-4.64747833487346	-3.18124093668580	1.50592384191161
O	2.67983383426402	-1.47566807323597	2.10023917225466
O	0.47353758118988	-0.52777607105512	-1.77046760406827
O	-1.74244297253066	1.11468265909690	-2.15192630374040
O	-1.20100433997085	2.71479786473112	-0.23908730964487
O	1.00160598033270	2.21348612157419	-1.99249849560467
O	1.21241021366022	1.22455945872562	0.34494812804380
H	3.87787259111461	3.57931136670652	-1.43901388307700
H	-2.76319523673382	4.76297640966935	-0.10139343780566

***cis-cis-C-fac* [Fe(AB)]$^{1-}$**

Fe	-0.10650307986481	1.41264199748543	0.35390837247462
N	-2.44673552816560	2.55381435317699	-0.75233926673827
C	-4.30652799347171	-2.28695727473784	-0.18533766962074
H	-4.81191309803120	-2.89466030740297	0.56959615538459
N	-2.88687386524952	-2.27585570733238	0.11712699740320
H	-2.30653212942759	-2.64631824016261	-0.62146880144952
C	-4.52498157840164	-2.98365470085113	-1.54497774438683
O	-5.71516399324433	-3.17085305144636	-1.87292228599807
C	-2.37685098861201	-2.04642058916734	1.33186134236132
O	-3.07485826596654	-1.66389048320944	2.27376431132425
C	-1.98969207914608	3.53948581182896	-0.01123295526141
C	-2.71522174909979	4.84692022824654	0.05818349019099
H	-2.79119502514031	5.30898266270972	-0.92545713643008
H	-3.72319837419398	4.71717298109122	0.45082494189396
H	-2.15999192892866	5.50651475399678	0.71833342637859
C	-0.90612334304021	-2.35077209241373	1.51760876386149
H	-0.54055819245911	-2.98473378861352	0.71346800572484
H	-0.83271335741279	-2.90932667161199	2.45018670651110
C	0.03981235601744	-1.13668198162275	1.59856167840817
C	-0.42289901633794	-0.15441552575939	2.70261995701632
C	1.45538574110602	-1.60554655690970	2.02123927886504
H	1.38196507761013	-2.29707537392149	2.85964695482332
H	2.01651392222334	-0.73388159531364	2.35666731403692
C	2.21055103527522	-2.28520655016027	0.90398059860593
O	1.83577770682671	-3.34819424950667	0.40394316023161
N	3.32308086032404	-1.66047611245203	0.51066852777370
H	3.65856060028436	-0.85253880011832	1.01318284321349
C	5.61090511263685	-1.48334990238541	-0.30392393931899
O	6.51198362431748	-1.86648115138542	-1.07640415980769
N	2.29848376766971	2.02984187291714	-1.00662518972535
C	2.62633151981260	2.18356797499080	0.25500464034194
C	4.03904419872003	2.44393487974801	0.65944045066070
H	4.02649127566370	3.02558970256848	1.57813289711793
H	4.54641004367752	1.49671903362446	0.85349513831446
H	4.60041792105164	2.98004568779229	-0.10136752674073
C	3.20649496470593	1.91806947085860	-2.14113197778270
H	2.57926370780616	2.04612757505622	-3.02054013410118
H	3.91477676375133	2.74485419641762	-2.11472004639354
C	3.94057143236388	0.58481656246316	-2.17106322634234
H	4.59963926527236	0.57908916669771	-3.04319267426047
H	4.58720436550988	0.53525406244792	-1.29657269875173
C	3.00633288670362	-0.61768555723135	-2.20607408240520
H	2.20765513611297	-0.47449187234511	-1.47810974201400
H	2.52008719836089	-0.66274232571706	-3.18429594103700
C	3.67662388033697	-1.95867503898810	-1.93474221141657
H	2.94412388810359	-2.74625819167496	-2.11444490663868
H	4.50332232081041	-2.12732493086351	-2.62593400492667
C	4.22729349771407	-2.14103051703468	-0.50884303435276
H	4.38237975075711	-3.21145757310221	-0.35775918785891
C	-4.68581778199811	1.51852189687531	-0.91579096325870
H	-4.71159805993290	1.68341061195945	0.16457019104818
H	-5.67848524446167	1.77679079312793	-1.29098329712737
C	-3.68319495654703	2.50276062563983	-1.51053768198257
H	-4.09826831088367	3.50608621068806	-1.53011085654159
H	-3.42845419228664	2.22582039825664	-2.53571721020386

C	-4.37515842273118	0.05026870109435	-1.20778602080318
H	-3.29653445943418	-0.07264593477147	-1.23297612764697
H	-4.75182480531204	-0.21429025307079	-2.19947329798832
C	-4.93951671719716	-0.89451693671540	-0.16013275524733
H	-4.76391169423555	-0.47593248092278	0.83086027835544
H	-6.01800127703313	-1.01115688219064	-0.27558258306729
O	-3.50874942756330	-3.29929903988887	-2.20437064588778
O	5.72161693490209	-0.63759013801162	0.61380565686781
O	0.11177682639898	-0.48004377315281	0.38088380955912
O	-0.67916030598141	1.03011729395540	2.29234963933126
O	-1.74081808926739	1.39634728654218	-0.72964178012749
O	-0.92331857520649	3.35941780200832	0.63921386232712
O	0.98667250869438	1.79871333637899	-1.27804409705105
O	-0.47786627613619	-0.51538678152555	3.87681278829658
O	1.72016209088042	2.09290100304679	1.13484568198897

***cis-cis-N-fac* [Fe(AB)]$^{1-}$**

Fe	0.15743830782097	1.47203425929643	0.74030398952198
C	-2.67588857951236	1.98183439900705	1.48312339264779
C	-2.96966982756855	-1.51088268845618	-1.29483197354743
H	-3.55876003030787	-2.42099512108903	-1.16887237310531
N	-2.02219756995178	-1.43094981536059	-0.20306736900375
H	-1.06435247536543	-1.18176608003370	-0.41879623167361
C	-2.21745354114929	-1.63471522623728	-2.63524499763413
O	-2.92772717613613	-1.89480001138294	-3.63104693452649
C	-2.30804443924914	-1.71105636041430	1.06528728671357
O	-3.45518270899807	-1.97585967073682	1.44839748087198
N	-2.41493271199761	2.81132714964892	0.48842639065159
C	-3.38169556408532	3.32453498131475	-0.47876359105035
H	-2.78648416010649	3.90411162187561	-1.17893191493764
H	-4.06959910006579	4.00901027937543	0.01671386455700
C	-1.16447981214457	-1.86181089363668	2.05385533966299
H	-0.94884554006425	-2.93083885686881	2.01660038005096
H	-1.58095662455374	-1.66685855228714	3.03867916949416
C	0.17474412457655	-1.08592866100925	1.90675958339530
C	0.35655926178776	-0.06238368307450	3.05877557282793
C	1.37325217060102	-2.05718077509713	2.08918919173962
H	1.14512743182343	-2.77672104334529	2.87509129283141
H	2.24708100295494	-1.48733234261562	2.40424053427895
C	1.69647792070355	-2.79587370937645	0.81170633045224
O	0.89369955524121	-3.55644716887753	0.26868666644239
N	2.91667288878120	-2.55570176123620	0.32462020203618
H	3.56135901631170	-1.97788583374186	0.84149703567233
C	4.97880408020922	-3.00920460192700	-0.89537729411163
O	5.55776376975750	-3.57690921491593	-1.84512851937667
C	2.56488109567860	2.48694593356295	-0.35295608286075
N	2.01332746431276	1.88182603563336	-1.37298094355003
C	2.59245937769345	1.61079195242077	-2.67798944209833
H	3.62104791045276	1.96252884588616	-2.67063895979118
H	2.03707464432338	2.19224014332218	-3.41647022096633
C	3.95095476959805	3.04227367541295	-0.42532886530771
H	4.15148938952350	3.57479542597459	0.49966174566427
H	4.06657958363187	3.72581662900390	-1.26478182214764
C	2.52679892633885	0.12790108200719	-3.01541586579694
H	1.47992281528061	-0.16932280219741	-3.07488418904597
H	2.95471972215166	-0.00604777111456	-4.01139217190659
C	3.24811645723372	-0.74030358633278	-1.99704047488769
H	4.32832419158372	-0.59477181683336	-2.08687977502917
H	2.97118562282865	-0.40229942347703	-0.99734109247608
C	2.90410650212212	-2.21815938808155	-2.11010449600132
H	1.81891878563362	-2.33150647830096	-2.14154754369615
H	3.30124559079475	-2.63993830593763	-3.03456015160130
C	3.43659657147711	-3.04506251561884	-0.93264289429216
H	3.12883693413633	-4.08223884144023	-1.06384136215541
C	-4.15020936458932	2.20742855672466	-1.18724236473812
H	-4.66382335750550	2.64461707180917	-2.04536028166570
H	-4.93482678264609	1.85435922741494	-0.51672511253795
C	-4.04238105131384	1.90182982647473	2.07967501161660
H	-3.94124705511415	1.98435352471453	3.16098589168919
H	-4.71217550427571	2.67800776952745	1.72268248170072
C	-3.28143192667752	1.01437002349279	-1.60679124072777
H	-2.33715853590230	1.04580215249537	-1.06571954275779

H	-3.02142063932027	1.08761717284547	-2.66433825018665
C	-3.94396522518448	-0.32879829076607	-1.32277637581400
H	-4.43069825720549	-0.29074948993765	-0.34770213101957
H	-4.72030484311113	-0.53830389307006	-2.06019927430537
O	-0.98027851887550	-1.45302635216835	-2.63073918622387
O	5.52115879702245	-2.40271601451716	0.05592062954900
O	0.30761419708189	-0.42707514677439	0.69323656941986
O	0.75105307607039	1.09492212655547	2.68598642100854
O	-1.74066995294011	1.28240291877187	1.93084398228176
O	-1.12804364847104	2.80935009022729	0.04903953541059
O	1.89066612676295	2.58257356654247	0.72181689967390
O	0.19849706216544	-0.40778543153797	4.22989541561630
O	0.75364776625760	1.43241764257602	-1.20344363775769
H	-4.47262965324648	0.92524105499538	1.85893144483764
H	4.68170126691005	2.24076248091637	-0.53563478200446

***cis-trans-C-mer* [Fe(AB)]$^{1-}$**

Fe	0.15743830782097	1.47203425929643	0.74030398952198
C	-2.67588857951236	1.98183439900705	1.48312339264779
C	-2.96966982756855	-1.51088268845618	-1.29483197354743
H	-3.55876003030787	-2.42099512108903	-1.16887237310531
N	-2.02219756995178	-1.43094981536059	-0.20306736900375
H	-1.06435247536543	-1.18176608003370	-0.41879623167361
C	-2.21745354114929	-1.63471522623728	-2.63524499763413
O	-2.92772717613613	-1.89480001138294	-3.63104693452649
C	-2.30804443924914	-1.71105636041430	1.06528728671357
O	-3.45518270899807	-1.97585967073682	1.44839748087198
N	-2.41493271199761	2.81132714964892	0.48842639065159
C	-3.38169556408532	3.32453498131475	-0.47876359105035
H	-2.78648416010649	3.90411162187561	-1.17893191493764
H	-4.06959910006579	4.00901027937543	0.01671386455700
C	-1.16447981214457	-1.86181089363668	2.05385533966299
H	-0.94884554006425	-2.93083885686881	2.01660038005096
H	-1.58095662455374	-1.66685855228714	3.03867916949416
C	0.17474412457655	-1.08592866100925	1.90675958339530
C	0.35655926178776	-0.06238368307450	3.05877557282793
C	1.37325217060102	-2.05718077509713	2.08918919173962
H	1.14512743182343	-2.77672104334529	2.87509129283141
H	2.24708100295494	-1.48733234261562	2.40424053427895
C	1.69647792070355	-2.79587370937645	0.81170633045224
O	0.89369955524121	-3.55644716887753	0.26868666644239
N	2.91667288878120	-2.55570176123620	0.32462020203618
H	3.56135901631170	-1.97788583374186	0.84149703567233
C	4.97880408020922	-3.00920460192700	-0.89537729411163
O	5.55776376975750	-3.57690921491593	-1.84512851937667
C	2.56488109567860	2.48694593356295	-0.35295608286075
N	2.01332746431276	1.88182603563336	-1.37298094355003
C	2.59245937769345	1.61079195242077	-2.67798944209833
H	3.62104791045276	1.96252884588616	-2.67063895979118
H	2.03707464432338	2.19224014332218	-3.41647022096633
C	3.95095476959805	3.04227367541295	-0.42532886530771
H	4.15148938952350	3.57479542597459	0.49966174566427
H	4.06657958363187	3.72581662900390	-1.26478182214764
C	2.52679892633885	0.12790108200719	-3.01541586579694
H	1.47992281528061	-0.16932280219741	-3.07488418904597
H	2.95471972215166	-0.00604777111456	-4.01139217190659
C	3.24811645723372	-0.74030358633278	-1.99704047488769
H	4.32832419158372	-0.59477181683336	-2.08687977502917
H	2.97118562282865	-0.40229942347703	-0.99734109247608
C	2.90410650212212	-2.21815938808155	-2.11010449600132
H	1.81891878563362	-2.33150647830096	-2.14154754369615
H	3.30124559079475	-2.63993830593763	-3.03456015160130
C	3.43659657147711	-3.04506251561884	-0.93264289429216
H	3.12883693413633	-4.08223884144023	-1.06384136215541
C	-4.15020936458932	2.20742855672466	-1.18724236473812
H	-4.66382335750550	2.64461707180917	-2.04536028166570
H	-4.93482678264609	1.85435922741494	-0.51672511253795
C	-4.04238105131384	1.90182982647473	2.07967501161660
H	-3.94124705511415	1.98435352471453	3.16098589168919
H	-4.71217550427571	2.67800776952745	1.72268248170072
C	-3.28143192667752	1.01437002349279	-1.60679124072777
H	-2.33715853590230	1.04580215249537	-1.06571954275779

H	-3.02142063932027	1.08761717284547	-2.66433825018665
C	-3.94396522518448	-0.32879829076607	-1.32277637581400
H	-4.43069825720549	-0.29074948993765	-0.34770213101957
H	-4.72030484311113	-0.53830389307006	-2.06019927430537
O	-0.98027851887550	-1.45302635216835	-2.63073918622387
O	5.52115879702245	-2.40271601451716	0.05592062954900
O	0.30761419708189	-0.42707514677439	0.69323656941986
O	0.75105307607039	1.09492212655547	2.68598642100854
O	-1.74066995294011	1.28240291877187	1.93084398228176
O	-1.12804364847104	2.80935009022729	0.04903953541059
O	1.89066612676295	2.58257356654247	0.72181689967390
O	0.19849706216544	-0.40778543153797	4.22989541561630
O	0.75364776625760	1.43241764257602	-1.20344363775769
H	-4.47262965324648	0.92524105499538	1.85893144483764
H	4.68170126691005	2.24076248091637	-0.53563478200446

***cis-trans-N-mer* [Fe(AB)]$^{1-}$**

Fe	-0.19935666882107	1.29568312247235	0.87828718622570
N	-2.65551401857997	2.59957211626416	0.73720573485465
C	-4.09826376498518	-1.77291603461102	-0.73169701342997
H	-4.77694141891688	-2.39080493382306	-0.14027542934269
N	-2.74580001004935	-2.02406656503908	-0.27582976897122
H	-2.10021417237206	-2.25757318614193	-1.01561859804284
C	-4.22272529891354	-2.23174750706571	-2.19984773360017
O	-5.37870306981541	-2.23704562910296	-2.67238692878284
C	-2.37229831493844	-2.14221090141247	1.00122501945972
O	-3.14468586165339	-1.93531145292133	1.94172308238751
C	-1.87091117012921	3.58461202379007	1.11970796963126
C	-2.34196841493695	5.00408250942858	1.04926075607584
H	-3.20732201849442	5.16630792726462	1.69133775281667
H	-1.53111629814952	5.64850892759332	1.37532789742769
H	-2.62139385536343	5.27322398124000	0.03110582269580
C	-0.94747339811802	-2.59407474781810	1.23494527400147
H	-0.59486751808239	-3.17093917844364	0.38290192745337
H	-0.95256924552205	-3.23640810644453	2.11363205339622
C	0.06747435794282	-1.45869458310114	1.45558487871882
C	-0.15249462527729	-0.73408427142327	2.81080901497506
C	1.48694497597543	-2.05456698144870	1.61989388123256
H	1.43698044749732	-2.89757742991341	2.31217533039440
H	2.11830094541245	-1.30045777456663	2.09087187528011
C	2.19456736236927	-2.53817712033582	0.36968529007190
O	1.63696689829201	-3.14249383159055	-0.54566397341295
N	3.50869481345376	-2.28458574171837	0.37484522636178
H	3.93851458801086	-1.85543388670395	1.18252239843029
C	5.86843464486921	-2.76943590587780	0.07842398400921
O	6.78338486246014	-3.31346300676104	-0.57224838862219
N	1.96910688441440	2.10798690997039	-0.74198858630588
C	0.93349473634399	2.20717199662685	-1.55129991260099
C	1.10973866689810	2.46734572337319	-3.01053789288235
H	2.10693074788658	2.80399018803197	-3.27386454794616
H	0.88521660248905	1.55436724090629	-3.56316707286724
H	0.38387795527379	3.22245158513394	-3.30759666308457
C	3.31225469713233	1.71643703214248	-1.15721095984135
H	3.95290377345122	1.89083344577858	-0.29547266615126
H	3.65475278858865	2.36079450642391	-1.96434152453900
C	3.29761785014116	0.24848073979326	-1.55370192323912
H	2.70290382336941	-0.27349587599336	-0.80902531488757
H	2.76841894236683	0.14396376688121	-2.50424499485761
C	4.65683372090402	-0.42143514720022	-1.66958339237071
H	5.21313502172955	0.02117041987939	-2.49896062970194
H	5.24316512607675	-0.22758335935439	-0.76693765221546
C	4.51743969534609	-1.93159194674134	-1.88705527946425
H	3.61273731144395	-2.12906829129925	-2.46552686947060
H	5.35465877257547	-2.31578493158315	-2.46802311747769
C	4.47424889634961	-2.75786275203912	-0.59411100646866
H	4.23283948351262	-3.78855833301993	-0.85966788432270
C	-4.16058711312268	2.13644934948830	-1.19806918252868
H	-3.57054248543245	2.77295765842605	-1.86184115366226
H	-5.20898159112971	2.29488725095935	-1.46795923164886
C	-4.01068627724739	2.65716289629081	0.22916720101227
H	-4.63544612188919	2.08510841697805	0.91663359318749
H	-4.34247950688631	3.69108550488236	0.27051625309140

C	-3.79543877214452	0.67296951255156	-1.45316354272514
H	-2.72463250657906	0.53975822512438	-1.31832886473816
H	-4.01836636367980	0.45679432662369	-2.50171527587526
C	-4.53937737381379	-0.31460034051583	-0.56748722244839
H	-4.39999951802461	-0.05372971615128	0.47920472060705
H	-5.61037869952970	-0.26669955195049	-0.77613434550164
O	-3.16966731641336	-2.54481270943192	-2.80017888815692
O	5.97189289282674	-2.22792070028502	1.20265597811191
O	0.01414328589229	-0.53933693805047	0.42286402593396
O	-0.05795882329188	0.54614652081103	2.75230024728607
O	-2.15931553685692	1.34766030340243	0.86609412706516
O	-0.71092546651249	3.30397507566995	1.53291959035475
O	1.70200576433445	1.85532526061700	0.57108949637787
O	-0.31932550213341	-1.36880949260587	3.84764087953792
O	-0.22898321782443	2.07682439766663	-1.07906503628057

***cis-cis-C-fac* [Fe(AB*)]**

Fe	-0.25502448990165	0.99428141674318	0.05869602645503
N	-2.33495156065567	1.62267037074461	-1.72417682211930
C	-4.32004889380717	-0.79153850214325	1.43772321470797
H	-4.20896243722654	-0.26953695921979	2.39045884469019
N	-3.16613432019246	-1.63781442605002	1.20862315592934
H	-3.41311557311541	-2.60761724862950	1.05642785381273
C	-5.56514267556550	-1.70061702516306	1.56452502593989
O	-6.62678664473229	-1.12486551056981	1.87363014448458
C	-1.91338461590367	-1.23397741229513	1.02512850425562
O	-1.61734590659444	-0.01373916883797	1.21894209742495
C	-2.36593777090187	2.62981740731861	-0.88121765879842
C	-3.39934041477071	3.69787081147488	-0.99253290830544
H	-3.87892017525438	3.72818199200810	-1.96579908490303
H	-4.16195561496965	3.53825127681120	-0.22934805759105
H	-2.91900907130319	4.65390824845952	-0.79363193382547
C	-0.94703615041478	-2.22371858207058	0.63428774994355
H	-1.23446201555713	-3.26261597756262	0.69987956795567
C	0.30514158590728	-1.90808996895578	0.19703870809029
C	1.32446626120962	-3.00862025854203	0.00236172901456
H	0.82437806609246	-3.95531886386826	-0.19230957728775
H	1.87601775132155	-3.10720143331527	0.93974942245305
C	2.27725726974816	-2.71844227757085	-1.13889047029685
O	1.93288690330404	-2.88635171388052	-2.30943630014143
N	3.48169766059769	-2.28141546077193	-0.77850284618017
H	3.73029308423988	-2.20984903486805	0.19878571604256
C	5.84624854372456	-1.73323943034706	-0.80486798499190
O	6.88871667539732	-1.45772873668175	-1.43043202030552
N	2.28748762985252	2.09936476762673	0.17552152105212
C	1.99708519250884	1.92280140921605	1.44506357498274
C	3.04828835678869	2.07847711602974	2.49544893212002
H	2.58984426537708	1.90845215749786	3.46491029902548
H	3.85078573448123	1.35573419984602	2.34979301976589
H	3.48361634695980	3.07689258133472	2.47379889879201
C	3.59187846939819	2.36827492256840	-0.40463476426696
H	3.41988384459744	3.06914384556384	-1.22162109547807
H	4.19765793053938	2.87198367297874	0.34468928664507
C	4.30027949346517	1.11619295266348	-0.90388835957826
H	5.29107522353987	1.42192709784596	-1.25148387902731
H	4.45873249457148	0.44514550142858	-0.05792803373743
C	3.55835210224196	0.39652257280748	-2.02081143679512
H	2.58569478663075	0.07623458089166	-1.65189945444050
H	3.36082413099150	1.11091739843367	-2.82492522812828
C	4.28893514335772	-0.79476313811182	-2.62349302648869
H	3.68694064780160	-1.17221436656570	-3.44949848073209
H	5.24805593346014	-0.48639986037081	-3.04067712670448
C	4.57855895394866	-1.96004525430352	-1.66701130760135
H	4.79960175121587	-2.83615647302465	-2.28335413852580
C	-4.67658396874480	0.79168964411072	-2.14713340483869
H	-5.23549518100891	1.64332850941537	-1.75384756329139
H	-5.24822773182495	0.42527702893747	-3.00193226482395
C	-3.34288813762054	1.26721336872945	-2.71888639876612
H	-3.49946945893477	2.11497874030768	-3.38492404788429
H	-2.87856068724581	0.47459943143585	-3.29960575356298
C	-4.58855712932027	-0.29895248345497	-1.07432132686638
H	-3.72582144136284	-0.93719974749393	-1.27457891322295

H	-5.46969300185708	-0.93959285834977	-1.15064561628769
C	-4.51042768236194	0.26086560455423	0.34241671087284
H	-3.69390748229316	0.97115979709172	0.42245929907903
H	-5.42965526346629	0.80156104969942	0.56956674436663
O	-5.41002092809865	-2.92290142526566	1.34343400758203
O	5.73168278028572	-1.86366875847379	0.43548566712800
O	0.73784420832088	-0.71775951331184	-0.03946223122802
O	-1.33336410564586	0.71559826368510	-1.56095782137889
O	-1.50492352542475	2.67751570294628	0.04370011154875
O	1.26937769274047	1.96299964175548	-0.71158729135560
O	0.80984714145966	1.60910878710791	1.75727879559706

***cis-cis-N-fac* [Fe(AB*)]**

Fe	0.37313873711039	-1.36119470945661	0.03924333044008
N	-2.04070707210854	-2.11905733015092	1.25326911205685
C	-4.33090854606847	2.30690402212633	-0.42108226293691
H	-5.04689488571394	2.68377575628791	-1.15379389894420
N	-3.02286797190888	2.22203879923870	-1.03578984182158
H	-2.29264764688022	2.67628014073774	-0.50303252407963
C	-4.26548676264481	3.33624076311516	0.72932143869064
O	-5.35445176072038	3.61391218549741	1.27061775579931
C	-2.74319363439409	1.61901047925966	-2.18984655359023
O	-3.59278582489694	1.08737734937440	-2.90271411972178
C	-1.93754246303427	-2.96004585736940	0.24926892910733
C	-2.99185148229434	-3.97321648889642	-0.03786211002195
H	-3.70979437092768	-4.09239729257202	0.76616171713562
H	-3.52362681808161	-3.67795585156341	-0.94310008635373
H	-2.50287384580785	-4.92623868102258	-0.23223542426526
C	-1.27555049138970	1.65186112118106	-2.59210154741208
H	-1.00051008178760	2.68153527833063	-2.81923357815602
H	-1.17407724038563	1.05293729431414	-3.49444374913362
C	-0.37180393121590	1.08391034279883	-1.52321286490864
C	0.43968867666800	1.93164230705495	-0.81775248054645
H	0.43713822485829	2.98024809069103	-1.07445404573177
C	1.37758284564935	1.47880912717297	0.16350687284878
O	1.44399762919192	0.27594899384645	0.53585011792637
N	2.19959417893299	2.38503040751980	0.71523316660343
H	2.10056183391431	3.30724057644109	0.32153928646506
C	4.56359987427868	1.68722797274618	0.24003510558744
O	5.75694972449836	1.80056323634613	0.59945061310534
N	2.85242333220730	-2.40233859125052	0.71434917910661
C	2.95065680003764	-2.24168034673724	-0.58812943375730
C	4.27257934692270	-2.25141994019589	-1.27564690199043
H	4.13466165145405	-2.65723589572022	-2.27520859355025
H	4.61695929665062	-1.21915751900080	-1.36642265199278
H	5.02559047746494	-2.82900624715223	-0.74813147701735
C	3.93815186035946	-2.38585473514319	1.68794422959570
H	3.54148587038572	-2.87177218630503	2.57768459288059
H	4.75281379025518	-2.99991632545271	1.31055791536223
C	4.42666787831117	-0.97247220024184	1.98325561533653
H	5.34262264371583	-1.04665690900587	2.57523866696459
H	4.71411144097439	-0.52157236859011	1.03713374054861
C	3.39630667540960	-0.10942223599930	2.71132009040554
H	2.40793593183251	-0.35052248328038	2.34275266535750
H	3.41331598730693	-0.38124810964607	3.77110992816720
C	3.60537579232086	1.40304167677146	2.63472723975757
H	2.88450412149382	1.87734468372341	3.30499829339207
H	4.59347238752818	1.64169848825882	3.03287439336426
C	3.52638378314017	2.15844338786136	1.29451414277206
H	3.85357683332993	3.16695965140540	1.54800863013823
C	-4.29301701986945	-1.11410031299054	1.45840193584434
H	-4.65680000903571	-1.67231674339098	0.59176226301055
H	-5.13139746793370	-1.03960091814382	2.15526837230342
C	-3.19003849266448	-1.91886026574694	2.12891299468653
H	-3.55697413672835	-2.88851091184321	2.45767583258084
H	-2.79840555479384	-1.40054109109146	3.00142917969427
C	-3.83766341086581	0.26809509051318	1.01887060718956
H	-2.87954830577760	0.17818092489923	0.50786315794995

H	-3.65734482229184	0.88958210228971	1.90083043613480
C	-4.82421895788658	0.94825628363068	0.08522784532357
H	-5.00348500246968	0.30207754722537	-0.77564301609183
H	-5.78387162809021	1.10367918727952	0.57980639276711
O	-3.13988899736955	3.79533753482061	1.02828774105356
O	4.15249657313535	1.26519068573359	-0.85862335994227
O	-0.45679618990190	-0.19073938609194	-1.37609137307905
O	-1.02955255215058	-1.22375739838322	1.40901174427858
O	-0.91198322389990	-2.89755347836345	-0.49139730518851
O	1.59748165140190	-2.35512212922838	1.23537816000381
O	1.89179475124946	-2.03839654846624	-1.25573423150285

***cis-trans-C-mer* [Fe(AB*)]**

Fe	-0.12536944449812	0.99271449002152	-0.44034313143103
C	-2.19226679929950	1.28896088807164	-2.34228439237228
C	-3.82098959865421	-0.75675414267808	1.93723169207220
H	-3.39443555153958	-0.47996679743846	2.90541038728290
N	-2.88554192156590	-1.61373447695394	1.23113601862076
H	-3.23093438248483	-2.55747482163462	1.10973367728159
C	-5.08647975938682	-1.59776411847029	2.24163943749192
O	-5.97444697327856	-1.02434242556394	2.90175390457454
C	-1.71189150661134	-1.25743853497423	0.71922065376417
O	-1.32490690698322	-0.05967623163351	0.85521164302779
N	-2.53061693865703	2.10043686208173	-1.36954963019730
C	-3.85481848429668	2.65353453037844	-1.09273679333616
H	-4.10803498714426	3.38678594626089	-1.85924681520076
H	-3.72249745393093	3.18992817106602	-0.15847129348133
C	-0.92892451612243	-2.25421085357922	0.04214626059089
H	-1.27545058552559	-3.27666763979453	0.06623136223682
C	0.22523014823173	-1.95643720828849	-0.62203192578367
C	0.97804014403127	-3.05591191508273	-1.33040647453984
H	0.72895154145179	-2.99354673333155	-2.39269220220663
H	0.64361852646388	-4.03546000118698	-0.98960831186348
C	2.49771252020090	-3.04609520090869	-1.28126383703856
O	3.11940695210989	-3.80069926478311	-2.03202629032554
N	3.08974727573622	-2.23335953269662	-0.41113364185885
H	2.52823972813004	-1.59581118559939	0.13163976932490
C	4.87320305599953	-1.33269816980091	0.99131317756588
O	6.09299800793093	-1.25196401772787	1.24492908027662
N	2.39874783936330	2.21047524004748	-0.34061870695624
C	2.08547956688747	2.05356020096762	0.92298421748210
C	3.06411435916998	2.30094748459258	2.01603055150285
H	2.52703285910083	2.70737495306929	2.87026778826713
H	3.50167661043901	1.34162604395349	2.29923180826798
H	3.86098970696565	2.98029359721387	1.72902613853655
C	3.71312336531445	2.52586572688368	-0.89061883612415
H	3.52413784498038	2.86610901140918	-1.90662973836707
H	4.13270294302171	3.36143807048461	-0.33380258664425
C	4.66152609608575	1.33566033814920	-0.87196556017829
H	5.64195196979248	1.68490154322728	-1.20578078879926
H	4.78466395986443	1.00893742217184	0.15954929393573
C	4.19737216555045	0.17989996613533	-1.75113207921387
H	3.13660195156518	-0.00762766625368	-1.58016542957786
H	4.28115908788421	0.48879338223199	-2.79612773684246
C	4.96961587636255	-1.12454237452266	-1.55836759023237
H	4.84808673758532	-1.73801748598965	-2.45143905023454
H	6.03789622487001	-0.92985073846909	-1.44956053087602
C	4.51393256979811	-1.97876610989062	-0.36172878605972
H	5.04178230184366	-2.93070985667720	-0.41149944942923
C	-4.98095573576271	1.62030923269695	-0.96968820492433
H	-5.80481880725588	2.12839090509580	-0.46247598476004
H	-5.35072031921012	1.36351107018324	-1.96221701178320
C	-3.10272200621621	1.02593800664774	-3.49203549922319
H	-3.83530352989278	1.81439913806307	-3.63746005337822
H	-2.49013945062226	0.93033825748396	-4.38609817947192
C	-4.63770635475920	0.33147172228683	-0.23091082970031
H	-3.86864127488211	-0.21111867833637	-0.78388695131962
H	-5.52321481172579	-0.30929493256490	-0.24716246039380

C	-4.17862170340534	0.53926408196871	1.20179796589313
H	-3.31555439062904	1.19912188394522	1.21781712183522
H	-4.97210025446587	1.01413401022698	1.77976844254253
O	-5.11425132361929	-2.77277167590051	1.80995273040177
O	3.93119857077568	-0.92845880093326	1.70947083717805
O	0.72997679190661	-0.77594901981517	-0.75881298705591
O	-1.05831179477135	0.72406659652000	-2.29049866281875
O	-1.57571003092798	2.35268102351955	-0.43383253819790
O	1.42322025423780	1.94140191577796	-1.24639540146230
O	0.91485139937473	1.64472504566387	1.20451224023959
H	-3.62447135490097	0.08051385298268	-3.33928982653334

***cis-trans-N-mer* [Fe(AB*)]**

Fe	-0.22135629633147	1.03161427846571	-0.92330304323877
N	-2.75070755777213	1.99045018596558	-1.72295875867837
C	-3.65506537148473	-0.99592253906533	1.52938943143672
H	-3.24116230706089	-0.64015979663130	2.47648281244915
N	-2.65027399790659	-1.76673553560319	0.82271155780862
H	-2.92932899251647	-2.72947816679114	0.67562122598958
C	-4.81708967260714	-1.95691109416627	1.88588970070578
O	-5.75915357727438	-1.45591596857500	2.52901290874312
C	-1.53273807583644	-1.30940245567806	0.27777055256302
O	-1.20317781977139	-0.09710602194032	0.45311079465977
C	-2.41368669567369	2.83298857791940	-0.77368428597339
C	-3.36284587052925	3.87739561884249	-0.29380979805483
H	-4.18763086366643	4.05152849809667	-0.97726026427599
H	-3.76463751680022	3.57457871429996	0.67385552183621
C	-0.72730463753164	-2.23302075353492	-0.48072240660229
H	-0.89580439184937	-3.28823697373011	-0.32580155392561
C	0.19311824734998	-1.84430333534292	-1.40633093238610
C	1.07127410812130	-2.87918845345583	-2.05306485603030
H	1.13054498791058	-2.68788597944413	-3.12430360215477
H	0.66582983285292	-3.88146158964684	-1.91374420813383
C	2.50738410798796	-2.90650241949541	-1.54402279812179
O	3.36279989402105	-3.51826826074642	-2.18342636354701
N	2.76029058237729	-2.26786409571975	-0.40364345718370
H	2.00927813618739	-1.86651184905324	0.14169643694724
C	3.87294866290601	-1.63635036165901	1.66966680022178
O	4.91947767541252	-1.41118895203513	2.30952553275523
C	2.21567851912941	2.37637918299671	-1.39537912890320
N	2.37996933298697	1.82746890064629	-0.21537834423419
C	3.60020170811744	1.69986767858239	0.58262969908361
H	3.82722723906030	2.66069740324470	1.04768983450444
H	3.31362051325575	1.00915148200374	1.36971362951980
C	3.29466588719312	3.17689058345671	-2.04122196189328
H	3.89757538366283	2.53906050859538	-2.68829856086741
H	2.82098215974940	3.93619928003766	-2.65872269884731
C	4.83269671796006	1.18268175359724	-0.15930054294593
H	5.27549901322669	2.00464946173655	-0.71746597980822
H	5.55873876642746	0.92472160107583	0.61303256083804
C	4.63669972374822	-0.00889353247927	-1.10862928851937
H	3.59924378306316	-0.05884034484621	-1.44554753838003
H	5.22022942653436	0.19501070683918	-2.00728195451640
C	5.07132360498673	-1.38559469590156	-0.60359799385487
H	5.30967332710948	-1.99893886122898	-1.47168916529912
H	5.98349010107418	-1.29922495375470	-0.01338937506274
C	4.05882436831256	-2.17483620492733	0.23202850298792
H	4.46383768489832	-3.18442469012862	0.34946745068064
C	-5.11723778204521	1.18289556481488	-1.40144224073824
H	-5.46558886217947	1.92885973261339	-0.68408537746465
H	-5.97306046639224	0.95819691619284	-2.04065631848820
C	-4.06881374008024	1.80356636671632	-2.32193012914754
H	-4.42451876910144	2.75868014129311	-2.70663975008113
H	-3.88529398573501	1.15433256088317	-3.17395197558644
C	-4.67950636971291	-0.07923364057122	-0.64862242672722
H	-3.92266518249066	-0.60861659825635	-1.23033289429045
H	-5.53232356162190	-0.75619443703077	-0.56458910240578
C	-4.15332997693226	0.22270407711824	0.75007557172971

H	-3.34608883897947	0.94633967162512	0.69833584569340
H	-4.94993640333078	0.67002395143000	1.34470962343871
O	-4.71327880185433	-3.14557186243849	1.50695920232003
O	2.69993031622086	-1.49704037217145	2.08182102258614
O	0.42016295915672	-0.63014850610611	-1.79039261624382
O	-1.80756301670848	1.08935227374686	-2.10796168118424
O	-1.26343312445365	2.73839462700652	-0.25508328128875
O	1.10042035389195	2.22989641364490	-1.98403162110212
O	1.29657202039163	1.20586724773488	0.31745286059607
H	3.94563014666803	3.65570912058133	-1.31521089181709
H	-2.80611676572236	4.80172022035160	-0.15187991209020

***cis-cis-C-fac* [Ti(AB)]**

Ti	-0.06928259199229	1.36649136704296	0.39924974947730
N	-2.35651496709421	2.50578464971768	-0.77458118080349
C	-4.43112591175950	-2.26313369819521	-0.08306638772395
H	-4.95137778388204	-2.76944054340739	0.73466661815155
N	-3.00254849599033	-2.32970987918218	0.16894942946335
H	-2.49749359093717	-2.85483790222668	-0.53183138478327
C	-4.73672489614288	-3.06590545454332	-1.36829364385994
O	-5.94303084257380	-3.17259882316758	-1.66657460359098
C	-2.39571801467813	-1.92518012905041	1.28545903772065
O	-2.97453354775671	-1.30268673310145	2.17632252663194
C	-1.95405949338615	3.46314408018445	0.02748951609562
C	-2.66086289599267	4.77011448028924	0.10759859830268
H	-2.68798395386306	5.25571290722068	-0.86690286020795
H	-3.68642763097772	4.62956978339046	0.44800447299481
H	-2.13163292632091	5.40421908674844	0.81195656481295
C	-0.94631839096331	-2.34203749642452	1.44432325993237
H	-0.60950795086210	-2.94605911064744	0.60524769720511
H	-0.91106195782696	-2.96623517381057	2.33817776736240
C	0.05026886809501	-1.19780076548230	1.62139394952467
C	-0.36389679445924	-0.21252203271331	2.73567734658753
C	1.45012421369939	-1.71818643369382	2.00069492771939
H	1.35275071902619	-2.47526999120823	2.77742934715483
H	2.02544569272647	-0.89103065018914	2.41426817751618
C	2.19990975087920	-2.32013373096741	0.83219956609539
O	1.80958482664951	-3.33804295722600	0.25986715030432
N	3.31425676208743	-1.67300839612121	0.48998535145777
H	3.64590072713993	-0.89222339394160	1.03906840306032
C	5.59204540534228	-1.37090214871185	-0.29578895243807
O	6.52541977254485	-1.70312761441186	-1.05082847735190
N	2.30066619138310	2.04148369445736	-0.98106616468679
C	2.62825640708298	2.21570059191185	0.27167578143677
C	4.01435558821090	2.52618405156440	0.69783189326501
H	3.97114351674416	3.09422786925671	1.62357376552187
H	4.54676006039111	1.58935178181020	0.88086450130333
H	4.55805529627520	3.08906080960039	-0.05637092753723
C	3.17109544186732	1.95813142713922	-2.14835566081281
H	2.51343947081455	2.08301044423080	-3.00558459269535
H	3.85936548984691	2.80085736523736	-2.12569741331439
C	3.92433696989743	0.63782316403238	-2.19740356471544
H	4.56559984366013	0.64860138563559	-3.08169140198722
H	4.58733939403923	0.59904538981559	-1.33485895342697
C	3.00904402700814	-0.58023391816799	-2.22304861514206
H	2.21541771041154	-0.45738932625412	-1.48554709382000
H	2.51228641982914	-0.63084870664710	-3.19520060767165
C	3.70751659946267	-1.90809697972108	-1.96141281887960
H	2.99786064267648	-2.71113450481024	-2.16011272411573
H	4.54706114635742	-2.04616168379472	-2.64370379722118
C	4.24597848735555	-2.09465264055520	-0.53255058172330
H	4.45081501839294	-3.15943388254644	-0.40085880242206
C	-4.60401824701920	1.50999411389955	-1.04459628930754
H	-4.72053601416847	1.74661149443216	0.01620747811897
H	-5.55456458678805	1.75639579464696	-1.52165030796759
C	-3.54189774023244	2.44206909621219	-1.61426274578461
H	-3.92030439732626	3.45718182979638	-1.69658557599199
H	-3.21809354338032	2.12172253533160	-2.60516314693021

C	-4.29502263013202	0.02245247531418	-1.21517279053557
H	-3.22235915602376	-0.12783575235273	-1.13297098074328
H	-4.58140679789994	-0.29879469553565	-2.22004755014212
C	-4.97851727098114	-0.83780066497598	-0.16597838974712
H	-4.84828589287498	-0.37306766097802	0.81062987130333
H	-6.05044712309753	-0.90821940722213	-0.35344283222624
O	-3.76326094459318	-3.54011936280075	-1.99662363518540
O	5.63867412291576	-0.52265597371798	0.62597250104034
O	0.15032108063648	-0.44465568270144	0.43965760404428
O	-0.54128789444863	0.99639019316914	2.28982374071687
O	-1.63132580529855	1.36181081250564	-0.67935514572250
O	-0.92975989462379	3.21648218126209	0.73302960843251
O	0.99009089113959	1.74404827188471	-1.18512189095825
O	-0.43528254813087	-0.54118005026358	3.90115904603090
O	1.69378656988933	2.07546082372825	1.12800724338845

***cis-trans-C-mer* [Ti(AB)]**

Ti	0.21201033736506	0.89137944317744	0.18739253283092
C	-1.86131767533361	2.41192372826871	1.37413924573490
C	-4.02373806356260	-2.15740932069917	-1.26846106530983
H	-4.67369634047374	-3.00613892339615	-1.06198791462357
N	-2.82743054001244	-2.26269242529081	-0.45719108040301
H	-1.99416102722022	-1.90290345090901	-0.89847936484402
C	-3.63271483177082	-2.20115043890236	-2.75912197971645
O	-4.58061082049179	-2.32306951159960	-3.56301397548434
C	-2.82388002100965	-2.49131412770538	0.85305854672021
O	-3.82341703394640	-2.85290866499206	1.47768506435861
N	-2.17666592356657	2.41044455689058	0.10953823120086
C	-3.42846574314419	2.77983249396807	-0.54448709339866
H	-3.15136447503818	3.05368332717421	-1.56042934074966
H	-3.82245192004581	3.66633850627729	-0.05412696431358
C	-1.54167948387644	-2.24937562073724	1.63195650137096
H	-1.34795480517423	-3.13739149240953	2.23175647980153
H	-1.80170609017441	-1.45439635502433	2.33481147663540
C	-0.25634525151776	-1.84176221768030	0.89849592671594
C	0.71262947639809	-1.28567858795860	1.95805691892270
C	0.37875184784046	-3.03647145209102	0.17174741725051
H	-0.36328476268241	-3.50189794921432	-0.47299165316612
H	0.67435039809272	-3.76055296472025	0.93103248094486
C	1.56916603872224	-2.68729522467110	-0.70283597701739
O	1.45842114210773	-2.64482809835990	-1.92800190038205
N	2.71802406042927	-2.44223483280598	-0.06622596790119
H	2.83940543716396	-2.65103901828315	0.92235379636946
C	5.31736990995219	-2.17346808800152	1.44506278650221
O	6.48479299442778	-2.04586474761947	1.87134087643473
N	2.61977222745194	1.83019183299999	-0.97945359836136
O	1.89761891646005	1.86580991878387	0.16840914881457
C	4.03921687929654	2.15573762080727	-0.86350445723878
H	3.48923539053685	1.70171109544449	-3.46061551582396
H	4.12683099720218	2.74943831977299	0.04291169248018
H	-3.04809703711400	2.13728362963711	3.08347071027722
O	0.86704091862950	0.74415008884419	-1.71456201350551
O	1.10682286144442	-1.95984771074391	2.88954400836841
C	4.89067309500060	0.89602204495605	-0.80533956501545
H	4.76246038707795	0.35312830113396	-1.74335388935719
H	5.93943806164590	1.19756582624067	-0.77013185624034
C	4.57314760954616	-0.02143755277661	0.37390519764390
H	5.08039012157584	0.35677216635451	1.26290341839332
H	3.50628890747724	-0.00364402915616	0.59692432860223
C	4.99362266899744	-1.47522263347407	0.11220394597498
H	3.76656094296974	-1.63407315735979	-1.65721731969724
H	5.92830123654149	-1.45909478251241	-0.45125599876498
C	3.97331819269008	-2.21111851277076	-0.75692733849985
H	4.39529859919393	-3.16656451392364	-1.08325980225644
C	-4.44027794032983	1.64158368866756	-0.52907050865404
H	-5.34516108224661	1.99419179295741	-1.02935573312600
H	-4.71413595651460	1.43268398384470	0.50843057474174
C	-2.73379408553485	2.95907754215651	2.43977029844412
H	-2.14839459679066	3.65254834712693	3.04212922892507
H	-3.61139286486146	3.46367121761898	2.05068447934471
C	-3.91221144466809	0.37412787490306	-1.18254688916966
H	-2.92220093243679	0.17242396787639	-0.77922989158471

H	-3.77546228092075	0.54383791397264	-2.25376758845603
C	-4.77682307148238	-0.85653132351464	-0.94966767626691
H	-5.08212788203068	-0.89307576915631	0.09792886448106
H	-5.68553556764907	-0.82152401334373	-1.55165151244193
O	-2.42006871606879	-2.08197486437507	-3.04186056219998
O	4.39023161483796	-2.78669601896258	2.03485513882521
O	-0.52249112468665	-0.78278364753518	0.01824977815192
O	1.03186603470145	-0.05108421234398	1.74453066748500
O	-0.73323775726723	1.88727255431805	1.67037917571273
O	-1.22474870115084	1.87023107474178	-0.69419059766430
C	2.01518933040064	1.22663421412974	-1.96992530153643
C	2.60950751559912	1.08192072836907	-3.32085271559546
H	4.32084587663634	2.78461356001799	-1.70583670856921
H	2.87531213128504	0.03607082989463	-3.47721881609476
H	1.85163869109668	1.34967006369325	-4.05522280502978

***cis-trans-N-mer* [Ti(AB)]**

Ti	-0.17365402563721	1.28889950983520	0.72663092189888
N	-2.63254014098722	2.58178808295564	0.53488316096639
C	-4.18765263040637	-1.80781006340159	-0.63073198089933
H	-4.84353496163437	-2.39183217642589	0.01866616337531
N	-2.81673507986419	-2.05150096757097	-0.22435093701515
H	-2.22875680849958	-2.39457954764092	-0.97111060382563
C	-4.37594242981512	-2.33490558260591	-2.06937753612119
O	-5.54649603849515	-2.32909340114169	-2.50154131172158
C	-2.38454712224579	-2.06040207117029	1.03738849594878
O	-3.08632076365701	-1.71743103566600	1.99046693673046
C	-1.88393189729360	3.51840151743895	1.07083767976959
C	-2.38182783107706	4.90836226001497	1.25829378118658
H	-3.20673780926070	4.92437728948687	1.97060190769646
H	-1.56739432873987	5.51779090555432	1.63722964537593
H	-2.73714376062716	5.32398227270031	0.31650484925093
C	-0.96698662979932	-2.55378555008167	1.24864301963174
H	-0.63447172743318	-3.14742779167853	0.40042291377447
H	-0.96878354580404	-3.18488096921807	2.13608968203024
C	0.05512561806768	-1.43802373976919	1.45051356564083
C	-0.14523803657348	-0.64304266061570	2.76341809400778
C	1.48301642729170	-1.99328800996709	1.58471177395374
H	1.45547781521362	-2.82528933434252	2.29098013944733
H	2.11121226476595	-1.22382464828423	2.03374396921383
C	2.17412724290000	-2.48251384839406	0.32506209708753
O	1.58717108142390	-3.02050461814064	-0.61074955281295
N	3.49676709425831	-2.29667539688642	0.35827120507067
H	3.93510410538654	-1.91923211408486	1.18834878840869
C	5.83925901987439	-2.86253876249494	0.09920291214886
O	6.74983384765171	-3.41890675799099	-0.54536316498905
N	2.07109149119158	2.14082142825928	-0.78135655991804
C	1.07517590043645	2.27198714319453	-1.62901643769332
C	1.28177737655167	2.64975020150894	-3.04893717897369
H	2.28819946277017	3.00078065073130	-3.25035800208495
H	1.06829512806712	1.78382297688855	-3.67635597954527
H	0.56457383505048	3.42974936023053	-3.29944668614053
C	3.43501363192774	1.73915467129190	-1.12165834900356
H	4.02709445120114	1.88756110113895	-0.22144926199312
H	3.82097123845024	2.39955213757379	-1.89461092737198
C	3.40188274308900	0.27991473127189	-1.55133798732131
H	2.75799037321315	-0.24298014114793	-0.84845339856158
H	2.91707522262354	0.21340320683676	-2.52854260460362
C	4.74468595006482	-0.42827200320129	-1.61475956715606
H	5.35462059140100	0.01372851790276	-2.40520135298015
H	5.28999793232002	-0.27353725301810	-0.67966734738607
C	4.56449690831285	-1.92834972281543	-1.87427176464249
H	3.67172815834162	-2.08376941839193	-2.48295217856181
H	5.40578344271034	-2.32373587401158	-2.44114443709644
C	4.46166881390357	-2.78468330769211	-0.60483959598720
H	4.18871096932537	-3.79924344578540	-0.89982396089075
C	-4.25402143658545	2.07246963813652	-1.28515604841987
H	-3.68457296551276	2.67599379407102	-1.99531450325909
H	-5.31052600105814	2.25349391224550	-1.49965749008138
C	-4.02272061376492	2.62426419533818	0.11708552392041
H	-4.60419512566412	2.07547411475274	0.85831388042917
H	-4.33546415228652	3.66392115340954	0.15602546389798

C	-3.94651549289545	0.59076268364221	-1.51673894606196
H	-2.87045607083280	0.43284094913924	-1.50473100254939
H	-4.29176233270685	0.33964515667534	-2.52254683341201
C	-4.60712248201167	-0.34057684288849	-0.51162674230740
H	-4.37471804368751	-0.02871219996023	0.50406706351706
H	-5.69215346424966	-0.29328178680927	-0.62276309231835
O	-3.35179762995274	-2.70803715329206	-2.68500617230096
O	5.93148435140350	-2.35504228626718	1.24030097068760
O	-0.03908966354697	-0.49857618883704	0.41406269196793
O	-0.05261671707066	0.64799591279655	2.57422726054003
O	-2.06189227755376	1.35324837777454	0.51346487777753
O	-0.71288576176900	3.17890792560234	1.42288518624361
O	1.68652144123224	1.77091154551972	0.47407611022794
O	-0.28923778360783	-1.17278069851588	3.84102025564583
O	-0.09262034781418	2.05604004628717	-1.18276149146286

***cis-cis-C-fac* [Ti(AB*)]$^{1+}$**

Ti	-0.26167968680386	0.89307888877446	-0.05903216852143
N	-2.34102145949184	1.64190080593498	-1.78938156978471
C	-4.25675074548961	-0.73943906979741	1.47419404877487
H	-4.08960158964355	-0.20768215693872	2.41252957028007
N	-3.13781227263934	-1.63241230100070	1.22364288113918
H	-3.43148814329746	-2.60305497007448	1.14733551047776
C	-5.51567295107920	-1.62090009691498	1.66661842987960
O	-6.55909516339126	-1.01237078935504	1.96365943668848
C	-1.89376957055250	-1.29599703124882	0.99001959837418
O	-1.54187627894015	-0.06545035321950	1.09611996478484
C	-2.33900444901369	2.61247991868456	-0.90764393516857
C	-3.32075692757501	3.72169287950392	-0.94363956125322
H	-3.85544246224269	3.77652864527444	-1.88636303594934
H	-4.03587745851985	3.58513763528751	-0.13155378856512
H	-2.78489523915015	4.65283880396913	-0.76853336498072
C	-0.94130377045743	-2.31570762982877	0.63449684524029
H	-1.19854569263842	-3.34698050652483	0.82258380794658
C	0.25807737374803	-2.00376607729843	0.10188809512923
C	1.33698232129960	-3.03444129794167	-0.07137355690374
H	0.88722422967620	-4.00737007953085	-0.26182245547163
H	1.87423171115680	-3.09951477022760	0.87712442397817
C	2.29725490901965	-2.71067947830463	-1.20029205864441
O	1.96249070857014	-2.85411695815179	-2.37503411032332
N	3.49391614988376	-2.27769308288086	-0.81266933250077
H	3.73302410317235	-2.23260298556813	0.16957454146777
C	5.85678974130878	-1.73352314525186	-0.77643846000400
O	6.91471119472113	-1.45437328356504	-1.37193529668781
N	2.25134785961744	2.04281628260099	0.16053052838484
C	1.89084933287161	1.89817987327610	1.41125693666940
C	2.82629872309940	2.14188911216129	2.53833272046455
H	2.27188738475664	2.06892225842664	3.46873087311325
H	3.61812142656564	1.39235675913070	2.53322579435806
H	3.28658456810625	3.12538449692271	2.46137869430403
C	3.55879868806812	2.34956160658611	-0.39921538931213
H	3.38397135587077	3.05654070778300	-1.20984460353270
H	4.13031834582789	2.85572722913017	0.37454220498623
C	4.29472873635382	1.11154584381607	-0.89205962076267
H	5.28759052460652	1.43843787707224	-1.21120277147628
H	4.44200250057764	0.43533968465172	-0.04842874109294
C	3.59197064393155	0.39630923428223	-2.03768321278509
H	2.61415750105891	0.05530355221187	-1.70364132649921
H	3.40769759279806	1.11685831549696	-2.83908791289304
C	4.34936670321856	-0.78264758793013	-2.62985235718558
H	3.77612561239192	-1.15352469159529	-3.47883981216284
H	5.31966154770714	-0.46421294968657	-3.01145071134879
C	4.61236215223295	-1.95446066885940	-1.67433484447498
H	4.84692140101416	-2.82807513089762	-2.28883493366802
C	-4.68177162207663	0.79824057818905	-2.12649598941744
H	-5.23024973634400	1.65155869891263	-1.72285871506419
H	-5.28140832769240	0.41873429450520	-2.95515740558366
C	-3.37632587631299	1.27802482097621	-2.75619655852893
H	-3.55369780277742	2.13133854838218	-3.40852429484144
H	-2.92368787340709	0.49108727855687	-3.35343516620482
C	-4.54449645129303	-0.28054913820696	-1.04519402248888
H	-3.67320865555980	-0.90299758190429	-1.25917633843179

H	-5.41099860343988	-0.94226636224600	-1.09726861267312
C	-4.45624631785982	0.29531002181789	0.36552116422411
H	-3.65163900969600	1.02023087818510	0.43640678159774
H	-5.37838083736222	0.82788767713323	0.59684773309852
O	-5.37347191786863	-2.85390244820367	1.50741601325960
O	5.70609948788438	-1.87111362427372	0.45930200749589
O	0.58954411720729	-0.77853533743648	-0.24341447197978
O	-1.34174781519004	0.73319772051869	-1.61134826635460
O	-1.45068838350924	2.55258943370938	-0.00324681077067
O	1.25710309602371	1.80906001557118	-0.73449888638321
O	0.68868134696841	1.52126120742878	1.61329586455841

***cis-cis-N-fac* [Ti(AB*)]$^{1+}$**

Ti	0.34988324523533	-1.20424461107215	0.06359642420546
N	-2.00933343512922	-2.11287043265162	1.27218503175561
C	-4.35259986838050	2.24210750434381	-0.43447846089545
H	-5.06738286639798	2.60649041781344	-1.17437920761020
N	-3.03426867063868	2.18807561267359	-1.03135456921087
H	-2.33296817044159	2.67873768735539	-0.49073544823312
C	-4.31488088305535	3.26820281019678	0.72129798259482
O	-5.40998008145992	3.51818412036106	1.26134876778642
C	-2.72644768505764	1.62183794173089	-2.19523505568378
O	-3.54443691791414	1.07754903209480	-2.93202256557347
C	-1.88703631705089	-2.89709921406728	0.23067717192138
C	-2.86164527357854	-3.96111848743319	-0.10350654299626
H	-3.59729182168936	-4.12389110410865	0.67645176241131
H	-3.37166893891568	-3.68476172774917	-1.02688840681479
H	-2.30989812772961	-4.88207214047879	-0.28607930110650
C	-1.25267386426977	1.71392313407370	-2.58274091117711
H	-1.02170506513362	2.74793286175350	-2.83677525026237
H	-1.11044557351023	1.09341065741464	-3.46502341329558
C	-0.34543434058789	1.23095571912811	-1.49218520658309
C	0.45060819078786	2.08017195750306	-0.79689892196435
H	0.47761465268620	3.12404056352090	-1.06850187533200
C	1.35962538147546	1.60300680651567	0.20083702894914
O	1.29987349211200	0.39700269056127	0.62091562432591
N	2.26295824275604	2.42993874861357	0.70226034550844
H	2.21432280073152	3.35755454025877	0.30823016304721
C	4.50304538850475	1.49035398349810	0.14058694609214
O	5.72651047868311	1.56801279866063	0.37965340892459
N	2.79185960680093	-2.30299349865935	0.74909289078742
C	2.87783898914157	-2.16531778978769	-0.54996352891120
C	4.14863205485630	-2.29506959246090	-1.29631972449811
H	3.94278450829301	-2.78418362934818	-2.24604771171195
H	4.51348568793680	-1.28459391175045	-1.49190157446775
H	4.90246429813852	-2.85118391275389	-0.74819152774734
C	3.85722582557924	-2.39235115173337	1.74333532335869
H	3.41000079791628	-2.88054744159693	2.60692034054412
H	4.63316286196601	-3.04565370727714	1.35203252864360
C	4.42021145398287	-1.01853035762929	2.08172239512775
H	5.27746290223913	-1.16420981873414	2.74273846638764
H	4.81813807059003	-0.59262354990306	1.16454570513043
C	3.40256266925057	-0.07991138781154	2.73660530317729
H	2.41226068419265	-0.28713250482830	2.35328968715611
H	3.36905656223847	-0.30690796751636	3.80521089960592
C	3.68339213058566	1.41590179883087	2.60298689073201
H	3.01115047928145	1.95188654573568	3.27617281759580
H	4.69518051239514	1.61875936322935	2.95881729707462
C	3.59660009999213	2.11739310223484	1.23658756362354
H	4.01947218696959	3.10473194870993	1.41337072698934
C	-4.27306352378232	-1.15889525818805	1.47893382615905
H	-4.62403554667010	-1.73249596492637	0.61724341470257
H	-5.11218637709267	-1.09683715770341	2.17494288475139
C	-3.15757037022767	-1.93841918957158	2.15858604090047
H	-3.49293816538586	-2.92207671535764	2.47883770926745
H	-2.77135666047144	-1.41608124087190	3.03073463821148
C	-3.84254531770841	0.22896264481846	1.03005315907708
H	-2.87394973971351	0.16208420165569	0.53539169254649

H	-3.69583306417189	0.86548259129820	1.90693367892747
C	-4.82296662260470	0.87316099755097	0.06490538741741
H	-4.96068358877652	0.21668050535224	-0.79600534438703
H	-5.79936853921888	1.00543974483832	0.53217842543848
O	-3.19921876621044	3.74928960106237	1.02440114188036
O	3.95824260450537	0.97925000166229	-0.85577179539846
O	-0.39621904175501	-0.05777745417192	-1.27934934476891
O	-1.01924899570495	-1.18742355069095	1.38289415193513
O	-0.86428342153103	-2.70099322007868	-0.50174814256178
O	1.53454056409015	-2.15164961077145	1.24264782942597
O	1.79045821805192	-1.87609533336752	-1.16103964290606

***cis-trans-C-mer* [Ti(AB*)]$^{1+}$**

Ti	-0.13866810193201	0.84733888658210	-0.64468113993200
C	-2.20200569347747	1.41085852836913	-2.46098793306398
C	-3.59126557917942	-0.75958906344968	2.00802876763623
H	-3.06335804612604	-0.48466606672091	2.92446842926441
N	-2.74987383002801	-1.67488705504257	1.24707635076877
H	-3.12831677322137	-2.61795284369884	1.23053331918339
C	-4.84294536713282	-1.56297359734301	2.44874035664544
O	-5.66175835702385	-0.94559135705760	3.15253395685358
C	-1.64170257451176	-1.38568952514310	0.60924243812764
O	-1.23482337624657	-0.17328306688806	0.62048212099373
N	-2.50770995482860	2.12576251296536	-1.41340488309765
C	-3.80280346070422	2.67975566557339	-1.01714961505560
H	-4.10582910258998	3.42391282707512	-1.75217843780938
H	-3.59539805305570	3.19863051018709	-0.08636443762316
C	-0.90025722265546	-2.40875763670567	-0.07657954653214
H	-1.18512196511206	-3.43991480389434	0.06261622919885
C	0.15631477682799	-2.09663159975263	-0.86010284520933
C	0.97823696697875	-3.13944633103954	-1.55048398518881
H	0.80480649306266	-3.05088747960541	-2.62516227621486
H	0.65876571739816	-4.13715736851580	-1.25268811791750
C	2.49085800516414	-3.06171046764989	-1.37646120621704
O	3.20457040581452	-3.75361254367018	-2.10116676359078
N	2.96495414828485	-2.25416934329118	-0.43334973804651
H	2.34898414004109	-1.70436172696567	0.14881280426173
C	4.48548729985387	-1.22500588002866	1.14780900228024
O	5.64171811819395	-1.08330478362862	1.59119615002126
N	2.34179322824199	2.14449218216035	-0.43731286421088
C	1.96292944601522	2.01575736891720	0.80507722003748
C	2.81039767210998	2.37985191774314	1.96086744979190
H	2.18129126430190	2.83907398741025	2.72053156270905
H	3.22931214144298	1.45330407205406	2.35851701269541
H	3.61744680289459	3.05350197026521	1.69079543611106
C	3.65873560615314	2.49700631906423	-0.96169117577323
H	3.48986805229856	2.79542744817523	-1.99405073195427
H	4.01585547119491	3.36708376288084	-0.41540071569940
C	4.63856870434874	1.33847462488899	-0.85604392317690
H	5.62025897678071	1.70583612926657	-1.16323154724139
H	4.73008922826283	1.06038833743887	0.19319597567134
C	4.24560395114800	0.13321765396939	-1.70607089064483
H	3.17249754834768	-0.04381288105157	-1.62551393454498
H	4.42835888494889	0.37905653810431	-2.75446217046727
C	4.98223549254888	-1.15935875891462	-1.35929581506107
H	4.98882164493974	-1.80936088158793	-2.23344780165699
H	6.02390026209466	-0.95448026104369	-1.10687928276338
C	4.36487052757853	-1.96280110524820	-0.20206899390234
H	4.91755526425990	-2.89831707070881	-0.11050016731337
C	-4.90087107076414	1.62693065257037	-0.84049874983093
H	-5.70740542743738	2.12025731188331	-0.29343450205130
H	-5.30900688855145	1.36901067431292	-1.81822725947419
C	-3.09544056674076	1.26653015858669	-3.63241563748394
H	-3.86392531630472	2.03283376166312	-3.66203103504952
H	-2.48520815721848	1.32057244504440	-4.53207863871762
C	-4.50177083024147	0.33726807173295	-0.12775362327250
H	-3.76250960013124	-0.19438878542478	-0.73093190537839
H	-5.38070552691445	-0.31119975121867	-0.10237711965893

C	-3.96894924815653	0.53625445026606	1.28195363492206
H	-3.10527672816906	1.19541291635281	1.26269216145776
H	-4.72926266737737	1.01410347678169	1.89994217343417
O	-4.91336517954803	-2.75539438023763	2.07366291975136
O	3.42415063278232	-0.81406332378789	1.67204147614023
O	0.54073708764603	-0.86348169786702	-1.09888083558006
O	-1.07191404584590	0.81069544699539	-2.42039807055639
O	-1.51777593763254	2.19493566945141	-0.48779552304305
O	1.41264827630181	1.73526969399873	-1.33827583528327
O	0.79776569382570	1.51530116926965	0.97937710042888
H	-3.56702328322881	0.28353429518183	-3.60235437309683

cis-trans-N-mer **[Ti(AB*)]$^{1+}$**

Ti	-0.23946109654353	0.88300820423952	-0.93668654169019
N	-2.71030329549779	1.99084268298583	-1.71915563131069
C	-3.64806531067564	-0.98138299215251	1.51001224597981
H	-3.21396334022588	-0.64157299770039	2.45264636187071
N	-2.67178204581681	-1.79981754070783	0.80763788303918
H	-2.99808776673435	-2.75812497669769	0.69662195643420
C	-4.83831758554090	-1.91048114610079	1.86519842031023
O	-5.77296152392851	-1.37752027003517	2.48764081785636
C	-1.53850872412522	-1.42588306455718	0.27758414764972
O	-1.13598949078631	-0.21305788975119	0.41464200707369
C	-2.33467909412044	2.77522150423540	-0.74051854577109
C	-3.17688362045229	3.88575238104255	-0.23715664778449
H	-4.01320871871018	4.10721929006240	-0.89174266839582
H	-3.55378789930474	3.61814775083896	0.75070094962672
C	-0.74294067150674	-2.38503581566662	-0.45291725532678
H	-0.88162286843575	-3.43511820304361	-0.24745583172100
C	0.14225622167015	-1.98840417173659	-1.38761909116227
C	1.08124685454596	-2.93414957267173	-2.05818947617101
H	1.12380681890401	-2.71366972625236	-3.12459355132204
H	0.73749747941760	-3.96127779244203	-1.94029587485663
C	2.52092326110907	-2.88848458413080	-1.54872647486702
O	3.38879464062560	-3.49313651003014	-2.17337894565781
N	2.75806291337261	-2.20031885712647	-0.43403957739662
H	2.00354139592268	-1.81780834308664	0.12162917434784
C	3.83307629438605	-1.56201065024834	1.65354952551098
O	4.86509763270021	-1.34181976154331	2.31665097446909
C	2.13916245438329	2.29727411571060	-1.39950010220516
N	2.32496468883185	1.77234534439629	-0.21926047090181
C	3.53342350914632	1.68426963692196	0.60563002043893
H	3.69882714545672	2.64745129227378	1.08842393499518
H	3.26213718635589	0.96322306899762	1.37058634871946
C	3.13678209089723	3.16005324774936	-2.07520481133737
H	3.75469128705313	2.55672196492091	-2.74141831774195
H	2.59731913131982	3.88808439791474	-2.67622712321990
C	4.79051552595881	1.24628918677684	-0.14364311348777
H	5.18407496867204	2.10044836003701	-0.69070205308666
H	5.53022650720063	1.02177378687523	0.62590194029387
C	4.66510887134801	0.05955467526640	-1.10958554475683
H	3.65385660860067	0.00173680658602	-1.51845319185587
H	5.30694726468144	0.27711977433432	-1.96404695141909
C	5.07798144507416	-1.31860847632730	-0.59338294069541
H	5.33328282815302	-1.93116891603060	-1.45738111429611
H	5.97797140890540	-1.23565105731760	0.01567929000118
C	4.04787491679285	-2.10471640131043	0.22153493442001
H	4.44901555593361	-3.11456432078632	0.34745749551366
C	-5.07988920569087	1.23607900703996	-1.41136207944147
H	-5.41195622065175	1.98273937013269	-0.68723898642318
H	-5.94139997062544	1.02728655850574	-2.04714444461520
C	-4.02953924944069	1.85148424049440	-2.33241522576374
H	-4.35167218586861	2.82843587966586	-2.68841800371047
H	-3.85829515170783	1.22057549607747	-3.20045919670121
C	-4.65432778640043	-0.03798913640708	-0.66966336067686
H	-3.92290959514919	-0.58557101368950	-1.26737217351486
H	-5.52170049414740	-0.69321406146501	-0.57227205519177
C	-4.10259496837648	0.24749136742089	0.72421915790025

H	-3.27286368737980	0.94559647951326	0.67603623530232
H	-4.87797775298968	0.72302404795338	1.32418933189719
O	-4.74902311140922	-3.10434536053616	1.50019292153448
O	2.65062719247554	-1.40722057688127	2.03326684898008
O	0.27296862209488	-0.72661121548623	-1.75226156349401
O	-1.80324474583686	1.03244489390771	-2.04662714007756
O	-1.19947335308242	2.52986641725293	-0.22141151455097
O	1.01472367337033	2.04213821140111	-1.95767688675303
O	1.25707586541783	1.08129261256133	0.25208500447030
H	3.77697032069245	3.67349640905290	-1.36429062650527
H	-2.54828205030813	4.76814693877356	-0.12996282277845

References

(1) Neese, F. The ORCA Program System. *WIREs Comput. Mol. Sci.* **2012**, *2* (1), 73–78. https://doi.org/10.1002/wcms.81.

(2) Neese, F. Software Update: The ORCA Program System—Version 5.0. *WIREs Comput. Mol. Sci.* **2022**, *12* (5), e1606. https://doi.org/10.1002/wcms.1606.

(3) Yanai, T.; Tew, D. P.; Handy, N. C. A New Hybrid Exchange–Correlation Functional Using the Coulomb-Attenuating Method (CAM-B3LYP). *Chem. Phys. Lett.* **2004**, *393* (1–3), 51–57. https://doi.org/10.1016/j.cplett.2004.06.011.

(4) Grimme, S.; Ehrlich, S.; Goerigk, L. Effect of the Damping Function in Dispersion Corrected Density Functional Theory. *J. Comput. Chem.* **2011**, *32* (7), 1456–1465. https://doi.org/10.1002/jcc.21759.

(5) Weigend, F.; Ahlrichs, R. Balanced Basis Sets of Split Valence, Triple Zeta Valence and Quadruple Zeta Valence Quality for H to Rn: Design and Assessment of Accuracy. *Phys. Chem. Chem. Phys.* **2005**, *7* (18), 3297. https://doi.org/10.1039/b508541a.

(6) Garcia-Ratés, M.; Neese, F. Effect of the Solute Cavity on the Solvation Energy and Its Derivatives within the Framework of the Gaussian Charge Scheme. *J. Comput. Chem.* **2020**, *41* (9), 922–939. https://doi.org/10.1002/jcc.26139.

(7) Cossi, M.; Rega, N.; Scalmani, G.; Barone, V. Energies, Structures, and Electronic Properties of Molecules in Solution with the C-PCM Solvation Model. *J. Comput. Chem.* **2003**, *24* (6), 669–681. https://doi.org/10.1002/jcc.10189.

(8) Barone, V.; Cossi, M. Quantum Calculation of Molecular Energies and Energy Gradients in Solution by a Conductor Solvent Model. *J. Phys. Chem. A* **1998**, *102* (11), 1995–2001. https://doi.org/10.1021/jp9716997.

(9) The PyMOL Molecular Graphics System.

(10) Gardner, S.; Carrano, C. J.; Mao, Y.; Küpper, F. C.; Cooksy, A. L. DFT and TD-DFT Studies to Elucidate the Configurational Isomers of Ferric Aerobactin, Ferric Petrobactin, and Their Ferric Photoproducts. *BioMetals* **2024**. https://doi.org/10.1007/s10534-024-00638-6.
